# Prediction of antifreeze proteins using machine learning

**DOI:** 10.1038/s41598-022-24501-1

**Published:** 2022-11-30

**Authors:** Adnan Khan, Jamal Uddin, Farman Ali, Ashfaq Ahmad, Omar Alghushairy, Ameen Banjar, Ali Daud

**Affiliations:** 1 Qurtuba University of Science and Technology, Peshawar, Khyber Pakhtunkhwa Pakistan; 2 Department of Elementary and Secondary Education, Peshawar, Khyber Pakhtunkhwa Pakistan; 3 Department of Computer Science, Abdul Wali Khan University Mardan, Mardan, Pakistan; 4 Department of Information Systems and Technology, College of Computer Science and Engineering, University of Jeddah, Jeddah, Saudi Arabia; 5 Abu Dhabi School of Management, Abu Dhabi, United Arab Emirates; 6 Department of Computer Science and Artificial Intelligence, University of Jeddah, Jeddah, Saudi Arabia; 7 Sarhad University of Science and Information Technology, Mardan, Pakistan

**Keywords:** Biochemistry, Biophysics, Computational biology and bioinformatics

## Abstract

Living organisms including fishes, microbes, and animals can live in extremely cold weather. To stay alive in cold environments, these species generate antifreeze proteins (AFPs), also referred to as ice-binding proteins. Moreover, AFPs are extensively utilized in many important fields including medical, agricultural, industrial, and biotechnological. Several predictors were constructed to identify AFPs. However, due to the sequence and structural heterogeneity of AFPs, correct identification is still a challenging task. It is highly desirable to develop a more promising predictor. In this research, a novel computational method, named AFP-LXGB has been proposed for prediction of AFPs more precisely. The information is explored by Dipeptide Composition (DPC), Grouped Amino Acid Composition (GAAC), Position Specific Scoring Matrix-Segmentation-Autocorrelation Transformation (Sg-PSSM-ACT), and Pseudo Position Specific Scoring Matrix Tri-Slicing (PseTS-PSSM). Keeping the benefits of ensemble learning, these feature sets are concatenated into different combinations. The best feature set is selected by Extremely Randomized Tree-Recursive Feature Elimination (ERT-RFE). The models are trained by Light eXtreme Gradient Boosting (LXGB), Random Forest (RF), and Extremely Randomized Tree (ERT). Among classifiers, LXGB has obtained the best prediction results. The novel method (AFP-LXGB) improved the accuracies by 3.70% and 4.09% than the best methods. These results verified that AFP-LXGB can predict AFPs more accurately and can participate in a significant role in medical, agricultural, industrial, and biotechnological fields.

## Introduction

AFP (Antifreeze protein) is essential for various species like animals, fish, plants, and microorganisms living in highly cold regions^1^. In ice recrystallization, small ice crystals bind with adjacent water molecules and form large ice crystal^2^. This ice recrystallization phenomenon is hazardous for cold-blooded organisms due to the formation of ice in their bodies. AFP interacts with small ice crystals and prevents or retards the ice recrystallization progression that leads to the survival of the cold-blooded living organisms in subzero and low-temperature environments^3^. AFP has other diverse significant applications including food preservation, human cryopreservation and cryosurgery improving, boosting freeze tolerance, ice and yogurt formation^4,5^. AFP possesses the characteristic of reducing the water freezing point without altering melting point. This property of AFP is called thermal hysteresis^6^.

In respect of above the significance, accurate identification of AFP is essential. A series of methods have been established for identification of AFP. For example, Kandaswamy et al. developed a method, called AFP-Pred to discriminate AFP from non-AFP. They used short peptides, secondary structure properties, physicochemical features, and RF (Random Forest) as training model^7^. In another approach (AFP-PSSM), these authors utilized evolutionary information in conjunction with SVM^8^. Yu et al. adopted multi-respective several composition features such as TPC, DPC, and AAC. They selected the best patterns via genetic algorithm and prediction was carried out by SVM. They also established a web server, called iAFP^9^.

Onward, Mondel et al. proposed AFP-PseAAC predictor employing PseAAC (Pseudo Amino Acid Composition) with SVM^10^. In another TargetFreeze protocol, the features are discovered by AAC, PseAAC, and PsePSSM, fused all patterns, and perform the prediction using SVM^11^. Pratiwi et al. adopted AAC, DPC, and physicochemical properties for feature engineering and RF as a classifier. Their novel predictor is called CryoProtect^12^. In RAFP-Pred predictor, authors split each protein sequence into two sub-sequences. Features from each part were abstracted by AAC and DPC. Info-Gain algorithm was implemented for selection of optimal features and the model was trained using RF classifier^13^. Usman et al. proposed AFP-LSE predictor. They used autoencoder with Composition of K-spaced amino acid pairs and achieved a balanced accuracy of 0.903^14^. In another work, Usman et al. constructed AFP-SRC improved method^15^. Similarly, PoGB-pred approach is developed by Alim et al. They employed PseAAC, AAC, and DPC as feature descriptors and PCA for reducing the feature dimension^16^. Recently, Miyata et al. designed a novel predictor using new datasets. They applied CD, DC, AAC for feature encoding and Light eXtreme Gradient Boosting machine for model learning^17^.

Although, each prediction system made efforts to predict antifreeze proteins. However, due to the variant behavior of AFP structure and sequences, it is still highly desirable to predict AFP more accurately. Considering this, we developed a protocol, named AFP-LXGB for accurate prediction of antifreeze proteins.

### Proposed method

In the design of AFP-LXGB predictor, we carried out the following contribution.

- Extracted the sequential patterns via GAAC, DPC, and evolutionary features by PSSM.
- To extract the local information, segmentation notion is extended into PSSM and split each PSSM into three segments. Further, the autocorrelation transformation (ACT) strategy is applied to each segment and finally combines all segments. Thus, a novel feature descriptor is introduced named Sg-PSSM-ACT.
- Developed another feature representative method named PseTS-PSSM. In this method, PSSM of the each sequence is decomposed into three slices. Further, the sequence-order patterns are computed using Pseudo strategy by extending to each slice and combined all the slices into one super set.
- Concatenated feature vectors into different groups and provided to RF, ERT, and LXGB for model training.
- A novel feature selection method namely ERT-RFE is introduced for the selection of optimal features.

**Figure 1:**
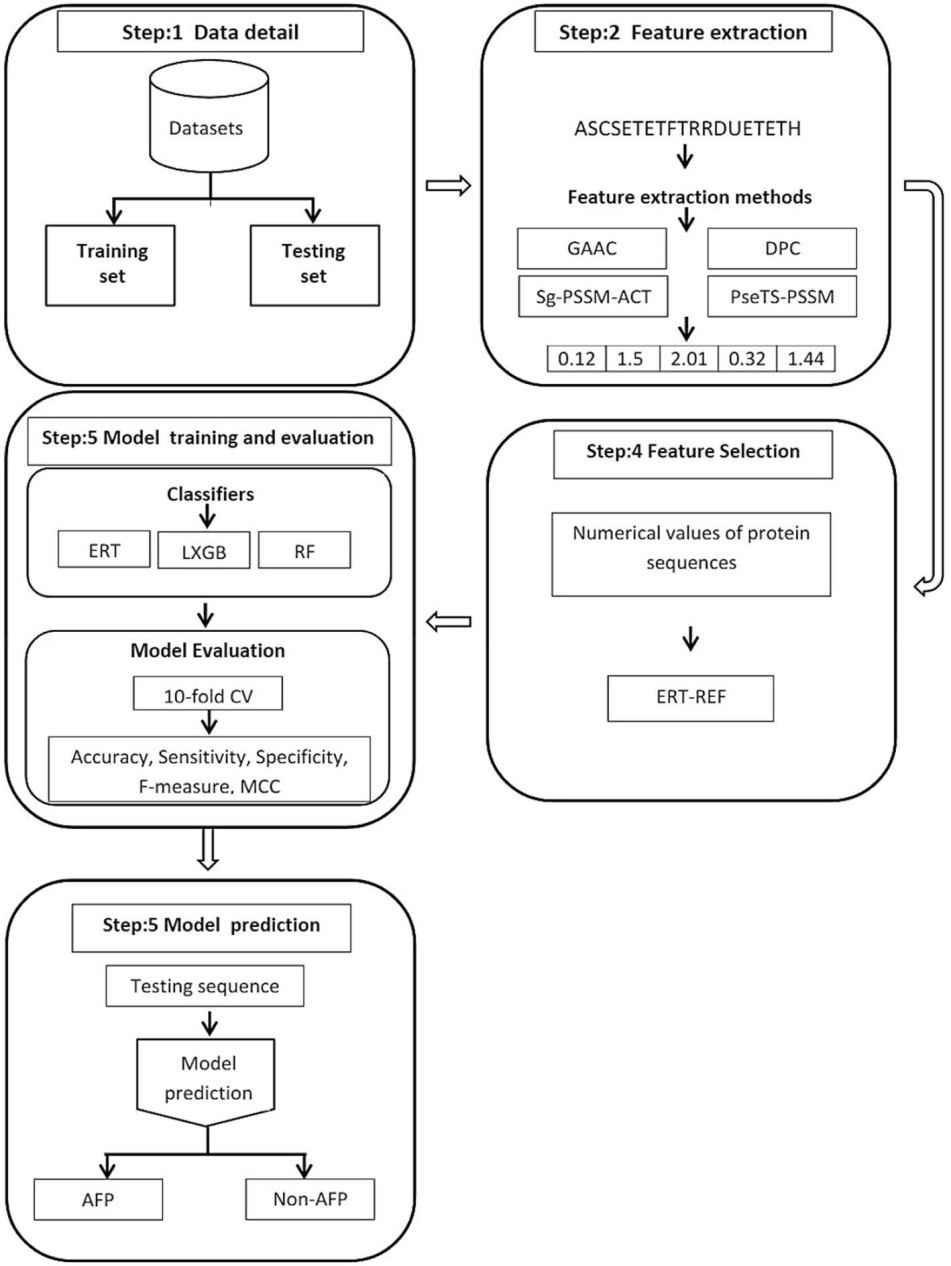
Pipeline of the AFP-LXGB.

The schematic view of the proposed work has been described in Fig. 1 and detailed in the following subsections.

## Materials and methods

### Benchmark datasets

To construct a promising method, we implemented datasets widely utilized by previous works such as AFP-Pred^7^, AFP-PSSM^8^, iAFP^9^, AFP-PseAAC^10^, and CryoProtect^12^. The AFPs (positive) set comprises 481 AFPs sequences. Similarly, the negative set containing 9193 non-AFPs instances was collected from Pfam protein families as explained in^7^. The datasets are provided in supplementary file.

### Feature formulation techniques

Discovering the discriminative features by appropriate schemes is an important step in the design of an effective computational model^20^. In this regard, GAAC, DPC, Sg-PSSM-ACT, and PseTS-PSSM are used for exploring the salient patterns from primary sequences of AFPs.

#### Grouped amino acid composition

The simple Amino Acid Composition (AAC) comprises 20 amino acids that compute the frequency of each amino acid^21^. GAAC classifies the 20 amino acids into five groups using the physicochemical properties. The five classes contain aliphatic group (G1: AGLIMV), negative charged group (G2: DE), aromatic group (G3: FWY), positive charge group (G4: HRK), and uncharged group (G5: CNPQST). GAAC calculates the frequency of each group using the following equation:

1 $$F\left(G\right)=\frac{n(G)}{n}, \quad G\in \left(G1, G2, G3, G4, G5\right),$$

2 $$n\left({G}_{a}\right)=\sum n\left(a\right),\quad a\in G,$$

where $n\left(G\right)$ represents the amino acids in a group $G$, $n\left(a\right)$ indicates the amino acid type $a$, and $n$ shows the length of sequence. GAAC extracts 5 features.

#### Dipeptide composition

DPC formulates frequencies of two connected amino acids of a protein sequence^22^. It explores the partial local information by computing consecutive sequence-order patterns and generates 400 (20 $\times$ 20) dimensional vector. DPC is formulated by following equation:

3 $$A\left(t\right)=\frac{n\left(t\right)}{C},$$

where t = 1,2,3,…,0.400, $n$ represents the dipeptide $t$, and $C$ indicates the total number of possible dipeptides.

#### Position specific scoring matrix

It has been reported that evolutionary information performs a crucial role in the construction of many predictors^23–26^. Considering this, the evolutionary features are explored by PSSM employing PSI-BLAST tool by aligning each protein sequence of the dataset with homogenous sequences in the NCBI^27^. The following equation is utilized for normalization of each PSSM.

4 $$f\left(t\right)=\frac{1}{1+{e}^{-t}},$$

where *t* represents each element of PSSM.

The PSSM can be denoted as:

5 $$PSSM={({A}_{1},{A}_{2} ,\dots .., {A}_{j}, \dots .., {A}_{20})}^{T},$$

6 $${A}_{m,n}=\left({A}_{1,n}, {A}_{2,n}, \dots .., {A}_{L,n}\right), \quad \left(m=1, 2, \dots ..,L\right),$$

where $L,$ $T,$ and ${A}_{m,n}$ show the length of sequence, transpose operator, and score of the residue in the *mth* position of query sequence replaced with residue of type *n*, respectively. The dimensional size of PSSM is 20.

#### Position specific scoring matrix tri-slicing

Recent studies have reported that local regions of PSSM contain more decisive features ^26,28,29^. To investigate these features, we incorporated the tri-slicing strategy into PSSM. We split the PSSM into three slices (parts) by row in equivalent dimensions. Each slice (S-PSSM) of the PSSM can be formulated as:first and second rank correlation

7 $$S-PSSM\left({\acute{\text{\AA}}}\right)={\left[\begin{array}{*{20}l}{P}_{b+\mathrm{1,1} } & \quad {P}_{b+\mathrm{1,2}} & \quad \dots . & {P}_{b+\mathrm{1,20}} \\ {P}_{b+\mathrm{2,1}} & \quad { P}_{b+\mathrm{2,2}} & \quad \dots .&\quad {P}_{b+\mathrm{2,20}} \\ \vdots & \quad \vdots & \quad \vdots & \quad \vdots \\ { P}_{b+N\left({\acute{\text{\AA}}}\right),1} & \quad {P}_{b+N\left({\acute{\text{\AA}}}\right),2 } & \quad \dots .& \quad {P}_{b+N\left({\acute{\text{\AA}}}\right),20}\end{array}\right]}_{N\left({\acute{\text{\AA}}}\right)\times 20},$$

where $\acute{\text{\AA}}$ indicates the number of $S-PSSM$ and $N\left(\acute{\text{\AA}}\right)$ rows in each $S-PSSM$, while $\left\lfloor * \right\rfloor$ operator shows the rounding down.

#### Pseudo position specific scoring matrix tri-slicing

PSSM computes the evolutionary features, however, avoids the correlation factors and sequence order patterns^30,31^. To cope with these limitations of PSSM, we extended Pseudo notion into TS-PSSM. Using Pseudo scheme, we calculated the sequence-order information from each slice and finally combined all three slices to make a super set^32^. The dimension of each slice (S-PSSM($\psi$)) can be expressed by following equation:

8 $$S-PSSM\left(\psi \right)= {\left[{R}^{A}, {R}^{C}, \dots ., {R}^{\psi }\right]}_{1\times 20},$$

where ${R}^{\psi }$ describes the corresponding residue type of 20 amino acids in a S-PSSM and $\psi$ is the number of slice. Mathematically, TS-PSSM is calculated as:

9 $$TS-PSSM={\left[S-PSSM\left(1\right), S-PSSM\left(2\right), S-PSSM\left(3\right)\right]}_{1\times 20}.$$

To calculate the PsePSSM (Pse) from each slice, the following formulation can be utilized:

10 $$Pse={[{\overline{R}}_{1},{\overline{R}}_{2}, \dots , {\overline{R}}_{20}, {\overline{R}}_{1}^{\pitchfork}, {\overline{R}}_{2}^{\pitchfork},\dots , {\overline{R}}_{20}^{\pitchfork}]}^{T},$$

11 $${\overline{R}}_{n}^{\pitchfork}= \frac{1}{L-\pitchfork}\sum_{m=1}^{L-\pitchfork}{[{\overline{R}}_{m,n}-{\overline{R}}_{m+\pitchfork,n}]}^{2} (n=1, 2,\dots , 20;\pitchfork<L),$$

where ${\overline{R}}_{n}^{1}$ and ${\overline{R}}_{n}^{2}$ are the first and second rank correlation factors, while $\pitchfork$ describes the correlation factor. TS-PSSM computes 60 features.

### Position specific scoring matrix-segmentation-autocorrelation transformation (Sg- PSSM-ACT)

Classifiers are unable to directly consider the correlation information of amino acids^33,34^. The correlation information is explored by encoding methods. We applied Sg-PSSM-ACT for consideration of correlation information. In Sg-PSSM-ACT, first, PSSM splits into three segments for extraction of the local region’s patterns^35^. Second, autocorrelation transformation (ACT) is extended into each segmentation in order to discover the correlation information regarding the amino acids of evolutionary features^30^. The ACT from the first, second, and third segments are computed by ${ACT}_{1}$, ${ACT}_{2}$, and ${ACT}_{3}$ using the following three equations.

12 $${{ACT}_{1}}_{n}^{lag}=\frac{1}{{S}_{1}-lag} \sum_{m=1}^{{S}_{1}-lag}\left({A}_{m,n}-{B}_{n}\right)\left({A}_{m+lag,n}-{B}_{n}\right), n=\mathrm{1,2}, \dots ., 20, lag=\mathrm{1,2},$$

13 $${{ACT}_{2}}_{n}^{lag}=\frac{1}{{S}_{1}-lag} \sum_{m={S}_{1}+1}^{{2S}_{1}-lag}\left({A}_{m,n}-{C}_{n}\right)\left({A}_{m+lag,n}-{C}_{n}\right), n=\mathrm{1,2}, \dots ., 20, lag=\mathrm{1,2},$$

14 $${{ACT}_{3}}_{n}^{lag}=\frac{1}{{S-S}_{1}-lag} \sum_{m=2{S}_{1}+1}^{S-lag}\left({A}_{m,n}-{D}_{n}\right)\left({A}_{m+lag,n}-{D}_{n}\right), n=\mathrm{1,2}, \dots ., 20, lag=\mathrm{1,2},$$

where ${B}_{n}, {C}_{n} \mathrm{and} {D}_{n}$ are the correlation factors between residues and $lag$ represents the differences between amino acids. This method 60-dimensional feature vector.

### Classification algorithms

To select an appropriate classifier for prediction of AFPs, we have used three classifiers namely RF, ERT, and LXGB. Among these classifiers, Light eXtreme Gradient Boosting (LXGB) has shown the best performance that has been elaborated in the following section.

#### Light eXtreme gradient boosting

Light GBM is implemented for model training and prediction. Light GBM was first introduced by Microsoft^15^. Compared with GBDTs, Decision Tree, and Random Forest, Light GBM has many advantages such as early stopping, bagging, regularization, multiple loss functions, parallel training, and sparse optimization^16^. Light GBM generates trees using leaf-wise strategy instead of level-wise which leads to a great decrease in loss^17^. The values of hyperparameters are provided in Table 1.

**Table 1:** Hyper parameters of the model.

Hyper parameter	Value
Max depth	8
Alpha	1
Era	0.1
Lambda	1
No. of estimator	500

### Feature selection algorithm

Past research works reported that selection of best features by an effective algorithm enhances the performance of a model^24^. Feature selection (FS) techniques are mostly utilized for solving diverse biological problems in Bioinformatics research field^36–39^. FS removes the less informative and noisy patterns from the original feature set. FS cope with overfitting problem and can boost the model performance^40^.

FS techniques are categorized into three classes: wrappers, filters, and embedded approaches^41^. Wrapper methods employ the classifiers to select the best features set. Filters examine the feature via information theoretic and correlation criteria. In embedded techniques, the classifiers first determine the important features by their coefficients and then select the best feature vector^42^. Extremely Randomized Tree-Recursive Feature Elimination (ERT-RFE) is embedded FS algorithm that evaluates the feature using ERT-based model and removes the less informative features recursively. Initially, the input features comprise a subset. In each turn, ERT model is constructed using the subset. The accuracy of the model is calculated and weight of each feature is computed due to its closeness to its target class. Based on weights, features are ranked and low-ranked features are eliminated from subset. When this process is completed, features with maximum accuracy are selected as final optimal feature set.

The feature selected from GAAC, DPC, Pse-PSSM-ACT, and PseTS-PSSM is 5, 88, 35 and 33, respectively. Finally, we attained 161 best feature set.

### Assessment methods for model evaluation

After designing a novel method, its efficacy is analyzed by appropriate validation methods^21,22,43–47^. tenfold is mostly used for assessment a model performance^48^. We examined the prediction results by tenfold cross-validation while the generalization power was assessed by independent dataset. Onward, Acc (accuracy), Sn (sensitivity), F-measure, Sp (specificity), and MCC (Mathew’s correlation coefficient) are employed as evaluation parameters. These indexes are expressed as:

15 $$Acc=(TP+ TN)/(TP+FP+TN+FN)$$

16 $$Sn= TP/(TP+FN)$$

17 $$Sp= TN/(FP+TN)$$

18 $$MCC= (TN \times TP)-(FN \times FP)/\sqrt{(TP+FN)(TP+FP)(TN+FN)(TN+FP)}$$

19 $$F-measure=2*(precision*recall/precision+recall)$$

20 $$Precision=TP/TP+FP$$

21 $$Recall=TP/TP+FN$$

## Results and discussion

This section illustrates the results of our implemented feature extractors with diverse classifiers. The performance analysis is explained in the upcoming sections.

### Results of classifiers using single feature encoder

The results of classifiers using each single feature set are reported in Table 2. RF achieved 69.83%, 76.16%, 86.50%, and 86.18% accuracies on GAAC, DPC, Sg-PSSM-ACT, and PseTS-PSSM, respectively. We can see that DPC features are informative which achieved good results. On Sg-PSSM-ACT, and PseTS-PSSM generated approximately same results however, these are better than GAAC and DPC. ERT yielded similar accuracies with RF using GAAC and DPC while enhanced the performance on Sg-PSSM-ACT and PseTS-PSSM.

**Table 2:** Results based on single feature descriptor.

Classifier	Feature descriptor	Acc (%)	Sn (%)	Sp (%)	F-measure (%)	MCC
RF	GAAC	69.83	68.34	71.32	68.97	0.40
	DPC	76.16	67.31	84.99	72.96	0.53
	Sg-PSSM-ACT	86.50	82.66	90.33	93.70	0.72
	PseTS-PSSM	86.18	79.40	96.13	84.66	0.73
ERT	GAAC	69.33	63.35	74.97	67.41	0.38
	DPC	76.36	65.66	86.96	73.35	0.54
	Sg-PSSM-ACT	87.83	81.66	93.99	86.83	0.76
	PseTS-PSSM	90.50	91.03	89.98	90.55	0.81
LXGB	GAAC	71.16	71.33	71.01	71.03	0.43
	DPC	84.00	83.33	84.67	83.67	0.68
	Sg-PSSM-ACT	88.02	85.32	89.98	87.59	0.76
	PseTS-PSSM	92.50	90.01	95.00	92.23	0.84

The best performance is secured by LXGB on all feature extractors. On GAAC, LXGB attained 1.83% and 1.33% higher accuracies than ERT and RF. Similarly, LXGB improved 7.64% and 7.84% accuracies more than ERT and RF with DPC. Compare with GAAC and DPC, all classifiers significantly boosted the performance over Sg-PSSM-ACT and attained accuracies of 86.50%, 87.83%, and 88.02% by LXGB, ERT, and RF, respectively. The better results of Sg-PSSM-ACT are due to several reasons such as PSSM explores the evolutionary profile, ACT considers correlation factors, and Sg computes the local patterns. The best performance is achieved by LXGB, ERT, and RF employing PseTS-PSSM on all five evaluation indexes. LXGB, ERT, and RF generated 92.50%, 90.50%, and 86.18% accuracies which are the highest outcomes among all feature encoding approaches.

### Performance of classifiers with heterogeneous features

Past studies have revealed that a combination of different features enriched the prediction models^36,49^. In this connection, we ensemble the features of different descriptors in various series combinations and summarized the results in Table 3. The accuracies showed by GAAC + DPC with RF, ERT, and LXGB are 78.83%, 78.50%, and 86.03%, respectively. We noted that integrated feature set achieved better prediction for AFP. Similarly, RF, ERT, and LXBG further boosted the performance with DPC + Sg-PSSM-ACT, which are 83.49%, 82.15%, and 91.31% in terms of accuracy. Onward, we analyzed the prediction results of classifiers over DPC + Sg-PSSM-ACT + PseTS-PSSM and “All feature set”. It is observed from Table 2 that all classifiers using “All feature set” attained remarkable performance with all assessment indexes. The accuracies secured by RF, ERT, and LXGB are 92.50%, 90.67%, and 93.67%, respectively.

**Table 3:** Results based on hybrid features.

Classifier	Feature descriptor	Acc (%)	Sn (%)	Sp (%)	F-measure (%)	MCC
RF	GAAC + DPC	78.83	73.66	84.00	77.49	0.57
	DPC + Sg-PSSM-ACT	83.49	76.67	90.31	81.87	0.67
	DPC + Sg-PSSM-ACT + PseTS-PSSM	90.33	85.32	95.43	89.65	0.81
	All feature set	92.50	88.00	97.00	92.14	0.85
ERT	GAAC + DPC	78.50	70.35	86.67	76.30	0.57
	DPC + Sg-PSSM-ACT	82.15	75.62	88.59	80.89	0.65
	DPC + Sg-PSSM-ACT + PseTS-PSSM	89.00	82.33	95.61	88.12	0.78
	All feature set	90.67	84.02	96.98	90.01	0.82
LXGB	GAAC + DPC	86.03	85.68	86.27	85.87	0.72
	DPC + Sg-PSSM-ACT	91.31	88.28	93.95	90.91	0.82
	DPC + Sg-PSSM-ACT + PseTS-PSSM	92.17	90.67	93.54	92.01	0.84
	All feature set	93.67	92.64	94.51	93.45	0.87

Among all classifiers, LXGB obtained the highest results on the training dataset using tenfold. LXGB improved the Acc, Sn, F-measure, and MCC by 1.17%, 4.6%, 1.31%, and 0.02 than the second best classifier (i.e., RF) with the same feature encoder (i.e., All feature set). From all analyses, we can conclude that fused feature set of All feature set greatly contributed to the identification of AFPs.

### Results analysis of classifiers on the best feature set

**Figure 2:**
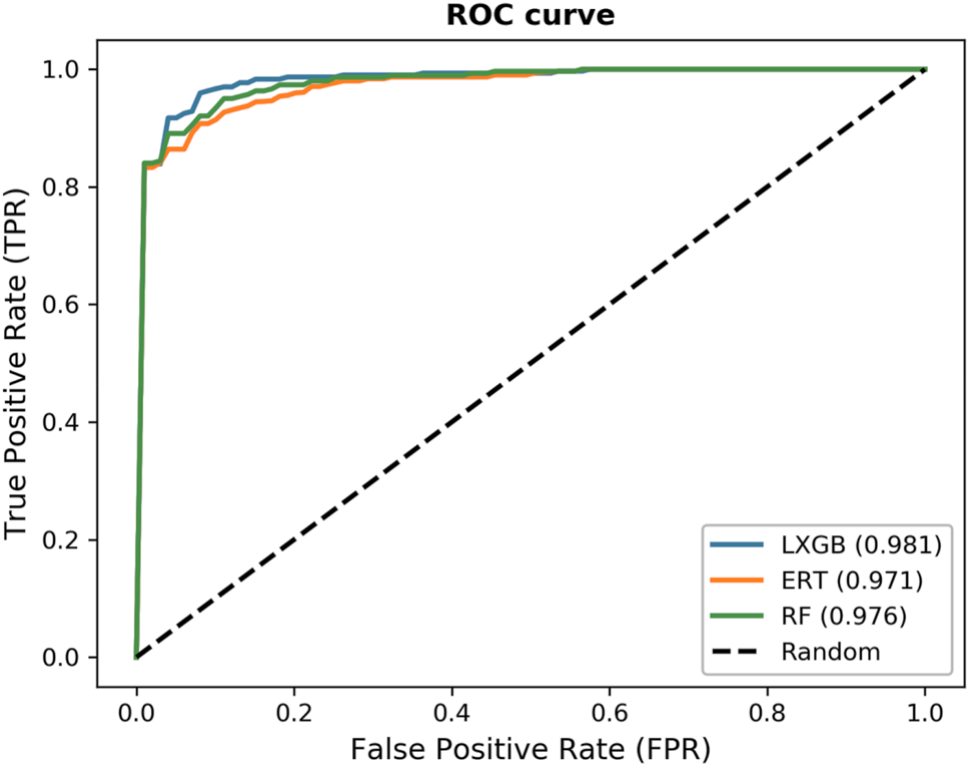
ROC curves of the classifiers.

Best features selection is a key step in the design of a predictor^50^. Many researchers applied the feature selection techniques and boosted the predictor performance^24,51,52^. During the process of feature selection, discriminative features are selected that can significantly boost the model performance. This study uses ERT-RFE technique for selecting the optimized features. From Table 4, we can see that RF with the best feature set produced an accuracy of 90.67%, sensitivity of 85.33%, specificity of 96.12%, and MCC of 0.81. Similarly, ERT reduced performance than RF and secured an accuracy of 90.00%. The LXGB shows outstanding performance over the best feature set and attained 94.00% accuracy, 93.00% sensitivity, 95.00% specificity, and 0.88 MCC. LXGB improved 3.33% accuracy than RF and 4% higher accuracy than ERT-based model. The results reveal that the best features effectively explore the local region features and sequence order information. Moreover, LXGB showed better performance mostly with individual, hybrid, and optimized feature sets than other classification algorithms. The ROC curves of classifiers are depicted in Fig. 2.

**Table 4:** Results based on the best feature set.

Predictor	Acc (%)	Sn (%)	Sp (%)	MCC
RF	90.67	85.33	96.12	0.81
ERT	90.00	83.01	98.99	0.80
LXGB	94.00	93.00	95.00	0.88

### Ablation study using imbalanced dataset

We performed an ablation study to check the effectiveness of the proposed study using imbalanced dataset. We distributed the dataset into 1:1 (300:300), 1:2 (300:600), 1:3 (300:900) ratios of AFP and non-AFP and the performance of the model with each ratio is observed. The results of the proposed work with different ratios of AFP and non-AFP are reported in Table 5. The performance of the model with 1:1 ratio is promising and found the best prediction results. Increasing the samples of the non-AFP i.e., using 1:2 ratio, the model reduced the points of accuracy, specificity, MCC, AuROC, and AuPR specifically sensitivity. The imbalanced samples of both classes show that it will not only overall performance of the model but greatly affect the sensitivity. Onward, analyzing the performance of the model using 1:3 (300:900), the model further decreased performance on all evaluation parameters. These results illustrate that on balanced dataset a model can perform better and produce effective results.

**Table 5:** Results with different ratios of AFP and non-AFP.

Ratio of AFP:Non-AFP	Acc (%)	Sn (%)	Sp (%)	MCC	AuROC	AuPR
1:1	94.00	93.00	95.00	0.88	0.9820	0.9883
1:2	92.46	87.76	94.43	0.84	0.9631	0.9740
1:3	91.33	85.91	93.89	0.83	0.9565	0.9591

The second ablation study is performed by applying a feature selection/reduction approach named Extremely Randomized Tree-Recursive Feature Elimination (ERT-RFE) to individual feature vector of GAAC, DPC, Pse-PSSM-ACT, and PseTS-PSSM. The features selected from GAAC, DPC, Pse-PSSM-ACT, and PseTS-PSSM are 5, 88, 35 and 33, respectively. All classifiers on each feature vector improved the performance. For instance, the accuracy of RF with GAAC before feature reduction is 69.83% and after applying the ERT-RFE is 70.11%. ERT and LXGB also enhanced the results on GAAC. Similarly, with reduced feature vector of DPC, Sg-PSSM-ACT, and PseTS-PSSM, all classifiers raised the accuracies.

Past studies have revealed that hybrid features enrich the predictor performance. In this connection, we ensemble the features of GAAC, DPC, Pse-PSSM-ACT, and PseTS-PSSM descriptors and make one super set of 161-dimension. The results are recorded in Table 4. The accuracy obtained by RF on “reduced all feature set” is 92.67%, while it is 92.50% accuracy before applying feature reduction. In the same manner, ERT and LXGB have also improved the results. It is concluded that reducing the feature vectors by an appropriate feature selection approach and then combining it all specifically raised the performance of a model.

### Comparative analysis with past work

We performed the comparison of the proposed system (AFP-LXGB) with the existing predictors like CryoProtect^12^, AFP-PseAAC^10^, AFP-Pred^7^, AFP-LSE^14^, and PoGB-pred^16^ on both training and testing datasets and summarized the outcomes in Tables 6 and 7. From Table 6, it is observed that our predictor yielded an accuracy of 94.00%, sensitivity of 93.00%, specificity of 95.00%, and MCC of 0.88, which are 3.70%, 6.30%, 1.1%, and 0.08 higher than the best method i.e., AFP-LSE. The proposed protocol also boosted the Acc, Sn, Sp, and MCC by 4.48%, 4.15%, 3.96%, and 0.08 are higher than the second-best method i.e., AFP-PseAAC. In the same fashion, our predictor surpassed other previous approaches on all four evaluation indexes. The efficacy of a novel model can be assessed by its high generalization ability. In this connection, we carried out the experiments on the independent dataset and it is noted in Table 7 that AFP-LXGB outperformed the previous methods in the literature.

**Table 6:** Comparison with existing predictors on the training set.

Predictor	Acc (%)	Sn (%)	Sp (%)	MCC
AFP-Pred	83.38	84.67	82.32	0.66
AFP-PseAAC	89.69	88.89	91.00	0.80
CryoProtect	89.50	89.54	89.50	0.79
AFP-LSE	90.30	86.70	93.90	0.80
PoGB-pred	89.38	73.17	90.01	0.37
AFP-LXGB	94.00	93.00	95.00	0.88

**Table 7:** Comparison with existing predictors on the testing set.

Predictor	Acc (%)	Sn (%)	Sp (%)	MCC
AFP-Pred	77.34	91.16	77.04	0.23
AFP-PseAAC	84.75	85.08	84.74	0.27
CryoProtect	88.28	87.27	88.30	0.31
AFP-SRC	85.40	86.10	84.70	0.28
AFP- LXGB	92.37	79.56	92.63	0.35

**Figure 3:**
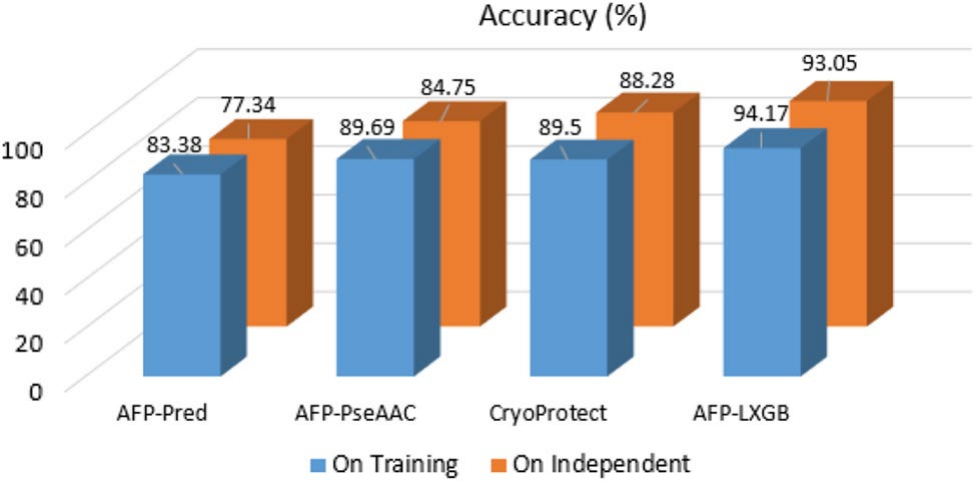
Accuracy comparison with the existing predictor.

On the testing dataset, AFP-LXGB achieved 4.09% (Acc), 4.33% (Sp), and 0.04 (MCC) higher than CryoProtect. Similarly, this work also enhanced the Acc, Sp, and MCC by 7.62%, 7.89%, and 0.08, respectively than second best predictor (AFP-PseAAC). The comparison has also been indicated in Fig. 3.

## Conclusion

In the current study, we established a novel predictor, called AFP-LXGB for antifreeze protein identification. It is a challenging job to explore the discriminative features of diverse and complex nature of AFP. To cope with this issue, we discovered the dominant information by PseTS-PSSM, Sg-PSSM-ACT, GAAC, and DPC. Further, we concatenated these feature vectors and applied ERT-RFE feature selection approach. The models are trained models with RF, ERT, and LXGB. After analyzing the performance of all models, it is concluded that AFP-LXGB has shown the best performance compared with the previous. The supreme achievement of the current study is due to several reasons such as effective feature coding approaches and appropriate classification algorithms.

In the future, we will apply more effective feature descriptors, feature selection approaches, and classifiers to further improve the performance of a predictor.

## Supplementary Information

Supplementary Information.

## Data Availability

The datasets used in this study has provided in the supplementary file and codes are provided at the link https://github.com/Farman335/AFP-LXGB.
