# Supplementary material for: Prediction of antifreeze proteins using machine learning
Source: Sci Rep. 2022 Nov 30;12:20672. doi: 10.1038/s41598-022-24501-1 (PMC9712683; doi:10.1038/s41598-022-24501-1)
Supplement: Supplementary file 1 — Supplementary Information. [file 41598_2022_24501_MOESM1_ESM.pdf]

>113927

MALSLFTVGQLIFLFWTLRITEANPDPAAKAAPAAVADPAAAAAAAVADTASDAAAAAAA

TAAAAAKAAADTAAAAAKAAADTAAAAAEAAAATARG

>210960

MKSAILTGLLFVLLCVDHMSSASQQSVVATQLIPINTALTPIMMKGQVVNPAGIPFAEMS

QIVGKQVNRAVAKDETLMPNMVKTYRAAK

>213510

MLAALLVCAMVALTRAANGDTGKEAVMTGSSGKNLTECPTDWKMFNGRCFLFNPLQLHWA

HAQISCMKDGANLASIHSLEEYAFVKELTTAGLIPAWIGGSDCHVSTYWFWMDSTSMDFT

DWCAAQPDFTLTECCIQINVGVGKCWNDTPCTHLHASVCAKPATVIPEVTPPSIM

>2315605

MRRQTTAIFVLLGLLAVFVVQGSTEDTGSTPTADNAPAASNGTAAPLSTANETSADPAGT

PEQAAEATVSSFTSIHSIFAASAVVLAHAL

>2411496

MSFKISTFTKIWLIIAVIVMCLCNEYNCQCTGAADCTSCTAACTGCGNCPNAITCTGSKN

CVRATTCTGSTNCNRATTCTNSKGCLEATTCTGSTHCHRATTCTNSKDCFEATTCTGSSN

CYTATTCTNSTNCYKATACTNSTGCPGH

>3004848

MKFSLVATIVLLALAQGSFAQGAADLESLGQYFEEMKTKLIQDMTEIIRSQDLANQAQAF

VEDKKTQLQPLVAQIQEQMKTVATNVEEQIRPLTANVQAHLQPQIDNFQKQMEAIIKKLT

DQTMAIEN

>3702803

MNIESSFCPILCICMIFLCLPNLSASQRCNNNDKQALLQIKTALKNPTITDSWVSDDDCC

GWDLVECDETSNRIISLIIQDDEALTGQIPPQVGDLPYLQALWFRKLPNLFGKIPEEISA

LKDLKSLRLSSTSLSGPVPLFFPQLTKLTCLDLSFNKLLGVIPPQLSTLPNLKALHLERN

ELTGEIPDIFGNFAGSPDIYLSHNQLTGFVPKTFARADPIRLDFSGNRLEGDISFLFGPK

KRLEMLDFSGNVLSFNFSRVQEFPPSLTYLDLNHNQISGSLSSELAKLDLQTFNVSDNNL

CGKIPTGGNLQRFDRTAYLHNSCLCGAPLPEC

>3876704

MIFLYSFIVALVAPQIGAIVEDFSCTTNTGATSKYKAGAFVCSNVISDAACKALYPVGGG

GDLTVGGDQERPFKCFSSATAAPAPIDADLKKVAIMYCPKTCGYCCETSAYSCENPPLPS

VNCSTIKPIQCTDPAWKAIIAMECPATCGLCSADSCVDAIEGCANNIEICNAVKLQDFVN

ANCRKTCKRCTSGPTATTSTAAPAAPTTTVAPAVVTTTAAPAAVTTTAAVTTTTVAAAAA

TTTAAACTSYIPDGRTRCGEYARSGFCTSNQYTTAEKRAACATTCKIC

>6434323

MGRKKIQITRIQDERNRQVTFTKRKFGLMKKAYELSVLCDCEIALIVFNSTNKLFQYAST

DMDKVLLKYTEYNEPHESRTNNDIMEALNRKEGNQGGGNSDDESPGPSTSPVIQITMPAV

VNGHSSNNSVASAASASAAAVAAAAAAVAAVEGTSSGAAAAAAALQASNAQRHHNLNLYQ

NLIFNPNYTRHLNQRNDPLSSTSVAPSSSSSKHLDFPPSTSFAYDTSRLHPIAAADADCD

LVPSSRAAADQNIWSSALQQRPVSQPAPSISNSSTNGISNGTSSLLSPNVSSLNGHSVLD

LGGPNLPYKLDPNTYVKMEPHSPPEKRPRITTEWRPQQLT

>9280346

LLLLNLLLLIRHCVVRCVTVNSFLIISVVLMFLCHEYYAVCTGGGNCSACTTACTNCVNC

PNALLACTDSTKCMKAVTCTRSTKCNKAVTCTNSSDCYKAVTCTGSTNCYKAKSCTGSTN

CYEATTACVNSTGCPP

>9280350

MVCQNFLLTISVVLMSCLCHEYYAQQCTGGSDCSSCTVACTDCQNCPNALIACTDSTNCY

QAMTCTRSTMCNGALTCTDSYDCFNADTCTRSTNCYTAKTCTGSTNCYEATACTDSTGCP

SSASVKQIK

>9280354

MVWVCKSSWLIIGVVLMCKYDECHSECTGGSDCRSCTAACTSCQNCPNARSACTGSTVCH

KAQTCTGSTGCYNAMTCTRSSECNNAQTCTGSHDCHNAQTCTGSYDCYNATTCTGSTNCY

RATTCTGSTNCHRATACTGSTGCPGSGA

>11181630

MKCLMLIMALAIINTVSSDGTCRNTNSQITNSKCVNSVPTNCYIDNSEVYGTTCTGSRYD

GVHITSSTTTGTSASGPGCTISSCTITGGVPAPSAPCRISGCTLRAN

>11181765

MEYFFLLIVLAIRTVSSDGTCVNTNSQITANSQCVKSTATNCYIDNSQLVDTSICTRSQY

SDANVKKSVTTDCNIDKSQVYLTTCTGSQYNGIYIRSSTTTGTSISGPGCSISTCTITRG

VATPAAACKISGCSLSAM

>11181767

MKCFMLIMALAINIVFSDGTCRNTNSQISANSLCASSTLTNCYIDDSQLESTTCTRSQYN

GAYVVSSRTTNCNIRNSQVHTTTCTNSQYEEGVYITSSTTTNTRVTGIACSVSSCTITRG

VVTPSNACRISGCILRAN

>11225528

MNFLKSFPFYAFLCFGQYFVAVTHADGSCTNTNSQLSANSKCEKSTLTNCYVDKSEVYGT

TCTGSRFDGVTITTSTSTGSRISGPGCKISTCIITGGVPAPSAACKISGCTFSAN

>12407647

MRGVVVVAMLAAAFAVSAHAEQCGSQAGGATCPNCLCCSKFGFCGSTSEYCGDGCQSQCN

RCGGTPVPVPTPTGGGVSSIISQSLFDQMLLHRNDAACLAKGFYNYGAFIAAANSFSAFA

TTGGTDVRKREVAAFLAQTSHETTGGWPTAPDGPYSWGYCFNQERGAPSDYCSPSSQWPC

APGKKYFGRGPIQISYNYNYGPAGRAIGTDLLNNPDLVATDATVSFKTALWFWMTPQSPK

PSSHDVITGRWSPSGADQAAGRVPGYGVITNIINGGLECGRGQDARVADRIGFYKRYCDL

LGVSYGHNLDCYNQRPFA

>12407649

MARFAALAALLLAVAVGGAAAQSVGSVITQSMYASMLPNRDNSLCPARGFYTYDAFIAAA

NTFPGFGTTGSTDDVKREVAAFFGQTSHETTGGTRGAADQFQWGYCFKEEINKATSPPYY

GRGPIQLTGRSNYDLAGRAIGKDLVSNPDLVSTDAVVSFRTAMWFWMTAQGNKPSSHDVA

LRRWTPTAADNAAGRVPGYGVITNIINGGLECGMGRNDANVDRIGYYTRYCGMLGTATGG

NLDCYTQRNFAS

>12838194

MLSLRSLLPHLGLFLCLALHLSPSLSASDNGSCVVLDNIYTSDILEISTMANVSGGDVTY

TVTVPVNDSVSAVILKAVKEDDSPVGTWSGTYEKCNDSSVYYNLTSQSQSVFQTNWTVPT

SEDVTKVNLQVLIVVNRTASKSSVKMEQVQPSASTPIPESSETSQTINTTPTVNTAKTTA

KDTANTTAGTTANTTANTTAVTTAKTTAKKPGHPHSRQPPWQVPSISCLFFSLVNSSSKE

NWGSRSPTSRSSSRAHFRPVLKHTRMKVLCPQSAAPPPWTTDLQH

>13345341

SSDGTCTKTNSWISPNSQCVRSTLTNCNVDNSQVYSTTCTNSRYNGIYITSSTTTGSRIT

GPGCSISHCTITRGSAAPAPACKISGCTLSAN

>21310268

MKFSLIAAVALLALAQGSFAQDAADLEKITQYFENLKNKMTEDVTAFLTNQDVANQAQTF

MQERKTQLEPLATQIQEQLRAAATKFEEHITPLAANVQPVVENFQQQMEALVQKLMEKTR

SISN

>21700301

MGSSHHHHHHSSGLVPRGSHMDTASDAAAAAAATAATAAAAAAATAVTAAKAAALTAANA

AAAAAATAAAAAR

>26328941

GRLAKFSEKLALAVTKGRGEERGDSSSVSHAADVSSLGKAAVPTACDDEDTLLTGDANSQ

HQVSGSEAGLSQEGRERLGDPVISPGVAEGPSPLQGPLEIREGEASLPGPESSDLGSGSK

MEATEDTGSMLRDPEGGATSPVDQEEEVDMDFLPQLSIEAMTVMRELTNLQLRKVCRYPS

PRTCAAELAALWGNVDEGSNRGALSPSSVRGKQGPEGALYPRGLGRGRAWGTPRRHTIGR

MVSSEGVHYPSSNPVSSDEFSDTHVMKGTAGLKEDQARSSGLTELDNIARHTNVRGRGNF

VHIPPSVLSSATWGVSSGMERQASGEQESSLAKKKPNMVWGKGSRPSHHGVTAAAATASA

AAPASASTASGALPKTSPKKKQAQEKTSSSDVSRGYQGKNLPTWGQRLKSAFEGPTTLPP

ISGVALLGKASKCSLPSGPKEFKSICTGKKSIAKKTREPQAGPKEDNSPRDPGLQAQVPP

HRAEQPSVCMYRGEMSRGDFNIRAPQVPANPQFFSLSQRCARPRAPPAPAGEQNQLMDPI

FPNEESQQVIHGTPGCAQCEMLQKEIDELKEQLGLALL

>26332695

MSAGSATHPAAGGRRSKWDQPAPAPLLFLPPTAPGGEVAGSGASPGGATTAAAPSGALDA

AAAVAAKINAMLMAKGKLKPSQNAAEKVLRALDALRFGIFFFLPFLHLSVAWSLGLMKMK

PVRVKITFGSI

>26333557

MQLLSLVQHGQGARKASKHAKATVATATTTAPPPPPTAPPVASQAAATAPPPPPDYHHHH

QQHRLTSNDNGGINREQPPASTPGDQRNSALLATLQARTNENTYAF

>26339980

MAAQAAAAAQAAAAAQAAAAQAAQAEAAESWYLALLGFAEHFRTSSPPKIRLCVHCLQAV

FPFKPPQRIEARTHLQLGSVLYHHTKNSEQARSHLEKAWLISQQIPQFEDVKFEAASLLS

ELYCQENSVDAAKPLLRKAIQISQQTPYWHCRLLFQLAQLHTLEKDLVSACDLLGVGAEY

ARVVGSEYTRALFLLSKGMLLLMERKLQEVHPLLTLCGQIVENWQGNPIQKESLRVFFLV

LQVTHYLDAGQVKSVKPCLKQLQQCIQTISTLHDDEILPSNPADLFHWLPKEHMCVLVYL

VTVMHSMHAGYLEKAQKYTDKALMQLEKLKMLDCSPILSSFQVILLEHIIMCRLVTGHKA

TALQEISQVCQLCQQSPRLFSNHAAQLHTLLGLYCVSVNCMDNAEAQFTTALRLTNHQEL

WAFIVTNLASVYIREGNRHQELYSLLERINPDHSFPVSSHCLRAAAFYVRGLFSFFQGRY

NEAKRFLRETLKMSNAEDLNRLTACSLVLLGHIFYVLGNHRESNNMVVPAMQLASKIPDM

SVQLWSSALLRDLNKACGNAMDAHEAAQMHQNFSQQLLQDHIEACSLPEHNLITWTDGPP

PVQFQAQNGPNTSLASLL

>26348120

MAQVAKKVHGSRAAAAAAAAAAKAKRSKPKTAAKRFKLNKKRNPRSKLPKRSHRSLIRSL

FQSRSCGCCRCCCGFCCYRSRPSRGRITFKFFQITKKGEQST

>26350641

CCLCSSRSPYSPVLRRSAKSRSRSPYSSRHSRSRSRHRLSRSRSRHSSISPSTLTLKSSL

AAELNKNKKARAAEAARAAEAAKAAEAAKAAEAAAKAAKASNASTPTKGNTETGASVSQT

NHVKEVKKLKTEHAPSPSSGGTVKSDKAKTKPPLQVTKVDNNLTVEKATKKTVVGKESKP

AATKEEPVSTKEKSKPLTPSTGAKEKEQHVALVTSTLPPLPLPPMLPEDKDADSLRGNIS

VKAVKKEVEKKLRCLLADLPLPPELPGGDDLSKSPEEKKTAAQLHSKRRPKYVLAFYWHL

G

>26388908

SSSGLPGHQACSSSRVPSGRSPRSGADVGRAGSSAAGRLLTATPAGRDSGAPGHGLRHLR

SRHSPGRAARAAARDAAHDTQPCGALASRHASPHARPRLRG

>30138722

MNKRVKPVLTYLLAALVIITIIVAWRVLRPAGLPAGIVAGNGRIEATEIDIATRAPGRII

EILAREGDFVQADRVLARMDTQVLRAQQIEAEAQVRRAEHAHQTAQSIVIQRQSEQTAAR

AVVAQRQAELDAARKRLQRTQMLAGEGASAQQELDDDQARVLSAQAAVSAAQAQVAAANA

SIEAAKSQVIESQSVIVAAQATVARLQADIDDSELRAPRDGRVQYRVAEPGEVLGVGGRV

LSMVDLSDVYMTFFLPAEAAGKVALGSDIHIVLDAAPGYVIPAKASFVASVAQFTPKTVE

TESERLKLMFRVRARIDPELLKQHLEQVKTGLPGMAYVKLDPAAVWPAELEVRLPQ

>30180978

MPIYEYACHACGLEKEHLQKMSDAPIANCPACGSSDYVKKVSAAGFQLKGTGWYVTDFRN

KNTRSDSKPKEESGKEAADTDKAAATTTTDSTTATATTASTSTTAPTVSSVD

>30249539

MRIEKKIKDHLVFSGDSILDALKRINDNQSRIVFVVQDNGVLIGAVSDGDVRRWMTQATE

FNLNLPVDHVMNRNFIARPVTESQHQIADYFDHKRDIIPLIDEQGRFVALARKSATGLQI

GDFLIADQNPAFIIAEVGNNHNGDIGLAKELVNLAVEAGADCVKFQMRDLSSLYSNQGRN

AEAGYDLGSQYTLDLLNKFQLNHDELCQVFDYCRQQDILPLCTPWDLVSAHVLDEYGLEA

FKVASADFTNYEMLETLAKTGKPLLCSTGMSSEAEIKGSVDLLRRLGAPFALLHCNSTYP

APFKDVNLNYLPHLKQLGGTVVGYSGHERGFSVPLAAVALGARIVEKHFTVDRSMEGNDH

KVSLLPEEFAEMVRQIRNIEEALGQGGERSLTQGEMINRENLAKSLVINCDLSQGQLIRR

SMITVKSPGQGLQPNRIDELAGKVAQRDFKAGDFFFETDITPKSVKKQHYVFSRPYGIPA

RYHDYRALIEGMKIDFVEFHLSYHDLDVKLSDYFSDPLSIGYAVHSPELFAGDHILDLAS

HDADYQAHSIAELKRTVAVAAELRQYFPATPKPVLVLNAGGWTPQNFLPVEARTKLYDKV

AKALDEIDLSTVQLAIQTMPPFPWHFGGQSHHNLFVDPDEIAAFCDKTGHRICLDISHSM

MACNYYQWDFNTFLQKVLPYTIHLHIVDAKGVDGEGVQIGHGDVDFTLLRDQLNQFARGV

QFIPEIWQGHKNKGEGFWSALAFLEKTSL

>31043802

MNLLIFLLCYFLGSPHYSEANKIKLKSSGRSADINHDSPCIWNKTQSWVHPYQSITVECC

DEELSKLIHKTILDAGNNAKLGNLAKFIQRRSQLFYHMSFETIVSRENFAISTHYHGTHS

CRVHDNNLYYLVYETPVQYDPFNMKTEDYLASIDSADPLGSTKPANLRGDFPDVREDSTA

GEDLSVQPSIGNILQYPNKFAWPDPDEEVSKKIMLAEQDAIRSINELMESVTEISVMNSG

AVVNSTDSLNITEIELPNPTPIPRNFANLREKDRLPENTHCDKERKDGNRCCDGRLASTM

RDAMRQMATSPDFGQGKEGIIAAELQQKVQQRFQKSYEIIVSQSDFVISTYTAGDNFCKF

DNKGFYILAYVSPKQYDIDEKEDEMKLAATSNKDPLGANTTMFENEAPWHVALKLDTYGD

RAGYPVGSHCTQARTGSKCCSLILFNAMKSGYDSHVATSNFDPYDIRNISKAVQWSVEEI

LQHSAEVIVSLDDFAYATYNNNSYTCKYRVDKYHILAYTTPNHDLDNYDEMATSIIDSEP

EAQIVYPAVDRTANQIPFQNTPSPYWTTPVPIPTQVYMPMQYTQQPMQPMYNQQPFFNQQ

PMFNQQPMYLQPAFNQAPLPYQYPAQPSQFNPFLASFRRFKRQIGRTPYPIHQNVYNDIG

SAKPFNCPADLSGLSGMACCDGGLQFEANKVIDQAKQEPDFDKHNTRNLAKLMTRAVQKR

FGTTFESVVAEADFSWGTNKFNGRTCKIDSQGYNALTYQSSSKPPPPSDFIDIPNDPTLG

GPTGSSGGGGGGNGGSGGGGGGGGGSGGSGGGGSNSNSGGGGGNGGGGNGGGGNGNGG

GAGDGNGGAGAGNGNGAGAGNGNGAGAGNGNGAGAGNGNGAGAGNGNGAGAGDASAAAAA

AQAQAAAAAQAQAAAAAAAQQAAAAAAANAQQAAAAAAAAANPLSALVAATGACFSLDT

WVTTPTGKKRMDQIDIGDYVLTADLEKTYFTPITLWIHREPEKVQEFLTIMTEYGKTLRI

TSRHFMYRNKCGKSYPQYIKMLPHDGEAIFASDLEVGDCVVVLYKGKYRQQKIETITRSV

RTGIYSPLTNNGRIIVNDMLASCYSEIQQNTLQTTFFWAYDKLRSVLVEFFGDLYNNKIE

LPTGTTLSRDIISLIVPIQK

>33088584

MSDKQSVSILIERRRISRRQVIAQRSPSSSAWRGERLRLLSFMPRRIDPLPVSLRYHPKP

LTSPRSCQEASIWHIRAGDDTGSKLSVFVVALASSSSSSTSF

>33640869

MAYPESQVVMGGLVHIPIIIGVFWALNNLTTGGSKAKKAAEAQAKQAAEEAAAKAAAEAA

AKQAAEQAAAKAAAEAAAAKKAAEAAASAAPAATAATPVSGEAETSQASNNDTQATPAPD

QEVL

>35356762

MEHHTWQQKIQASRRIRKRDQAQRSLQSGMVLATATKVKKCESFSLFILRWARQRDKDQE

ENGQVEQRSGGQASFVIKIGIIRKETFFQPFFVTDLFVQGQQLETALGMPTRMKSTCLGG

YCVRSREGDGWHFPLYFHLRIDSLFPKGSSF

>37911972

MKFSLIAVIVVALAIGSESASLVKRDAPAELDKIAKYFQDLVDNLKNVEGAELANKANAY

LEQSRAQFQPMVEKLQEQLKPFSGNIEEQIKPLAASVQSQVAPLAGMVQTHVEDMIKFVA

DQAKAMLPPQ

>38503469

MAAATPAQRAAATATAAAAAAASAAAAAAATTASTAAKVSAGAAATAAAAVVAAKNAATA

VAPNTGAITAATAASATAAAAAKAAQATADAAATKAAAAAVTSKAAAAALAAL

>38603509

MFSSTYLLAIIALAVSSVFAAGPTAVPLGTAGNYAILASAGVSTVPQSVITGAVGLSPAA

ATFLTGFSLTMSSTGTFSTSTQVTGQLTAADYGTPTPSILTTAIGDMGTAYVNAATRSGP

NFLEIYTGALGGKILPPGLYKWTSPVGASADFTIIGTSTDTWIFQIAGTLGLAAGKKIIL

AGGAQAKNIVWVVAGAVSIEAGAKFEGVILAKTAVTLKTGSSLNGRILSQTAVALQKATV

VQK

>39576100

MRNINTAKPKDPERRDALGASKRFAGLLLLVLKKEERPLNFFNFDLKKLVMIGIVLALPL

ISINMQQRPQESNWLAKPFSLLGSAVSESFYGFSSGVKGTTAMYLDLINIKKHNEHLTST

NNELQARLEKMNELLIENDRLRGLLTFKEQTKMSLMAAQVIGRDLVIDHNTITINKGTQD

GLKAGQAVITTGGVLGYIFKPDTFTSHVMLITDRYAVVDGIVQRTRAHGIVEGKSQYTSS

LQLKYVERTEDVQEGDLVVTGGLDNIFPKGFPVAVVESVEKKTFSVSLKVDLRPVVDPYK

VEEVFIVLQGNNEDFGDKFAPASATAETPADGSAPAAAATSAATPAATPAATPAATPAAV

KPAATPAAKPAATPKPQENRQ

>39651373

MSDLSAAPSDAKTALRAAALARRSALSQGDRSAAAAAIAARGLPVPLPDGAIVAGYAPIR

GELDPLPLMHTLAAHGARLALPVVTRQGTPLVFRAWREGDALPRSALGIREPSSDAPEVT

PDIVLVPLAAFDASGHRIGYGAGHYDCTLEYLRARGLVVAIGLAFAMQQIPAVPAAAHDV

VLDYVLTEQGLLTFRSR

>45357246

MITYGEQGCPPLQGANMWYREGTITFTQGSDALSGTGTYWNVTANGVLPGMIVIGPDNKL

YEIKRVISDTSLILAEPYTGETQKEVPCRIITTYEGDLTQFSARFTALMTRMSADSKTMR

SWLTAVDEVTLEREDGTEVTVKSLTQIVDEHNANQKWYTDNADAINAAGEKAREAAERAL

AAAQSSSEARAKADEAAQSSASASEYKTAAELSAAASKASEHGAAESAASSKASASAAKT

SEDNSAASETNAAESKAAAALSASSSANSASEALQYAESAKTSKEAAAASEAAAANSENE

ARTSKDTAVAAAAEASANATSADASRHDVDTNKAEVSRMKDEVFAARDSTIQYSEEAKTA

ADTAAREAATKTSDQLLSAVKSEAEKANSASASAQGFADDAKRFRDEAQEIAEGSKVNDA

TTSQQGVVQLSSATDSESETLASTPKAVKTVMDAVALKAPIDSPALSGAPTAPTPAITAA

GREIATAAFVASKVAQLVGSAPEALDTLNELAAALGNDPNFATTITNMLARKQPLDGTLT

ALSGRSPQGVIDYLGLLNTVNLAAGSIQKSQNGADIPDKRLFVKNIGAVSSARISFVKES

GWYKLATVTMPQGASTALITLIGGAGYNAGLYDQAAISEIVLRSGNWNPVGITATLWQRS

PAGAQGVAWINTSGDVYDIYVNVGQYSIDVIALSDCTNNASIVLFGTPEYVATKPASSTN

GANYILYSSVLPPPESYPVGAPIPWPNDVAPSGFAIMQGQTFDKSVYPKLAAAYPSGVLP

DMRGWMIKGKPTSRAVLSLEQDGIKSHAHNAAASSTDLGTKPTTTFDYGTKTSSGFDYGT

KSSNSTGAHAHSLSGSTSSSGAHAHTVTAHTQYPRSTDSRNQNAVGKQYNTQQTTANAFN

VWTSSAGDHAHSISGTAVSAGAHAHTVGIGAHAHSLSIGSHSHSVAIGAHSHTITIAACG

NAENTVKNIAYNYIVRLA

>45357308

MSKSLLDLLNKTRGDIASKRGNNVDLTRLKDGNNYLRIFPNKDDPNGVFFQTFGMHYVKH

QNEEGKDVTTAYICEQHTHGHACQLCEMVMEGRARFKGNKAMEERINSMRATPRYLVNGV

LSAREDFADAEKCQLIELPSTVFDDICKVMSEDIADDIGNPLSKEEGYAFLIKRTGSGRD

TKYDVSPKRKVYKGDIPEKLWTTQHDLIAYANQADETRLLSTARTMGRLIGIAAPAATMS

SPAISSAAKSAAAELPGFGSITGHTEGAAAVATAHTPAPESTSLVDEEILRAAEAEFKPE

TKPEKVKAPEAAAAASASASAAAASVPADEGLDDLLAELDAL

>45435436

MKWGKLALLNVILWHSYTVFAQSIALVQPVALTRSVALTRSVALVLPVTLASPSITVSPS

VPMPVANTLSGNLSSVGSDTLANLMSLWANDFNHHYPGVNLQIQAAGSSTAPAALAAGAT

QLGPMSRPMKAGEIAAFIQRYGYPPLAVPVAVDALVVFVHQDNPLNRLTLSQLDAIFSQN

RRCGEPQKIQRFGDLGLSGDWAARPLQRYGRNSASGTYGYFKRRVLCDGDFINNINELPG

SASVVQAVAGSLNGIGYSSIGFRNSGVKSLPLAASGQDFVMPTAENVSNGLYPLSRYLYI

YINKAPGQPLEALTAAFLERALSPEGQKLVSHDGYLPLPPAMLNNTRKTLGFTD

>45435722

MNSNFRRHLVGLSLLVGVAVPWAATAQAPISNVGSGSVEDRVTQLERISNAHSQLLTQLQ

QQLSDSQRDVDSLRGQIQESQYQLNQVVERQKQIYQQMESLSGGQGAQNSASAASGATAD

NTAAGSSGNADAGAAASTAAPAASTGDENSDYNVAVSLALEKKQYDQAITAFQSFVKQYP

KSTYQPNANYWLGQLYYNKGKKDDAAYYYAVVVKNYPKSPKSSEAMFKVGVIMQDKGQSD

KAKAVYQQVIKQYPNTDAAKQAQKRLSAL

>45435725

MWKSSVGKATEQNDKLNRAVIVSVVLHIILIALLIWGSLTQTTEMGGGGAGGEVIDAVMV

DPGAVTEQYNRQQQQQTDAKRAEQQRQKKAEQQAEELQQKQAAEQQRLKELEKERLQAQE

DAKLAAEEQKKQVAEQQKQIAEQQKQAAEQQKIAAAAVAKAKEEQKQAETAAAQAKAEAD

KIVKAQAEAQKKAEAEAQKKAEAEAKKEAAVAAAAKKQADADAKKAVEVAEKAAADAAEK

KAAADAEKKAAAAKKVAAAAEAKKKAAAEAAASTDVDDLFGGLANAKNAPKSGSGAGAAA

AGKGGGKKSGASGADISGYLGQITGAIQSKFYDADLYKGRTCDLRIKLAPDGLLIDVKAE

GGDPALCQAAIAAAKQAKIPKPPSTDVYEQFKNAPLVFKPQ

>45436240

MNASITGGENRENTFTMTLARLRANPKIPLLIAAAAAIAIIVALMLWAKSPDYRVLYSNL

SDRDGGDIVTQLTQLNIPYRFADNGGALLIPAEKVHETRLRLAQQGLPKGGAVGFELLDQ

EKFGISQFSEQINYQRALEGELSRTIGTLGPVLNVRVHLAMPKPSLFVREQKSPTASVTL

ALQPGRALDDGQINAIVYMVSSSVAGLPPGNVTVVDQTGRLLTQSDSAGRDLNASQLKFT

SEVENRYQRRIENILAPMVGNGNVHAQVTAQVDFASREQTDEEYKPNQAANQGAVRSQQV

STSEQLGGTNVGGVPGALSNQPPVAPIAPIEIPQPAGAAANNAAPANTAATANANTTATA

AKASSSNSRHDQTTNFEVDRTIRHTQQQAGMVQRLSVAVVVNYTSDKAGKPIALSKDQLA

QVESLTREAMGFSTVRGDTLNVVNTPFTASDDTRGSSLPFWQQQSFFDQLLNAGRYLLIL

LVAWILWRKLLRPMLAKKQVADKAAASVNNIVQTAQAAETVKQSKEELALRKKNQQRVSA

EVQAQRIRELADKDPRVVALVIRQWMSNDQ

>45436761

MKTVLTLIVAATLSLFSVAFAADTVVPQTTPTATAPAPAPAPAVHNASAKTMHHKKAQKP

NTIKNTHKTAPEQKAQAAQKHHKAAHSHKAPTVAPAAK

>45437103

MKMKKVLALMVAATLGLSSVAFAADTTATATPAATSTTATVAAQTKATQHQKHKVTKKTT

EQKAQAAKKHEKKASVQKTPVQKAQAAKKHVKKASVQKAPVQKAQAAKKHHKTAKKPVAA

PAA

>45437651

MLDKIKQKLSPGQLGENTKTILMSAVALLVTAAIIFSLWRSSQGYTALFGSQENIPITQV

VEVLEGEAIAYRINPDNGQVLVAENQLGKARILLAAKGITATLPIGYELMDKESMLGSSQ

FIQNVRYKRSLEGELAQSMMALSAVEYARVHLGMSEASSFAISNHADNSASVVLRLRYGQ

TLSTEQVGAIVQLVAGSIPGMKPANVRVVDQHGELLSQAYQANSEGVPSVKSGTELAHYL

QSTTEKNIANLLNSVIGANNYRISVSTQLDMSRIEETAEHYGPDPRINDENIQQENSNDD

MAMGIPGSLSNQPIPQSQAGQTPAAVSRSQAQRKYIYDRNIRHVRYPGYKLEKMTVAVVL

NKSLPVLEQWTPEQQEELKRLIEDAAGIDVKRGDSLTINMMAFAVPTLIDEPVMPWWQEP

STFRWAELLGIGLLSLLVLWFGVRPLMKRYSRKGSENLPLAISSASADEALDHVDTGVDG

AESSPRTENAFSASSLWKSDDLPEQGSGLETKIAHLQQLAQSETERTAEVIKQWINSNER

IKSQPE

>45438617

MKLMRTTVASIVAATLSMTAVSAFAAASLTGAGATFPAPVYAKWADSYQKETGNKINYQG

IGSSGGVKQIIANTVDFGASDAPLTDEKLATEGLFQFPTVIGGVVLAVNIPGIKSGELTL

DGKTLGDIYLGTVKKWNDPAIVKLNPGVKLPDQNIAVVRRADGSGTSFVFTSYLAKVNAE

WKEKVGAGSTVNWPTGLGGKGNDGIAAFVQRLPGSIGYVEYAYAKQNNLAYTKLISADGK

PVSPTEHSFSSAAKGVDWSKSFAQDLTNQKGDDVWPITSTTFILVHKEQKNAANGTEVLK

FFDWGYTHGAKQANELDYATLPAEVVEQVRAAWKTQIKDSSGKPIF

>51095271

MKYTLIAAIVVLALAQGTLAVEQSPELEKMAQFFEGMKTELMATVQKVSESLQSQTIIED

GRTQLEPIMTQIQEHLAPLATSVQEKVTPLAEDMQQKLKPYVDEFQSELESVLRKLLDQA

KAITQ

>51490682

MQYDSPITNTEFQTFLTTSSISDDTAAAISTLLNLDSADTINLASWDGVNAPEIPTGQEG

AADVVIVNVPGAATDLVPVEIPDSLNSAKAFIFDSNASLAVTFDAPVAAESASLARVAAD

TTAGIEFLVTTGAGNDVITVNGDQNSYIDAGNGNDTIVTGNGNNTVVAGAGNNNVTTGTG

NDTIILSGTGHTDIVNTGAGYDVVQLDGSAADYTITAGNSNNVTLTGAQTAAITGAEFLT

FADGSSVALAQSEAEASALRLYEGILGRDADQGGAQNFIAQVEAGTALTDIANSFLNSAE

FGGAATEASIDSLYTSLLGRGADTAGSDSWEAIIANGGSLADVAAGIAGSAEAQEQDQSN

GTFVDSLYLNALGRPSDEAGHDAWVAQLFNGASRAEVAAGIVGSAEAAEKINSDFIDALY

LSATGRASDEAGKAGWTEVLANGGTQADVAIGIVGSQEAIAHNDNVVVLHGAV

>57635192

QISRNSRCNRSTLTNCYVDNSQVDTTTCTSSQYNSANVMTSTTTNCNIRNSYVYTTPCTS

SQYDGIYITSSTTTGSRISGP

>57635194

QITNSRCFKSTATNCYIDNSQLDTTTCTNSQYNNAYVVISTTTNSIIDYSQVYSTTCTNS

HYNGVHITSSTTTNTRITGP

>57635206

QISENSRCVNSTANNCYIDNSQVDTTTCTRSRYINANVMISTTTDCSISNSQVLISTCTN

SQFSGIYMTTSTIRDSRISGP

>57635212

HITNSECVTSTLTNCNLVNSQVDTTTCTNSEYKDAHISTATTTGTRIA

>58220717

MALTTKWFLIAVVVMCLCSEYYCQCTGGSDCTSCTVACTNCENCPNAVTCTDSTNCINAQ

TCTGSTNCNNAVTCTGSYNCNKAVTCTNSFDCFEAVTCTDSTNCYKATTCTRSTGCPNKR

>59897266

MAITKIILQQMVTMDQNSITASKYPKYTVVLSNSISSITAAELTSAIESSKASAAAAKQS

EINAKQSELNAKDSENEAEISATSSQQSATQSASSATASANSAKAAKTSETNANNSKNAA

KTSETNAASSASSASSFATAAENSARAAKTSETNAGNSAQAADASKTAAANSATAAKTSE

TNAKKSETAAKTSETNAKTSENKAKEYLDMASELVSPVTQYDWPVGTNNNSVYVKIAKLT

DPGAVSCHLTLMITNGGNYGSSYGNIDFVEISARGLNDARGVTSENITKFLSVRRLGSPN

LAWDNQLRYGLVEGDGYFEVWCYQRAFIKETRVAVLAQTGRTELYIPEGFVSQDTQPSGF

IESLAARIYDQVNKPTKADLGLENAMLVGAFGLGGNGLSYSSVQSNVDLINKLKANGGQY

WRAARESGANVDINDHGSGFYSHCGDTHAAINVQYNTGIVKVLATTDRNLASDIVYANTL

YGTANKPSKSDVGLGNVTNDAQVKKAGDVMSGDLDIRKETPSIRLKSTQGNAHLWFMNND

GGERGVIWSPPNNGSLGEIHIRAKTSDGTSTGDFIVRHDGRIEAKDAKISYKISSRTAEF

SNDDTNTAATNLRVSGKQHTPIMLVRDSDSNVSVGFKLNNMNAKLLGIDIDGDLAFGENP

DHKQNSKIVTRKMMDAGFSVAGLMDFTNGFAGPWEAKNISDQELDLNSLMIKKSDPGSIR

VYQCVSAGGGNNITNKPSGIGGNFILYVESIRKVGDTDFTNRQRLFGTDLNREFTRYCSN

GTWSAWRESVVSGMNQDVSVKSMSVSGRLSGNELSVGGAGVLNGNLGVGGGATSKMPSSD

KGIVIGRGSIVREGGEGRLILSSSGGTDRLLQLRPAGATSLDNQVEISCTSASAGDTKIS

FGQGAAIRCNNAGSPIISAKAGQMIYFRPNGDGISEGQMILSPNGDLVVKGGVNSKEIDV

TASQSLPLKETTATTGIGVNFIGDSATECSFGIENTAGGSAVFHNYTRGASNSVTKNNQL

LGGYGSRPWLGSTYTEHSNAALHFLGAGDTSATNHGGWIRLLVTPKGKTISDRVPAFRLS

DNGDLWLVPDGAMHSDLGLVRSIETLNAAVPRFNAPSIQDGRGLKIVAPQAPEIDLIAPR

GSGASAPAIRAMWCDGSLADTTRYIGATQPGSTFYIGASGHDGEKFDSMRGSVAIKSAGG

WGPTSTPTQVVLETCESGSISRLPRWGVDHNGTLMPMADNRYNLGWGSGRVKQVYAVNGT

INTSDARLKNDVRAMSDPETEAAKAIAKEIGFWTWKEQADMNDIREHCGLTVQRAIEIME

SFGLDPFKYGFICYDKWDEHTVVSEYGPANEDGTENPIYKTIPAGDHYSFRLEELNLFIA

KGFEARLSAIEDKLGM

>62127205

MPVLISGVLKDGTGTPVQNCTIQLKACRTSTTVVVNTVASENPDDAGRYSMDVEQGQYTV

TLLVDGYPPSHAGVITVYDDSKPGTLNDFLGAMTEDDVRPEALRSFEAMVEEVARQASEA

SRNATAAGQASEQAQTSAGQASESATAAVNAAGAAEASATQAASSAASAESSAGTATTKA

GEASASAASADTARTAAAASAAAAKTSEANADASRTAAGDSAAAAAASATAAQTSAERAG

ASETAAKTSETQAASSAGDAGASATAAAASEKAAAASAAAAKTSETNAATSASTAAASAT

AASSSASEASTHAAASDTSASLAAQSSTAAGAAATRAEDAAKRAEDIADVISLEDASLTK

KGIVKLSSATDSDSEALAATPKAVHAVMDEVQTKAPLDSPALTGTPTAPTPETAAAGIEI

ATAAFVAAKVAQLVGSAPETLDTLKELADALGNDPNFATTVLNKLAGKQPLDDTLTALSG

KSVDGLIEYVGLRETINHAADALLKSQNGGDIPEKPLFVQNIGALPASGTAVAANRLASR

GALPALTGATRGSDSGLIMGEVYNNGYPTQYGNILRLTGTGDGEILIGWSGTNGAPAPAY

IRSHRDTADAEWSEWAMLYTSLNPPPNSYPVGAAIAWPSDATPAGYALMQGQSFDKSAYP

LLAIAYPSGIIPDMRGWTIKGKPISGRAVLSQEMDGNKSHSHSARAQDTDLGTKSTSSFD

YGTKSTNTTGNHTHQFGGYINSYWGDSNHTSFQPGGGAWTQAAGDHAHTVYIGGHEHTMY

IGPHGHVVIVDADGNAETTVKNIAFNYIVRLA

>62127439

MPVLISGVLKDGTGTPVQNCTIQLKACRTSTTVVVNTVASENPDDAGRYSMDVEQGQYTV

TLLVDGYPPSHAGVITVYDDSKPGTLNDFLGAMTEDDVRPEALRSFEAMVEEVARQASEA

SRNATAAGQASEQAQTSAGQASESATAAVNAAGAAEASATQAASSAASAESSAGTATTKA

GEASASAASADTARTAAAASAAAAKTSEANADASRTAAGDSAAAAAASATAAQTSAERAG

ASETAAKTSETQAASSAGDAGASATAAAASEKAAAASAAAAKTSETNAATSASTAAASAT

AASSSASEASTHAAASDTSASLAAQSSTAAGAAATRAEDAAKRAEDIADVISLEDASLTK

KGIVKLSSATDSDSEALAATPKAVKTVMGEVQTKAPLDSPAFTGTPTTPTPPDDAKGLQT

ANAEFVRKLIAALVGSVPESLDTLQELADALGNDPNFATTVLNKLAGKQPLDDTLTALSG

KSVDGLIEYVGLRETISRATGAMQKDQNGGDIPDKKQFARTIGAVTSTTITFGESGWFKI

ATVFMPQTTSTAVIKLYGGSGYNVGSFEQGAISELVLRSGNGNPTGITATLWRRSPVAAN

EVAWVNTSGDTYDIYINIGQYAYWLIAQYDYTGNANVTLYSTPEYSSVQPGNSTSGQTYT

IYSSLMKPTAGDVGALPITGGRLNGSLGIGTDNALGGNSIVFGDNDTGIKQNGDGILDTF

ANSQHTVRVAPGEMQVLGSIRAGNAKRMTMTSSNNSVLNAQFNLWGDGNRPTVIELDDDQ

GWHLYSQRNTDGSIQFVVNGQVIPDNYGNFDARYLTSGNVYTKGESDNRYVQNIQRGAPV

WPGKVDEYGPAEAPAGCFLTQARHDPTTAYGVTFAYRPLQMWVGNGWRTING

>76583840

MIQVTRKQIVAAAAVVVVALGAYYGWTLLRHQGPGDGFASGNGRIEATEIDVATKLPGRI

DAILVDEGDFVKAGQPLANMQIQVLRAQYDEATAQRQRALNTAAGVQSQVAQRKSDKAAA

QAMVVLRESELDAAERRLARSQTLSREGASSLQELDDDRARARSAQAAVSAAAAQVAAAQ

AAIEATEAQLVAAHSAVTAADATVARVQADIDDSQLASPRDGRVQYRVAQPGEVLPAGGK

VLNLVDLSDVYMTFFLPETVVGRVALGADARIILDAAPNYVIPATVSFVSSTAQFTPKTV

ETANERQKLMFRVKARIDRELLQKHLKLVKTGLPGVAWVRVDPSKPWPAQLMVKVPQ

>76875408

MSKVVDVVVDTGGDIFGLGRSVFNKTVGALWDSLTPDLPEEDLATLAKGLQKGIDQPRRI

TFGRDRVGGVIAHQAEVERGEKKFVQMVVLINGTPIDALEEIYIADKPITDYPTESWDYE

LSDGRHTAANAKAVAKMAGWTNEHIGIDQAHIFIELENNREVFEDGISETEFLIRGARVW

DPRDTTQNPDDETTWAWSQNAVLCALHYVRFYGAHEVPFDRLPLGWWIAAINVCDEDAEF

KDAAGKITTEKRYTTNGSFTFSSKPLDVLNQLEGCFAGKIFRQMGQWYVRVGAWYGNPTY

TINADDVHGNIKIKWHADLRDRANVVRATFTDPEQNYDRTDAPPVVSAGYQAIDNQVLEK

SITLPFVRSGTTAQRLATIHLEQTRLGEIELPLKHKGLAAAVGRTVYLNLPNESINNKIY

RVVERRFRLDGGVTLMCVEDGPNLWADNLVPGAQDLTPNSDYLVGKPQPIFDVRVTIDGD

GNGIIKWNHPAPLAVNEYDVEFINTAADEQVFKTSVTYTQVTIPNLQLGEYTARISAKNI

FGQRSLLVAVQFSVLTPTLPTVYVTADYNQITLTADIATAGIGTAFEWEFLGTTESPQSG

ERVLAQIYNRIGLKSETEYKFRVRSVNHLGASDWVDVIANTTLVDLTEFINGVELTQLSE

DAQTLIEDMNTQVDRLRPETENNLPSLIAKNIDALTGLAEKVQVLDAENPNSIPFQIDQL

VNIVDVINAENPNNLQQQLADSNSKINDLERVTEVLDETKQNSLPALIKINNIAIEQQRL

AQQSIGLSLLNVTSAYTNWRNEYERRAFNNERLIDAAVYVDPDTGTIINRAFAYADESFN

SATLMIEGANSKITIASQQIAQSQSRISQAEAQLIIQAAQINQKATFSEVESQIAGALAA

LQPAYSWQFNTSSEGFDPDSHNATGYIVATAQISSPAISYNAAENPMFRLRVRKHSDATW

RGDIKFNGGTTTLHLPEPAGTDFETLTLDATGTAGYMGTITSLEFDLGACDIDFIEVGKR

GANDLTLADITARTTELEQDINAATGVMAQYATTAWVNALGYQTQSNVQTLIDSFNAQYS

IAATLQEFNDQDIIIKANAAQTWINGAEATIRDQVTSILNSDDGVNQRISVAEQSIDAIA

GEISLSITQVSGLKLDVKKLGLNEVIAAYNKMQQDQELAEQSFSLAAANQKLTAVTNDVE

SLATQTLELAGLYGQNAAYLTSLNQAFANERTARSTTERELRAEITSEGTRTIAQANERL

EAVVGYCVDAQGNRVDEPDAMACIALGHDWVDGPLVQLINDYTSVFVNEKGYQTVANVQQ

FISTFDGQYSITATIQTINDEGIITAAKEAQQWINAAEGTIENIVTQFVNKPNGINDNIA

FAYDLIQANADDITVTANAQQQLSVRMGEAEADLNRIDDLVITEQQARATLGTQLRIEFQ

TQDLAMLATANEFTRAVTGYCVDANGERVDQDDAVQCELDGHTWIDGPAVQRAINISVAY

VDEKGYQTQSNVSQLLDTFNATYQVSATLQEFKDNGTLQKANNAQLFINAAEGYIENQIT

LFNDKEDGVNATFANVKQRLDAAEGAVTTSIKQMQGLELEQQAQGLNDVIAAYNKMMQDQ

DLATLNVKASLANEKLQAQTTGLESLAKQQLELAAIFNSSNAIITSLNKAVANQYEASVV

RDQRYQATFDNVTARFSDVTTAIASIDEANTLRDIEFESFVADTIASFDDVSETFASQNQ

AFSTLQQTLTAKINDDTEAAKNTAIATAQEYTRTAVGYCLNAQGQITSENDAVQCVADGG

SWVNGPLAEFIANMQISDGENTASIKQLRQLFTTVEGKLVARGGWTLDNNGRVVGIAGYN

DGEVGNLDLIANVIRQGVMVNGTFVPTIYLDNSDRLNPVQTFRGRLVLSDGHQVSTLNDI

KAQDGKDGADGADGADGQDGATGPQGIQGAKGADGLTTYTWLKYADNASGAGLSNSPTNK

EYIGFAYNKTTATESTNPADYTWSKIKGEDGANGTDGVPGAKGADGKTTYTWIAYSENVS

GSGMYQTPNSNTKYIGIAVNKTTATESGNPADYTWSLFKGADGADGQNGLNGQNGNRGSI

EVQIATSTGAWSDATANASVPGSPVEHDRVTIYKSSDAKVQTTKRFNGSSWASYTLRVHG

DALIDGTVDGKVFRASSRIESPIIMGGEIVITEPSSDYLDIVGRSLPFGPTADLISWYGP

KINGVTWNSSTQSAIYAGMSKSNAVSYKTNNGGFYFGGTFQAGTLSISRQATNTSGGLNV

LTGSFTIPGGTTSINVTASVTGRGGASGGGTCPTSPYKPSSTVDIEMLVGGTWQAIAGNN

AIGTASCTQEGPEIILSVTTGVSANTNLTVSQGQLLQLRAVARTVSFPYNEAGLKMSGSR

SLSIVVTGSN

>77388297

MPLALPFTSLMALYWQTGLMVAEAQTVMTLRTLGMMGVLTPHPLENQRMVTEKSVAFAQA

AQAATAAAMQGKRPDEVAAAALQPIRRRTRANAARLTRAAKRPKA

>77389918

MEGVRLDLCLLADIRAGDVAAPCHAAAAEAVAAAGYRVGVLPVAPGSIAADPFRLDEGFS

RLFASGRACRLAPGDAVDCTLVLAFDARLFAAGPLPGLRIGAARRLVTVERAAALASLPY

ADLERLAEAARLALGGPPVWAPTTAVAREALTFAVPDWPLTPEDWLPVAPPLAEAGPRAT

HRGRPAIGAARIARVRPGAALPRGAVPLPLWRLRLSPDGERPSWPPAAPVEIWPDDRISL

ADFLPLVDLLANADEAADDPCPVEALLALRAGTVPYLPDGCRSTFGAAALYGAPGDVARR

ALDLQAEEGLARNLRAAGAEALAGRFSPEAFLARLAPLLGPPRPAPFAPAVLARPPARVL

FLSTNGVGMGHLTRQLAVARRLPGRLEPVFLSHSQAVDVARSFGFPAEHLPYHAASGEAR

AHWNAGLAETLGAAIAFWQPAGLVFDGNVPFAGLMAALEEAPDLARIWIRRGLWGAGRDP

EALERGAAFDLILEPGEPAAALDEGPTAVRRGETRGVAPVRLLDGAEIPGRAEACAALGL

DRAAVNVLIAPGSGNNFATGGLAARAVAALAGRRGIGIAVARWMISHEAPELPAGVVALT

GFPFARHLAAFDFALAAAGYNSFVEHLERALPTLWTPNEHAEQDRQIVRARWAERQGLGL

TLRLGEEMRLGTALDRILDPAERAALRAAGASTAEALAARNGAVEAAEAIAALCGTAIAR

GR

>77390013

MTIDLRKTAIAALAALSLVPAAAAPAHALGKKDKAFIAGAATAAVAGVVMQNMRANGQWP

FGQQQPRYAAPPAQPQYYVQPRATYATTAPRQVTYQPRYVAPSVHSTPAAQAFRELSPQM

RREVQISLARYGYYSGSIDGAWGPGTSAAVDAYARDSGRIGQLGSVAGAYGVMDSLGG

>77390077

MTRTLASLFGPLQPMALAEDLAAPLLAMPIPEGAADPAAAARAPAAGPTVPDRFTVARGL

AVVPVRGILTPNMAQYERWFGWATYHGLAETMAHLAASEDAAAIVLEIDSPGGLVCGIEA

AAEAIAAAAAVKPVHALVSPLAASAAYWLASQASEIVMTPGAVAGSIGVALTAAAHVQPG

ANGAQIFEMSSRHARAKRPDASTEAGRAELQRSLDEAEAAFHAAVSAGRAIPAAELAARL

SVTDDPQDGGATFRAPEAIRRGLADRIETRAAFYARLAARTAPKPRSPSRAFAARAAAAA

ALARS

>77390079

MARQNLDDLRRARKAAADTMAAVAARIGALEAAEAPDAAALETETAAFAAAEAAFAKADA

AVTRAAAVEAAQAAAAQGDGAGAGGGAGAAGADAVPAVASDPAHRGVAAGFMVQALARTK

GDRDKAARLLEAEGHGAISAALSGASEGAGGVTIPRPQAAELIEMLRARVVVRASGARTL

PMPAGEMRHAKQVGSAVAAYAAENAAIAPSQPSFDKIDQSFKKLLGMVPIGNSLLRHSGV

AMAQLVRDDLLKVMALREDLAFLRGDGSADTPKGLRHWMLPANWSAAPVAATPAAAEAAI

RRTVSLVEDADVGMVSPGWIMRASTKNWLASLKDPNGNPLFPSIGASAQLMGFPIRTSSQ

RPARRAHTTGAARCRKRDSSTPSPRSGAGPSPR

>77808090

MAQMKFILVAFLVVLAVSWANACKGADGAHGVNGCPGTAGAAGSVGGPGCDGGHGGNGGN

GNPGCAGGVGGAGGASGGTGVGGRGGKGGSGTPKGADGAPGAP

>82409799

MPIYEYRCGSCGFEKEYLQKVSDAPVAACPACGSDAFNKLISAAGFQLKGSGWYVTDFKN

KNGGQSKPKADADACVAKSESAPAGDSACPACTPIDG

>82494436

MAFKTCALTKNWLVIAVIVMCLCAEYNCQCTGGADCTSCTAACTGCGNCPNAATCTDSQN

CVTAATCTRSTKCNTARTCTNSKDCFEAATCTDSTNCYKATTCTIQQDVPDIRFFYCQQ

>82615624

MSIRTITAMAGTAFIMGAAAIAFSSPASALTMKECSTKYQAAKDAGTLGNMKWNDFRKAQ

CGDDAASAPAAAPAAAPATKKAAKAAAPASNDGAKSLTMKQCSAKYQAAKDAGTDNGMKW

NDFRKAECGPGADPVALSTDGDSEPAAPSVAAPKGVKFPTAVSAKYSKESAGKARMHTCL

DQYHALKDANALGGLKWVQKGGGYYSLCNARLKGNS

>82615849

MTDSSANPRNYGERPVHEDDPLMELSRIMDFDTPADDNVARNERRHDSQFEDQGRAEPRF

DSAQDDPSFDPVLDLERELMGHFDDYTQPTAHSETVSTATFGVDGERAYGEQSPLEEDAF

AAALEEEFDLDLGSAEAAPSPEIFEFEDVDRSEPVPQFDYNDYSQARDSSEHAHMASPAA

HDDRQQPIEADAGEYDPTVYQPAIEPGEQAWREDTGVQDNWQAQDEWPAQNDRLEQNDWS

EQNDWPAQNNWNAQQPVDAPATHFAAEPAQQPLSLEDELENLLFGDEPQPVASRNSYSDH

VEPVSYAPEQPHQGEPEAPVSEPHLDAPSHAGQGYASRTGEAPAYPYYPRSNFAPGVAAP

GVLSSGPQLKTPQAPIVQDFVAQDPFRPIEDEFSLEDDFTFEAEPETAAAAESTDDDLSG

LDEISLTEDDFGFEPSGDTFDADVADTADEDFFNDEDFFTDVELDLHEEEQEASPAPAYA

HAIHSGEDYRPAASFAAYTDMQAPARMPAPEPAPEVETLTVAENKVEQTHSLDLPEVNYG

EEEAGTNLSELEAEFAEVFSTIGVDENVQITEGQSEADRAFEDIFRESASTYMPNSGMAA

AGLGAAAAAAAASYRRAGSEAAPAAATTASQDDFYNHWAAQGAQTMEGGDYGERAAMPTE

DDLGGAAEAYRNRPVRGRRGLILASVAGVAVLLGGIGYHFLGGGGSGEPVVIRADNQPIK

MQPENPGGTTVPNQDKAVYDRVAGTLPNNPEQKALITSGEEPVDISGTDDSEYNATEEPG

GNVPQNNAQQAHSGTHEPLIQPREVETMIVRPDGTIIQPSFGHAAQPSVADNMAPPAAPA

ARDEIGALAAGNEPPAPQAQQSATPETPRLPTRAPIVPSRPAEQPVNIVGNVPQRAQASA

APQVASAAGAGGYFIQIASQPSAELAQKSYANMAQKYASVIGGHSVDIKRADIQGKGTYY

RVRVQAGSKEDALALCSRLKSAGGSCFVTQ

>82616228

MADPKFYLGMGCSSAVTYDELLPLAEEALTKGGCSRPDGIATLSTKRGNPAWVQLAVHYG

CELRFFDASRLEDETPRLNSPSQETFKAVGCHGVAEAAALAAAGPNGHLVVGKIASARST

AALAMVPTFPL

>82616363

MRFACLNTGFLALWLFVMNRNFLFFVSAIVLFALAACGKGDEEKAAAPADGQAASQPATP

VEPSAGEKGPDLLKSMRENSGVMTPEEKAAAIERARANAETAAKAVGQSVEQVQAAGEAA

AVAAQRLLEERQPQ

>82616390

MSVISLKSFVVTAAIGVAAMFSASASANAASAVMAPAITTAAHSSVMNVDYRRHHHRPIV

RGCSVEGAKMKAHRMGIRNARVTYRGRTVTVRGFRHGRPTSVTFADTRGCPIIR

>82616680

MAKAATPKTTAAAEAKPAAKAPAKKAAPKTTAAAKPAATKSGAPKAAAAGAIGHITQVIG

AVVDVKFPEGQLPLILNALEVDNQGHRLVLEVAQHLGEDTVRTIAMDATEGLVRGQEARD

TGEPIMVPVGVETLGRIMNVIGEPVDEAGPIKTKATRAIHQNAPEYIEQSTEAEILVTGI

KVVDLLAPYAKGGKIGLFGGAGVGKTVLIMELINNVAKAHGGYSVFAGVGERTREGNDLY

HEMIESGVNKLGGGEGSKAALVYGQMNEPPGARARVALSGLTVAENFRDQGQDVLFFVDN

IFRFTQAGSEVSALLGRIPSAVGYQPTLATDMGAMQERITTTTKGSITSVQAIYVPADDL

TDPAPATSFAHLDATTVLSRSIAEKGIYPAVDPLDSTSRMIDPKVVGEEHYAVARQVQSI

LQRYKALQDIIAILGMDELSEEDKLTVARARKIERFLSQPFFVAEVFTGSPGKLVDLADT

IKGFKGLCAGDYDHLPEAAFYMVGSIEEALEKAKKLAAEAA

>82616703

MALKIRLARAGSKKRPYYHVVVADVRAPRDGRFIETVGSWNPVLPKDAERVKLDAERIQH

WIAQGAQPTDRVLRFLDQAGIAKRPSRNNPTKGEPGKKAQERLALAKQAEEEASAKAAEA

AAAAAAPAEEAASE

>82616831

MAILLIAEHDNATLSDQTAKALTAAAQIGGDVDVLVAGKGAKAAADAAAKLKGVRKVLLA

ESDALENRLAEPTAELIVALAGNYDTIIAPATTSAKNILPRVAALLDVMQLSEIMEVVSA

DTFKRPIYAGNAIQTVQSTDAKKVITVRTASFQATGEGGSASVESVNAPADPALSSFVEN

ALSGGDRPELTSAKIIISGGRALGSAEKFQEVILPVADKLGAAVGASRAAVDAGYAPNDW

QVGQTGKVVAPDLYIAVGISGAIQHLAGMKDSRIIVAINKDEEAPIFQVADYGLVGDLFT

VLPELEKAL

>82616895

MPVLNRAIEMQAEIAAWRRKLHQNPELLYDVHETAKFVEEKLKSFGCDQVETGIGRTGVV

GIIKGRHGDGHAIGLRADMDALPITETSGAEWASQNPGKAHSCGHDGHTAMLLGAAQYLA

EARNFRGSVALLFQPAEEGSAGGLAMVEDGVMDRFGISEVYGVHNMPGLPVGQFAMRKGP

IMAATDEFDLFITGRGGHAAQPHRTIDPILAGSQLMIALQGIVSRNTDPLDSLVISVTKF

MAGEAYNVIPEKAKLSGTVRTLKKETRAFAERRIRETAAGIAAATGAEITVRYKNNYPVT

FNHDAQTEFAARVAATVAGEGKVDTNIEPMMAAEDFSYMLEARPGAYIFLGNGDTPGLHH

PAYDFNDDAIPYGVSYFVAVAETALAA

>82939275

MKKAALALAALATAATGIATPTLAADNGLRVQIQYYDDDGYRVPRQGFDGPGRHDRRPNW

GDGYIRREVMNPRRVARSLERRGYDVGDMRLERDTYFVRATRPSGRRVVVMVDAYNGNII

GERRAGPRPSGY

>82939460

MADTNLDKALEGLNQAADAVRQAAENAGGFGDAAAAAAHAASGGAIDPFIFRFAIFILSI

FVGYYVVWSVTPALHTPLMAVTNAISSVIVVGALLAVGLSLSGWATGFGFIALILASVNI

FGGFLVTQRMLAMYKKKEK

>83842416

MNPLTLMTLNANLTKLMIDTQAVMTLRLLGMAGALPQTRGENARMVNEKGPAMAKAYQAA

TKAAFAGGTPDQIFSAAMVPVSKKVRANRKRLTK

>89573790

MQAAAMSVSFTRPALRAARPAVSARRTMTQRSMVVRALGEESLGDKAKNAFEDAKDAVKD

AAGNVKEAVVGAADDAEGAAKDAGRKVDRNTDNLADKASNALGDAKDAAKDAYNTVKDKV

SNAADDVEGAAKDTGRKIDRNTDNLADKASNKFDEVKGDVKDAARDAKRSVKNEANKL

>89573792

MAGNKPITEQISDAVGAAGQKVGETFEAAKAQAASLTGTAEQKATEAKHDANRQGGGVVD

DIKGAAAEAQHRAGETAEKAKHNVQEGWTETKHKVDEARPNATR

>94549115

MPLYEYECKQCRERFEKIQKFSDEPEKVCPKCGGEVERLLSAPAVQFKGEGWYVTDYAKK

KGGAAKSSSTDSTSSEKKSESAPASAETPKPSTSSDKKS

>108871254

MFGAISNFLKIVLIVLFYQLCCTHAQSKYQIAPYDSNWFEASEYCHRMDMRLAIVDSEEK

HNAVVKEAKAAKLHSSGFFGVWLGATDLARSGNFIWHNTGARLRYARWGEGEPSGGREHC

VVLYYWPKQRFNWTWNDAPCSTELYAICENYEKAACIQEF

>108884197

MALSLYLIAVICSLVGFTASQQTCDNDNRFCFPNVVANWIGAAEYCSRNGWRLAVLDSEQ

KQQQVEELAQRVDAFKTAKVELWIGASDLAREGKFMWHPTGLDVSYSKWIAGMPDNKDGY

EHCVHLWYEPSRLINWHWNDVVCASMRRFVCEQA

>114341455

MTSPADKPALRSRMIELRRMAAVRLPAAGDELARAFPEAWLPAAGEPVAGYWPLDSELDP

RPLMKRLNDLGHSLCLPVVQGHGLPLVFRGWADGDALECRAMGVMEPRDDRPAFRPGLVL

VPLLACDRSGNRLGFGKGYYDYTLAALRAAGQVRAVGLAFDCQVLATIPAERHDQALDGV

VTQSGFMSFSARLSGTLDA

>114543540

MFSQMLEWQRSALKVASLAAEAQQVVAYRTLGMAGLWSVDPSENVRMVTEKSPALMQSWW

SASRAAMGGAAPERVVMAWAGPLERKARSNRKRLAKRGPNMNFGA

>115362378

MPIYEYACQSCGKIIDVLQKISDPPPPACTECHAENTLTKVVSRSSFVLKGGGWYSDLYS

STKKDGSSSSSSSSGSSASSSSGGSTSSSSTASSSASTSSSPAPAPSAPASSTKS

>115367318

MRSLKTGVLFLLFSLMTACGQQLVEFPDDTNDPGDDAGSGGDAGSGGDAGSGGDAGISPT

VVSTRPANAATSVAVNAPITATFSTEMNPATLSTAFTLRQDATFVAGDVAYLGTTATLTP

KGNLAPGSVFTATISTASKDLEGHALATDYTWSFTTDAHIISPTVIASSPVNAATNVSTN

KRIMATFNKGMNPATITTSTFTVYQGATAVAGAVTWSAATNEATFTPTLPLELSQTYTAT

ISTGAQDAAGSSLATNHDWSFTTGACSQLPVELGSAGNFAVMAGSTVTSTGQTSVTGDLG

VSSGTAITGFPPGKLIGAKHAGDPTAAQGIADLTAAYNNAAGRSLCPVTVAGNLGGQTLT

PGLYKSTSSLAISEGDLTLDAKGDGDAVFIFQMASTLTTTAGRQVVLTGGARSTNIFWQV

GTSATFGTTSSFQGTVMADQAITLNTGATLNGRALARIGAVALDDNTIVKPAP

>115370281

MLTQLTVDADEVALFVKDGKVEGKLGPGRHQLDTKNIPFLSRLLEKFTGGDLFISEIFFV

STREHTGVKFGGPIGDVRDPETGLGIGTMVYGDFSIRVTEPERLVVGLVGMGRSSNEDFV

GWFKSQVLKVTRDRIAELLVKKRWPLLDVTSGAYTEEIETEVIGGLKPHVDSYGLTVVRM

GNFHVSIKEEDEATLKKFSKDAAYSRLAGGFNQYAQGQAMLGASEGMAKGGGHGSGSDGA

LQGMGLGMGMGMAQMFANNQQQARQAPQGPVPEAAGGGEARSPAQRLKELHELHKAGVLS

DDEYSAKRAELMKLL

>115370365

MGAPRFVFYAVNGLGLGHVTRLVSIARALRRLSPGCEVLFLTSSEADHVIYREGFAAVKL

PSKTIREHCGLRKGSYLKLAQTVTWNTISAFDPDVLVVDTYPTGSFEELLPVLRWRQKNV

FVFREQRAEAAGSQLLQATLRLYDRILIPHESVSQVGPVPEPTKALAVGPILIRERHELP

TRAQARKALGLPEEGTLLYASFGGGGDPEAARALTLTAQVARELPGVRLVVGAGPLWREP

PPTLEGAVVLQGRYPALDFLPAFDAAVTAAGYNAVHELLYAGIPSVFVPFERMVDDQEKR

AREVTAAGAGLDCTPLTREGLTRAVREILNPDVRQRLSAAARKKVERNGAEPAARALLEL

LA

>116227037

MPLYDYRCHRCGEVFEVRQKFSDPLLTEHEACGGEVERLISAPALQFKGTGWYVTDYAKN

GKSPSTGTNAKSEGKADSGAGKSESASESKSEAKSESKSDSKPSPAPASTSSEK

>116292707

MGDGAARAYSAEAFVWLIPQGAATSACYLAMWRYPSTASQKRELWPFRPQTSHETTGLGC

PRCSGPHSSLHCCIRRRPPTGPTCPGKPLIFSSGRAIGNPDLVESSARVDYVPALCFYMT

PFAEFLVSASHQLALTTSLPFVHTRGRVPGSVITNVISPQSLRPCPSYTPDRIGRIPRYC

ELLAGTYADNALCTYQRPFA

>118173533

MPTYSYACTECDNRFDAVQAFSDDALTTCPKCSGRLRKLFGSVGVVFKGSGFYRTDNRSD

SGKSSSNGSSSKSSENSSSSGSSSSSSDSSSSSSSSSAAPAAAASS

>118341863

MLTVSLLVCAMMALTQADHDGVLKGTATEAGEVSPVFRSRRALVCPAGWTLHGQRCFYSE

ATAMTWDLAEANCVNKGGHLASIHSLEEQLYIKDIVAGIVWIGGSACKVAGAWSWTDGTP

VDYRTWCPTKPNDILSDCCMQMTAAVDKCWDDLPCPASHASICAKAAI

>118347567

MNILPILILIFWTKQIIAQFAYSDQIQTSDLEVVPYSAILNQNYDQTVSKTVTFTNQFQN

VPKIIVGYKIFDYDRDGNIKSFELVVSKITTTNFLISFTKYQDTKVYRFVATWIAIDSIY

AFYKQVVLNFSPWPSSNNQVTVSNPFSMTQIDNTKVQRKVIAFVQGTFFNKSPSNRNTFQ

MYATVNTNTIDVNVISNDPTQSLSQIKVNYIEFYSNITTPYYDVTIINDQDFQSYNPNKY

CPWTYGKTKTLDFNYASDVNGVVAAITEYDIDFTLGQSSRLQLNLISYTPIYKQLNYNYI

INLDSSTCFYSMGAYIVAFKMYDCSQNPSTPINFSPQYQCVSQCPVGYSQQKRDNSQNFS

YCQPNPCSIANCLLCDTNGLCIQCITGNYMYNSQCSASQPANTYCDNNLICKSCSANCQA

CLNENSCTTCQSGFYLYQGSCTSTQPSNTYCDNFVCQKCQSSCSSCTNGSSCTVCNTGLY

FYQGACTSQQPDNTYCDNNTLICQSCSSNCSKCSSQNSCTTCQSGFYLYQGSCTSTQPSN

TYCDNFVCQKCQSSCSSCSNGSSCTVCNTGLYFYQGACTSQQPDNTYCDNNTLICQSCSS

NCSKCSSQNSCTTCQSGFYLYQGSCTSTQPSNTYCDNFVCQKCQSSCSSCTNGSSCTVCN

TGLYFYQGACTSQQPDNTYCDNNTLICQSCSSNCSKCSSQNSCTTCQNGFYLYQGSCTST

QPSNTYCDNFVCQKCQSSCSSCTNGSSCTVCNTGLYFYQGACTSQQPDNTYCDNNTLICQ

SCSSNCSKCSSQNSCTTCQSGFYLYQGSCTSTQPSNTYCDNFVCQKCQSSCSSCTNGSSC

TVCNTGLYFYQGACTSQQPDNTYCDNNTLICQSCSSNCSKCSSQNSCTTCQSGFYLYQGS

CTQTQPIKTYCDNNKNCFGCLDSCSQCSNGSSCTSCNSGLFFYEGSCTAAQPQNTYCGQN

QVCTKCTVNCSSCDSTLKVCNQCNPNDYKYNQQCYSSQPPQTYCDNNKNCFNCQIYCQSC

SDGNSCITCKNGFYFYQGKCTQAQPNNTFCDINLICQSCQASCSSCTNGQSCTSWQCTSS

QPNKTFCDNNLICQSCLDQCQSCSNKTSCTACQSGFYFYQGNCTSTQPSNTYCDSNKICQ

ICLSSCSSCSNGTSCTSCISGYYFYQGNCVSTQPSKTYCDSNLICQNCMPSCSSCSDGSS

CKSCNAGLYFYKGNCSPSQPDQTFCDSSLICQSCMSQCSSCSNQSTCNTCQSGFYFYQGN

CTQTQPIKTYCDNSKNCFGCLDSCSQCSNGSSCTSCNSGLFFYEGNCTVSQPQNTYCGQN

QVCTKCTVNCSSCDSTLKVCNQCNPNDYKYNQQCYSSQPPQTYCDNNKNCFNCQTDCYSC

SDGNSCTTCKSGFYFYQGKCTQTQPNNTFCDINLVCQSCEASCSSCTNGQSCTSWQCTSS

QPNKTFCDNNLICQSCLDQCQSCSNKTSCTACQSGFYFYQGNCTSSQPNNTYCDSNKICQ

NCLSSCSSCSNGTQCTTCISGYYFYKGNCVSTQPNNTFCDGNLICQSCQASCSSCTNGQS

CTSWQCTSSQPNKTFCDSNLICQSCLDQCSSCSNKTSCTACKSGFYFYQGNCTSIQPSNT

YCDSNKICQNCLASCSNCSDGSSCTSCAPGLYYYQGNCNQDQPNNAYCDSQKICKQCNNP

NCKLCDNTLNQCTQCDTNTFLYNQQCYSSQPPKTFCDSKLICQSCQSQCLTCSDASTCIS

CVNGSYFYQGSCTISQPNNTYCDSNKICTKCNNSTCNVCDSTLSICIQCRQNNYLYNQQC

YSTQPSQTYCDTNLKCQNCLTQCQTCQNATECTSCPNGNYLYRGSCLTSQPNNTYCDENK

VCNQCSDSQCMYCNQQNECIQCLQSLYLYNQQCYPNQPPQTYCDKNLKCESCHFSCETCS

APELSTTCLTCASPSTRVFDSYKKECICKQNYLEEGSIDCIEKIDVQMQVYQSQEVIFYS

SVAVSSILVLGVNLPNFQHFSFFTLFENLKIAPEASNSINQIIIFTGVLFLSLFIGLIFK

IAKCQFRMISLISFVLIFKVLFKDGMYEDSLSISVILIFLFLYSVKTALQIRGINEQKQK

QIKSKTKNNSFLESSMNIGSKNSPTPNWLFNWMLWRQFMGLLFPTCYQLYKYCNINSKKS

LY

>119376268

MSLFNILGGEFIEIIEWTDDSRDTMVYRYPTVGKAIKYGAKLTVREGQAAVFIHEGQLAD

VFAPGLYMLETNNLPILTRLQHWDHGFRSPFKSEIYFVNTTRFNDLKWGTKNPVIARDPE

FGPVRIRAFGTYSMRVTDPGRFMTEIVGTDGEFTRDEISFQLRNIIVQEFSRMIAGSGIP

VLDMAANTGDLGQMVAKAISPTVAAYGLTIPEFYIENISLPDEVEKMLDKRTSMGIVGDL

NRFGQYAASEAMLNASNQPGGMGAGIGAGLGAGMGVAMAQRGPWGAVPQQPPQQQAPQTP

PPLHHPETVWHIAVNGQADGPYGRAHLGRLVREGGFTRETLVWTPGQDGWKPADEVAELA

QLFTVAPPPPPLPKAE

>119377766

MGFGMMNYSAMQAPLMLWQQMARMAWESQMVIALRTAGMMGLLRQDAAEPQRMVIEKADA

ASEALHAALRAAGRGERADRVMAAALRPYRRRTRANVRRLSGKD

>119392500

MSDNQFPFATLGNAIGFITKLDGSVTVQSIDGQERVLKLGDPIFFGETVLTGGSGSVTIA

FVDGTDVVIGGDSIVEMTDEIYNTGDNEDLVADSSSEIDALQNAILAGDDPTLIQDAPAA

GNTLADQQRVDVSIERNDNSAQAGFGVDTQSSLPTYGYDTDNGNGGQATEREYSAPSLSR

TLNQSPLLINLDIDPVTGDSVINAAEAGGTVTLTGVVNGDVFSSGVVTLVINGVTYSTNV

NPNGTWSVSVAGSDLSADSDRIVDASVVVTNGAGQQGTADSTESFIVKTSSRATIRVNSI

TSDDVVNAEESNSTITVSGRVGLDASAGDTVSMTINGTLYTTVVLANKTWSVGVSGSDLA

QDNSFQVSVTGQDSAGNPYAGTTTSTHTVDTSADAGTVTVNAITSDDVINASEAAGTVAV

SGTATGGDIAEGDTVTLEINGETYTTTVDANGEWSVDVAGSDLAADTAFDAVVTSSDAAG

NTVDTTGSSTHTVDTEATAGTVTVNAITSDDVINASEAAGTVAVSGTATGGDIAEGDTVT

LEINGETYTTTVDANGEWSVDVAGSDLAADTAFDAVVTSSDAAGNTVDTTGSSTHTVDTE

AT

>119392501

AGTVTVNAITSDDVINASEAAGTVAVSGTATGGDIAEGDTVTLEINGETYTTTVDASGEW

SVDVAGSDLAADTAFDAVVTSSDAAGNTVDTTGSSTHTVDTEATAGTVTVNAITSDDVIN

ASEAAGTVAVSGTATGGDIAEGDTVTLEINGETYTTTVDANGEWSVDVAGSDLAADTAFD

AVVTSSDAAGNTVDTTGSSTHTVDTEATAGTVTVNAITSDDTIDGIELGQTISISGKAVG

GDISVGDVVKMTINNTEYSTTVKAGGIWMIAGVLGSDLAADSEFDVVVTSSDAAGNKVQS

IGTSTHSVDLSAEANFSLAEGQQHVLTNLPEGFGFPDGTTEVVTNFGGTITLGDDGEYRY

DAPVRDHGDAVSDKDSVTVTLEDGRTFTVNLDIQDSAPVAVDDQDSIVVQHEEFEVSEIA

ASWVSYTHGESVTTFDGTSDLGGVDNDSAKDQIRWGNPAESKQSGYGFIDNDSNLEGRFD

LNQDISVGTFTHYNYPVYSGGAITSAEMSVEFSVLDHLGVSTPVTLTVNFDHNETPNTND

VNASRDIVTVQNTHVTFERDGDIYTVQIVGFREVGNPDGEVVTSIYTNENAATSYELVVR

VVEGDGYSLPSTEGNIFDDNGLGADSLGADGSVTVVGVAVGAIVSSNESVGHSIEGQYGN

LVLNSDGSYVYDVTASVSDIPAGATESFAYLIQDQDGSTSSANLSINVGTNTAPKAQDDS

TPDSLFAGLVGEYYGTNSQLNNISDFRALVDSKEADATFEAANISYGRGSSDVAKGTHLQ

EFLGSDASTLSTDPGDNTDGGIYLQGYVYLEAGTYNFKVTADDGYEITINGNPVATVDNN

QSVYTVTHASFTISESGYQAIDMIWWDQGGDYVFQPTLSADGGSTYFVLDSAILSSTGET

PYTTAQEQALEINVDRLLDNDTDSDNGDTLSVTSISNVKNGYAYLDAEGIIHFTPVKGFA

GVATIDYTIEDGNGGRDTATVSIDVTPESILPMVTVNVSQSNSFGFWDGTSTQAEITHSF

DHYIGSAFDASNNNVAVTGNVSATLNVLAGDDKVSIDGNVEDVLVAANVAVLDMGTGNDQ

LYVAGDVLGKIDAGTGNDEIYIKGDVSAAVDAGTGNDEVYIGGNLSGDLDAGTDNDNIQI

GGDVNAALNAGTGNDNLIIGHDVSGIVNMGTDNDTVEVGRTINASGKVLLDTGDDSLLVS

GDLFGEVDGGTGNDTIIIAGKVSGNIQGGTGNDIVRVQSQVWAEANISLGTGDDVLIVEH

ELHGTVAGNEGDDSIYLKFYTKEQYNNNSDLRNRVANFEHIRVSDGVVKGSPADFADYKH

GGYSYDVSVSIDTSNANVGASTVTLLGIPIAGVTLMLAGQPLTANATGGYDIEVSSDQTA

IGGLKLISDVALTNLEITTSVNAIVEAEVSDLYLDGTGVVGDLVDSQTVDTLIGGQGDDV

LFGGDDSLVDTLTGLEGSDIFILNDTTDVLNIDTITDFNAAEDALDLTDLLTGIAGSPGK

DADVDAVTQFLTENVKVTDGHVKVGGEDVANFGSDSNFDSNGVDGVTTADSIKVIYNNEE

YSINIDG

>119669763

MKIRLSAALGALALLQFLGASPVCAAEGPRDVHALLQQGKAAEALVLATRLVEAQPRDAE

ARFARGVALAELGRQDEAISVFLKLTQDFPSQPEPYNNLAVLYAQQKQYDKARATLESAL

RTHPSYAVAHQNLGDLYARLASQAYEKALQADSTRSTETPTRLALITELKSDAARTAAGP

RASTAAAVPPAAPKGPALAAASGSPTIAAAPPTTAVAPPPAPPAAAPAKPVAPSAPGSAA

PAVAATPNSPAIPQTTAGAPPQPAAAPRAASVPVPPAAEAPAGTTSQPGADTAVAERKPA

AAPEKAAQDAVLRSVQGWAQAWSRKDVKSYLAAYDKDFEVPDGRARAVWERERQQRVGKA

GAITVEVDNPRISVNGDRATVRFQQHYRSSGFNGSTNKTLELVRRGDQWKIRRETVGG

>119670076

MSQLIEVKVPDIGDFDSVPVIELFVKVGDTIAVDDAIATLESDKATMDVPSSAAGVVKEV

LVKVGDKVSEGALLIKVEAAGAAAAAPAAAAAAPAPAPAPAAPAPAPAPAPAGAAPAAAGGV

VDVVVPDIGDFSDVPVIELFVKVGDTIKVDDAIATLESDKATMDVPSSAAGVVKEVLVKV

GDKVSQGSLLLKLESGAAAAAPASPPPLQGEGRGGDGVKPASNVPHPPPSLPLEGGGAKT

AAAPAAPSAVTLGGKVHASPSVRAFARELGVDLAQVKATGPKNRVLKEDVAAFIKGAMST

GVVPGKTPAAAAGASLGGGLDLLPWPKVDFAKFGEVEVKPLSRIKKISGQNLARNWVMIP

AVTYHEDADITDLEAFRVQMNKEYEKSGKKLTMLAFIIKASVRALQEFPEFNTSLDGDNL

VYKKYFNIAFAADTPNGLVVPVVKDADKKSVFQIAEETGALAKKARDGKLGPADMSGACF

TISSLGGIGGTYFAPIVNAPEVAILGVNKSVMKPVWDGKQFVPRLTLPMSLTADHRVIDG

ALATRFNVYLAQLLADFRRVML

>119672438

MSLAIVRARALDGLAAPEVTVEVHLANGLPAFNLVGLPDTEVREARDRVRAALVTSQFEF

PQRRITVNLAPADLPKEGGRFDLAIALGILAASGQVEAAALDHYEFCGELSLNGGLRAVR

GVLAAALAAAGAGRALVLPRANAAEAALAPAATVLPADDLLAVCAHLNRHTLLEAQPAAQ

PGPQDAALPDLADVRGQAQARRALEVAAAGGHSLLLFGPPGTGKSMLAQRLPGLLPPMDE

AEALASAALQSLEGVFDAARWGLRPFRAPHHSASAAALVGGGANPRPGEISLAHHGVLFL

DELPEFDRRVLEALREPLETGAVTVARARRRVEFPARFQLVAAMNPCPCGYAGHPRRACR

CTPEQVARYRARLSGPLLDRMDLTVEVPALGHAELADSAPGEASAAVRARVLAARELQRA

RQGRPNARLEPSAVGALCTPDDAGARLLAQAMERLNLSARAYHRVLRVARTLADLAGIEV

PGAAQVAEAIQYRRSLDAR

>119672492

MRTTALALTLLALATAAVVYAASEAPVPAATQAAAPASPLLDHSFRRLHGAETLNLRERY

AGQPLLIVNTASHCGYTGQFKELEAIHQRYRAQGLKVLGFSSDDFNQEADNEAKAANVCF

VNFGVTFDMFAPIHVRGGDAHPLFRELARQSQAPRWNFHKYVVDRQGKVVASFESAVKPD

APEVLAALERAIAGAR

>119863656

MNIIDRVKYNGPGDVFAWRWPHDSLVWGTQVIVNQAQEAVFYKDGKALDVLGPGRHTLKS

ANIPLLEHLVNISFDNQSPFAAEIYYVNKAVNLNMKWGTQEPIPILEPVYNIYIPLRAFG

QFGIKITDAKKFVTTLVGTVDEFDAEQILNYFRGHLMSRIKDFISKKVINDRISVLNISA

HLNEMSAALQNDIMAEFKRFGIEIVNFYLNSINVPQNDDSVIRLKEILAKKAEFDIYGET

YNTVKMFDVAEKAASNEGAGGVAGAGVGMGVGFGIGNQMMKLNSTSMENEQSTIMAGPSC

SSCQQTIPQGAKFCPSCGSEQVQAQVKKFCVDCGTERSLDAKFCPGCGKKQ

>121308436

MAFKTCAFTKSWLVVAVIVMCLCTGYYCQCTGGTDCTSCTSACTSCANCPNAQTCTNSGN

CVNAQTCTTSTNCNRATTCTNSQDCFAATTCTSSSNCYTATTCTDSTNCYAATTCTNSTG

CPGYEVLLLSKIIEHDD

>124511116

MPPVIVGIAAGVAAAGVVSTAAAIAIGIGAAALTYAATPKFDSGSFANEAFSQQQMLRSP

VEPRRGIYGRAMVSGPLVFAEETGTDNAYLHLVIPLAGHRCDGIEKIYFGDEVAWSNGVM

SSKYATFARLKIHLGNQTTSDPNLTTECSQWTSSHVGFGVTYLYARLKFDTTVFPNGVPN

IKALVRGKRIYDPRKPTHNWNDPATWEWSDNWALCCLDYNRFESGVGAAAHEIDVSYFAA

AANDSDQLVEYKTGKFEKRYTCNGTYNQDVSPSSIMEKMLTAGAGMHVYVSGQYRLYAGV

YQGPEVLVLTEDDAAGDIDVRPYTPRSDLCNAVRGTFVDPSNFYQPTDFPPYESSYYRAQ

DDGEYIDHDLDLPFTQSVWTAQRLGKLYLEQKRAGMQISMPVKMIGVAVSVGKVVGLSLP

RLGIDGTFQIVDWQFDYGKPVSLILVETSPELFDYAMGSYTERDLTPNVILPNPATVPTP

VGLQYLEYQDDPQWQGELTWSAPANNSSYRYVIEVSNGLGEVVYQTNADATRLLLPKFDT

GFYTVSVWALNLFANRSNVPATITIGATLPAPVTGIEVIAGPLTLTLRPTTAAQIAQTTQ

FEILGSITNNIAEAALIGTGKEAVWPDRHPNTRYHVWARTINNYGVSSWYGPVQATTSAD

NSSIIDLIGNTFKNYTWFAWADDNQGTGFTTVEALGDGKAYMGLATDKPTASPSGNWQDY

TWSKIKADIGPVFTPEEEAKLDNLLAGKLPNAPDKDMLAAQDALNNPALANNLITALSLL

NQGINAPALGGETPSGAQSKASSAQSAAIAAAATDATTKANNAKAQAEANAALDALNKAN

TAKADAISTASTDATTKANNAKAAAESYALAKANLAETTAKAYADGIVSDEEARAIADAQ

AKADAAKAAAIAAAAADATSKANNAKAQAEANAALDALNKANTAKADAISTASTDATTKA

NNAKTQAVSSAATDATTKADAAKAAAIAAAAADATTKANNAKAQAEASAALDALNKANAA

QSAAEQFTRKAVAEFISSHPSIVAVDGNTVYCYAPPNGAWSSGAYSSEGFAGGCILTLRL

RAIPAMDIGRWMMGINDDPLTNHNYNSIDYAFYIRHTGNIECFESGYGKGIIGTFVAGDI

LQIKYDGANVTYLKNGSVLRTVATAAGRVFYMDSSFHGADANSGAEAIVFAPTEGSQVSI

DAAKASAIAAAAADATSKANNAKAQAEANAALDALNKANTAKADAISTASTDATTKANNA

KTQAISSAATDATTKANNAQASAIAAAAADATTKANNAKAQAEANAALDALNKANTAKAD

AISANDILGLTTYGNYSSVLGATCGHNSPHASWDGSVYSKLSHNGGASITVTVESLSGSS

RRVMVGLTTIPSAIARYTDINFAIYADLGRYSVYENGSSKGVILDRAPAIGDIINVKYDG

LKVYYSINGTVLKETVASRDLTLFFKAAFYGSGFYLSNITFTTLSNLVVASDDALNKANT

AKLQAIGDVAAGNFAAQTALITTLLANSGLFNQLKSRLGSFGGLTAETIAALAIATNHLQ

AGSVTVDKVGANAITAEKIAALAIATNHLQAGSVTVDKVGANAITAAKIAALAIATNHLQ

AGSVTVDKVGANAITAEKIAALAIATNHLQAGAVTVDKVSANAITAEKIAALAIATNHLQ

AGAVTADKITADTALIQKLIANIGLFNQLQAQLGMFGGLAANSIAAGAIQTDKLAILARE

YVNPVSISGNLIGWGGVLEDGTGGNSAIISYDSTEKALVITSNSMHSVWCKSWKVNHDKI

YRVSMKIKASVAAGLYFIGAQAWNYGNVGGTAQSANAQGNMLLYRYSPIRAAQTSSINSY

FVNSGVPTTDYIDHTVYLIGANRDVNDCPAHAGPSSSPFIKLDAAATDAGIRFLNWSNTS

TKKLFVKDISVTEVGAGQVVAQNIAANAVTADKIAANAVSAGKIAANAVTANELLADTAL

INKLVATSALFDSLVARLAVFGGLTANSIASDAILGRHIKAGEKISSPIIEGGEVRLIGS

NIMKIESATPFGPDSLVYWRGPRLLVGGNPDWPNIRKSNATRWTDSTGDEYFGGSLSAGV

LKTGISNPTKTVYQVGTYIVELGPFSSNGKPRNIIISYGVNGYAQRTTAPNDMVNPSLGW

HLERKVNNGGWIVVTSGHFQGASEYEFDGESRMFWVSCICSGSLTYTDNLTTAGEYSYRV

KVLSYTHYLRDYQSIGITSTEQ

>126708477

MLALMAAEASAVITMRTLGFMGIWSVPKSENTLMVSEKQAAIAEAGHGLIRAAWRGETPE

AVYDAVVLPFDRAARANRRRLSQRGFRRP

>145568689

MPTLLHDKGTPMKAPAQKTLRRVLFALVALVVIGLLAWSELRTDGLGDGFASGNGRIEAT

EIDVATKLGGRIREISVDEGDFVQPGQVIARMDTEVLEAQLNQARAQVRQAENAILTAQA

LVTQRESEKATAEAVVLQRRAELTAAQKRHQRTETLVGRNAMPRQQLDDDLAAMQSAQAA

LAASRAQVLSADAGIAAARSQVIEAQSALEAAQASVVRLQADITDSELKTDRVARVQYRV

AQPGEVLGAGGKLLNLVDLADVYMTFFLPERQAGRVAMGSEARLVIDAAPQYVIPARITY

VASVAQFTPKTVETESEREKLMFRVKARIDPDLLRKHMEQVKTGLPGVAYLKLDADAEWP

AHLQINVGR

>146191040

MKAAQHTKQQLRATALAARDALSEDDRAAAAQAIAARGLPFTPKPGMVVSGYAPIRNELD

PMPLMRELAGQGARLALPVVLARGHSLSFRAYAPGDRLTLGALGIPEPSPVAAELVPDIM

LVPLAAFDRTGHRIGYGGGYYDYTFSHLRKSHHVIGIGLGFAVQETEAIPALAHDAALDY

VLTERETLDFRSH

>157694790

MLTVSLLVCAVMALASADDNSTTSIVDVSVAEDNSTTSLDVNAAEDNSTTSKKEEVAPSC

EIGWSEFNGRCFLFVSTEMSWADAEKNCLLKKGHLASVHNEEEYKHIQAVVNAHTGGHPT

TWVGGSDCQKEGIWLWSDGSGFEFDSWCEGEPDNYVGAESCLQINANESHCWNDFPCSTV

LPSVCASVPQSS

>158329021

MTLSSPTTFRSEKARLRAEALLRRADMGAERRAAASDAAARHALMALGSVAGRTVALFAP

FRDEIDTGPLARKLRDAGARLALPVVIGRDRPLIFRLWDENDLLEPAGAYGIPTPGAQAP

QVTPDDLMVPLAVFDRAGARIGYGAGFYDRTLALLRREKPIRAFGLAFACQETDHVPAEP

HDEPLDGMVTEAGVLFLGGSDADSLSR

>159121621

MPIYEYACQACGHKFDTLQNSSDEPLTHCPICQEPTLKKLVSASAFHLKGTGWYVTDFKN

PTKPENTEKDKADNTTTPKEQNKGTDNVQESTAKERVAEKKSVDKPPKTDPSEKKS

>159163462

GSSGSSGSVVAKVKIPEGTILTMDMLTVKVGEPKGYPPEDIFNLVGKKVLVTVEEDDTIM

EELVDNHGKKIKSSGPSSG

>160369818

MALSLFTVGQFIFLFWTISITEANIDPAARAAAAAAASKAAVTAADAAAAAATIAASAAS

VAAATAADDAAASIATINAASAAAKSIAAAAAMAAKDTAAAAASAAAAAVASAAKALETI

NVKAAYAAATTANTAAAAAAATATTAAAAAAAKATIDNAAAAKAAAVATAVSDAAATAAT

AAAVAAATLEAAAAKAAATAVSAAAAAAAAAIAFAAAP

>160430555

MAIVEVVKYDGAPNVFAWKYPNNELGTWTQLIVNESQEAVLFKGGKALDVFGGGRHTLET

ANIPLLNNIINLPFGRRSPFSAEVWYVNKVYSLNIKWGTTSPIQIQDPKYGIFIPVRSYG

QFGIRIEDSKKFLIKLVGTLNVFDDNNILQYFRGLYLTMAKDTISSYLIQKKISVLEINA

YLDEMSNYIVERIKPTMDEYGIGLTNFYVNDINVPEEDTAVKKLKDALAKRAEMNIIGYD

YQQQRSFDTLESAASNQGGVSSGIMGAGMGLGMGINVGNAIGTQFGSVSNALNTKGGNKK

ECVKCHAAIEAGTRFCPSCGNDTSKTESENSNTIKCDNCGAFYSANTKFCPECGNRYNPC

PNCKADIPDGATNCSSCGYELPIPCPGCGNLVHSKKHKFCPECGTPLVKKCPKCETVIEG

SPKFCPECGEKLS

>161381453

MNPFALFAMQSQLASLAVETQIVMSLRILAMAGALPARPGENNRMVAEKGPAMAKAFHAG

TQAAMSGKSPDQIMNASLAPLARKVRQNRKRLMK

>167867796

MAYISLNWFEAVEYCNRRGMQPAILDSQAKHELAVREAQATGRQSSGFFGLWLGASDLAK

HQSYVWQPTGAPVLWAKWSPGEPTGDPEHCMNLYYWPDRGFEWTVNDAPCDTTLHALCQE

RSTKKPTGQTCVDCTVQYHISSNELTWFESIEYCNRRGMRPAVLDSQAKHDQAVREAQAT

GRQSAGFFGLWVGASDLAKTGSYVWQATGAPVTWAKWSPGEPSGPPEHCMTLYYWPERQF

DWEANDAPCDTTLYALCQKEVAN

>167880585

MFKTVFLLFGFGCLLVCAQKPEPKEYVVIQKESNWIGAIEFCHRIRMQPAIVSSAQQQRQ

IEEAVAASPEPTPGPMGKKTYYWIGASNLSSKRWFIWEPTGQPLIYAKWEANQPSPKFNC

VALGYSASTLERGQWSTADCDQQLKQFVCEASPR

>182636254

MALARRAIVPAETARAFAAVLAETGPQLAKDHRAEIIGAYAAIGDEVATSPLLQMLAQAG

FATALPVTGKRGTPLTFRLWRPGDPVAKGKMGITEPPATAPEVFPDLLFVPLAAADRAGN

RIGYGAGFYDLTLAALRSQRPICAIGIAFSCQIFEAVPAEAHDQKLDYLLTEQGLIACLS

SRPFTD

>198265564

MGILDFLTGEFIDVIEWTDDTRDTMVWRFEREAHEIKYGAKLTVRTGQAAVFVHEGQLAD

VFTPGLYMLETNNMPIMTTLQHWDHGFKSPFKSEIYYVNTTRFNDLKWGTKNPIMLRDPE

FGPTRIRAFGTYSVKVTDPAKFLTEIVGTDGEFTMDEISFQIRNIIVQAFTRTIAASGIA

VLDMAANTADLGKLIAGAIADTIAEYGISIPELYIENISLPPAVEAALDTRTSRGLTGDL

DAHMKYAAAEALRAGGEASGAMGAGMGAGMGMAMAQQMANPWLNSAAQQIAPTAPQAAPP

PPPVEHVWHIAEAGKTTGPFSKASLGRMVVDGTLTRASIVWTQGQDGWMAADEIAELAQL

FTVMPPPPPGA

>198429567

MKIIFGVLLVLCIVALAVDARGGRGGRRGGGGRGKGRGGGRGRGGGRGRGRPAPTPAPVC

KWCFTSDRCYSPADTTPNAACVDCLTTACARDLHDPYGSKLGLDCFERQWTRTCSAQCGA

FNQTRFDACHDCLDICHEKIGDTSPPPVVCKSNFQNRNPCVSCNTPTSCYNSSELWSMLG

GHWRCARSVYWTCFHGGMVNVTTCMQRWSDPNTGCGSVPMPADLQARLNACNPCKTACKT

VGACVANCSRNEVYPKVIPCAICNEFCNATNECQASTCAPYCNTCNACYTRCEVGNFGFE

NPCNCVNTDGPTGGYGPGNSYGRARGRGRRSVRGGPGGRGRG

>206741909

MPIYEYECMQCHKIHEVIQRFTEEPLKNCPVCGGEVKKLISQSSFILKGSGWYVTDYARK

NNSGNSSSNSNKTSKKVEGKST

>208970877

MLTVSLLVCAMMALTRANGEEDTSINGTVTNDTNIDVLNARNGCPPGWEPFDGRCFKLVT

SRLTWAKAEKNCQAFGGNLASTRNSEDYNFIQQMTTELTWIGGSACQETNAWFWSDGTPM

DKPLWCAGQPDGALAQCCLQINTGDGKCWDDQPCRNLLPSVCIKK

>209732078

MKVLIIFALLCVALSARAAAVPVESDAVAVEEPKSAPEEVVEVEAPAAEEKLSDAQGVFS

EAENEEVEVAAAAPKDKEARRFCPDGWFSYQSKCYMFVNTPRSWFGAEEHCNELGASLAS

ASSSPEYRYLQQITRTANRATAWIGGFYLQGTWMWIDRSGMYYTNWYSQSTATSNSCMYL

QSAVGQGWRNLGCGTQYPFICVHNYRC

>209735910

MKFSLVAALVVVLAIGCESSSLVKRDIPAEIETLTKYFQDAIETVKSHELISQAQGYLEE

GKTQITPLTDKIQEHAEKIQEQMKPFVSDIEKQVRPIADNLQAQFMPLVDKMQAQFKPLA

DDLQAQMEQLFQTVVDQTKALLPPQ

>213954649

MAIIDVVSWSPQGSRVVYAWKFPETNLATYTQLIVQESQEAVLFSKGQIIGKFGPGKHTL

NTENIPILRNLFGIPFGGKNPFTAEVWFVNKVQSFSIDWNVSGMPIHDPDYNTQLPLVAV

GRYGLKVNDSETFLIKAVGTKSEFTQDDLTNQFFGEFSTKTKSQIAQYVIKHRVGYKYIS

AFLDELSGYLKNVLNPFWTNLGLELLQFNITNIDIDKSTEEGRKVAEAIATQASMSITGH

TWQQEQMFNTANNAIEGFGNGNGGLLGGLMAMSMMGGMNNSGMAGGMMQPQYNQPTFGGN

QQVGQQGEQAPIKMVFCSNCAKKYPSNSSFCPHCGNPYNPCPNCGADNDTNAKRCVSCGT

QLQQTNNITPEQTIADVCNRCGTVFPPSAKFCPKCGNKR

>218683619

MSFTRNHQTALLLATAMVLGVTMADGPSRVNLLKAGKFALLTKTGVTTTGITKVKGDMGT

SPIARAALTGFSLVADSTNEFSGSPLVTGNVYASNDAVPTPQLLTDAVLDMQAAYTDAAG

RPDPDHLNFGAGSIEGETLLPGLYKWDAGVSFTDGVTFNGSSTDIWILQIGAGLNVGSGA

KVKLAGGAKVKNIFWQIVGPAVLGTGSHVQGVFLCKTNMVFQTGSSLKGAVLAQTAVTLD

SARITKRSFCDITVGCETS

>219675136

MPIYEYQCTECGELHEALRKISDPPLTDCPACGEPTLRKKVSAAAFRLAGSGWYETDFKK

SGNKKNLAGDNSPDKPSGDKSGDKKDSSATKTPKPDSSGSSAA

>219680643

MPIYEYQCGQCHHSLEAIQKVADAALTECPECGAQALKKRISAPVFRLKGGGWYETDFKT

GNKKNLAGEKGADGAKTNTAEKSAGSTEGAAKKPGADAAATAKSSS

>219999175

MAIIDRVKFDGLRSRDWIVYKHPVEDLVYGTQLIVGEGQIAVFVKGGEVCDLFTPGTYTL

DAKNLPILRVFVNLPFGGKTPFSAEIYYINTTTKLDINWGTSDPVQIIDPKYYTRLRIRA

FGQMGLKLDNYETFFRELIGVMNPADIIKFNNVIDFFKGMLIQKIKSIIANVIVNQKISA

LEITARLDDIANYTFDLIAPEFAKYGLSAISLIIKSINFPDEDFEEINKILKNKAEFEIM

GDNRYVTKRSFDVYDHAASNNSGVAGAFAAGGVGLGVGLGVGASLGSDIHNTINTRAAAD

KATIACPSCKAQNPAKSKFCCECGKLLETPKKVCASCGVLIPGDPKFCPECGTSMGTQTC

ECGAEVPVSTKFCPQCGKKL

>220976749

MTTAHRPTWKAAVGRAQEGGWAAGGALSTNSSAKDLASHTKLKFRKVGDELREALLQESL

LKMEEAERSADLTAKRYNGEEEEERGRQLLLKQTADVDEGEIKKRYDDEDDVGADDDEEE

DDDDLDSDDEDEEAALQAELAKIRAERAAAKAKEDAEAAAEEQAQMEEAALTGNPLLNSS

SSAAAAASSGRIKRRWNDDVVFRNQAKNEPDQNKKRFINDTVRNDFHKRFLNKFI

>221115558

NWGDWVNGTCSQPCGEGYLTRTRFCNNGLQCIADGLYKSVQIQDNIYCNLKPCDCNGGCI

DKFSTCVNNVCQCQGNYSLINGNCTGNWGNWVNGTCSQPCGDGYLTRTRSYCNGGCTGQF

TTCVNNVCQCQGNYSSINGTCTGNWGDWVNGTCSQPCGNGFMTRNRTCNNGLQCITGGVY

KSVQIQNNINCNLKPCDCNGGCTGQFTTCVNNVCQCQGNYSSINGTCTGNWGDWVNGTCS

QHCGNGFMTRNRTCNNGLQCITGGVYKSVQIQNNINCNLKPCDCNIGYVRNSTTLLCQRD

SNWTAISDCNAVCNGNQTFQYNNVPFTVVDEPCSLCQGYCGVNSTCLCSNDAQYADSNGV

CQNYCNIGYLRNSTTQLCQRDSNWTAISDCNAVCNGNQTFQYNNVPFTVVDEPCSLCQGN

CGINSICLCSKNTQYADSNGVCQNCGVNERVFLSSVTCDCDIGYLRNATTLLCQRDSNWT

PTSDCNALCNGNQTFKYNFIPFTEVVEPCNLCQDCDIGYLRNATTLLCQRDSNWSPTSDC

NALCNGNQTFKYNFVPFTEVVEPCSLCQGYCGANSTCLCSKNTQYADDNGVCQNCGINEH

VIPPSTTC

>221157589

MPLYEYRCRVCGYRFEKLVRLSQADQPIACPNCGTSETERLLSAFAARTSEGASSATSCS

TGGG

>226518533

MMDARPVNVGLGSAPAQRGVKRQRDWSDKPTPKSGFCEHGRRRSRCKECGGSAICEHGRQ

RAQCKECGGSAICEHGRQRSHCKECGGASICEHGRRRSQCKECGGSQICEHGRHRSQCKE

CGGSQICEHGRRRSVCKECGGSEICEHGRQRAQCKECGGASICEHGRVRSRCKECGGASV

CEHGRQRRYCKECGGASICEHGRQRAQCKQCGGSAICEHGRQRSHCKECGGASVCEHGRR

RSQCKECGGSQICEHGRHRSQCKDCGGSAICKHGRRRSVCKDCGGSAICEHGRQRSRCKE

CHGSSVPIGSWVF

>226521577

MTTVWEPDLDELLAIVAETEGRASEEEEDASNKGTKRKRAPYTKGPCEHGVKYRSKCKVC

SACPHGRERRYCKDCGGSKICEHGRQRDYCKECGGGAICEHGRERHRCKECGGSGICEHG

RRRSRCKECGGSGICEHGRVRSRCKECGGGSICEHGRERSRCKECSGSGVCEHGRERSKC

KECGGASICEHGRQRSHCKECGGGSTCEHGRERRYCKECGGSGICEHGRIRSQCKECGGS

GICEHGRRRSDCKECGGSQICEHGRIRSTCKECGGSQICEHGRQRSYCKECGGGSICEHG
RRRSRCKECGGSQICEHGRERSKCKECGGASICEHGRQRSQCKECGGSGVCEHGRQRTRC
KECGGASICEHGRVRSQCKECGGGGICEHGRQRSKCKECRAAKAGTHS
>226716086
MMKPEIRRWLVRGLAVAGVVLVALLVWKLVYSGGEDGRFARGNGRIEATEVDVAAKSGGR
VAEILVNEGDFVQAGQLVARMDQASLAAQLTQAQAQLANALSSRETMLAQVAQREADVVM
AEAVLVQRRAELDVSGKTHARSKALLADRATSAQQVDDDAARLRNAEANVAVAKAQIAAA
RAALQAARAQVVQAQAGIDAAEAVVKRLEIDLADGDLHAPRAGRVQYRIVQPGEVIAGGG
KVVSLVDIADVYMTFFLPEQAAGKVGLGSDVHIVLDAAPQYVLPARVSYVASVAQFTPKS
VETQDERQKMVFRVKARLDPALLKKYAEHVKTGLPGMAYVRLDPAAAWPAKLEVQLP
>226716294
MTIGYRKFLCLFASGLTLRYNFLFSEPCGHCGCNRHVRVHFVIFAALQHLVTCNRSKQGF
LPEGCMPIYEYRCTDCGHAQEHLQKMSDAPIAACPHCGGGAYAKQLSPTGGFSLKGGGYY
ATDFKNKGASCPAAQANGGGCGGCG
>226731877
MLTVSLLVCAMMALATADDAYVTSSNSSSNVSNNSSSNSSSYAEGPACPASWHKYNDRCF
LFIPRTLDWSEAEKNCQSSKGNLASVHSVEEYQFIQMIIKQQTHGNPITWIGGHDSPKNN
VWFWSDGRPFSFTFWCAGEPNNGYGNQDCIQMNFGEHNCWDDVQCSIKLPSVCARNP
>227844233
MGLIQAVAGAIGGAISDQWLDAIVATDMGEGIVFVRGHQARRHNAGGDIITDGSKIIVYD
NQAMLITDSGKIVDFSTDPGAYTFSSATTPSLFNGNFEGVLRDTWERFKFGGAPSGQQKA
FFVNLQEIKGIKFGTPNAVQYFDNFYNAELFLRAHGTYSIRIVDPLKFYAEVVPRDAERV
HIDDIKNQYQAEFLEAFSVALNKMSVDGIRISQVQSHVGELSKYMRDALDEEWEATRGIE
IQAVGIASISYDEQSRKLIDMRNQGAMLQDPSIREGYVQGAIATGIQNAGSNPAGAGSAM
LGVGMGMQAGGGFMQAASASNQAQMAAQPQAQGMSGTGSTTGGGTGATNSPGARFCSNCG

TQFGQPKPNFCPQCGQPVA

>229366598

MKTLTVSALVCALMVLTRAAVLPDGMPVKDQIAKSHLVKRSVSCPGGWSPFNGRCFRYFP

RPLTWAKAEKNCESMGGNLASVHNILEYHEIQRLILSGSHEYKQTWVGGSDAQEEKQWFW

ADGTPFRYLNWCDREPNNSRGRQHCLQVNHGAEKCWDDVECYLRKPSVCAKKI

>229433188

MALVDIVKYSGTLEEFIWKYPRDDLGTWTQLVVNETQEALLLKDGKICDLFTTGKYILES

KNIPILNKIINLPFGGESPFKVEVWFINKRYNLDVKWGTPSPIQLQDAKYKIFIPVRSFG

QFGIRIEDSKLFFSKIVGTKTIFTKNEINLFFRGLYLTKIKDMISSYLVKRKISILEINA

YISELSDYISDSMKQVFKGYGIELVNFYINDINIPEDDSGVKRLKEALAKRAEMDIVGYN

YLQERSFDTLEGAAKNAGTSSNFINSGIGMAMGVGLGNNFSNKVNSNLLINTEEEKIKCI

KCGTELKRGAKFCFECGEAQQKKCPNCGALQENQNAKFCSECGIPLVKKCKKCGKVVEEN

IKFCPECGNRID

>238523662

MFLREDTMPIYAYRCEACGYAKDVLQKMSDAPLSQCPECGKDAFRKQVTAAGFQLKGSGW

YVTDFRGGASGSSAPAAGDEKAGGEKAGGETAAAPAAADSTASAPAASTPAPAAAPASST

>238715887

MQKFSDAPLTQCPACHQPALSKLISAAGFQLKGTGWYATDFKPGSKSKPDSSS

>242276178

MAGIINKIGETLHVGGNKKEDEHRVESHGDYKGERHDEKTGVLHGLGGHKGESHGDYKGE

RHDEKTGVLHGLGGHKGESHGDYKGDGEKTGVLHGLGGHKGESHGDYKGENTGVLGFGGH

KGESHGDYKGENTGVLHGFGEHKPDHYGHGEEHKEGLVDKIKDKVHGDPGHVKGEGVVKK

KKDKKKHEHGHEHGHDSSSSDSD

>247547816

MAVVEVVKFDGPPDLFVWKYPNQEMGTWTQLIVNETQEAVLFKGGKALDLFGPGRHTLST

ANIPFIQSLVNLPFGGRSPFTAEVWYVNKINSLDVKWGTASPIQLQDPKYRILLSVRSFG

QFGVQIEDSRKFLIKLIGTLPAFDKDALVKYFRSLLMMNINELITTYLAVKKISILEINA

YITEIAKHIEERIGPVFQDYGIKLLNFYIDSINTPDDDPTTNRLKEALAKKAEMDIIGYT

YQQQRTFDTLEGAARNENTGSSGIMGAGIGLGMGAGIGGAFGGQMSGLTQQMNMTADKPC

PQCHTTNREGSRFCNSCGYSFVSAPSAPAVGPKETECSVCGKSFSAAAKFCPHCGDKNRL

CQNCGTDNPDEAEFCVKCGHTVGIKLCSSCGDPLVAGAKFCPNCGASAVLKCGQCQHEVK

PGQKFCLECGNKLIP

>253984525

MIISKQITPYIVYYEDSIQHALQKISSNTRRLVYCLSETGVLEGLVTDGDFRRWVVSTQE

INLQKPVQQITNTSFVWVREGESPEKIEALLSNKITSIPIVDEAGRLTAIAWREEPVLRF

GNREIRHEKPAFLIAEIGNNHNGSIDLAKRLVELAAESGADCAKFQLRDMESLYANSGEA

VSEDLGSEYTLDLLKRFQLSVDEMFEILDFTRQCGMVPLCTPWDLASLARLDEYGLEGFK

IASADLTNHALLEAAARTGKPLLLSTGMSLEQEIQDSVALLKQQGASFALLHCNSTYPAP

MSDINLAYLDRLREISQSPVGYSGHERGFEVCIAAVARGAKIIEKHFTVDRNMEGNDHRV

SLLPDEFATMVKAIRNVEEAIGSSSTRTISQGERLNREVLAKSLVAARAIRKGEKISEEM

INARSPGRGLQPSRLNDLLGISACRDMAPGDVFFESDIMGESAQPRPYHFDRPWGIPVRY

HDFRELTQDVPMDFVEIHFSYRDLDLAPSSYFSSPSSLGLVVHSPELFAGDHVMDLSSED

DAYRSRSIAELQRVINRTRDLKRFFPRTERPMIIINAGGFTSDHFLPETRRQVLYDRIGA

ALAEVDSEGVEIIPQTMPPFPWHFGGQRYHNLFMDHIEIARFCEKYGTRICYDISHSKLA

CNYFGWSMQHFTRDVGKYVAHLHIVDAKGHHDEGLQIGAGDIDFESLARDLKQWSPGVSF

IPEIWQGHKNGGEGFWIALDRLEKSFSAQRKEEVID

>254980987

MPIYEYLCNSCGAEKEHLQKINDAPIAVCPVCGSNNYTKLISAAGFQLKGSGWYVTDFKN

KTKPVETKPAAKESTESTPSAAPATADTVAKKESSPSTTAAATD

>255039719

GNIVVSPATSTAITGFSLTMDSSGEFSTSTQVTGSVYAPDYVGSALTPVVNDMMGAYEDA

VGCATSDDDATTNLGAGNLGSLTLEPGVYTFTTSANIGGDLTFTGDENSVFIIQTTGNVL

QTAATNMILDGVLAKNILWLVAGSVTVNAGSHME

>255296128

MPVYVYQCKNCNHVFEQRQSFSDDALRICPQCGEETLRKRYNTVGVTFKGSGFYSTDKGN

S

>255349292

MSLITINHTLVVTALLFAAVALLGVPMAEGLRQEKRGGLRRQLDDEPLSAVKLLTAGRFA

ILTKTGVTTTGPTDLKGDMGTSPITGAAITGFGLITDPSDTTFSTSSLVTGQVFASDYTS

PTPNMLTVAVLDMQAAYVDAAGRPDPDYVELGAGNIEGLTLEPGLYKWGTDVGFTNSLTF

DGSDTDIWILQIDGDVTAGSGAKVKLINDAKAENIFWQIAGKTDLGTTSHVEGVFLCSTA

ITFKTGSSMNGAALAQTAVTLDSATIVKESVCDVDVGCVAPN

>255692874

MAIIKSIYWEQNNQEELVYKFPFNNVTLGSVLTVNESQEAFFFKSGTLYDSFTAGRHTLS

SANLPLLEKLINLPSGGDTTFTAEVWFISKLDKRNMLWGIGGLRVVDPYFQIPIKLSARG

QYGVRISDGGLF

>255709878

MSLLSIITIGLAGLGGLVNGQRDLSVELGVASNFAILAKAGISSVPDSAILGDIGVSPAA

ATYITGFGLTQDSSTTYATSPQVTGLIYAADYSTPTPNYLAAAVANAETAYNQAAGFVDP

DFLELGAGELRDQTLVPGLYKWTSSVSVPTDLTFEGNGDATWVFQIAGGLSLADGVAFTL

AGGANSTNIAFQVGDDVTVGKGAHFEGVLLAKRFVTLQTGSSLNGRVLSQTEVALQKATV

NSPFVPAPEVVQKRSNARQWL

>256590641

MLIDRNLAKYIVFSEDSILNALKKISDNKSRIIFSVTESGVLEGILTDGDFRRWLVGQDT

IDLNQPVVDISNKHFKYAAFDEDPQKIQGYFSDTIEFIPLLDQNDHLVAIARKRPAEIQI

GDFTINNDSPSFIIAEIGNNHNGSLPLAKQLIDEAIASGANCAKFQMRSLKSLYSNAGDA

NDASEDLGSQYTLDLLSRFQLSPEEMLEAFDYCRERGILPLCTPWDLDSLEILEDYGMLA

YKVASADLTNHELLKALVKTGKPLICSTGMSTEAEIKESVQLLQRLGGIYVLLHCNSTYP

APFKDVNLNYLTRLKELGDCPVGYSGHERGIHVAIAAIAKGAKVIEKHFTLDKTMEGNDH

KVSLLPDEFRAMVEGIRQVEEALGTTGERRLSQGELMNRETLAKSLIINCNLSPGQMITA

EMIEVKSPGKGLQPNRKSELIGTRAKRAFKAGDFFFPSDLEEAQIQPRSYSFKRPWGLPV

RYHDFKKLLVKSNPDLLEFHLSYKDLEQDINQYFDREYDLDYVVHSPELFAGDHVLDLCS

VDPNYRQHSIEELQRVIDLTRSLKPLFKKALKPLIVTNIGGFTSDAPLAMAKRQKLYDLL

IESLEKLDQEGVEIIPQTMPPFPWHFGGQRYHNLFVDPDDIAQFCSSYGYRVCLDTSHSK

LACNHHNLSFKEFIQQVGPYTAHLHIADAEGLDGEGLQIGEGDLDFPALAEDLAKTCPQA

SFIPEIWQGHKNEGEGFWKALEKLEESEF

>256686716

MPSYAYRCKGCDHSFEIYQSFSEDALTTCPQCEGPLRKVFSTPGVTFKGSGFYRTDSAKK

ASSSSSS

>257049854

MNLESIQEKLFERKLDLIEYFKAAFDIYKEFLKNNKLLVFSTCLLLMLITSLDIFNKYLM

ALVVKYREVKIAFLVMMLLILELILSVGHSFLIGYYFKKIVMGIEGTENSFEFKKFFLKM

LKFIGIQYCLVTFFLVVSLELNLLHLNFIDLMLKIVLIAMFFKYFLYFETYYVRDFKIMD

SIEYSHQLSKANRLRKIIPEVIFILISIVFVFIISLGISKIFGINYQIEIITTVTSIIGF

VFWAIYFQVLSISIFLNVEYDYLKNQEEKNINSDSNLDMESETIDCKNEENDN

>257196605

ALSNLLTYGSNGNQVTAKDQTAAKASYAALQQEIMADYADMKAAVAAADTLEAKQAAATN

ASNAMSQKVYNTTLKM

>259347286

MREKTGQPCDPLPGFAGKLRFKLAKTPPKRAALWFQDQWIATSWETAVGGNTLGIFDFLK

GEFIDVIHWTDDTRDTMVMRFEREGHAIKYGAKLTVREGQAAVFVHEGQLADVFTPGLYM

LETNNMPVMTTLQHWDHGFQSPFKSEIYFVATTRFNDLKWGTKNPIMCRDPEFGPVRLRA

FGTYSVRVVDPARFLTEIVGTDGEFTMDEISYQIRNIIVQEFSRAIASSGIPVLDMAANT

ADLGKLVAAEIGPVVAEYGLSIPELYVENISLPPAVEQAMDKRTQMGIIGDLGRYTQFKA

AEAMEAAAKTPNSGMGAGLGMGMGMSMAQQMSQAMQGGASSQAAGQTTGPWGARPAPAAS

HPSPQAAPMAPPPPPVEHVWHIAENGQTSGPYSKARMGRMAQEGQLTRDTHVWTPGQDGW

MRAGDVTELAQLFTILPPPPPPPPPAGS

>260080993

MRSNFHPLAASFIVRCAFLHSRRFTDSLFQLLSSLISLTSAATAIDLGVAGQYDVVARSA

ITLGALAEITGNVGLSPGLSTALTGFTLVPVEDHGTFCSAGVKYCGADSLSTSATSLLVK

GRIDAPDFPSSPAILGQAATDVVAAWKSAFSQELSPADYTKRDFAGGLLSDLTLAPG

>260858626

MKKFIFLAMILMLASSCGVIQRANMRRAIKNSGGSCEYVSGVGEICAVPIRQ

>261281858

MAIIEVVKYEGPPDVFAWRYPNQELGTWTQLIVHETQEAILYKGGQALDSFPAGRHTLST

ANIPILSNVINLPFGGKSPFTAEVWFVNKLRSLDVKWGTSSPIQLQDPKYNIIVSLRAFG

QFGVQISDPRKFLGTMVGTLPTFDQGTLVKYYRGVLMSNITEIISSYIVRKKISVVEINA

YIAEISKHIMEAIAPSFEEMGITLLNFYVDSINIPEHDPAAVRIKEALAKKAEMDIIGYT

YHQERTFNTLEGAAKNPGSPAAVMGTGLGMGLGMVGPMYETARHMFDHAKPQEKNDLQIE

QKACTKCGMLNGEEARFCSGCGQSFQTQAEEAPAKAILCNDCGQPLAPNAKFCLHCGDPY

HACPKCGHDHPAGAAECPDCGAPLPMPCRACGELVDAEAKFCPHCGSSHALTCPGCDHEI

KPGQKFCMECGHKLM

>261373699

MKLLRTLAVAATLALVACGDENAVALNEPPVSSQEDSSSSSDVIETSSCSETVVTSSSSQ

TPSTEISNGSIITDGQVIDLRDGRTYKTTVIGNQVWMAENLKLDVTQYVDDGAYKDEYNF

YDALWSGVRSREISDTSEHFYPWDIAIDTAGIFGSSAKGCDHETPCDLTGMVRGICPEGF

HVPNPTEIEQLTRAIGGKCGTSKKLKAKQSGWIFDNGTDEFGFAAIPTGYNVVYWGDGMN

GFTFDYELNPQGKFLTTVDTLTWNIAIDADYAISYREEYEVVDFTEEYHWYMCSLRCLRD

EPAGVDWVDPPEPTSPALPEFEYGEFTDERDGQTYKTVVINGKTWMDQNLNYVFDVVDTN

AACRLNEENDDDEEYEDECKKSSDYSCKHPLIGSPVSCKNKYSIDDTYCKEHEATCKNYG

KFYTWNQALIACPSGWHLPDNNEIEDFMESINPYTVYEGECLLKHTRFKKIEDPDLNKLL

YKDFGMDYFQMQFWTSTIANFNDDYANTSGYACHKTDLQNVRCVKD

>261379763

MFNFIKKQFIDVIQWPNPDDSLLMWRFPIADEEIQNGASLTVREAQMAMFVDEGVTADVF

GPGRYTLNTQTLPVLTNLKNWDKLLSLRLNRMFTFSIPNNSSHENGVRLNQLPCAMLSLA

RCNCVRSVCILTASAIRQNSLKKSVVWRRNTAA

>268610137

MKIADIIKYEGDNSTFVWKHPCEDFNTSSQLIVHESQEALFYMNGQALDLFGPGRHTLET

ENIPLLRRLINIPTDGKTPFHCEIYFINKTEQMAIRWGTDSKVQYVEPTYGFPLAIGANG

EMTLAVEDSRNLLINLVGTEALLDRQHLVNYFRAFLMTRVKTYLAQEMRNNAISIFEIDE

RLEEFSKGIHEKLKPDFAEYGVDLRKFFVTGIVKPDDSEQYEKFKELHFRQYADIAEAKL

KQQVGIIEQQTAAQRMVIQSQAMATKRQQEGYTYQQERGFDVAEQVAQNEAVGQFTNMGI

GLGTMAGVGGAVGGVVGTAINGAVSGIDSQPSTPPVVPIKCFCDNCGVELEAGTVFCDNC

GTKQEAPPLNNVIEQLLSAVPMTSMSGYTTRLNDTGCPSATSFSGRHSAKR

>269211798

MAAPASAAPLLAAGLRLDRPGASAPRHAIGKIPFEAGRGLSLGHDRPHFH

>269790595

MPTYEYECVSGGHRFERFQSFSEPAIEVCPECGGQVRRVLFAAPVIFKGPGFFRTESRKN

SDGSGESNKDKSESSNGSSESED

>270276968

MGLRRMTVCAVPPRLQLLCDTRIHSFLGGYMPTYHYRCKQCGYDFTEHQSFDDPAITVCP

KCAQETVIKVFSAVPISFKGSGFYRTDSGKH

>281403110

MDDYNSFSTIDHLVEFGMGIAIAQQMVNTMNHCIGNMQVPGAGNTLTSGKPTPQYHILAN

NAVAGAFSEEELNTLAKAKTLTSETMVWKPGMSGWMQAKNVPEIQKIILLNT

>288330009

MGLFNKLRNEFIDIIEWTDNTTDTMIWRFPRYQSEIKNGAQLNVRESQVAVLVNEGQFAD

IYQPGRHVLNTNNMPILSTIMGWKYGFNSPFKVDVYFVNTKQFLNVKWGTANPIMLRDPE

FGPIRMRAFGSYCFRVNADPRKFITNVAGTNGNFTTEGITQQLRNFVITKFTDHLGESKI

AALDLAGNLNEFSASLTEALKPDFEEYGLELTNFLVENISLPEAVEKALDKRTSMGVIGN

MGAYTQMQFADSLVEGAANGGGNVAGNAMGMGMGFAMANQMTNQMANQMAGQMAQPGAQQ

APQQPAVGAGAPGMPPPPPPQPMFHLSVNGQQQGPFGMPQLQQMAQNGQLTRDTYVWANG

MASWEFAKNVPALAALFGATPPPPPPGT

>288567398

MFNFIKKQFIDVIQWPNPDDSLLMWRFPIADEEIQNGASLTVREAQMAMFVDEGVTADVF

GPGRYTLNTQTLPLLTNLKNWDKLFESPFKSDVYFFNTKQQLARKWGTSQPVTVRDAEFG

AVQLRSFGMYAYRISDPAKFFKEVSGVAAEYSGVELETQLRNIAVTQLAAAFGSSGIPFL

DMAANQVLLSQKIGELLGAEFAKLGLTLENFTVESITLPAAIQEALDKKISMGVIGDLGR

YTQYQTAESIPLAAQNEGGLAGIGAGLGVGAGVGQAMAGAMAGMMQPAAQPAAQPAQNVQ

TVSEDPQAKLAKLKSLLDGGLISQEDYDKAKAEVLKQLIG

>288865819

MDHKTDETGSACPCKWVKCPRHGDCGSCREHHEKEKKFPPFCEKNPGKKKGGAGSSK

>B9NHG7_POPTR

MGSENCVATDICIGSEGCVAAKSRIGSENCIAADFCIGSENCVAAKSRIGSENCIAAQSR

IGSENCIAAQSRIGSEYCIAADFCIGSENCVAADFCIGSENCIHHHLSLSKYIYIFLWVF

TILLTFDKKKRKSRPVVVEREFRERCSPSPEASPPDYFAAELVSSFFVSPMASPMLEPLF

HSLRLETKSMRCDFLETLAAEISEARVPTRISVFSLTISGLGGCTVEIVSFLKLAGISSL

LLVEAASGYPPWSILLVLFWHYVESCCKGAIACLGLLRLPSGSILGSYGICFHSIFLFIV

AMDPDNLGEVLSPYAWASCCALCRCSASLLLVENFWMEFVLVAI

>Q8MIS5

MSDSKEPRAQPLGLLEEEELITSSMNFFPRDFGFRQTRGYKSLAGCLGHAPLVLPLLFFT

LFTGLLVAILVQVSKNPSSQRLDQSKQDEISQDLSQLKAAVERLCRPCPWEWTFFQGNCY

FISNSQRNWHDSITACQEVGAQLVVIKSAEEQNFLQLQSSRSNRFAWMGLSDLNQEDMWQ

WVDDSPLSTSFKQYWNRGEPNNIGEEDCVEFNGNGWNDDKCSAAKFWICKKSAASCSRDE

GQLLSSASASPIAHAA

>A1A5G9

MHCCSRHLAAFHWICLLIQCCCLVFASDLQLTSPIGENAPFAIYSPEVGGCLAVRDSVVG

LQWSCNDSSLDQQWLWVSRRRLFNLGSLQCLWVPSLSNGTSSTISVLPCDHENFHTIRRC

EAQLEQLSNALFPQTTNTTSLQEPPAKSAIQWLINGTNRSICSAVFWDIYTIQGNSNGRP

CAIPFKYDNQWFYSCTSTGREDGHLWCATTVDYGKDEKWGFCPVKSDDCEAFWDKDPLTQ

NCYQFNFQSALSWSEAWTSCRQQDAHLLSITEIHEQTYINGLLTGYTSTLWMGLNDLDTN

GGWQWSNGSPLKYLNWESDQPSNSMEENCAVIRTESLGAWQNRVCGNALPYACKKSPGSS

RRSLPIEPQEPDVAIDCDPGWQSFGTNCYRVNSEKKTWQEARKACVRVEANLVSIHSMLE

LEFVSKQIKQDVEELWIGLNDLKHQMNFEWSDGSSVTFTSWHPFEPNNFRDSMEDCVTIW

GPEGRWNDSPCNQTLPSICKKPGNFNQGGKVDTHGCQKGWSWHSPSCYWLSDESVTYSEA

TKACSDRQAMLATVHNRFDQAYVNSLLYGSEGSYYWTALQDINDTGTFRWLEGEEVIYTH

WNRDQPGYDKGGCVALATGRSLGLWEVKNCVEFRAKYICMQTLVTLTPLIPARPIPSLNG

SCSDGWNSAPNLRYCYKVYHSEKLQDKKTWISAQLACREIGAQLLSVSSVEEEHFVGHLL

NKIFGESDTDFHEQHWFWIGLNRRNPSGEQGWVWSDGQGYSYHNFDRSNHDDDDIRRCVV

LDLSSLQWVALECESQMDWICKLPKGTEVKEPEVSKGSSEWITNENAQYKFFEHHSTWLQ

AQRICSWFSAELVSIHNQAELDFLGQSLQKFSRGQEQHWWIGLHTYENDGRFRWSDRSVL

SFVNWASGRPRPISREKKCVYMTASRGDWGDQKCDTALPYICKRTNNTLPVPTTPPIPTN

TGGGCPRSWLPFLNKCFGVRADSKAETKTWQQARDSCKVLGGELATINNYLEQAFITSIL

PNITFDLWIGLHNARKQFQWVEGQVLNYVNWAPREPSGYGTSGSSEKPINCAVVWHGSPP

PFTGRWDDRNCLEEKHGYICQISKVASLNPPAEPFPPAPGSILRYMNNTYVILQKPMTWS

DARLLCETRNATLSSIPDPYQQAYLTLAVTGLKTPVWIGLSNEEGGRSYSWLTNDSLSYT

NWRDGEPQLMSGCVYMDFQGTWSTANCESKLQGAVCKFNTESRKMHKWSEAGNCPRSLSD

SSWIPFRDNCYSFHMEIRVSQREAAKKCHKVGGEVLSILDETENVFVWEHLQAYESQSRG

AWLGMSFNAKGGGLVWNDNSPVNYSNWGQQDAGPSLLSPNSCYWVQGSNGVWSLGSCTNN

TMGVICKLSRAEETAHSKSVFPEHTTAVAVVILSTTALCVLLVVVIYLYRRRKHAAELGA

FESARYSRTSTGPGGSAEKNILVSDMEMNEQQD

>A2TBB3

MLLEIKYIFWICSAFSLTNAFRAVKLEKSSGVKGSLSGRVNLPCFFSTIPTLPPSYNITN

EFLRIKWTKIVQSRDGKDPKETTVLVAQSGSIKIGQHYRGRVSVPSHPEDIGDASLTIVK

LRASDAGAYRCEVLFGIEDTQDTVSLDVSGVVFHYRASTDKYSLDFEAAQKACIDSGAQI

ATPGQLKAAYEDGFEQCDAGWLSDQTVRYPIRSPRAGCHGDKMGKEGIRTYGRRPALEKY

DVYCFVDELEGDLFHTTVKNKFTFEEAKQECEKKNAALATVGDMYAAWRKGFDQCDYGWL

ADGSVRYPVSVARPQCGGGLLGVRTKYRYSNQTYFPHSQERYDAYCIQGKRNITEAVSIR

LILPTEASTTSVIKTVEKLPEKVTIKPVYSDGRRPATESISHLKAETGVTPDTKQKETVV

PTDPSALSFQEEGTLASKLESTQDVIEPYAYQTEKIGVESEITKSPTGYPVEQQYSIPDN

SIKLTEVLYQSLQPPKGSMEAKSEEIIPTVIIHHEIVSDKDIITSLPEIRTGPEETFESS

VVTVSSLKQDSDASSTQPELDVFTVYKTSTSMQPAVTSGFDSSEEGSQPTVSSESIVTKE

KHVVSTVHEDEPTDKVQESTTLPVTEDVIIESEDSFAAVEEESVLQSSRLSPSDSPTQVT

SPVLDVTEATSTSKEPKPTDTVDKVPETHISHKETSVEQITLKPVSISTQKPHLIDVEPE

EETSKETIVIDESVSRLKVTTESDMPGKQEEDVDSEYLTSGTSGKEPTQKPECEDTSAEA

ETNVHETDLSQNRSSINVIVVHLRDNETGPVESIIHVLDQGGLQLPEHNESSEENDYPII

IDVIHIVPPDIDNDSDCDNTTDVSTPPPLQFINGKQEITTAPGDSNAEELRQDQIESATP

SENVSLTQFSEVTERVISQTDVPSITTSLVVDIGESISHTKLQEPEFSGDTEFYAETSTS

QKTDLPPTPSMLSLNRSEHLSTLESLPDTDSKVLSTIESVTEESRIALEHVTDAPSSQLI

CEIATHTSEQTLSPKTEKTPKEDFIPTEEYTKETEEPSIVTEASIVYAEPEGSGDLDSQS

IVRTDAPSSQLIPEIGTHTSEQTLLPKTEKTPKEDFFPTEEYTKETEESFQDTDSKVLST

IKSVTEESRIALEHVTDAPSYHLISEIATHTSEQTLLPKTEKTLKEDFIPTEEYTKETEE

PSIVTEDSIVYAEPEGSGDLDSQSIVSVTASVQRPEEIKSSPTKVIIKADTPMPTSFPGI

DLSSEEVKSVTSVFDQVTKIITTTKEEYEGSIFTVDPTKSFGLFEEGEGSATDGVILDQQ

PAVSSSRYATETVPTVTDSAQVKPTDFITFSSEAISSHEIIKTVTTSDISSHSSASQVVT

VDSLLMPEGSGDMDDANKYVSSTVSQEKHGLEILSTDSAKTADTLLKYAITEETVILKED

YVPTETSDKQSTTAVTEEIEKFTITETEEESVVSGDPESPSEKLQVSTVQSPLTDGDSKQ

EVIIYSTHFPPESNHTAVEHLGSSPFPVLVSTASSSIDLAISDKTSALKTSTILPFTGEG

SADGDLEIILTSKPSLDSIKPDPEAVTVFTIFTDDKTIETGITHSLTPSNISSDLPVVDQ

GSGDLINSETEIEITTSKDFATVSTIELTESADTTDVLGGTVVKQLAAEPETFGTETPPS

LEHITTLEPQKTIFTDSLFTDTGSGDTDDMVTFSPTLTSLSQRKHETETIRAVTSVELST

VGLEVETEADLKETLRVESEITNDKEKPQLQTTSITSQPTSEQATQVPLKYILSTEYPIS

EGSGDEHQFAESTTSKTTEHETTSIITAQSEDEHSTKIVFEGSGDESIGTGTTIYVTKTK

SQIEDISSVFPTEESTVSLEADTVKIDLHVENHTKEAYTAESINTDTVSETGTQACCSTP

VHPTSGHISLHTVQETASAHSPVIIESSGDTEDISTATETYATKTDIEDISTAFPLEKTS

LSPARETSFDLKIDTKDAATVENETSFSTASQEISKVVTQATVIVPGDSGTKVSVITDSP

TYESSGDEEILIGTTTHTATPKLDSVIISTSPPTKEHTESAEAETDFELADIKEKETGEL

DTVISTDQEVSKFTTQASSTLYIYSPTEDISMFPAKTLVSTLPPDESSGDGVDSFPVTKT

YAIKIHSEDISTIIPTEQPKMSLSIETSEASVILETTTKEIKVDSEIDISTDAHQLSVPG

TQASATISVPPATTVFIEDSSGDDADIFTDTYPIETKVDSKADRTGFPVEKTTVGFTAEV

ADSKTGVSGEAEIESETTVILPSHSTSEITPSLPEKEIISTDESSGDDGFLLTMSKAYAT

ESEPVSITIVPTEGLQVSLEVETSESGLKSESSTEDDEAAESEKGIKTDMLETSTIVTQA

IGISSHVAGEPITSSSVRKHSTTDSVFLDQGSGDQSQGDLLGVATTETSEVKTEGATEAI

GTHSAATDMQELSKTATQTPSATLRPEISQYVTDTPHKEIISTKSEILEAGSGTDEDMFQ

DVVSTPVIKTESKSPESRYVSIVQPTTEYTEHTALPSTIKDDDINEPREYIFSTKSPDSD

VGSGAEVGTSEVKTSTEETRALKPQFILTTNAPEKATDGITAYLSSSFMGEVSEIPLIEQ

GSGDESQQATVIDEETKTESKFEDQTETIMSPVSEYPKILEFKNVTTDRDSEIPTKESET

PEPKIRVSTSPKEILPEVKSTQDVPISHTLSMTELTIGLEDEVTSKPSVLIFEESPVSTI

WEETEKNETISEVISASHSATEFILDDKTSKQISEELELSTVEKDIKLEFKSTPATILKT

TESLSTKKTFLYEEGSGDLISMTIKEDKPIYTVVTLPFVEDDEAPVSTSVTKDLTELETD

ISQETSSAITEHYLRSTQQPEISTETPHVSSKDILEILKITETSTEPIKEESTYTTTTPT

EQPEIIDTAFELFSGQGSGDITDDPDTPSASIKIDYDSFSTTQEKHIEPLLRTDKPQVHE

TKSSKPDADMYSKVTSHMYVDSSLLETTDTDFLFIETQTMSLIRDSVLAKEESESDQELI

GVRPSTVVATIRPDIEDIVTSSPIIPESPNEGGPEVTSEQEVTPKEYVLPISASAFPSEV

HVPTEKELEASVTSRPSMLVDKEYQETGSSDIVILSSVTESTTDGDKVSDIDQTAETLLT

TTALTYTKTTIESEVEGDKFSPRLEDSSKVYRTSDSVPFETVTSQALLLREQSEIDSITT

KATFESEYVSKEEGDGEIRTSLVEEHITSDDSKSEHVTEETKLIDEPIASTPTYIHKDKV

YVSKVTEETTVVTEELSPKYPVTVILVNGASDYTGKIMPSTLPSAGSGTDHVVSEQEVSA

DIAATYKPEDVHIVDTTDSPLDHSEKTDILDESVSLTSDHKEDTTSSQQYVQEDIDHIHK

HTLPTNAPSIDSDNVLNQLYGQLTDTDSPPSLKSEESFTPFPQYSDEKVETQSALVVQKE

FSTVSYNIAEPVDENEIDPALLEEEDEAMAAVTSEPEIEVTDGIEIHISTSNNVEGAELQ

FASQDPCKVNPCQGGGTCYARGGTSFVCTCMPGFSGDQCEIDIDECQSNPCRNGAACVDG

IDSFKCICLPSYTGSLCEQDTEVCDYGWHKFQGHCYKYFAHRRTWDAAERECRVQGGHLT

SITSNEEQTFVNRLGHDYQWIGLNDKMFENDFRWTDGSTMQYENWRPNQPDSFFSAGEDC

VVIIWHENGQWNDVPCNYHLTYTCKKGTVACGQPPFVENAKTFGKAKPRYEINSMVRYHC

KEGFVQRHLPTIRCRGDGRWDLPKVSCLNPSNFQRTYSKKYYYKFTPPEMRTSMNSPKHH

HSWSRTWQDSPR

>A3RLP6

MGWIRGRRPRHNLEMSEFHNYKLGLAKSDFSTRCQKQRCPVIKSKCRENASPLFFCCFIA

VAMGIRFIIMVTIWSAVFLNSLFNQEVQIPLTESYCGPCPKNWICYKNNCYQFFNESKNW

YESQASCMSQNASLLKVYSKEDQDLLKLVKSYHWMGLVHIPTNGSWQWEDGSILSPNLLT

IIEMQKGDCALYASSFKGYIENCSIPNTYICMQRTV

>A4GHD1

MGWIRGRRPRHSLAMSEFRNYNLELAKGDFSTRWQKQRCPVIKSKCRENSLFNQEVQVPL

IESYCGPCPKNWICHRNNCYQFFNENKNWYESQASCMSQNASLLKVYSKAEQDFLKLVKS

YHWMGLVYIPTNGSWQWEDGTILSPNLLTIIEMQKGDCAVYASSFKGYTENCSTPNTYIC

MQKTV

>A4JYN2

MFLHILLSAICLCVLSSSSVLSSATDDSSALKVAIPASSPVSAVLGGSLTLPCLVSLPRT

PSLGRHAVLTQPRVKWSFLSSDRETEILVARGERVKVSEAYKGRASLLNYATSSADLTLR

LEGLIHNDTGFYRCEVQHGLEDSHDQAQVKVKGVVFLYRHTSSRYAFTFDEARDACKDIG

AEIASPEQLLAAYHSGYEQCDAGWLSDRSVRYPIQMPREGCFGDMDGLPGVRNYGTMEND

ELYDVYCYVENIHGEVFHGSTPQRFSLSEAKAYCEQQDAQLATTGQLYAAWNDGLNHCSP

GWLADGSVRYPIVTPRERCGGSEPGVKTVYRFSNQTGFPEPNTRHDAYCFKANSNSQTAP

PADYMATSPEETEQHIVTLAEPQEEYSVHQVTQRSENEAQGAVESFSIYSTQTASETEEH

NHTSSPQEDIIIGSRKDLTTPTSTEGNTETIEVTWQNVGSIPEIYIDPKRNNFMLEGEPE

IKASAVDGVKSSNHRHYQPMPDTNLEPGEPIDFIPPTVAHHGTTEEPSSPPKEINGSKHY

QPMPDTNLDDEDHMTTQSTTLQYDSSNNTTQDASVQSTYHPDGATLETNAGNITEAPESD

DNQGLFTHTTQPTSTSTSSREGWLESSGEDSTTEKAIYLDLTTKEAVNLDSTTEEPIHLD

STTEEAIHLDSTTEEAIHLDSTTEEAIHLDSTTEEAMFSTHNSEIYRASTAETLTESYSS

THLNNISPTDDVTDHFTLSTTLRETELSESSGDHDEVLPVILFTTPMPHLLDSLTTRAPV

QLEISSPQQIEGVLSDSTTASALRVVEEEVEKVEQEELGEQPETKITTSTELNHSTVTLS

IDGSDEEESSGEPSENWTSVTENPYSNTATITPSNLTDLQEHNETNSDDEDNQAITVEPT

TDLEVTFLPHGTQTSIWQSVVTSPGELNADVEFSGEAALTTDDMRALEESESTNAPISEQ

TEETLKPSSTTDHNDADDDDNDNDDDELITKAITGNEEDENDVKTTHESFIIPVRPTQRV

LIRTSYISDACLENPCKNGGTCVDSGGDQRCLCLPTYGGDFCETDLEHCEPGWEKFQGFC

YKHFAKRQKWEVAEQHCRMCGGHLVSVMSPEEQLFINDKYREYQWTGLNDKTIEGDFRWS

DGNPLLYQNWYRGQPDSYFLSGEDCVVMVWYDDGRWSDIPCNYQLSYTCKKGISAFCGQP

PLVLHAKMFGRRQLKYRANSQVRYYCESGFIQRQNPIITCQSNGLWEEPKIMCSPAGPTY

SNGDLVTWRTGQNKEIVIEDSTTKTPEYLDIKWNF

>A5HUL1

MEEVREYPHVLGTEWSRREGPSRGACVTFQLTMAAVFTVLLITAVAFAVQAFQPHPQPCA

QCPFDWIGFRGKCYYFSEDESNWTSSQNNCSALGASLAVFDSAEDLSFTMRHKGSSPHWV

GLSREGKEHPWEWVNRSPLSHLFQVQGDGLCAYLGDAGLSSSHCSARRNWVCTKPALQKP

RKNFCIST

>A5JPG5

MKILVAVFLVLVVVGTAAAGCPDGWTQFLDLCYIYQSAKASWASAQSSCQALGGILAEPD

TACENEVLIHMCKENGDAGSFGPWLGGQKVGGAWQWSSSGAAFDYLRWGPHEPNNSGGNE

DCLHYNWLSWNDLRCHYQASYLCQRAAE

>A5WUG2

FTEIYSIDGNGFGQTCKFPFLYEKKWYADCTTVDEPDQRLWCATKTDYSLYEQWGYCPTR

KYWTKHPLTNVYYQLNDRSTLTWYQARKSCQQQGAELLSISEPHEQSFIAGSLWIGLNKL

DVSSGWQWSNGQPLRYLKWLSGFPSSQPGYNCGVLKNGYNSEWSNDACSEKRGYICQRGH

SVPTVPPEVTTGFCQSPWIPHSGNCYLLHRTKQTWLEARDICLREGGDLLSILSTEEQSF

AITQLGYSKTDQLWIGFNDRKTQMLFEWSDQSSVPFASWEVGEPTHSAQHAEDCVLMRGE

EGKWADDVCEKKYGFICKRKTSTKASNNDTVVTNPGCKKGWIRYGYYCYMAGSETKTFEE

AKQTCEKAESRLVDVSSRVENAFLVNLVGARPEKYFWIGLSNQKDVHTFEWTNTKQVPFT

HFNSGMPGRKQGCVAMTTGIVAGLWDVLSCSNKEKYICKQRADALVTTAAPPTTPSLDCP

TEWTSIGTRDLCVKHFNVPSLQMKTWDQALDYCRELGGDLLSIHHESDIPWKQGGGYPSW

IGYRMYDPSVGYVWSDGSSSSYQSWASDEPNNLNNMENCVEMRVSLWDDDGMWNDVNCKD

KKDWYCQIHKGKTPVEVNITEPVYNVTEDGWIEFRGSQYYGSEYSAMSMHEARAFCKRNH

GDLVVINDEEERLFLWHKSKELYNDFLIGLTVDLDGSFQWMDGSPVVFQAWEANQPAFKN

SEERCAKMTISQGLWETVNCGDEYNYFCKRSEAPPVNATVAPTQPPKGGCAPEWTQFEGK

CYNVRGEMKKWSEAREYCREHGGDLTAIMSKFQQ

>A6NAB9

MEYQSSVENLDEDGYTQLDFSSRNITRRSIVSEKGLCAASSHWRLIAVTLGILCSVMLVV

TVVLSTSGVFSSSCSPNWITHEDSCYLFSTLLDSWDGSKRQCFQLGSNLLKIDSSKELEF

IARQVSSQPDHSFWIGLSRRRTEEPWLWEDGSTLLSNLFQIRSTVTKKDSSHNCAWIHVS

DIYDQLCSAHSYSICEKKLSV

>A7MC71

THSSSEASLKVIICRPRFMMFLLRSLLLLSIVFSMVGADEERLRCERGWSRSGSRCFRFF

SRSVDWVTAERNCQSLGGNLASVHDQVENDFLLSLGPDCEAINCCFNGQQCYYGNAVTVQ

CIRDGQFVVVVARDVTVPRLSLDTVSLLGGNDPPCSPVASNPYFAVYQFPVSACGTNVIE

ERGHVVYENRMVSSYEVAMGPLGSITRDSQFEVLFQCRYSNTAVEALVVEVNAIRAPPPV

AALGPLRVELRLANGQCVTKGCAEGDEAYASYYNEADYPVTKVLREPVYYVDVHILERTDP

NIVLMLGNCWATSTPNPLSVPRWDLLVNGCPNQDDRYLTTLVPVTASSGVHFPNHHKRFI

VKMFTFVDPQSLSPVQQTVFIHCNTAVCYPSATQSCEQSCARKRRDVSSMPFSNENTVSS

GQVTLLP

>A7RES7

WITQEGSSFCYLFSKTRTTWQDSKLQCNAINADLVTIDSQQEQNFVRTVARGSFVWLGFT

DLRTEGRWIWESS

>A7RIS3

MQLINILAPLIGFFLSDVNHFNGSCFAISADKATWENAKMTCESHMMQLASIHSLNENRL

ISNMMENENSTAWIGLRNYTGAKGLAWIDGTPVDFLNWASSEPNWNGNCVRVRARDDDWL

DRQCSSTYNFVCKRPCNSMNSTAEC

>A7RP19

CLEGWEIYQANHSCFKFVSEKRPWKDASRYCQNIGGNLTSIHSAKENDFVSNLGEGNAYW

IGLNDLKNEKAFVWSDGTSVGFTQWAFKRPSDNGKDKDCTYLLKQSKTWIDFSCANSYPF

VCRYPLEPA

>A7S3Q6

LKCGSGWTGAMGDFDAFCYKVVFDKSNWDDARANCQSAGGDLFSVTNAYEQRFLENFTNI

ESWLGYRDQKAAGTWRWSDGSKYMTSSYTNWDERSPNNGGVTCAIVTKKGRWHDEQCQAK

YSYICKKPSESGQGNQGV

>A7SYQ8

CPDGWRKFQDSCYWSDARHMSWYSARSTCLSLGGDLVKISSSQENSFVARIKRYQLAWIG

LKKQGSWFLFCLWSMISFRFAQWAPGEPSNLNMHELCCAIGYKGVAKWDDGGCGGHLGFI

CERKVNR

>A7SYR9

CPAGYSRFGSNCFKYHTTPVTWDNAVLRCANENATLVSVRNQDEEKFMRTRLLISHMVCF

IGLSDRLQEGHFPWLDGLHFKPGVCAPVNAHGGAAKNCVAWTTSKPISCWSDKSCSEKHP

YMCRMPVEGSVRANRRLR

>A7TC24

MHSYFLFLLDVCGENWTFFNGYCYLTSNTCLSWKQSESACAVLGSGLVSVHNYEENVFIQ

QQHNGEKSWVGLSDVTSEGRFVWADGSPTNFTNWAPNQPNDYKNQDCVHTLGVNYGYKWN

DVQCSSCHNYTCKRGV

>A8C7X1

GDKGPQGGAGQPGQKGEKGDTGGPGVQGLRGEKGFPGPPGIQGFKGQPGSRGNPGSKGSR

GSGGRAGPPGANGEPGTAGLPGRDGQAGPQGPQGPSGDRGQVGPAGAQGPRGPVGPIGAP

GPPGLPGLTARSAAAAAVPLVPFSMQSEAPAPTVSAPGCPQEWVGFRDKCYHFSKELHNF

DDAKKSCDAQTASMVIINDNDEQKWLQKQTSGKGYFWMGLTDREEENAWRWLDGTEPAFS

QWKPGQPDNWSHGHERGEDCAGLIHEGLWNDFFCEDLISYICEKAIEYPKNSRIIAPAVE

GEETEEAWLSQRRYDEHH

>A8CVN6

MTLDDLKSNSMKDQPDEKSNGDKAEGPRSLSTLRWRPAALILGLLCLGLLVTVILLIIQL

SQVSDLLKQQKVKLTHQEDILEGQALAQRQAEKSSQESQRELKEMIETLAHKLDEKSKKL

MELQQQNLNLQKALEKVANFSGPCPQDWLWHEENCYKFSSGPFSWEKSRENCLSLDAQLL

KINSTDDLEFIQQTIAHSSFPFWMGLSLRKPNNSWLWEDGTPLMPHLFRLQGAASQMYPS

GTCAYIHRGIVFAENCILNAFSICQKRANLLRAQ

>A8E4S9

MMVMLRSLLLFFFLFCMGIAAERRCPRQWRRSGSRCFRLFSTSVNWATAEKNCQRLGGNL

ASVLNDVENDFLLSLIPNSKRFFIGGYNVDEQNWFWSDGSPFGYTNWCSGEPNNMNTEHC

LEINWTANRCWNNLPCSVELGYICAKNLNDCS

>A8MWX1

HKCSPCDTNWRYYGDSCYGFFRHNLTWEESKQYCTDMNATLLKIDSKEEKLFLTSFRILT

IVFIWVGLSRQKSNEVWKWEDGSVISDSLFSQSNFQNGKGNMNCAYFHNGKMHPTFCENK

HYLMCERKAGMTKVDQLP

>A8WAA6

MLLEIKYIFWICSAFSLTNAFRAVKLEKSSGVKGSLSGRVNLPCFFSTIPTLPPSYNITN

EFLRIKWTKIVQSRDGKDPKETTVLVAQSGSIKIGQHYRGRVSVPSHPEDIGDASLTIVK

LRASDAGAYRCEVLFGIEDTQDTVSLDVSGVVFHYRASTDKYSLDFEAAQKACIDSGAQI

ATPGQLKAAYEDGFEQCDAGWLSDQTVRYPIRSPRAGCHGDKMGKEGIRTYGRRPALEKY

DVYCFVDELEDTEVCDYGWHKFQGHCYKYFAHRRTWDAAERECRVQGGHLTSITSNEEQT

FVNRLGHDYQWIGLNDKMFENDFRWTDGSTMQYENWRPNQPDSFFSAGEDCVVIIWHENG

QWNDVPCNYHLTYTCKKGTVACGQPPFVENAKTFGKAKPRYEINSMVRYHCKEGFVQRHL

PTIRCRGDGRWDLPKVSCLNPSNFQRTYSKKYYYKFTPPEMRTSMNSPKHHHSWSRTWQD

SPR

>A8WGQ6

MSAYREEIHNNLIDGPDDDYSGAELRYSVFQNPGKHRKRFCQEPFKVATACLTAFCLILL

LVLVVTGLHAGKTSSSGQGSSSSAAAAHQQTGSINVTALTTELQTAKREKSELEKDKSEL

EKKKRELEKEKSELEKKKSELEKRKSELEKEKSELQKELLQLKDKVTKCEVTPAPRTTPA

PTTSPCPQNWKHFNGSCYFISVTTRSWTDSQTYCKRYGGHLAIILTAEEQTFIWDLLPRG

YWNAFWFGISDEKVEDDWHWVDGTKLVGGFWEDGEPNNHIDEDCGYMIKTDVLTRVAIKS

WYDAPCHMSLPWICEKPASS

>A9JRA9

MAMLRNLLLLSVMFSLGNAGIGTCQCPYGWSKFGVKCYRFISQSVTWATAEKNCQSLGAN

LASVHSKAENDFLLSLIPSSSTRCWIGGHDGENEGRWLWTDGSVIDYNNWCATEPNNQNV

ENCMEMQWTVNRCWNDQACSTSMGYMCAANL

>P81114

DCPSDWSSYEGHCYRVFNEPQNWADAEKFCTQQHKGSHLVSFQSSEEADFVVQMTRPILN

ANLVWIGLSNLWNQCNSQWSDGTXLDYKXWREQFECLVSRTTNNEWLSMDCSSTHSFVCE

FQA

>Q90WJ8

MVSFKLPAFLCVAVLSSMALVSHGAVLGLCEGACPEGWVEHKNRCYLHVAEKKTWLDAEL

NCLHHGGNLASEHSEDEHQFLKDLHKGSDDPFWIGLSAVHEGRSWLWSDGTSASAEGDFS

MWNPGEPNDAGGKEDCVHDNYGGQKHWNDIKCDLLFPSICVLRMVE

>P07306

MTKEYQDLQHLDNEESDHHQLRKGPPPPQPLLQRLCSGPRLLLLSLGLSLLLLVVVCVIG

SQNSQLQEELRGLRETFSNFTASTEAQVKGLSTQGGNVGRKMKSLESQLEKQQKDLSEDH

SSLLLHVKQFVSDLRSLSCQMAALQGNGSERTCCPVNWVEHERSCYWFSRSGKAWADADN

YCRLEDAHLVVVTSWEEQKFVQHHIGPVNTWMGLHDQNGPWKWVDGTDYETGFKNWRPEQ

PDDWYGHGLGGGEDCAHFTDDGRWNDDVCQRPYRWVCETELDKASQEPPLL

>P07307

MAKDFQDIQQLSSEENDHPFHQGEGPGTRRLNPRRGNPFLKGPPPAQPLAQRLCSMVCFS

LLALSFNILLLVVICVTGSQSEGHGGAQLQAELRSLKEAFSNFSSSTLTEVQAISTHGGS

VGDKITSLGAKLEKQQQDLKADHDALLFHLKHFPVDLRFVACQMELLHSNGSQRTCCPVN

WVEHQGSCYWFSHSGKAWAEAEKYCQLENAHLVVINSWEEQKFIVQHTNPFNTWIGLTDS

DGSWKWVDGTDYRHNYKNWAVTQPDNWHGHELGGSEDCVEVQPDGRWNDDFCLQVYRWVC

EKRRNATGEVA

>P24721

MEKDCQDIQQLDSEENDHQLSGDDEHGSHVQDPRIENPHWKGQPLSRPFPQRLCSTFRLS

LLALAFNILLLVVICVVSSQSIQLQEEFRTLKETFSNFSSSTLMEFGALDTLGGSTNAIL

TSWLAQLEEKQQQLKADHSTLLFHLKHFPMDLRTLTCQLAYFQSNGTECCPVNWVEFGGS

CYWFSRDGLTWAEADQYCQLENAHLLVINSREEQDFVVKHRSQFHIWIGLTDRDGSWKWV

DGTDYRSNYRNWAFTQPDNWQGHEQGGGEDCAEILSDGHWNDNFCQQVNRWVCEKRRNIT

H

>B0FFI7

MEKLAVLLLLSAAIALGDTNLTQLLGLEPLLKTEVEQTPPVGAQVAAVQQGTKEMSCPSD

WHPYGSRCFRFVSIPRSWSDSEQNCLALGGNLASVNNLLEYQFMQALTKNTYGHLPDTWI

GGFDAVKEGLWMWSDGSRFDFINWNIGEPNNAGEGEDCLQMNAASEKLWFDVPCEWKFTS

LCSRRM

>B0R0P1

MDLVYVNSDFVCSATASSDKSSHDKGQKTFNKDHHRWTKVLLIALTVCLLFALGAVCTLA

VFMARTETHFNVSVSDQEHNATDYKEQLDVLHIQHQEMLQKLNRLNESSGCALCAVHWTH

SGGKCYYFSTVKMNWTQSRDHCVTKGGHLVIITSKAEQDFLASKISVTHWIGLNDMHTEG

RWVWVDNQPLNKSVEFWMKRVNGNNEPDNWTKNHPGGEDCACLGHSLGATEFWNDDLCTA

TKRFVCEAAAAIN

>B0R0P2

MGSVYENSVFAFSTGHFSNYRESKREVNTAKWTKVLLIVFAVSLVFALGGLCAVRIQYVS

VSAQLSAQETNGTIMSRQLDKLTANFTTVRDHLHINELLMKELTANYSRVKEQLSISEET

VRKLSRFNQSSGCAICAIHWTHSGEKCYYFSTVKMNWTQSRDHCVTKGGHLVIITSQAEQ

EFLTSNVKETHWIGLNDLDTEGRWLWVDNQPLSQTEEFWMKRENGVSEPDNWTKQHVDGE

DCASLGHPDGETDFWTDAYCFEEKRFVCEAAAAV

>B0ZBM3

MHAEEIYTSLQWDIPTSEASQKCQSPSKCSGAWCVVTMISCVVCMGLLATSIFLGIKFFQ

VSSLVLEQQERLIQQDTALVNLTQWQRKYTLEYCQALLQRSLHSGSDCSPCPHNWIQNGK

SCYYVFERWEMWNISKKSCLKEGASLFQIDSKEEMEFISSIGKLKGGNKYWVGVFQDGIS

GSWFWEDGSSPLSDLLPAERQRSAGQICGYLKDSTLISDKCDSWKYFICEKKAFGSCI

>B1AR34

MTKDYQDFQHLDNDNDHHQLRRGPPPTPRLLQRLCSGSRLLLLSSSLSILLLVVVCVITS

QNSQLREDLLALRQNFSNLTVSTEDQVKALSTQGSSVGRKMKLVESKLEKQQKDLTEGSE

RTCCPINWVEYEGSCYWFSSSVRPWTEADKYCQLENAHLVVVTSRDEQNFLQRHMGPLNT

WIGLTDQNGPWKWVDGTDYETGFQNWRPEQPDNWYGHGLGGGEDCAHFTTDGRWNDDVCR

RPYRWVCETKLDKAN

>B2BSM3

MSDSKEPRVQQLGLLEEDPTTSGIRLFPRDFQFQQIHGHKSSTGCLGHGPLVLQLLSFTL

LAGVLVAILVQVSKVPSSLSQEQSEQDTIYQNLTQLKAAVGELSEKSKLQEIYQELTQLK

AAVGELPEKSKLQEIYQELTQLKAAVGELPEKSKLQEIYQELTQLKAAVGELPEKSKLQE

IYQELTQLKAAVGELPEKSKLQEIYQELTQLKAAVGELPEKSELQEIYQELTQLKAALGK

LPDQSKQQQIYQELTDLKTAFERLCRHCPKDWTFFQGNCYFMSNSQRNWHNSVTACREVR

AQLVVIKSAEEQNFLQLQTSRSNRFSWMGLSDLNQEGTWQWVDGSPLSPSFQRYWNSGEP

NNSGNEDCAEFSGSGWNDNRCDIDNYWICKKPAVCFRDE

>B2RTX0

MHNLYSITGYPDPPGTMEEEEEDDDYENSTPPYKDLPPKPGTMEEEEEDDDYENSTPPYK

DLPPKPGTMEEEEEDDDYENSTPPYKDLPPKPGSSAPPRPPRAAKETEKPPLPCKPRNMT

GLDLAAVTCPPPQLAVNLEPSPLQPSLAATPVPWLNQRSGGPGCCQKRWMVYLCLLVVTS

LFLGCLGLTVTLIKYQELMEELRMLSFQQMTWRTNMTGMAGLAGLKHDIARVRADTNQSL

VELWGLLDCRRITCPEGWLPFEGKCYYFSPSTKSWDEARMFCQENYSHLVIINSFAEHNF

VAKAHGSPRVYWLGLNDRAQEGDWRWLDGSPVTLSFWEPEEPNNIHDEDCATMNKGGTWN

DLSCYKTTYWICERKCSC

>B3FVQ2

MAEICDPKEPEEKTWTGPVLVERDLGLLRRLRNSPGCLTWPLLLLLLFVSLGFFMLLVTT

LVQVSRIHQSLQRERETSRRPTAQEKIQSSLDKFLQQMTWMNATLAGLCHPCPWHWEFFQ

GRCYLFSQTQSDWKSSLSACKDIGAQLVIINSTAEQKFLKSWYVRYNKATWIGLSDDTNE

GSWQWVDNSPLQLSFWKEGEPNNHGDEDCAELHNDGWNDSKCTVENAWICEKPSSPCPML

>B3KV04

MVPEEEPQDREKGLWWFQLKVWSMAVVSILLLSVCFTVSSVVPHNFMYGKTVKRLSKLRE

YQQYHPSLTCVMEGKDIEDWSCCPTPWTSFQSSCYFISTGMQSWTKSQKNCSVMGADLVV

INTREEQDFIIQNLKRNSSYFLGLSDPGGRRHWQWVDQTPYNENVTFWHSGEPNNLDERC

AITNFRSSEEWGWNDIHCHVPQKSICKMKKIYI

>B3V3S6

MCDPKEPGDPEEEMFGGQRTTKKYTGLLRSSRSLPGCLTQAHLPLLLLLISLGFFMLLVT

TLVQVSRIRQSLQKETWDHQENPSQVDVSQKRTRSDLDEILQQLTWMNATLAGLCRPCPW

KWELFQESCYFFSQTQNTWKESISACQNLRAQLVIINSTEEQKFLKSWNTRNNQRTWIGL

SDHHNEGSWKWVDNTPLQLSFWKEGEPNNHGDEDCVELYSDGWNDNRCSTENFWICKKPS

SPCPGP

>B4DNS7

MRSQMEKKLKVFSFFTHQKKKLEGQISARQQAEEASQESENELKEMIETLARKLNEKSKE

QMELHHQNLNLQETLKRVANCSAPCPQDWIWHGENCYLFSSGSFNWEKSQEKCLSLDAKL

LKINSTADLDFIQQAISYSSFPFWMGLSRRNPSYPWLWEDGSPLMPHLFRVRGAVSQTYP

SGTCAYIQRGAVYAENCILAAFSICQKKANLRAQ

>B4XWC3

MNTSTHLTVTHHTDWSTRTLRRTSMRSTCPARYELFNGNCYRFSTDQKPYNEAQAICQEE

VVIWQPAKNNETHDFLANHVRDTTKGNTWIGLSDVVTEGQWVWDDGTLLVGDGIWGTGEP

NGGTVENCVHIYPLKDYRWNDSTCPTSRYYICEISG

>B5BUZ2

MDEDGYTQLDFGTRNIHKRPVKSEKGSPAPSSRWRSIAVALGILCLLTVVVAAVLGALGV

FSGPCLPNWIMHAKSCYLFSFSENSWYGSRRHCSQLGAHLLKIDNAKEFEFIESQTSSHR

VNSFWIGLSRNQSEGPWFWEDGSAFTPNSFQVRNTAPQESLPHNCVWIHGSEVYNQMCIA

SSFTICEKEL

>B5U215

MDTARVYLSLKPSKTAAGAQCVSPPSLPPDACRCPRSHRLALKLSCAGLILLVLALVGMS

ILVRVLVQKPSVEPCRVLIQENLSKTDSPAKLKCPKDWLSHRDKCFHVSQASNTWKESLA

DCDGKGATLLLIQSQEELRFLRYLIKGKGSSFWIGLSYTLPDRNWKWINGSTLNPDVLSI

FGDTKQNSCASVSQDKVLSESCSSDNLWICQKELKCECMCNGS

>B5U216

MDTAVVYADLHLARTGEPKREPPPSLSPDTCQCPRWHRLALKLGCACLILLVLSVIGLGV

LVLTLLQKPLIQNSPADVQENRTKTTDSPAKLKCPKDWHSHQDKCFHVSQTSITWKGSLA

DCGGKGATLLLVQDQEELRFLRNLTKRISSSFWIGLSYTLSDEKWKWINGSTLNSDALNI

TGDTEKDSCASVSQDKVLSESCDSDNIWICQKELKRESTCNDS

>B5X2M5

MEKVENYTSLHEFTEDISSRGNKPILNNQNTQGLKRGSECLRGQTALFVLIGLLASICAN

IALSVLLFSRPLPSVPLEAAALTLKLNSVRGRYVRLCDDYSKLGQSCSKTVRKCRECPED

WIHVDEKCYSFSNDKMDWPSSRDSCTSLGSHLTILHSKEQHDALEKEARRIGGFDYHFWI

GLSDIEKEGDWRWVDNTTLTNKYWNEHSSEPDNHQSGGSHGEDCATLDSHSLTWFDVPCD

NIYKRICQMDAIRLD

>B5X696

MFLLWRTWVPRESTLKRTHLSRCLRKQTPDRVFSLEPSEELGVVQSMSLEEEQVQTESSG

ICPTGWFKHGHRCFTYIHTERAWAESEQYCVFQGANLASIHSTEDNHFIHEMIHRQTHDF

PWAWIGGQDATQERLWLWSDGSRFYYRDWSNVQPDNAGGNENCLHINYGEEKRWNDNVCR

MKLPSVCSMKL

>B5X834

MENLIRNLPIESEEMGGKEMQGLQTRSSSCPPGWHSYRTRCYHYVPIMAKWPEAEHYCLL

LGGNLASVHSLSQYNFLQSVIQSSANGAQRTWIGANDAIKEGLWLWSDGSRFSYQNWGQG

QPDNSNHGAGNDNTNGHEHCMEMNYGDDFRQNDAPCWMQLPFMCSRKL

>B5X9V5

MAMLTILLLLSAAIVLGEAFDLRAAKAGVVEEQQEAEAAESDRPCPGGWTKYKSRCFMFV

NNAMTWPQAENHCLSFQANLASIKDCVENYNLQQLVLRNTGQHQPTWIGGFNSVQNKLWF

WSDGSKFDYQGWGQGEPNNYGGNEHCLQMNAGGDKTWNDLNCEVKLPMVCALRTC

>B5XAH4

MADYVNKQVIELNKVIGENENRATRRVKTETHLSDGRTRLYKLAAVCFGMMCILQVILNI

SLKLAFCTGRVVEERDMAVPTRTVLGCAEGWSLSGSSCYFLSTERKIWEESRQDCLERGA

DLVVVNSRNEQKFLTELNRNINGVWIGLTDRETEGTWKWVDGTPLTTRYWGQNQPDNGAV

FVVYIGEEDCVEINYGNLDPVNKWNDIACNLQFNWICERVI

>B5XD87

MSVPLCFLCLLSLAVGALHCTPVSDQEGELSEPRYTDCPQGWSNYNNRCFRYVASQLDWA

DAESYCVSLGANLASVNNKGEFSFVKNLIKSFDPAESYTWIGLSDLHKEERWMWSDGSKV

VYTIWSSGEPSGSRTENCVQTNYLNDKLWNDAVCSYKSAFVCATRPGL

>B6RAZ0

MKTFCCTLLLLLGCALHRGAEGSRCRGGFHKHGGSCYWFSNIRGTFAEARSICRFLGSDL

ASITSAAEDVFIRGYATQRGKAKVYYLGGADLGLESSWIWTRNKPFTFTNWGSGQPGNSK

NNEHCLALQSSDGYRWHDYNCDFIANFICKM

>B6RAZ1

MILYIFLAVCFISNTAEGFCQDRWIQFGDSCYGLIEDDEGWAQAKYLCESRDTHLVTIES

AAENDFVKSYARKQAVVTDVWIGASDIWAEGIFRWVGSDTNVTFTDWNAGEPNGISDDEN

CAELENSVDYLWNDDECTKQQHFMCETRFLSVVVG

>B6RAZ2

MRLEVLSFLLLTFASLAQARGRCPLGFFRHGKSCYWISTTVASFAEAKTYCQYMQSHLAR

ITSRYEDAFLRGQVRRRGKGTDYWLGATDLNVEGVWLWEGNKAMTYTNWGGDNPNNYKGV

ENCLGLRKENNYQWNDFQCHHRLNFICEKNL

>B6RB57

MKYIVLIEIYIVYLSRAQVFGDCLDEWTSYAGSCYALVDAPETWAGAAEICERFGGYLVE

ITSSDENDFVKRFVKARGNANVWTGGSDLLSAGHWRWLYSRDIITYFEWASGEPEMVSTD

RCIQLWSEKAFSWDDTPCDDENAFVCETGTDGPDIVG

>B7K5X3

MFSLAKLIDFRGGVKLSLLIAGGIFSVLMNQSAQATTFKNSQYFLTTLDTWTGAQAQAQA

IGGNLVTVNDLEEHQFLLDTFGTAEPLWIGFTDQDEEGIFKWISGESVTFTNWVLAEPNN

AGGGEDYTVMNVHLPGGWNDASGTESLRGIIEVQSVPEPLTILGVGTAIGFGTAFKRKLT

KNTKKK

>B7TXW4

MSPLNPRPYSMSSSAHLQDAPPLLSGTLTQNEGQTSLRQSSSCGPSAASASESLSGYTES

RIPHSKVRQGKGLRSIFPESRVKRYCCYGGVITVVAIAIVVPLSVTLSVKQMEQTSINNT

FAASINNTSAASINNTSAACPSNWTEYGNKCFYFSEYTSNWTFSKDFCAAQGAELARFDT

EEELNFLKRYKGSSGYWIGLHRESSEHPWKWTDNTQYNNLVPIRGDGQCGFLSDQLNISS

SRVYVERPWICSKPKKYISQSQ

>B7ZN67

MDTARVYFGLKPPRTPGAWHESPPSLPPVRVLIQKPSIEKCYVLIQENLNKTTDCSAKLE

CPQDWLSHRDKCFHVSHVSNTWEEGLVDCDGKGATLMLIQDQEELRFLLDSIKEKYNSFW

IGLRYTLPDMNWKWINGSTLNSDVLKITDDTENDSCAAISGDKVTFESCNSDNRWICQKE

LYHETLSNYVGYGH

>B7ZS04

MRWNMLLLLILTHASILLTSALEENSENSKTLKVTIGSHPARPVLTGTLTIPCHIRYQSP

SEVISAGRQAVLATPRIKWSFISQGKEVEILVARGRKVKISEGYRTRALMPHYAESVNDA

TLILNSLITNDSGIYRCYVQHGIEDDYDMLEVKVKGVVFLYREGTARYAYTFSMAQEACN

RIKATIATPEQLLAAYHSGYEQCDAGWISDQTVRYPIQIPRDGCYGDMDGFPGVRNYGVL

DPDDMYDVYCYVEELNGEVFLGSTPNKFTLQEARDYCKRLGTNIATTGQLYAAWNQGFDL

CSPGWLSDGSVRYPIVTPRERCGGNASGVKTIFMFRNQTGFPDSMAKYDVYCFRDKSRIT

PQNLDDGQPAPVEKVQNIITLTESFKKLMFPEGIAENEAQGSVDTIPLNKTKMADSQQIL

EELRETITKEQESTLQESTPTIKSSTSNSYLSYSPVVTEQEREDDKEAETAKEDASFTQK

TPETKVAFIPQNKSVFTLQETTSSMLEESVAVPTQQVPGDSTTEVVLVNVDAESDYFYDN

ITNLPDEFGSEESGTLNQTFNTTTQSPKRSLETDQLMNNHGEKENEEVYNISVTTNKQSE

ASSNPVYPTQAAYVTSLAGSGIENKDGGNLLLHSTGDIDDTSSGGFPNLVTPGEFSISSS

GAGAQVFEASGHGDNLFTEDLNISPKPFVSLLKELVTSEFPVSTKPPMYSVDTRLDRSKD

AHTNLFTTQVDSSLETSGQSSSQATLEEASGDGSGSLWAESSGQTNGTQDASTNIVEHSG

NPTSHPEIKQITQSVAHSTKFFGKNPVDKKDENLETATVQISQLKSSSVISTERQENINE

NSLSSTALPNSNSREVMTVSPVYYVPLRHTLSPSPTDGSEDSTTHILEKTANPNVSMSAT

SPPPAMPTERAILGASINLSDVCYPNPCGNGGTCIDEEDGDFLCLCLPGYTGKICEINVE

TCLGDWDAFQGFCYKHFHARRSWEEAENFCREAGGHLTSIMTPEEQAFLSNKYNDYQWTG

LNDRTIEGDFQWSDGNPLLFENWAHGQPDSYFLSGENCVVMVGHNEGKWSDVPCNYHLPF

VCKMGLVSCGPPPDVPNATMYGRPKSTYQINSVIGYGCKDGFLQRNSPIIRCQADGFWEE

PQLSCIPNASFQ

>B8A5M2

MWISAAFLLFALAVNGVKSDVPNISRRCPSGWEKFGSQCFKFFSEYKTWAEAEKHCVDLG

GNLASIQSDITHNFLIAYLKRQEKGITRTWIGAHDATQADIWFWSDGSKFEYSAWHSGEP

NNGGNAERCAEMGFGDEQRWNDARCETRLNFICYRMTRINM

>B8JM29

EEKSWSDSRQFCRNHGGDLVIIKSEEKQRFISSVVKDDSWIGLSDTQTEGTMKWVDYSPL

NQGFWASGEPNNAGVNEDCVELTPTRPVLNNWNDEPCSRGKKGICEK

>B8JM30

MEMDTIYENVEDVKDEGKAQQNRRSCLMLATVILGTICVILLVFVILQHTRAGSNRLGLF

FISNNTMSWSESRQFCRDRGADLVIINTEEKQRFISPFVEDFLWIGLTDEEIEGNMKWVD

NSPLKQGFWVDGEPNNLNGENCVIIVPVENFLKNWNDVPCTFTFKALCEK

>B8PS70

MSAKKASQPMLNTTGSLQEGEMGKMFHGKCLRIVSPESPAKLYCCYGVIMVLSVAVVALS

VALSVKMTPQISTINTYAACPRNWIGVGNKCFYFSEYASNWTFSQTFCKAQEAELARFDT

EEELNFLSRYKGSFDYWIGLHRESSEHPWKWTDNTQYNYSLSIRGVERYAYLNDIGISSA

RVYADKRWSCSKLNSYSLQCKTPFSPMSRE

>B8PS71

MKTEVTTTDFLEEGETVEMRSSRDEDSMRLMKTEDTTTDFLKEGETGEMLHGNCLRIFSP

ESPAKLYCCYAVILVLTVAVVALSVALSVKKTPQISNINAYAACPRNWIGVGNKCFYFSE

YASNWTFSQDFCKAQESQLARFDNQEELNFVKRYKGSSNYWIGLHRESSEHPWKWTDNTE

YNNLVSIRGDELLGFLSDSGISSSRNYIKRNWICSKPNNYTSQCKSR

>B8YM31

MEESLYSEFSKFPRRRRRCCRRGMQLALVGLMIAALWAGLLTLLLLWHWDTVRNLKQLEE

AAALNVSQVSKDLERHKGDQMAQKSQAAQMLQDMARIQAEQKRMVSQESELFRNLDGLRA

DLSNLKSYSLNERHRALHSLGRLQEEVEKLWLELHESNGSVCNTCPEEWVHFQRKCYYFG

ETAKKWIQAKYACSSLQGRLVSIHSQEEQDFLTKHAHIRGSWIGLRDLDIEGEFIWMDEK

PLDYSNWQPGEPNDAGQGEHCVMMQASGQWNDAFCGSYLDWVCERLATC

>B9EJA8

MRLPLLLVFASVIPGAVLLLDTRQFLIYNEDHKRCVDAVSPSAVQTAACNQDAESQKFRW

VSESQIMSVAFKLCLGVPSKTDWVAITLYACDSKSEFQKWECKNDTLLGIKGEDLFFNYG

NRQEKNIMLYKGSGLWSRWKIYGTTDNLCSRGYEAMYTLLGNANGATCAFPFKFENKWYA

DCTSAGRSDGWLWCGTTTDYDTDKLFGYCPLKFEGSESLWNKDPLTSVSYQINSKSALTW

HQARRSCQQQNAELLSITEIHEQTYLTGLTSSLTSGLWIGLNSLSFNSGWQWSDRSPFRY

LNWLPGSPSAEPGKSCVSLNPGKNAKWENLECVQKLGYICKKGNTTLNSFVIPSESDVPT

HCPSQWWPYAGHCYKIHRDEKKIQRDALTTCRKEGGDLASIHTIEEFDFIISQLGYEPND

ELWIGLNDIKIQMYFEWSDGTPVTFTKWLRGEPSHENNRQEDCVVMKGKDGYWADRGCEW

PLGYICKMKSRSQGPEIVEVEKGCRKGWKKHHFYCYMIGHTLSTFAEANQTCNNENAYLT

TIEDRYEQAFLTSFVGLRPEKYFWTGLSDIQTKGTFQWTIEEEVRFTHWNSDMPGRKPGC

VAMRTGIAGGLWDVLKCDEKAKFVCKHWAEGVTHPPKPTTTPEPKCPEDWGASSRTSLCF

KLYAKGKHEKKTWFESRDFCRALGGDLASINNKEEQQTIWRLITASGSYHKLFWLGLTYG

SPSEGFTWSDGSPVSYENWAYGEPNNYQNVEYCGELKGDPTMSWNDINCEHLNNWICQIQ

KGQTPKPEPTPAPQDNPPVTEDGWVIYKDYQYYFSKEKETMDNARAFCKRNFGDLVSIQS

ESEKKFLWKYVNRNDAQSAYFIGLLISLDKKFAWMDGSKVDYVSWATGEPNFANEDENCV

TMYSNSGFWNDINCGYPNAFICQRHNSSINATTVMPTMPSVPSGCKEGWNFYSNKCFKIF

GFMEEERKNWQEARKACIGFGGNLVSIQNEKEQAFLTYHMKDSTFSAWTGLNDVNSEHTF

LWTDGRGVHYTNWGKGYPGGRRSSLSYEDADCVVIIGGASNEAGKWMDDTCDSKRGYICQ

TRSDPSLTNPPATIQTDGFVKYGKSSYSLMRQKFQWHEAETYCKLHNSLIASILDPYSNA

FAWLQMETSNERVWIALNSNLTDNQYTWTDKWRVRYTNWAADEPKLKSACVYLDLDGYWK

TAHCNESFYFLCKRSDEIPATEPPQLPGRCPESDHTAWIPFHGHCYYIESSYTRNWGQAS

LECLRMGSSLVSIESAAESSFLSYRVEPLKSKTNFWIGLFRNVEGTWLWINNSPVSFVNW

NTGDPSGERNDCVALHASSGFWSNIHCSSYKGYICKRPKIIDAKPTHELLTTKADTRKMD

PSKPSSNVAGVVIIVILLILTGAGLAAYFFYKKRHVHLPQEGAFENTLYFNSQSSPGTSD

MKDLVGNIEQNEHSVI

>B9EMN9

MAMLTISLLLCAAVALNGATVLGLFQGFGFEQALHLGAKGAEEGVVSAETRNQCPTGWFQ

FGSRCFMFVETARSWPLAERHCVSLGANLASVHSSADDQFLQEVVGSKTGGFSTTWIGGF

DAVQDRLWFWSDGSEFDYQNWAKGEPNNSGGREPCIVINWGDEYRWNDIKCGNSFPSVCS

KRICEIQKN

>B9EPI6

MDNDENIYANASSSRASPREGAIYFQWWKRPSRAAAVCLGLLCVLLLAGIISLSVYQRNQ

STSYNNLTKARDQLQKERDQLQKERDQLQKEKENLNKKIKGRRCPEGWREFEFSCYYITN

ENKAWSQSREECHEFGADLVIINSKEEQVFINGLNQTKNHVWIGLTDSVIEGTWKWVDGT

PLTTANWGKGQPNSNKGIDQDCGAIWDHSGWWNDEKCLSKHKGICEK

>Q7LZK5

DPGCLPDWSSYKGHCYKVFKKVGTWEDAEKFCVENSGHLASIDSKEEADFVTKLASQTLT

KFVYDAWIGLRDESKTQQCSPQWTDGSSVVYENVDEPTKCFGLDVHTEYRTWTDLPCGEK

NPFICKSRLPH

>Q56EB0

MGRFIFVSFGLLVVFLSLSGTAADCPPDWSSYEGSCYRVFEQKMNWEDAEKFCTQQQTGG

HLVSFQSSEEADFVVSLTSPILRDSFVWTGLSDVWKECSFEWSDGSDLSYKDNYQFVFSE

YECVASKTKNNKWRIIPCTKLEYFVCEFQA

>P22029

DCPSGWSSYEGNCYKFFQQKMNWADAERFCSEQAKGGHLVSIKIYSKEKDFVGDLVTKNI

QSSDLYAWIGLRVENKEKQCSSEWSDGSSVSYENVVERTVKKCFALEKDLGFVLWINLYC

AQKNPFVCKSPPP

>P22030

DCPPDWSSYEGHCYRFFKEWMHWDDAEEFCTEQQTGAHLVSFQSKEEADFVRSLTSEMLK

GDVVWIGLSDVWNKCRFEWTDGMEFDYDDYYLIAEYECVASKPTNNKWWIIPCTRFKNFV

CEFQA

>C0HAX0

MKVLIISVLLCVGLTLREAAGQQQAVAVEQLSIVPVLEEAKETGPLKKLCDAPMETIVEL

VENKVELALPEEGEAEADALALVQAPAIRFLHCPDGWYLHQSRCFLFVNSYLSWHNAEEH

CNTLHANLASVQNPRQYRFLQQLTTMANRHSAWIGGFYLQDRWLWIDRVGFYYENWYTHL

DVDRNACIHLRTSVGWSNAHCGTGLPFICVKMSC

>C0IP18

MAEMYEHKEPDDSEEETFGGQRLAERHPRPLHSLRSLSECLTWGPLLLLLLLFVSLGFFT

LQLTTLVQVSRIQCLQRDSGDRENNSLDKWLDTRFRSLTEVAEKQMQSNLEKILQRLTRM

NATLAGLCHPCPQNWEFFDGSCYFFSWTQSDWRSAVSACLLIGAHLVIIESTEEEKFLNF

WYPRNNKPTWIGLSDHHSEGSWRWVDDSPVQLSFWKKGEPNNHGDEDCVELHNDGWNDGR

CVTENPWICEKPSVPCPVL

>C0JP79

MEYQSPLENVDEDGYTQLDFSSHNLTRRSVVSEKGPCAASPYWRPITVTLGILCLLMLVI

AVVLGTMAIWRSSSGNNMLKNDSFPSRNKNNPSQPTQSSLEEGEDTTKALPTTGVLSSSC

PPNWITHENSCYLFSTSLDSWNRSKRQCSQLGSYLLKIDSSKELEFISRHVSSQPDHSFW

IGLSHSQTEGPWLWEDGSMLLSNLFQIRSTVTQKESSHNCVWIHVSDFYDQLCSVPSYSI

CEKKLSI

>C0KTQ4

MRTYAILPLCIVLLSAAGCVSAYCPWGWRQLDSFCYYASSTSMTWHQAQRFCRRLGGDLV

KITNARENEFVLAVARKSAPTRKQVWIGLMWTANDFYWSDYSVPVYKAWAPNEPNGKSRE

PCSNMWTGYTSVLPIRASGYWNDMPCTVSSHVPFGLVCKKLA

>C0KXG5

MNSNFNISLFLLAAIACLSEGCCPERFMQVGRRCYYYNSGKVDWHDANRACQRLGAHLVS

IHNAEDSREVYALWKSLVDADRRDDRDAAYWIGLNDDRIEGFFEWSDGTRLGYSLWQRGE

PNNTNGGEDCVAPRNDGRNDFDRQRWNDGTCTDRKSYFCRRSL

>C1BWF3

MGVKTVSILACVLVALLGTGASRYSSHYRVCPNGYSTAKTGSHRCFQLVTKQMGWQDALD

YCKKDGGTLASVHGYQEYQFLLSMVKKTQDISAYSWVGLNDLDQEGSFVWTDRSDTEWFW

STSARAQQWGEEDCVALNSYTGAEGFDDITCSSSCTFICMRAAVV

>C1BX08

MAEMIYSNVTFTPHPNRDAGLRSNVNEAMEHEEDVTYSEVRTGAGQQANAQKESNPSSGN

PSAKVGSKVVTTEKESARVTFVSLVCLCVILLVLSITLGVLYAEEARSRAAVAQMMENLT

AIRCASGWEFYNGSCYHFSEDKLTWEQSQYACIREGGHLVIIESQQEQNFIRLKVGNMND

QNNYWIGMTDMKVEGVWVWMDNTPLNDSIKYWDLNNGTDVSAYPEPNDWKPGEDCAQMGR

RCSGQISCWLRQCM

>C1BX78

MMSQEIVYSNETITLHQNQDKEEGKSDLGPPQVGSKGGSQRVTMVTLACLWILLFCLATA

WVVLFVSNEVKDNISDQREVLSSELQFCEKNLTVVNNHLTTLRAKRCASGWEFYNGSCYH

FSEDKLTWEQSQYACIRDGGHLVIIESQQEQDFIRMKVGNTEVTNSYWIGMTDIKVEGVW

VWMDNTPLNNSIKYWDLNNGTDISAFPEPNDWKPGEDCAQMGQRCSGQISCWIDTPCNVP

FKRICESH

>C1BX79

MMRVTEASSTDQDCEQGYVTKQDIRESSGPAQHSGTRVYILVGVSFGLLCVLQVTLNVSL

NLVYSYKNVTDLRQTTYNTLLCKKDICKACPQDWKRFGCSCYYISTVLKSWTDSRQDCLD

RGGDLVIINSREEQAFIKSFNYRAWIGLSGREVKRSWRWVDGTPLTTSYWEDGEPNNAGG

EEDSVLVRRIDVPLAAWNDVSCDHENSFICEGTVGEINQ

>C1BXK2

MVMLNILILLSSIVALGDFSALGNANPVDEQEDWGCGRSCPSGWTRHNSHCYIYDNTART

WSQAEQNCVYLGGNLVSIHNDGENEAVKDVVFKATNSYPLTWIGGSDLYKTKVWFWSDGT

KFGYSNWASGEPNNGGGTEHCGQINYPGAGYWNDLSCEYPLPSVCKLRKQ

>C1BZ08

MPDEIYEDPQGPEDDECRKRKSMDIDQVSANVLNPRRKHGVSGQNSVNPYRVTTVCLGLL

CVLLLIGILGLFCYQINLLTSYNKLMEERDQLLRITEMLNNKIRVKFKGGSCPEGWNKFE

TSCYYNTTLKKTWEDSRQDCIDRGADLVIINSREEQSFVNQLFGPGNYTWIGLTDSDTEG

TWIWVDGSPLTTKFWDKGQPNNLNSQDCGEVIHSSYDPGMWNGDGCHKTNSWICEI

>C1BZJ2

MSGEVIYSNVIFIQNQKQAAGAKGCVFGKALELEENVTYSEITIGAGKQGNVQTESTCPD

IPAPPVESKIFIPEGRFRRVVLVTLVCLCVILLVLTITLGLLYALNKIHLKAEQVRIKEY

LRIQKEQSSELHHIERNLSAVNDLLAKAQDEKKWLSSELENNTRNITALNNLLKSVQARQ

CASGWEFYNGSCYHFSEDKLTWEQSQYACIRDGGHLVIIESHQEQDFIRMKVGNTDITNS

YWIGMTDIKVEGVWVWMDNTTLSNNTMYWDQNNGTQVSAFPEPNNWESSEDCAHMGVRCS

GQISCWFDYGCNIPSKSICESRSIK

>C1J0C7

ACNKKLGYICRKGNSTDNTPPPGKDQPNFCPAAWVPYAGNCYYLQRTKKMWNDALAACHR

EGANLASIHNIEEHSFIISQSGYLPTDELWIGLNDQKTQNLFEWSDRTHVTFTTWLVGEP

SHFINRLEDCVLIKGKDGKWADHACEMERGYICKKKSSSKPEGAPEVVSLGCQAGWVRYG

SYCYMSAIESKTFNEAKQICEQTGANLVDVASRYENAFLISLVGLRPEKYFWIGLSNTES

PESFRWSNSDKVKFTHFNVGMPEKHRGCVAMLTGTSAGLWDVLDCNSKQKYICKKMAEGV

TTTQIPPTTQPLSCPVGWTKKDPRNCIQIYSRPKEQKKTWFEARDFCKAIGGDLASFHSQ

KQINNLQYGMGETAWIGFNLLNINSGFVWTDGTPSDFENWSFGEPNNHNNQELCTESSFY

YGRKWNDRDCEAYNDWICQIAIGTTPKSPPTTVTQVEYNKTSDGWIQYNDSQYFINEESL

PMEDARSFCKKNNGDLVVITGQTERKFVWKQISRGSENQYYIGMIVNLDKSFNWVDGSPV

VYTSWDQNEPNFANNDENCVTIYKSMGFWNDINCGVPLPSICKRSSNFINSTLPLPTVRP

GGCSPEWTIFQGSCYKLFQNKMTWHDARNLCISHGGNLVSILTHEEQAFLTTLMLDSVDD

IWIGFNDVNWEMRFLWTDGKGVSYTNWAKGVPSSMPDGPSRMYEFGSENYDCVVMLRSPE

KMTGMWKVQMCGDQQGFICKRKTDTQLIRPVTTAAPQHFFTLGNDSYKVQKEKMSWDEAR

RQCKASDADLASVRNSISQAYTILTVSNFKEPLWIGLNSNLTSGQYRWVDNWLLSYSRWA

TGEPKNNLACVYIDTDGRWKTATCNNTYYSLCKQSTDIAPTDPPQMPGNCPESKKRKTWI

PFRGHCYAFMSNSENWAHATVECIRIGGSLVSIEDPKESHFIQRNVELMQDGVRSFWIGM

HRSYMGDWMWIDNAVVDYTNWRTQVTNNAGHCVEIQSSSGLWNAVNCNSYKPYICKTEKV

VPPTKKPLVPLAVVDAGPHSSAGIVVGVVLVIIAVAGLATFLFLKRIPRPAIGECTFDNR

LYINSDLTQSPTTIDTKGLVANIEQNEHA

>Q91ZX1

MSDSKEMGKRQLRPLDEELLTSSHTRHSIKGFGFQTNSGFSSFTGCLVHSQVPLALQVLF

LAVCSVLLVVILVKVYKIPSSQEENNQMNVYQELTQLKAGVDRLCRSCPWDWTHFQGSCY

FFSVAQKSWNDSATACHNVGAQLVVIKSDEEQNFLQQTSKKRGYTWMGLIDMSKESTWYW

VDGSPLTLSFMKYWSKGEPNNLGEEDCAEFRDDGWNDTKCTNKKFWICKKLSTSCPSK

>Q91ZW9

MRMHTRLQFLKRVSNVAYSHGQEQAKKEKVYKEMTQLKSQINRLCRPCPWDWTVFQGNCY

FFSKFQQNWNDSVNACRKLDAQLVVIKSDDEQSFLQQTSKEKGYAWMGLSDLKHEGRWHW

VDGSHLLFSFMKYWNKGEPNNEWEEDCAEFRGDGWNDAPCTIKKYWICKKSAMSCTEK

>Q91ZW8

MSDSMESKTQQVVIPEDEECLMSGTRYSDISSRLQTKFGIKSLAEYTKQSRNPLVLQLLS

FLFLAGLLLIILILVSKVPSSEVQNKIYQELMQLKAEVHDGLCQPCARDWTFFNGSCYFF

SKSQRNWHNSTTACQELGAQLVIIETDEEQTFLQQTSKARGPTWMGLSDMHNEATWHWVD

GSPLSPSFTRYWNRGEPNNVGDEDCAEFSGDGWNDLSCDKLLFWICKKVSTSSCTTK

>Q91ZW7

MRAPQMGSLGFLDKGHIPLVLQLLFLILFTGLLVAIIIQVSKMPSSEEIQWEHTKQEKMY

KDLSQLKSEVDRLCRLCPWDWTFFNGNCYFFSKSQRDWHDSMTACKEMGAQLVIIKSHEE

QSFLQQTSKKNSYTWMGLSDLNKEGEWYWLDGSPLSDSFEKYWKKGQPNNVGGQDCVEFR

DNGWNDAKCEQRKFWICKKIATTCLSKW

>C3KHH6

MEMQDITKEVERSKEDEGASEPMLEVKTEEEGEPDHYSKLQIPSEDIYSEAVYGGTPVKT

RSGKQTEGNTRLYRAGCLFLTIICLVLLLVIIILSVKLQTGSTVCHERAETTAADSRGPP

FAPSCSHEECMALFPDTLPQRTECQQCNAGWLPYGRSCFYFSAFRLSWANSQKNCTTSGG

ALAVVTSRKVQDFLTGNQKTNYWIGLRQKGATWTWVNNDVLQESYWADSTPDGDCGILSG

RKPPEKNWMKASCEAVTYYICEVQL

>C3KIA1

MSVTLSSMSGVFKEDSVNLMASDNRSKYGWRSQISVWWIGAAAVCLGFLLLFVILGVVAH

NSRTIGHQDSKCLTLIYSLTIDRNTLRDERDQLKINSSNLTKEMEVLQSQYNAMAVSRDN

LQEEIHRLNLSRTDKPCYQGWKSFRDKCYYFSPTGVTKTWESSRKDCKERRADLVIITTT

EELDFVSRTYEITWIGLSDKEQENKWKWVDGTNLLSDEFWQKGEPNNNNNEDCAEVSRTA

KKFNDVPCARTFSWVCED

>C3KK79

MKTLLVLSVLLCASFAAPAEDKVKAPEAAVEALKEQIVAVHELEEKMKKDEEVSVPEDSV

AVPRNAPEEAAAPESRYNFCPNGWFSHGSRCFLFVSTPMTWYSAEEHCNNLGGHLASATN

PREYSFLQQMTQTAGQSIAWMGGFSLQGRWMWIDREGFYYTNWYSQSSSSSYPCIHLRSS

YGWGNTQCGSAYRFICSKNPFSC

>C3UZX8

MTSVFPLALLLCLSSGLLTAYGHTPCPHGWRHCGSRCFSYNAGPKNWIDGEFFCISTGGN

LASIHSAEEHEFIRHYIREVTGTDKNAWIGGHDGVKEGTFLWTDGSKFDFTSFATGEPND

FGAGEDCVVMNFNGNNWNDGSCDNEAAFICSKNSV

>C3UZX9

MTSVFPFALLLCLSSGLLVAYGEQACPPGWTQFGSRCFAFYMQRKTWFDAETFCQTAGGH

LASIYSAEENTFLQDFIFQVTGAHTTSWIGGTDTVKVGTWMWSEGSRLKYKSWAAGEPNL

PGVEDCLLMNWVGTTGWFDWVCANQASFICSKNSV

>C3UZY1

GWLFFSVSANHHLSKDGIRLSVCFVALFVHRTVGSIRSCPPGWTQFGSRCFSFNFQGKSW

TDAENFCKSDGGNLASVHSEEEQLFLRDFIKKVTGEAKWAWIGGFDSVQEGVWMWSDGSI

FDYKSWAPGQPDNADGIEHCLHMNFQDKWNDTPCSRLLPFLCSKNL

>C3UZY2

MASGFPFALLLCLSIGLLAPSCHGRPSCPEGLERCYPSPGAPPSPHGLFTLTATGSTQSL

SSSSSSPRTPEHFPPPSAWPSTVLQPTGHCPPGWTQFGSRCFSFNIQRKSWTDAENFCKA

AGGNLASVHSEEEHEFLRTFIKQVSGLNRRTWIGGSDSVQEGVWQWSDGSPFNYRRWHVG

LPDNLFLVEHCLHMNFLGMTWNDDWCAKIFPFLCSKKL

>C3UZY4

MASVFPFALLLCLSIGLLAPSCHGQTSCPGPCPPGWTQFGSRCFSFNFQGKNWIDAESFC

KAAGGNLASVHSEEEHVFLKAYINQVTGANGRAWIGGFDSVKKGVWMWSDGSTFDYKNWD

VGQPDNFDGEENCLEMNYQGRNWNDAPCARTTVIIFPFLCSKNL

>C3UZY8

MASGFPFALLLCLSIGLLAPSCYGQTSDTDSEIVPPGSGEATDPSSSSSDLRQGSCPPGW

THFGSRCFSFYFQRKTWVDAEKVCKAAGGNLASVHSAEEYDFLKTYIHQVTGENKVTWIG

GFDSVQESEWMWSDGSTFDYTSWDPNQPDNEGGVEHCLEMNYHDNWNDYPCDKTRHFLCA

KKL

>C3UZY9

MASGFPFALLLCLSIGLLAPSCYGQTCPGHCPPSWTQFRSRCFSFNIQGKSWTDAEDFCQ

ATGGNLASVHSAEEYEFLKIYIHQVAGEIEHTWMGGFDSVQEGMWMWSDGSTFDYTSWAP

GTPDNSGGVEHCLENYKGKKQQHMKHLVF

>C3XPL0

GTCPSGYSKFQRSCFKSFPNPKTHILAERDCKAVGGFLAVPKDRETNTFLARQGNKIVRR

GKFYIGLSNRNRGKQWFYADGTRLGRYKNWRPGEPNNYRGKNEDCVELWWNGWWNDVPCS

YKYRYHCEVKLPRGHCKFLSLF

>C3XQT6

GPCRNGWTEHDNHCYKLMTIKASWATANANCKLLGADLVSIQKQPEHKFLKGIITNAPKG

KLGLVWIGAYLDGVDWKWTDGSPYSYARWAPGEPNYRNKSLGEICVGVYSKVGKIGAWND

VLCKEECPYVCKTPQRR

>C3XTW4

KCENGWSQHGGHCYLVGLQLSTWDEARWDCKTRGGTLTSIASDLEESFVSETILQSAHAF

WIGGHDRRTEANYMWVDGSKF

>C3XXH1

MCPSASCPEGYIMWIGVCYKVFNTEKTFSDAAATCGEDGGLLAMPRDAKTNAFLVSLSGK

RAFWIGLHDRDEEGSFEWVDGSALGAYTSWSPGQPDSLRGDEDCVYYPAQKDTWNDAKCD

WTYRFICQAFPACCKAL

>C3XYK3

MSWHEAEALCNMYGGHIASISSSEENTFVYHFISPFERAWIGLNDMYKEGTFVWSDGTDE

TFTNWAPNQPDNANIGEHCGEFYNNAAPGQWNDLPCNQDRPFMCKLHM

>C3XYK4

MGVFLVVVFAVSLSGCTGGPLGKGFHQNRAVTSCPPYWIPWEGACYRFFDHALSWRQAER

MCALFASNVVSIHSPEENNFVYGLTAHYVRTWIGLNDINADGEFTWTDGTDENYLKWAPG

QPDEAYNGEDCVEFWDKGAEGEWNDLDCGALRPFTCKIHLE

>C3Y8H0

CPKPGYSNFNRACYKQFAVSKTYDDARQTCTSDGGKLAMPKDSDTNAFIAGLGNGVHWIG

LNDIDTEGQWVFEDGQTLESTDYWGIGEPNNVNNEEDCAAIGYSGSAWIDLPCSQTHPFI

CQLDHGMQYIALQILS

>C3YHS1

MKNWKALGSRLPLAKETPRGHHAEVEVYNARLSTKQQEVEHVPAGDINAPPTLPPRNLPP

TKPEAENPGDKRSITTLGSWPLKQDNDDGRKNLSPTRKCHSLETGGKEKEKGDESNGDLD

RKYEDVDPAGESIFAWSYPPTKQDDVGAFGRNLPPPLPPRKSGNIPDTCGNTTSVSSSEL

QHAASTELAPGHGTKMRRSSTTEQRTDKVWKKYEDVELDSSGVDVVLGSGLKRAVPDLPP

PRNRDIEEHSHGRRRKMTARSTEFSRGKVLQRYENADLQSSGVNVVLGSGLKRAVPDLPP

PRNRDIEEHNVPKEDKEDKDEEVAGLVHGVRDSVPSPYHLDMGDHRLVWSEHSDQSYCDT

YFSQDHTYSINPNKDPTDNSTETEVYNSSQEEKCEDLEDKNVKESSSGLRKQEMDVNNEE

TGRRYEDVDPHGESIFALPSGLKSNEHSPRHLPKKDVHDSRPSSKKQHVEDVPAPLPDKK

PSLRHHAMGGKEKEQLDGSGEEPNRKYEDTDPHGYGIFASSPETKIQCGEDTDGRDTPPP

MPARNAHHLTIKDKEKDQKDEENRKYEDTDPHGNSIFASGPGSKEQCTEDTDGPNAPPPL

PGRKYENVGPAGESIPGLGPGLNRAAPGQHPTARPDPRQESRSDTSRTSGDTSRDGERDA

PGFCDRVRDCAKALWTKGKSSSLFWLLLGCGVVVTAAAVIAAVLGPRLMAVRTESISNTQ

FGAHLKHPDIDDCTRNPCQHGRCVNKDGDYKCTCSPGWTGQNCQQDKDECTRKPCQHGRC

VNKDGGYRCSCYSGWTGQNCQRGQNCPSGWSEYNNHCYKLMTDKISWDKANTKCEQQGAK

LASVQNEDENNFVADVIRNAKGFLRYHVWIGLKREPDGQFRWADGSLLSYTNWAPGKPDN

DGFLAFGKGEGCGVMYSKLSTSNRDVHPIGCRVCPALPMVTWSSISREIPLPFWSGLSAG

DVSGPGFSWVQGCRQTTMDRREFLQAARYGDVEKVRRGLEEGVDVNIKDGVSTTERRGEA

RSRLILNTITFASWRTLLCVTSNLIFSHSNPETSRLRKGTLLDPLMPKNPTRAVIQVLKP

FLREDLLKGFKCRSVLGGYRRVPIT

>C3YJF1

MQGDQQQSQTGDTGTTPMQQPQTDWRSIADAAASIPNALYVPRADRTYPGGTSGHRALCS

FIRSHHSCIIAGFAVLLSLVAVGLAPLTFINIEEISQLSTTVNDLNRVRDDMRRDLDKER

NRTAAMEQRLNEMSKTPAPLCQGGWSNYNNHCYKLFKDKVSWSTANERCKELGANLASVT

SADENNFITRFIANAPRGTWIPGVWFGLNRLDGKWKWADGSALSYTNWAKGEPGNNMPWT

TVHCGNVYSKSDDALAS

>C3YJP6

METTSSLITESQPTDSSGTCPVGYQRFQRTCYRFSTDQKPYSEARAICHGDGGHLATVKN

NETHDFLTNHVRATTRDHTWIGLSDQVTEGLWVWDDGTSLVGDGIWGTGEPNGSTGENCA

QIYPGHHNYRWNDSTCPSSYYYICEVLA

>C3YLI4

MQIFLTASCPEGYTMWKGACYKAFKALKIFVLASVACHKDGGLLAMPKDAETNAFLISLY

KSVGNYAFWFGLHDRYEEGSFEWVDGSALGGFTYWSPGEPMGSGDCVLSAPQTGKWQDIP

CGNNADFIC

>C3YMB1

CQKGWKENKDFCYKFVTSTANWISAASTCRKQGADLASIRNSEENYVIKDLIKDGPKHVW

IGLHREGGAWMWQDSSPVTYTNWAPGEPNNRDGRENCVIIYSRVGKWNDISCLCKYPYIC

KKQKN

>C3YMY1

MIFSTGCPTGYTAYGGSCFKANSQETTYSHAREMCAADGGILAMPKDAGVDSFLRGLKNV

LDAISRFWIGLSDQNDEGEWMWEDGTTHDTIGDWSNWQLGEPNDNQGGEDCANYGGSGWN

DAPCSSAYKFICQLK

>C3YQ56

MPQRHSHYLPTACPAEYKLFGHNCYKAFTTSLSWQDARSTCIADGGQLAEPVNASINQFL

IELGDTKGSSLDFYIGLHDQNTEGAFEWQDGTPLGSFSDWGYGEPNNGGNEDCALFNQFN

VWNDSSCSYHSPFICE

>C3YQP8

CQSGWEEYDNYCYKFVRRAAKWSTAESKCRQQGASLASIKGRSENVFILHHFSYGKRAWI

GLRRTGKSWKWNGGSPATFRNWAPGEPNNRFGRGENCVQIDLRRRGWWNDKTCGSKYSYI

CKKLKNPA

>C3YQP9

RCEAGWEEYSNYCYKFVRRTATWRAAESRCRQQGAVLTSIMGSQENRFVSSLFSRGRRAW

VGLNRIDKSWQWIGGLPVTYTNWAPGQPTMKKWGQRERCVHIYLKRRGMWNDDTCGTKYS

YICKKPNKCF

>C3YUW8

NNHCYKFVRENARWSTANAACIGYGADLASITNREENTFIKHLISNAPEDSVWIGLCRTG

GSWKWNDGSQFSYTNWDSGEPNNEHWNNEDC

>C3YUX7

VCQSGWTEHNSHCYRLGDGPADWDTADKMCKEAGANLASVENDAENNAILSAINTEFPVV

WVGLQDVNKGGALTWTDGSPVSYTNWAPHEPEN

>C3YWI6

QCQTGWNEYNNHCYKLMAESVSGDSANQKCRGMGANLASVRDSQENTFIANTIKEGDMVW

IGLKRKKKGFEWTDGSQFSYTNWAEGQPDNGN

>C3YXV7

TVNQCEDGWDEYNDYCYKFVRKAAKWSMAKFMCHEQGAVLASIKGRPENGFISRRFSNAW

IGLHKTGRSWTWIGGSPLTYKNWAPSEPNNYLNRREYCVYMLLKPWRKRGKWNDVTCGSA

RPYICKKPK

>C3Z141

SCPNGYTTWSGICYKAFNTEKTFSDAAAACGEDGGTLAMSRDAETNAFLLSLHNAVSNGP

FWFGLHDQREEGSFEWVDGSALGTYNFWGPRQPNDYGGSEDCVVYSNDMWHDFPCDYRGR

FIC

>C3Z3G1

TERKCKSGWKEHKNNCYRFMTEKAGWSTANARCKDRNANLVSIHDKAENNFIQHLISKGG

KYYPVVFIGLHWKNGQWKWSDGSRLSYTNWGPGEPNS

>C3Z4C7

MIVSHFKGDKCLKDYVLHDGSCFKAFPKAVTYSEAEATCAAEGGFVAPTKTEELLRLIRK

LIYSVKPLANFWIGLDDRQTEGVWRWSDGTVLGAEDFQTWVPREPNNYQGTQHCGRYWAS

MEQWDDVVCYVRYLFVCQIGKDFMWPEH

>C3Z7X1

QALPCQTGWSGYKNHCYLFVRNKVNWFQADKECKQHGANLASVTNADENNFIARLLTGPA

KDRDLVWFGLKIQHRQWAWVDGSPLIYTNWAPGKPGN

>C3Z8H3

MSTEKLLIDTDGSFGRRPAHVPFAWLFQLVTAILSVITIVLVVHGGTRLATQDRELSTLK

TVVSDYQKEVLAMKAHQEARKDAEVTFLRERVVALEAKLTSSTSANGDINLQAELGDQVD

PSVTETTDGGATWRPGAVHHQRAKRAASSVTFPAGACLQGPPGIPGRDGRDGLPGRDCPC

GAKAPLTCPGGYVQFRDKCYQFSTTQKQYNDAKAVCLASGGHLAVAKDEATDNFLVNEIR

KRGNPETWMGMSDQVQEGLWVWEDGTVLTGWSNWLPGNPSSTGEDCAEWKNVYDYKWNDD

ECFHTQYFVCEVDAS

>C3Z9L6

MQDIWPTFCKISNIITALQCPSEWSKYNNHCYKLMTDRRKWFAASSRCRQHGAMLISIND

QAENYFVKNLISNYKRKVPAIWMGLHKKSGQWKWTDGSRLLYKNWVPGEPNNNKWYSNFK

GENCAVVYSKWNDFRCGAAYAYICEKQSNN

>137265

MLGLQIFTLLSIPTLLYTYEIEPLERTSTPPEKELGYWCTYANHCRFCWDCQDGICRNKA

FKNHSPILENNYIANCSIYRRNDFCIYYITSIKPHKTYRTECPQHINHERHEADIRKWQK

LLTYGFYLAGCILAVNYIRKRSLQTVMYLLVFLVISFLLSQLMLYGELEDKKHKIGSIPP

KRELEHWCTHGKYCNFCWDCQNGICKNKAFKNHPPIGENDFIRYDCWTTHLPNKCSYEKI

YKHFNTHIMECSQPTHFKWYDNLMKKQDIM

>122151

MAPRSLLLLFSGALALTETWAGSHSLRYFSTAVSRPGRGEPRYIAVEYVDDTQFLRFDSD

AAIPRMEPREPWVEQEGPQYWERTTGYAKANAQTDRVALRNLLRRYNQSEAGSHTLQGMN

GCDMGPDGRLLRGYHQHAYDGKDYISLNEDLRSWTAADTVAQITQRFYEAEEYAEEFRTY

LEGECLELLRRYLENGKETLQRADPPKAHIAHHPISDHEATLRCWALGFYPAEITLTWQR

DGEEQTQDTELVETRPAGDGNFQKWAAVVVPSGEEQRYTCHVQHEGLPQPLTLRWEQSPQ

PTIPIVGIVAGLVVLGAVVTGAVVAAVMWRKKSSDRNRGSYSQAAV

>82000822

MLPSLQSLTKKVLAGQCLPTDQYYLLKCYDLWWYDSPITFDHNLGLIKSAGIKDGLDLNT

ALVKAVRENNYNLIKLFTEWGADINYGLVSVNTEHTRDLCRELGAKETLNEEEILRIFID

LKFYKTSSNIILCHEVFSNNPLLQKVNNLKMRIEIFWELRELIKKTDLLNNEFSLNTLLL

KYWYAIAVRYNLKEAIQYFYQKYTHLNTWRLTCALCFNNVFDLHEAYEKDKIYMDLEEMM

RVACIKDHNLSTIYYCYVLGANINQAMLASIQYYNIENMFFCMDLGADVFEENMPVGEGY

ELIRNILSLKIYSPSTAPLPKNTDPEIIDHVLKNYKSKNMMTFLSYDLR

>81861101

MADSAQVPALVYLVTGGCGFLGEHIVRMLLEWEPRLRELRVFDLHLSSWLEELKTGPVQV

TAIQGDVTQAHEVAAAMAGSHVVIHTAGLVDVFGKASPETIHKVNVQGTQNVIDACVQTG

TRLLVYTSSMEVVGPNVKGHPFYRGNEDTPYEAIHRHPYPCSKALAEQLVLEANGRKGLR

FGGRLFRAIPASVEHGRVYVGNVAWMHILVARELEQRAALMGGQVYFCYDKSPYKSYEDF

NMEFLSPCGLRLIGTHPLLPYWLLVLLTALNALLQWLLRPLVLYTPLLNPYTLAVANTTF

TVSTNKAQRHFGYKPLFSWEESRARTIHWVQAMEGSAW

>74749902

MAELRQVPGGRETPQGELRPEVVEDEVPRSPVAEEPGGGGSSSSEAKLSPREEEELDPRI

QEELEHLNQASEEINQVELQLDEARTTYRRILQESARKLNTQGSHLGSCIEKARPYYEAR

RLAKEAQQETQKAALRYERAVSMHNAAREMVFVAEQGVMADKNRLDPTWQEMLNHATCKV

NEAEEERLRGEREHQRVTRLCQQAEARVQALQKTLRRAIGKSRPYFELKAQFSQILEEHK

AKVTELEQQVAQAKTRYSVALRNLEQISEQIHARRRGGLPPHPLGPRRSSPVGAEAGPED

MEDGDSGIEGAEGAGLEEGSSLGPGPAPDTDTLSLLSLRTVASDLQKCDSVEHLRGLSDH

VSLDGQELGTRSGGRRGSDGGARGGRHQRSVSL

>74963705

MSGVTAIEIPQWIQDNQEDFVPPVCNKCMFSDQLKVFYVGGPNQRKDFHLEEGEEFFFQR

KGDMVLKVIEKGQVRDLVIKQGEMFMLPARVEHSPQRFSNSIGLVVERERKNTEFDCVRF

LVGSSNITLFERWFYLTDVVKDLPPLIKEFYGSNEFKTGKPGKGTFACNAPYEARWTDLP

VPINRKEFIYDHISEVKNGPVRIYGAPEYKTEVMLLGEGSYDLESGTVELLIWLQENTFA

VVEESGFTYAMKSETMVRIKPNTKCLLNVKGGFAITIRMPA

>46576443

MMNDAKRNQFREDDEEDRLIDWGSTQSLQSQFFDRRPAVVARQLLGCGFARRIEGVWVGG

WIVETEAYLSSRDAASHSARGEKPGNASMFGRPSTLYVYPIHAKHCVNLVTESVGCGSAV

LIRALQPVWGIDRMFQHRGLHRSETTDGRALTTGPGRLCQSLAIDRTCDGVDPIRDPNWC

VFSGPKLPSSRVTTTPRIGISQAAELPLRFFVDGNRYVSGLVRHHRRPRRDSL

>A6QP41

MLFEGKVSPQTQNLQAECREWAGRSLHLRVLGRQLIPPADEGVEHFQSSRPASAIQRPFL

DDCGHSSNIRELCISGSQIVPAVLPAPSLPGPDGTRVVDLMAHSSLEEEIFDVDGKIEEY

FAFDRKQDGEDCLEQKPAHRRRTWRKHGLPPVSPHDCIRDAVAAEVFDHVWMNVVEVLEK

LTRKHWELTEGKKQKEKLKVAENKSPPTVISRINADVSSVPPSRSSETRSVSLASHLNPQ

QIHRFSNNFYSDLNGVMTIQAKPLQQRPTCFADRTQSEQEDKPLGVGSAVLSSAQNRLAR

ITDARGPQTSAKKTLAHRRLPSLTSDSQRLKIPSVYSDEVLRGTKLQTGVDHMGSPPTQT

PRSRLPPIGSETGEHHVAGPGSRPVSLRGRHLQNHGLSALPDSVERSPLRERSLTMEQFS

RPSTTHTFRSDTPRKGSLTLMEFAGHMWTGQGFLTGSQYPPKSFQRTTLTLRKRFQVAS

>O18741

MVLCLNVILFLTLHLLPGMKSSMVNLINNGYDGIVIAINPSVPEDEKLIENIKEMVTEAS

TYLFHATKRRVYFRNVSILIPMTWKSKSEYFIPKQESYDQADVIVANPYLKYGDDPYTLQ

YGRCGEKGKYIHFTPNFLLTNNFHIYGSRGRVFVHEWAHLRWGIFDEYNVDQPFYISRKN

TIEATRCSTHITGINVVFKKCPGGSCITSLCRRDSQTGLYEAKCTFLPKKSQTAKESIMF

MPSLHSVTEFCTEKTHNTEAPNLQNKMCNGKSTWDVIMNSVDFQNTSPMTEMNPPTHPTF

SLLKSKQRVVCLVLDKSGSMSAEDRLFQMNQAAELYLIQVIEKGSLVGMVTFDSVAEIQN

HLTRITDDNVYQKITAKLPQVANGGTSICRGLKAGFQAIIHSDQSTSGSEIILLTDGEDN

EINSCFEDVKRSGAIIHTIALGPSAAKELETLSNMTGGYRFFANKDITGLTNAFSRISSR

SGSITQQAIQLESKALKITGRKRVNGTVPVDSTVGNDTFFVVTWTIQKPEIVLQDPKGKK

YKTSDFKEDKLNIRSARLQIPGIAETGTWTYSLLNNHASSQMLTVTVTTRARSPTIPPVI

ATAHMSQHTAHYPSPMIVYAQVSQGFLPVLGISVIAIIETEDGHQVTLELWDNGAGRDTV

KNDGIYSRYFTDYYGNGRYSLKVHAQARNNTARLNLRQPQNKVLYVPGYVENGKIILNPP

RPEVKDDLAKAKIEDFSRLTSGGSFTVSGAPPPGNHPSVFPPSKITDLEAKFKEDYIQLS

WTAPGNVLDKGKANSYIIRISKSFMDRQEDFDNATLVNTSNLIPKEAGSKENFEFKPEHF

RVENGTKFYISVQAINEANLISEVSHIVQAIKFIPLPEDSVHDLGTKISEITLAILGLPM

IFSVF

>17432907

MHCKVSLLDDTVYECVVEKHAKGQDLLKRVCEHLNLLEEDYFGLAIWDNATSKTWLDSAK

EIKKQVRGVPWNFTFNVKFYPPDPAQLTEDITRYYLCLQLRQDIVSGRLPCSFATLALLG

SYTIQSELGDYDPELHGADYVSDFKLAPNQTKELEEKVMELHKSYRSMTPAQADLEFLEN

AKKLSMYGVDLHKAKDLEGVDIILGVCSSGLLVYKEKLRINRFPWPKVLKISYKRSSFFI

KIRPGEQEQYESTIGFKLPSYRAAKKLWKVCVEHHTFFRLTSTDTIPKSKFLALGSKFRY

SGRTQAQTRQASALIDRPAPHFERTASKRASRSLDGAAAVEPADRTPRPTSAPAIAPSPA

AEGGVPGAPVKKAQKETVQVEVKQEEAPPEDAEPEPSEAWKKKRERLDGENIYIRHSNLM

LEDLDKSQEEIKKHHASISELKKNFMESVPEPRPSEWDKRLSTHSPFRTLNINGQIPTGE

GPPLVKTQTVTISDTANAVKSEIPTKDVPIVHTETKTITYEAAQTDDSNGDLDPGVLLTA

QTITSETTSSTTTTQITKTVKGGISETRIEKRIVITGDADIDHDQVLVQAIKEAKEQHPD

MSVTKVVVHQETEISEE

>81935510

MFSLQELCRKNIYILPYPLGEHVLQQLGLYWKGYGSLQRIGDDHVLLQQDLIFSINEALR

MAGEEGNNEVVKLLLLWEGNLHYAIIGALEGDRYDLIHKYYGQIGDCHKILPLIQDPQIF

EKCHELSTSCNIRCLLEHAVKHNMLSILQKHKDQIRFHLALTQILFELACHERKNDIIRW

IGYSLHIYQLETIFDVAFAHKNLSLYVLGYELLMHKVNTEAANIDLPNLLSYHLRTAAAG

GLLHFMFETLKHGGYVDKAVLSAAISYKHRKVVAHFIHQVPRKTVEKLLLHAVQTRAPKK

TLNLLLSSLNYSVHPITKQLVRNVVDYRSTLIVKLLLMRRKRKLNLVDAVLARLVRYSTY

TDTVKFMGEFSVSPEKVIKMAARESRTFMIEMISKAVWKNHPQTMIHHLKQLADTMKPQS

GKDLIIYTIHYIYQSSNLLVAEEEKNIFKLAKFYANHNSVNRFKQICEDYYTLDVDTRFK

TLILECFEIAVQKNYPRIATIVDDFIRFLFYKGDITEEEISEAYSLKNAELYVDLKWLQQ

EEN

>112833

MPNYAIFRFEKHKTVGTIKAASLHMTRGRETQNADPDRKELNEILKGSTDPSADVKSMLN

KIQKETGKPLRKNGVQAIELFFGMSPEWSKQATPEKLEHWKTITQQWAEQTFGENNLVSL

QLHADETTPHLTGFMVPRDPDTGRLNASRWFDGRKALSALQTDYAASMEPLGLARGVKGS

KATHQRVQRHYGNINKTLQLDPKIQAPIPPSIFTNKEEWAEKERLKAQKSALSVIQPLAD

KAARYVEEKKRADRAEEALSLARRKADSMRAIPLSDVLKTLGMELDPADKKQWRDPEHRF

RITIDNYKFYDHSAQKGGGGAIDLLMHTTGQDYKGALSWLADRFGDETARHDMLLNDLYR

SKIRINEAKQRPAFKQPEHKNEPKIREFLNSRGISFNNIPDSIRTDDRGNVAFLMYDDKD

TLQGAELRGTSSGFKGLALGSSREAHFTGSINVKNDEKYDLYIAESAIDAISVVGFLSPE

KIKAGVKLLSTSGVRTSLTKTLRKIVEKASSVHIAYDWDAVGQRAASLLVGAIKAAFPTK

KVENWLPPKEQMIHGKDWNDLLMVKRGLKKTVAPKQTVKRKIRF

>3023202

MLQSIIKNIWIPMKPYYTKVYQEIWIGMGLMGFIVYKIRAADKRSKALKASAPAPGHH

>112851

MTSRMAILSGLLFWLLLEWNPAFAYSPRTPDRVSETDIQRLLHGVMEQLGIARPRVEYPA

HQAMNLVGPQSIEGGAHEGLQHLGPFGNIPNIVAELTGDNIPKDFSEDQGYPDPPNPCPL

GKTADDGCLENAPDTAEFSREFQLDQHLFDPEHDYPGLGKWNKKLLYEKMKGGQRRKRRS

VNPYLQGKRLDNVVAKKSVPHFSEEEKEPE

>A0A1G5

MRVRTVTRFTSLVLLLWMSCVKSEYLFTMPRSLRGGSQGKYCLTLRNLDHENPAGYNCQV

SLHFLTSESIITSEHIYAFNSTESDWSQCVNFDAPFKEDRYTATVTVHIDGKKLFMHDEK

VDVWTAKNITLIQTDKPQYKPGQTVKFRILRMDYRLLPLTDLFELITIDNPGGVRVMQWK

NVDVSKGLVSLEMKLSDDPVLGTWNIKALGADDTVQSFKVEEYVLPKFEVKIMPPKYLLP

TTTSISGKVCADYTYGQPVKGSLTMKVCFGPEYYPSYYPEQPCVDVIETNFDGCHSFSIS

PSELKLGVQGYPTWGSLKISATVRETATGIELNGTSTGPPLTNDPLKIEISDESDGYFKP

GFPYKGKVTVTLPDGNPASNEVIRIKAEKYNLQYYWSREFTTDSSGIINFSLSNLGRDVY

SLSLMATAVKYEKQAEIGYRPYSLRVFTPNGYRTITQWFSPSLSSIYIPTIKDSVPCDST

VDLDVIYSTDGSSTNVFNFVVKSGDDVIHMVRKKVVFDGVDIPMNVLQFNPAEELEYHEK

EVTKAPRPPRPPFVTKGSPLPKVPEPEPVPVVEEEIIPKVSTPLGGGSNQGAEVQEELVV

PEVVKSLGVEKAIEGGKNPKRRSSDEPGMSYGIGYFKMPVPIKTSGSEVTVLVYYIRQDK

EVVATSLKIPVESCFKNKVKMEFASAKVRPGEETMFRLQASPNSLCSVGMVDKSVNLMGG

DHQLTPARVLDEVKKVQTGGRYHSYWFDDEKYCMKKNKNETVSKGMYHHHHHFTSGRPET

KDSIEAFRSSKMLVVTDVLLETRPCSEPSPYYPIAYSSVVAETMGLPGLAGERSLNFRAK

SHPKGNEMERESAEDTQVQTVVRSYFPETWLWDLHTIGDDGVVNVTTEIPHTVTEWVGNT

LCSNSKDGVGISPMIGITVFQPFFLSFTLPYSAIREENLPVLVTVFNYMTECLTMEVRMK

ETKDFRIQSVSGAILKMCVCGGDSKSAKFHIVPLTVGEIDLEATAVSIEDDATCVNQIIS

KEGVGVQDGVRRKLLVEPEGIPQEYTTSFYLCPEGRLLSQDIDLPVASADKLVPDSQRAK

VNVIGDIMGPTLSNLKDLLKMPYGCGEQNMASWSPNIYVLQYLTNTNQLTDAIQDEAKGY

MRVGYQRQLKYRHHDGSYSAWGDNEYQNSTGSTWLTAFVVKSMAQSRPFIDIDSKDLHLS

MQWLLKHQNGDGCIQSVGKVFSSYLKGGLADGENVGGLTAFALIALLEAGIDKNDPAIVN

GFTCLSKQQTNADTYTLTVMAYAYTLYNVDSPKRGQIMAELEARTRVPNPGQKHWIREEE

EKKEKDSNYFYWRAPSAEVEMTAYVLMAYIAGGQEGAVSTAQPVVQWLTKQRNAQGGFSS

TQDTVVALQALSMYATLVYQGGLDISVRVDTPSKSYQTGINDSNSLVLTTWDLSPQTTKL

NVQVQGKGCTMVQANMKYNIYKDEEKETGQASFEVKVSVYRSRTNIDNCKRRTLRICARY

ALPNFSNMAIVEVKMITGWIPVKSTVKELLAAKQIQKYEINPDNVDFYFDEFDSQERCFA

FEVEQTDIVVTDPKPALIKVYDYYETKDSVMILYDIKTTCGTKEELPFPKP

>A0A7B9

MSAIPALVKLGEVQMDTDSLRLEQQLQLRAMSDVIDKMTLEQARDRLKDMLKQAMIRDNL

YAAIIKKNWGLEPTTPPNH

>A0AEN7

MNKTTDMDGTAYDFTYEDLAWSITFPKSKTHAKELAQLELLEKPATHFVPATISSDSDSY

MISYEIDKHTYGFEQIRKMEREDKLRALRNIADLSELLNTRYTFFLHPDNLIFDINLVPR

IVHRGIKNILPPYELTEETFLKQYQCFAIAMFSKKFTFENLYNGSLSSARGTQFEKNILD

AKTVQEVAAVLEEAYIKENKSMQKNMARVPKKKYGAFRGLAVGFIIVAILLAIPVSYFAF

VKVPLQDDLLTANEQFLKTDYDKVITGLEKVDPEKMPQSVQYELAYSYVNGEKMSDKKKE

NIMNTISLKSDPKNLLYWIYNGRGEFSESLDIAKLLDDPTLAMYSLTKQIEQVQSDTKLS

GEEKVEKLKTLEDSLKAYDDKINEQTKETDESTNTEAK

>A0AFE5

MKQPQKLFKYLFIIMIMGISLWIGLGPEMEVHAATLNESTPINQLFPDPVLAEKIRSTTA

KPSVGSTVTQSDLNKVTYVNIQGYGKEPIKSIEGMQYLNELSYLSLDGNQVSDLTPLANA

TKLTYLTLSDNNVSDVSSLKNLSKVYLIGLKNNQVEDISSLSNLTALKYLYLNGNKLSDL

SAIANLTTLDILEVKNQQVTKQSVAFQNNLVLPNTIKDTKGASIAPTNISNNGTYSNNSL

NWSLPELTNEVTYSFSQTVTAGSKISTEFSGTVTQPIHETIYHTAIFDVDGVQINDTKEI

STLIEEPPTPTKEGYTFIGWFDEEGNEWDFSTDKMPEKDLTLYARFDKDTSEVVVPEEGT

ETPAEEVEVPEEGTETPAEEVEVPEEDTETPAEEVEVPEEETETPAEEVEVPEEETETPA

EEVEVPEEETETPAEEVEVPEEGTETPAEEVEVPEEGTETPAEEVEVPEEGTETPAEEVE

VPEEDTETPAEEVEVPEEDTETPSEEVEVPEEETETPAEEVEVPEEEIETPAEEVEVPEE

ETETPAEEVEVPEKDTETPTKKMVASKQVQEISAESTTLIKNPEILETISSDVVEEPIEN

NKKAVKSINLNADSSKSQENTKDDSDYEEVKSNKIAENKKVSTQTSSFLPIIGDSQNFWF

SLMGMIIMALSLVFGRKKRL

>A0AL47

MKNKKRWLLIGVAILIAGLLIGSKVYMDKQQEAKVRKEKMIEIVKSEEAKKEFEVVLTNL

DSKALTSDGKINTYSISNDTIRKNLMGGINVVLVINDDANLTIEYTLNMINGHLEAGGVS

YSGSLANLLEQNHNE

>A0AZH0

MIGLESLYTLAGLMFAAFAYFNLTDRTNPRRVVNFAFWAIYAVTFLFGALLPHFVTGCLA

IALAVIAGSGKLGRGRSDEAGEAAAGRRETLAQRFGNRLFLPALLIPLVTLIGTFALKLV

PFVEPKSVTLISLVLGTIVAFVVALAMLRDSPVHALKEARHTMDAVGWAAILPQMLAALG

ALFAVAGVGGVVSGLVKDWVPIDSPFAVVAAYTVGMALFTMIMGNGFAAFPVMTAGIGLP

LIVHQFHGNPAIMGAIGMLSGFCGTLMTPMAANFNIVPAALLELKDKNGVIKAQWPTAVL

LLAVNTLLMYAFVFRF

>A0B1W2

MTTPFRLGDHVRWNSEAGYVTGTIIAIHTADFDYKGHRHRHRASPDDPQYEIKSDRTDHV

AAHRGRVLERIAGKDDA

>A0B526

MADQRSPSGLATFSRGTALWWQQAKLLTSAIVAVVIAAILVGALAGGAYLYFVTSAETRY

LTIKHVEAEILTVVPMANPNVSFHLGNDDTALPPAAALSFTEDAADEFFIYARNAALYAV

LTAIAVTFLSVLFWMEYGRGKLTDKQVRGAKLVKAKELIKELEARDDASPYRIAGVPMRK

KAEVLGTLFSGAQGSGKSQQFFALMDQVRARKKRAIVYDPSGEFTAAYYREGKDVILNPL

DARSPNWNAWREIQSEIHYDGLAEGLIPIPPNVADPFFAIAGRMVFKGAIRALGGDNKRT

NAALYKAIAHSNLEELHAILANESAATYVDPVTERTGMSLKMTVQNQLDCFRYLHDEGEP

FSIREWIQNEDEDDDSWLFITVNEEQKTALLPLISLWCDIAIRSLLTLKPIHRERLWYFY

DELPTLQKLEIMKLAVTNIRKYGGCFVLGIQDFSQLFQIYGEHLARTIISGCQTKLLLRV

TDGDAAKALANAIGQADLDEKEESLNLGIDERRDGMSVFARRNLRDLVLSSEILHLPDMQ

GYLVTPSDYPVARVKYEYVPHPQTVTPFIPRQTYDLWLPAGAAADRPAAPERQSTALPSP

PSPTARFGSDPSPSHSPSPNPSGAPTTAAEDSATNAASAGPAVGAPAPNTSGCVARPSNV

ISLSTGEIVDGDTGEVHSTSAPREPVTGERPTGRAGLGDLLG

>A0B548

MEAKKSLSEINERIRDGSVRVVTAEEMPSIVEELGPDGAVREVDVVTTGTFGAMCSSGVF

LNLGHSDPPIKISRAWLNQVEAYGGVAAVDLFLGATQPSEDRGIEYGGAHVIEDLVSGRA

VDVMGEGVGTDCYPRMEIETTLHLEDLNQALMVNPRNAYQRYNAATNSSDRTLHTYMGTL

LPHFGNVHYSGAGVLNPISNDPGFEYIGTGVRIFLGGAQGYIVGPGTQHSPGTGFATLMV

SGDLKRMSSEFLRAATFTKYGPTLYVGVGVPIPILNERLALSTAVRDSDITVPVVDYGVQ

RRDRPVLKSTSYAELRSGFVEINGKEVPTASLSSFHMARMVAGTLKKWIERGEFLLTEQA

EPLSKSGVSRPMKQTKELPYVGDVMNRDVVTVGENISVPEAARVIVGSRFDHLPVVSDDG

KLMGIITTWDISKAVANGNISRVSEIMTRRVYTATPDEPIELAARTMDIHSISALPVVDK

DNRVIGMITSNDLSRLFAGRRSI

>A0B6Q7

MFPGLGGRGMSPKKMKGMLKSMGINIDEIEGVEEVVIRTSDREIVIKNASVAVMEAQGNR

SYQISGDVTELPRVSPSDVELVASQTGASPEEARAALMDCGGDLAEAIIKLSSKTS

>A0B7J2

MRNLKTKHSFFVVNLGLALIISLMLQASAEFVTFHGDNQRSGNVSGSGPDSPELLWSTSL

TGHGYIGGSASISSGRVFVSNWPDMTFKGELGLACIDERNGTVLWINPVGGKGGASTPAV

FGNKVFTGSLTGEIYCVDVLTGKTLWNRTIERDPKYWGVASSPLIENGTLYVMSFSDGAL

HALSLDGDELWNVSTGSVSPFASPAASGERIYFPGGDPALYCINATTQKIVWKTPADAVI

TSSPAIWNSTVFFVTERSISALNATTGSTLWQRSINGTESTPAIAFGRVYVGTADGHVVC

LDTNGSQIWETEVNGPVRSSPLFLDGRIYFGTNTDGGAVYALNASDGSVVWMYTVNEYIM

SSPSASDGILFIGADDGRLYAFSSLQKDLLWSGEVVLENKSLNVSVKGDVYTISMKSALG

ALISFASSNGINVSVNDSLISIYGLTVESIGDLVSTKERAWRYWVNYPDEPVPLTGPESA

MLNDGDRLVFYYGERNSRPEDCPRIEITARLSKPDALFVTVGQQPSLREVMRDVPLNITL

TSPDMLDPEMNLSKYSLIFLEMIGSDSASVLETLLEEPKRRGVPVVLLNSPGYQNLASVN

LSAHPDIELYWEYGGVENMRRLIAYLAAHFCGVDVMVDAPMPAPKEYIYHPDAPDLFENI

TSYMEWYRYNTSAPTVGIASYYGDMGQPDRIDLIRAFERRGANVICIGFSNASSLERFFV

LNNTSLVDLAIVTKSFRLSYGDPDRGVEILESLNVPVLRGMRLYYQSPMEWTNSSINPME

LYFQVALPEMDGIFDPIAISGKNDTVYSSIEPEVERIADRAMAQIRLQKPNSEKRVAIIY

YNHGGGKDNIEGCYLNVPRSLRNILEHMKRLGYRIESDVPDEKVLVDLLAHQGTNVGTWA

PGELDAMVKGGNATLIPAEEYIQWFEELPEDRQMEAVNHWGPPPGDIMVFRNSSGSYMVI

PKLSFGNVILLPQPSRGWLENGTVLYHSTDVPPHHQYIAFYLWLKHEFGADAIIHLGKHG

TQEWLPGREGVVGGDDWPALLVQDVPVVYPYIVDNIAEGTQAKRRGDAVMISHLTPPIVA

AGLYGNLSDLKESVDEYRNVLNESVKDEYRMKILETCRDLHLDEDLGANLSALQEPAAFE

EFLPELEDYLEELKSSFMPYGLHTFGQPYENDSLVAMVRSMLGDSYIKEIEGALNSSDVD

RIENVSSSLLYEVVINGSSPETAQENLLGETGSNLTEILNLSRIYADGLRSCENELTNMT

NALSAGYIPPSPADDPIRDPQVLPTGRNFRSVDPRRVPTPAAWEVGRKLAEELLDEYRLK

HNGTYPRKMAIVLWAWAMTDHGVVDSEILQLIGARPVYDAYGGVSDVALVPLSELGRPRI

DVVVVPSGLYRDLFPEKLRLIDKAIRLAANDTDATYPNYIRENSEILKDMLLQTGNYTAE

DAEFLSRSRIFLEAAGTYGPNLDAPVSASDRWENDSELGSLFISRMSYIYGDGIWGGRFD

SGRSLSLEQQMEVYTANLRDVDAAVHHTNSNLYGFIDNDDVFQYLGGIALAVRTVTGSTP

EMYVTDVRDPRKERVHELGEFFSRELRTRYYNPKWIQGMMEQGYSGAREMDRFVEYLWGW

ETTVPELVSENTWNEVHDIYVEDRYNLGLQEFFRENNPWAAQVMDARLLETARKDRWHPS

AGVLEDLAEEYRDLVEDYGIACCHHTCGNILLKEYMSGMLPSTTTSSTGHAVKSGSSSRS

RHPYMENSTIPQGVGSDMEKQPESEADNVRGYVMENVTVDAAVPPVSGVPLFGIALVLFL

LLLVAAGMWRRG

>A0B846

MEIKEGSLVRYNGTGTVGVVKLIKEEDGETWALLDSTDLYYLTSYLEPIEKVPERKELSG

MSLEDLTERMKEEKELMERARLHDENLECGG

>A0B8V7

MCELSVYMVAGGKKELVMESVVRIVVMDGRILFEGILGGSMEVDGKLKEVNILSQEVLIE

A

>A0B8X4

MQYEFLEHTADVKFRAYGTSPEEMLRNAALAMFSAMIDTKTVRHEMVWEVELEAEDMESL

AYKWLSELLFLFSAELAVFSDFDISLSHNSVWRLNARVHGEHIDRKRHSFETEVKAVTLH

QFEVRPGDSDRPWIMQVVLDV

>A0BDT6

MTEGQSDTFWKSLYIYFNEMDFESQLYVKKIYKIIILTVAIISFIIAFFLERFSVCVYST

IGASVLCILVLAPAWPMWKKNQPNWQQANWQKQK

>A0BI83

MLQRAVLIFCPLCELEFEPKLTKTNICPNCLIAQMNITQDIDNDKEILLCRNCRRYQRPP

WVLCERDSKELLAICLKKVIGLQEKTIIDTKFIFTEPSTKQIKLQITVQKEAMNGTCIQD

SKILNFEEVYHQCEDCKREFTPHIWGACIQLRQRVNHKKSFYYLEQLILQHHMNQQMLKV

ESADDGMNFYFKQKNQALHMLEFIRSYLPIQVKESKEVYSFIKYTFVVDVPRICKDDLVI

LPHKLCHQLGGINRIQICYRVASSIQLIDPTKLKVCEISAEQYFKHDLDIRTMPLRDNCD

EFTILDVEKIKPSKNDQNHPFLNSLCTVELMKDNDSDYNQYQVKSHLGNFLSAGMTVQGF

DLNKYNNELDEIYDKPEIIIIRRKPEKNKKRKTQLKRLDIEENQEKKNKKKEGLEEQQIE

EFLEDIEQDQHLQETLNLQQIQTQQ

>A0BIE8

MSNKIEEEKPAQQPQQIQKIFDEDDDDFEEFEQEDLEVNHDLKIDIKQWREDWDDEDLTD

EFSIQLKQELAQK

>A0BLQ7

MGLNYLQKKNWHPGSLKNQEIVWIREQIQADIIKREQERAKKLAEEKNIEELKRIQVEAG

LIPKSHLDRMEWMYDVSKLEKQNQNSAEEYLLGKTATSQEVEKLADQREKAKQTQQYQTV

FKEETTNAKCEDFVLVHEDPMFQIMKGEQQIKQVLLQNPLKVKQLQEKIEANKKIKKEKS

HKEKKDKSKKKNGKKDKKSSKKSKKHNRSSSSRSSSSSSKEQEISTAISTGSSKSQIYQQ

YLKERLGNIAVTDEHGNVKADFSLMKKKYRKNLNPQQSQEELRNQMAADGQQRLQQQLKQ

IDEEPKQQQQGVGGGQFLNKIRKEIYNQNDSSAYQDRIKRNRHFQERFNE

>A0BPJ7

MSYRELRNFCEQMRALGYHRIISMENFRRPNFELVADILFWLAQKYDPNSDISDNIDEER

HRVEFIKQITTLFVSKARLKINPKRLYMADVYAVQEILKISTFLYKAQVSPPADEEEIHD

FSLPSKLSNIKSHKLLAQEITDLATRLYDQLGKEDEVKVAREKALQFLSNVSRGGNSQSE

QSQIQKCIQTILKQQDSNIQEMSKYVGGLERDQKQLEEKIKRKTKELEQAEKRLKGMTSV

KPAYQEEYDRQEYELEKLYQIYVEKFRNLVYLEHVLDAQNRQAELEQRRKDDQLKGVRVQ

IQNAQGKELRGDDDENDEQLDQLGGDSRLQSSNKEKRNNDFMRNQQGGFNRQQMDEDGGE

DVDDIDDIEGGVDDDEEEEDEDDGIENLDDDNEADF

>A0BSS6

MQDKQKPKLFLEKLFDLLESGEASNIIQWTVDKDAIKIINRVQLIRDILPNIFKQTSYKS

FTKQMNLYNFKSTKDESGFTVFINPHFTQSSLNLTQIMAEKRTIKKSKKEDTKKRSELQQ

EHEQLKQKLMALFKYQVTLQNEIKTQLEIHKHLQMRVGIIKKCIYHRKENGLKRRRKIYN

FLNSFFTHLKSSVICIHLTFTEIRSKISNFES

>A0BX84

MKGDYNFFSDKKDDPLDAVCPKLSYQQRLWGFLICSGIGWFLEFCAFISFFQGNGKEFAI

IFSIGNLVAIMSTLFLSGPKDQCKKMADKSRLISTIIFFSTLIVTLVLAFATDLTFLTLL

MTLVQFCAYVWYVLSFIPFGQRMLKKFFSSCCEFE

>A0C384

MGMCQATKRNNVQQNKKSDISQNASQVILDQYPMPKPKLGIGLPFMIKFFEQIKIIEVTE

SMALGVNQQKQVSKEQDYQCIYLYSEKAKIDIFECGFTPIDISRPDKIIEDKKQYKFLFD

FKNLWVFRDNQEEIEQVLNIFKEINVKVKQIYLFTKPLNTFIEHYQMLGEISILNSYYPL

LLFDYKDVQDATKRGDDTFRIYLHSEEAFEILIRQNQISLYNRNLQIRNLIFLYQVGNFS

EKLGQNQYKVLCYNLIQQPSGQINQQKLVKELIPQLQNLQQMGSNIVLIYSSKESQGMKA

ARLAIEQYLARGLLIYPEDIQKYNEQLRSLQVTPEQLIQQQQQQENNRLNQKQQEIIQLS

REQEIINLYDRWLKKIKAQQILLSSHNLFIKLITNIIQYPNDEKFRTIQKSNKTLNLNIL

QYDEGRQILQLIGFREGETDFKNIFELGLLKMARADIEIAWKKQLEKSLT

>A0C428

MLTRLTRFVLNLQSQQFSNLQIKQLLHLMAFSHLINCLLLAFGPFFVVYKARSLAEQGAY

KTVLISILGCLLTQFCKLFLLASLSVITEQLIHIKLLFDLIDCYGIILVAKQKTNIDDKI

SRVFAIAIGWALMDCVMKHLFTFIPNATTDEFTWTFILRGFTANLEFFETLFLVGLLQSK

NKISYALILLKVLVLPNFQDIITRGGLSSAYMLVSRFVI

>A0CAD5

MPAEIARGKKYQQQHINNDLPTKDELFSQLGIMPDDDSVRANDHRGDHLRISKQLIQDKV

RDYCISLQQEQLNYLPPKPPTPLVIKQKVSTQRLTSQELEKMLEEHRQQMRPIAEQYEQM

RLSDVRSKSYNPKSQRSKSPKVKVTYHEEPVNIDKLRRSQKDIEGPHGDFLKGVLPKQKL

QKFKSESKLISDDDFQKKFKNYLRNKRKQDLSRNSSQEKLEFDLYQSTSPNRGRWNQKHI

NRAVFKDRSVLGNDSSISKSKSISKSKSKKDKKRDQSMSEQQLCEQIFKQIDNKQVGLVD

KVQTIKYLISNPEIMEAFNINPKDLNQQVNKFPTKQQGVFTQSEFTKFLQRYKSQYDHEA

FGGLSHVSQQQQVSSCLLNEDQIQILKNIFDQLQNDMIAQRKELIQAFRNDIQVVRMLHV

QAAYVAAIDKILNLESILSQLEAQAFEFPYISWKQFMDAFEYDTMVSKQQLSFANLKESQ

KQVDDQDRIDAPEELQQLIKDQFNKIVMDDYVTAFDLIECIRRAPLYSQLRKQIVRKQSK

KSDIKEETLEQVLKRIEETAEEYLTFQEFMSYFTRRGQPKFNEEVASLAKSVNVTEALQN

DENRIDGYDSDPELHTYQDKMLPRERLIKKHPSQQALLTYSHRKKSLPSKTPITKEDFLS

AIQPKPQYGAHSKPSDYQFKVTQPQPFNFDKREKERSLSIREKKLQEMLDDKKRNNSFKS

FKAKEVPQIVKQDGLYEKIMNDNEKRREEVKKNSVQLTLKNERPFSFYKRDKHSSKKPRR

KYWEERERFVFKAKAIPWHVHVELLRQMEQDEETKRKERIAKHAQELAMSSRMPPRMEMH

ERQKQGQPQSPKYMKTQLSYKAKPIPDFEKLHSSFQDQLNRKKLQMRTTEPEPFNFQQSH

KGVDRPYLDRENETKINPVKEDKIEEARKKMSIKPARQPPSTKKWESNCEFLKKERIKKA

EKQEQLKKEEQERLAKKEKFKQRVQQSEAIQDNSKKLEQQRKDRIAQLKKESKEQEMKQK

QIIENCKRKATEQPLLVERPTKRDVELEKMKQLNKIDKMLKQNGVMNRDEYFNKEEKAML

DDIHYLKKHGYEDDFEKE

>A0CDB5

MKKAHKYKKKNLEVCCPYNPNHLMPFSQLWFHLSSGCEDKQKFGHLYQICPYNSLHILQK

EHYEDHIKNCQKKIDVDDDLLKQMSNVAQEQFVEENQFQDQFISRNCNNKYKKYY

>A0CEV7

MSRYPQVFLDFQIGTQAAGRVIFDLFNDVTPKTAENFRGLCTGEYGNVGMAKKTKKLHYL

NTNVFRIADNMLIQGGDIINNDGTGGASIYGQTFVDENFSRRHACAGLLSMANRGRNTNN

SQFFITLKPCPHLDGKHVVFGQVIDGIEVIKRVGQVTVDMQDRPRIPVIIINCGEVSESK

NWLICDPFKKEIMDEIHRDRLKALYGQEYLDELDKEEEQKLQALNPEKYKQQSLENEEES

NKQLLVEQLLQKQTEKQEQEYDSQEKLHQFEGKNFMTQKHKERYLELTKKIQQSKVLNDK

AVLNEERQNTDAQYDKNLRKGKYLKKKEAEKQELEFKQIDEDKDYLNRMTLKHDEEQEKK

KEYFGWDVFNEDAVYNAYKKRCTTLAKNEGKYKQQMESNQEFLPTNEALERLSSDIANQQ

ERRKEFSRRRRFNEDQPVTYINERNRIFNKKLERFFGDYAADIKANLERGTAS

>A0CJS0

MLPKIQGQTVGRKENLASQQIVEDHLKNILKKQENQNYKAWKTPYAPKNPHSPFGDFPRE

YLPKEKANQQKVIEPVHETSQNALITNKYQGTHQGTGGRTSSQLQKKEEYRPAPPKAGAY

DQRTISVSDFRRYYDRGDLPIKVDHQGSVNRIIWKISPEQLDYHHYLPIFFDGLREKMDP

YRFLAILGTYGLLENGGNKILPVIPQLIIPIKTNLNTRDPSIMEVQLKVLQKLVLSGEMV

GEALVPYYRQLLPIMNLYKNRNSNLGDFIEYNQRKRVNVGDLIQETLELFEQTGGEDAYI

NIKYMIPTYESCILN

>A0CKT7

MISNQYDELMNSEFDVREFILKNFVQANFEINYKKIEQSMPQIAQIQSQTCEDIASSVDN

NLEDYVEISTQLTSLQSQLDNLTLQFSNITDIIVLTQDNLQQRIYQINVIVENLKLIQNL

QYSMKDLRKLINVLESVDFSKGMSNYNLLETSELIVKSRQLYTELDKSPHCRQLMLQFDL

LQIIQQNQCQFQNLLEKEFVDCLQNFNSIKFACILRSFQKMQLNKTPEKLLVQFLLKPAF

DMAFAKLNKNLLTKDTSNLEQFLQSVLQIYENNIEIAKVETETFSFKNILVELLYIYLST

KECVNIYSLGVLDLFQQNYQNIQNVKQTLKVNFEKGSEVEKKFMEKWNLSTYFSMRQAEI

IKKLESELSIQLTNNEYNFHLVLQNIIENCFSQKVYISLLRQKFYTLAIQIITRFCNFIK

SNKNSIQTDKIPIIINNLSKINLSVLQQDIIMLQLDKIRSVISELIDIAIVQIKIECQQN

LEPFSGIPAKFRMTNRDMPNQPSNFCINIFKPLQSYLLKLEDDSTKKLTCEKVCNTTLER

FQVIKKQALDTCDKNEELMAKMGVKKNDALDNQKIRMQFQLDEEEIKNIMITKLYQ

>A0CLA3

MIDTGKTNKYLHTPFEMFLMVIITAVSNGVMIPQIIYTYKIKNVHTLFLSGLLFVSSFMY

HLSESIGVEPIILKEVDWHQLDNIASICGLMELFNYLMQNNRSTRKSIQWINFIFVVIIQ

ESHAWNVWATVIPILISLIIFIIKQTFYPKKGQIVNKDYLHKGLFYLFIGFIAFYFGLDE

YKDYLRLWHGLWHLAMNQAYFHLYQLFNPKVYTFLECWDLKQEYAKE

>A0CSH2

MSQQQSQQIRNHFLDKKLIGTLFEEEEITKKDNFYNMEARDEKGHKRFHGAFEGGFEAGF

KNTVGSKQGWAPQKFISSRTNRAKYNNQTIKDYMDEDDIGRQKLGVNVTIQPEYDTFGQN

EINFLQQQLGSGSGWLLGSAPEELIYRDNKSVGYQVLMRLRKQVIKKRSTNVEQEKQETS

QAHNLKYLNSTNNQYTGLGYKQNIDTFGNPSNPLEQAFWTFIKQNASIIIDEDEEKRKKD

NRIKMNGFTSQDREEEYNNTIEDDDEPYQNKRKQVKGALDVGMRFTKDMIPLRIDTKDYL

INVPEDYDPYHKAKKLLDQGKDYSFLFINKTNFKKPASSSKREEIFREEETASSIKRDIS

KQKFVKGSNQTMLVTQQPNQQQMAENQDNINILNLFPHQPDKQQRYAYFVRQKLNKTTEE

QFQVDEEEKVDFERLYQMFQVTSKNVIEKKQEPQKPTEQQEKPNPFLSQRVVKRWIPEDL

FCKRMGVPQPYNDDEKAMIEKINERSHQKPNMATNNRNFLFMDKKEFKSGGFMQPQEFTQ

STAQFDPVALGLPLSFKSHSYERNQQERLLQLENMERAQTQVVSKQEKINIKFSEDKEDM

LPSRIQDQNIFEFIFGDDEEDDQQ

>A0CTY4

MDFIKSNPELKQKYDQFLFQQKKANTQTNHLKTKPLTDPEYLKLKDIQEYLDQEGGMMIE

PTPHFVLKAFDQNGEKVFFNVTAHSVVDAPEEKQLIDYNNEVGIRVPMSVGSIKEDHDVK

GDTCKVIDLVINPTVATNLTQDDNLKTFFCQLVQTYVDQKYKLKLQDKFISLKMKYKGKS

VQFQRVKGKKPPKVQVIDEKPNQSPVQNNDDEDETEQIRKRQREEFEKQSISTQPPAFVT

YEPEWELYLIYPNNEQEYDGDLDYNAIKQYRFQILTPLLITGKAIKLKVDEEQFQMVAGK

FYKISLRFPSKINKSSVKALFLTEKRTLWISADVKKEHEEEGNQQNEEQQVNEIQNAQQQ

SGSNYDLQSNLIFDIV

>A0CUE2

MAINLFQSSTNNIFGPFNTNSNNFPTNNNFNPTVPGSYRGTFKSQRITYGLMDAEDQNST

LYTTNMFMQQEYKGISAIQIRLEDYYLIRSQQIQDTHKSQLETAVRKNNFNSKLIKFTNQ

QNLKPPQFQDQNDIFRRQNNSFNQSNDLFKQPNSYQNIFNNPSDNNRKDIFSNNSNDLFN

KNNNNNPPNIFNAPPSNNLFNTNNNSAANLFQQQTNNSNNIFQQNNNNNRPFNSGNNNNN

IFNQSTNIFSQQPNNNNMFNSQNIFSNNNLFNSNNLFNQPQQNIFNQPMMFQYPNNSNII

QPILQAPLLGSIDYLKFQENQFKDFTQQVDSQIVMFEQQKQLSLEEYDCMLRESSKMNFD

YRLQNQYTQSMKSSKTFKLQNQKSSNMSNKTISIRQDKKTSSTYQAHSLNTETKSIFNRE

DRKPISKFQDNCQYTDSKSEFKSFVQRDERKSQSMFWLDIEIKFLESENSLTIYQEFQIK

SRVQNVKQFVENHLENIEIFGSLSQQYFNNCQIYNENTLVVDNSIQLAKLSDHRLIFHYF

YVKLPKLIRDGYSSNLHECPDLKQAQNFRVSNKFGKIVWTQPINIYYVDLDKVIDIKQES

IEVYDTNKIHELLKPDVGVKLNTESLITFKFSIKNPQINQIKFKNNLIAQAQKQGLQFMS

IDFQTFEYTVKSFHFSGYHFNSDYSNTDNQNYQDQQQQVEMQTSGDGDIFEQEENDQEPS

EIQSEIELELKYKFDFSFQDMRNKEKSINYQISNDNINKFNSRLSQKIVIPLNFVQQNQQ

LQIDQNLLQNYFNHYQKQEQVKKSQTISILLLDCLYQCITPSEEIIRSFQLFNILFGVPT

IDLVNFLQLQSNYVVSLQDVKKNSKIKYIRGLRLSLYLEQFKIYFNQVEYREQQIIQQQQ

SDPLQNVFISLNIKPKTHNEIKLNKSDWIDRFTDWRINRSQSQPLYETQWWEQLYNEQKD

HKIVSSQYAALIYLLLKKTKQPISEYKKLFEYLHSNLDTYTIILLNIMLSKQQEVSEQLK

IIGRSVITKLLKSSMPKQLIFSILETFRDKVFQKEELTRVQLLLGKLGYFNNLDGKDIKD

VDAQIDKKQFIQAAKIIIEKRLENQYYSILGQTILPYLVICDYYNEGDLRLLIKHIIDKN

NRDDDVIIICQMYLNDKQMNESSIIKWVTKNEYQYHLKQIMLKVYSDQS

>A0D485

MQKRFFIALLTLMLVQTKSVMKGVIEFTAPSCCDPIIRKITFPDKFSDIPQILVTIRAIE

YDAGPVGFFCQIQDVTEQDFTFQLTVLHATLKVLAYEYIAINQDEVEFGYFNVDALSMLK

KAEAGDRVIPSLLTFKRTFTEDPIVQVFLVGVESQSKLIQFEVSPLTVSLHGVQLNFKKF

GDTSVQKVQLAYVATQSSKELAQQVTHTGQKQAHIEPVDHTEESQASEKVEETKEFLQLR

SKPKLIQINGINGLRFTSLPVTFMWQGDQIDPTALNVNLRITLDKGNQAVLGLYYFEQKE

YKKCPIVYSMCNYQGSKVYLCKDVSDFNSIMRLAKSIYIPEKASVVVYQDPSYSGAKSKI

TKSIPCISDWFTDVAETEMSDIKQFLQINQMKRHKDQEFKEIHLDNQKQLNKFNMVQLPR

WNEAGDKIEVVQAILDSNSPPANSYKQELIQKLNEEEESFLKKRQIY

>A0D4Z8

MNPTVFDLFAQIKGLYTCKCYELLQRDDEFKKIARQSKISKSQLDSLEDKTALITRLKKI

INESKTAHKDALLSAKKFEIATQEKEKVFNENSKLKNQIKTLEGFRNTQTNQLNEYQEKY

SKVVDEEKQKKTELVEGFQQEIKDISTKMEEVSNAKHKSQAENEALKEKMKEIQEHVEKR

DKIFDEELNKLDSQRKERETKIFEQINNISQSLGTTDNSEDLLQKIKEEEALVEAHLNPH

LQKAEEFQQIIEKTNQQFNLYKNETEKLAQQCRSLEQKSQACQRKCEKSDIYIVDQAKEY

QKLQVQYKQKQEQINSLLTLKSTLQSGLL

>A0DBQ0

MNIIDEILTIGNDLSGPQIFAICMKWYEQFQDDLYNCNPNFSKIDNYSLCICQEEQVQIN

SILNNVKREFNMQHIKPIIIQQNSLEQILKVDKRNQAHYVSKALWEYLQEQMKGGPCIPL

YTMTKQESYLARTMFANLKFMTNNDIDDLMPNLVVPVDMTQIELLIQKEDDSLIYLTKVV

PLAFTIEQLITKIITPLFDISNLMCYNYYLKKFLSNNNSTFICELQKKSLWFLKSSMTIS

TGGIAFNENLDEAQMREDFQSLKTGSTESTTESRISLSIDNSQSQISPLHKFDNEGNHIN

LMLRDLDDFKNEISTVLKQNKNQQLVLLKFEQAMENINQIIQSIEQENCFKKEQQEEGTN

DELIEQDI

>A0DBS3

MSLRSVVEIVTHVESFRNVDLYYQGVYFLRITIHNDAPQDVHPSIHIQANKIYAHPHDPC

ESYKTYPQEAQQNVNPPKYYETHIFRPASIVTSNSAFYTKAFFIKFCEEEIELNDICNFR

IEFDAGPKKEQSLIMQVDLMFFDCLNSQKDQPKQEPLYTKQDKEDYAIPDGKIQATAKFK

IKNVLLPNHQFVPIIFEDQNFCQANMVVHTITLDYRFRTHPIQLFQFARLKFEERNQLLE

GKVPSTLDKNKNQSKPLINSDQISMYEAIQKQFKEMKGLDYYSMQQEFVESLKGYYEQLY

QHYNLIYSKCILEKQRKHFKKYLYPPLKLIVPEYITSETYQNMKGQLTRKELNQRIEEKF

HTSDPETIIHSILNETNLISCQLFQMWIKVLDLYRISPRFCVALLQFDYQKILKNRWQQF

CIKQPPQQLLDKNLGVEHRQKSEKMRLETKVQELGVEDLNSFPKTDTQPIIFEEFTPRED

KDKNEENIVDADITNSEYDIMSYRGIHLIVLVHGFQGNSYDMKLFKNYISLAHPEAMFLC

SSINEENTEGNIQEMGEKLATEVINFISENCPENTLGRLSFIGHSLGGVIIRASLPYLDK

YQDKMYTYISLSSPQLGYYYNASKIVDAGMWVLKQWRKSKCLEQLQMTDNRNIEETCLQK

LALAKGFAWFKNVCFFSCIQDSYAPYDSARVQLSKEALEDQKNKPYVQMVKALLRHLENT

NVYRIDVNFEIQEKNLDTLIGRTAHIQFLECQPLLRMIVSLYDQFFC

>A0DGY1

MQNFKRVVILLSKTSGRDKSCRILQYFGKFCAEQLKELKQDELSLKCKNLSSNMSLTRKV

LRFGRTIGIIISIMELSKQKGNKAIILNKILMNISCFLYFLVDHTHWFCKIQVIQNPQLE

AKADYWSDALWNFEAFFDCVALILEIREEQNKSQETKSSQRLFNLKLDLLRAFMDLLSAY

GFISNGRVPGKWIGFFGTISSIIGLKQQWDAAK

>A0DNK3

MGREFSGGRGDTRGGRGGDRGRGAPRGRGAPRGGRGGRFGGAPKAFVVPHPRLAGVFVAK

GQQEALVTKNMVPGESVYNEKRISVEDKQTGEKVEYRVWNPFRSKIAAGVIGGVSDIFIK

PGAKVLYLGAASGTTVSHVSDIIGSEGVVYAVEFSHRSGRDLVNMAKKRTNIVPIIADAR

KPLEYRMLVGMVDVVFADVAQPDQARIVGLNSQYFLKKGGHFMISIKANCIDSTNRAEVV

FQHEVQRLKDEGLTPQEQLTLEPYERDHAIVIGVYNV

>A0DPP0

MQISKAIIYGTIATWLGRRSDEKKTHSWICYVRGAHNEDLSYFIDKVVFVLHSSFENTNR

VVSQHPFVIAETGWGQFDIIIKIYLKGDYDQPLVTVHPLKLYQNQTQNIPLTRKPVVSEQ

YDEIVFINPKPELLEILNAKPTQENNQVEEEVQVDQKEPDFEQMTPAQILKYNQPHFTIF

DINESKVTIEKAIQIVSQDILADYKKKNHQLDTEIQELQIEYENLQNKVTQTQQ

>A0DQA6

MKALRFKHLYGEEIKSKFEQVSPTSITCDSTMVKANALYTALLWQTGGGGLVCVVKSDKP

TKLPFDTPMIKGHQGTILDLDWYPFDDEYLATSSQDQNICVWKINDLTSDITEPLTTLSG

HDKKVNLISWNPTSAWVLGSASHDQTVKVWDVQGGVARNTINANALPYSIDWNLNGSLIG

SSWNDKKLKIIDPRQQTISLEVNAHQGTKPQRFAWLGSTGYFVSVGFNKTQGREFSLWNT

SNPSEPVSTTQIDTGSGVLYPYWDEQLKVLYLTGKGDSSIKYFEFLDGQLHFLNQYTSNK

PGKSYAFFPKRAVDVNACEVNRVAKIEENALSYVQLIAPRKANTFQEDLFPPCPGTQPAQ

TAAQFFQGQNSNPVLVSLKPDQQQQQQQQQQNVVFTQQQPKPQPQQQLTQPDNQKIAQLE

LIVQNQQAQIRELQAEVEKFKHENDVLKEQLNQNEQQKY

>A0DTG4

MDDSFEINHESEELSEESVRDEDDFIERVNQLKKIVPNKQDDFTIMEEGLPDDLLELHEK

IQRKQQKKIEQKPKKQSYVPVNQIRTTLIIHLFKLYRDHKTMNNQTEFAFKLSNFLYPTI

EMIETKLNQAKNLQEQVSALYQGFALTFQLKNQFSKGIQIYNLSPSEYIITFAQLCKYYG

LNVRLVRAFDVGFLGLELKFKIRTIKKTDQESEETEQNTKTIFDLNSQFMTQSDNVESLN

KFIFQKSNKKDKTLIVSQELQKQQQQQNENKSQNDLQYELKKSKKKDNKKQQNERQINQD

IQTQNQNIWLEFYDSNSNSWIPFDPISDRFVLMDINILKAKLQNSFSHFIIAAQDLTFKD

PNFQINRLFGNTHFVDVTLKYSYYFFGQRFQLSLERWFNQLAYNAKLIYKGLQQLVNEPV

SANIIVPSPGSYTNEKEFKYSQFYAIASQLSQYQMIHPDAKPIGVKFKDEDIYLQSDVII

LHSRDKWREYLREVKLDAQPIKEVSQKFDKTKTTALFALWQTNDIKVSLEDNDGNLPNNA

YGNYETFSFPPPKGTRLVRLQGIKQLLSKNNIKFIEAVDGFDSQNGRMFAQKCGYLIFNE

DYDKIIALYEQFKIEINEKNKINKKKELLKQWSDLFKTILLKRDLQAKYQQIN

>A0DTT8

MIAELINQNILQKIILVPCGNRKDKQLTDGLHRYKMLQLLVETKDQLKNIDSVFIDDYEL

QNGQLVPTYYLLQKLREKYQNVHFVIGSDLVNTLPNWVEGQKLIAETNFIILNRSSHKIE

QITNLPPKYELVQNFEYGISSTEIRKRIKNSKTEYLDCLGVLTPEIIEYIKVNNLYRS

>A0DVP8

MIQMDVKEKKMYKFPTLSQRQMDLLTKLLSQQDRQNALPIINALTGNNGAQLRGLDTAIS

ALDKNHRDMFFQYVLPKMAKYALDMLELSPKKELLISEGSIDFTRKEIYQFLSLSFFGLL

VTQDEKFPDTYNLGHILQTDAEKSKCYLYYFIMASPDLENEIITVERIAFNADYHINQFF

PNSQAKILEDAQLWSNCEKSLQTIEFVDQKIEEQQNSILVDFANKYVGGGVLSYGCVQEE

ILFTIMPENIIAVLFCKVLDFHQVVIVKNTIRYSDYEGYANSFRFVQRQPKNMNQNILVL

DAINYKNNPDKQFQQNEIMREINKAFIGFSLSQSAKEEEFLYPISTGKWGCGVFKGNTQL

KTIIQLLAFSASTKSENQKRRMIFSTFKDKQLQFIKKDVDHIIGKYKTVGKLFSQLMKVT

PKYGVFEFLLGQK

>A0E089

MDKVQIALQNKFKKNNKLAITTLRKANQFDCLAVLIGTSHTSNIGIQKGFQQWYLGCELM

DCILIMSTKMLCIIADEVMFQKLKHLSDIKMKTFTIFFLIKNIKKNNHQQFQFALERLRK

EYPGNNYRLALNLSDGQKSPLITEFNQFIDQNHLIKVDCTSFLKELINNDNKDIFEYYNT

CGKINSYYMKFMSQRIELAIKFNENTTNYSITQAVKREKSSDLNQMAIRRKFGLQGNYDI

LSSTVQSGGQYNVSASESTQSRLVGDVVIYSFCCQYMQSQSYCTRTLLFQPNQELEQIYR

VILNVHAFALGLVKEDIQFKQIYRETQNIWETIFKDDPEMKMKFPTDIGYLIGSQMLIDN

HNIETIQDRMAVVIRMFVDNILVQLPFYPERTNIAICLADTIFVVSGIEDCVITKAEKEF

TFVSYQPTEEGERFFKSTFQKNENSDVLHQSEKITREQFEQAELNKIKNDQEKLKEIKQY

ELEVRLNDQQTRQEPKLLVKMDQLQAFQKEDQFDQYPKGEIAVDQDKSAILIPIIGTHYP

FHALTIQNVSVKELPNGAGEITIRFWTNEFHIDTREFPSMDQDQMFLKEITLRNQEFIKL

QDIENEINVCRDDARRKQIEKQLEVDKFDFVIEKLTVLPKNTPCLSKVYMRPTQSQKTRS

PEGFVECHENGFRYKSARGEVIDFTFTSIKHCFFVSPEDEVIACIHFIFKMPIKCGKIMF

SQIQFYRDIEGASEQEAARRKVRLFDIDHVFDKKVQDRRLEELKNFESFIQQSEQYYKRF

NIKFERLEKQYSFEGNYAKERVVFQPTQSCLVNIVDQPFFTLTLENVDIMCCERVQEETI

SFDLVAVLKDLEAQVIRIEAIDREDLKKIQQWLNKKKILFFQTTSGLMWRNMQFSIQKDF

PLFVYDGGWATMMKDHMEHAPIQQFNDEPLFEPDSSNGPTSVSEFEFEQDKKNNKYLHLQ

KDDESDFSDLVDSEDIMSELDIQERRKRKKVKYNFID

>A0E192

MSENQRTMTLTQLTHMIEEKVTEIQANPDQRISELPQLIELLKHENVFIVQMTIMALTDL

FIDIAPLYKIDQQAHEFKVTKFIKKEEKQVLNFELSLIKNYYQFIKAQFTFVKLISQGPL

VETCYQSLCKLLNSLFHFNYNRELVQYVTYGLLTCNEICYNSLANILRNKKHSLHESKLQ

ILTQIQKLYQTKHEDQLPDNLVDLVNQIQIDTKFLPQELEKQEKEKKRAVKQKKREYFSQ

KEKDKKKQKLTKEEMAKQKKLLKEVQDELQEAQGDLDKKTLAKNNGEILSKIFYIYFKVL

KLPRVSKYYESALNGILQYVHLINIELIQGIFECLLSSTNILRQKKDNLLKYINLRLKTI

FALQSIMDGPASVFGVDDKETMQRFYVCLLDMWTNKKIKITEEEESLILRILDSAFIRKR

HFSQEVTGSFVKMLIQLANNSENVRFVYALCYCIKLVMQKYQKTQKMLEEDNEGFGMNSY

NVKCDDPSCTNALNSSIYEEVKEIKNFRNSTTNKR

>A0E1T6

MKLLVLIFLSHLLMVHSQETEDIKGYMYVFAQEWPGSICKFQKCTKTYMGNYDNARWNTH

GLWPNTMLATSCGFISNCRDETYDESKLTVATKTLIDVTWNGMYSDTLTFRKHEWEKHGT

CHPDNLTQNGYMSKVGNLNNQYNYYKILASAGIYPDNSRELTDAEFRAPFTKTLGISTAM

TYTCQKDSSTGKFYIAEVRTCFTQAMKARTCDCSKPISAFVTCGKSFYYPTFQLSNDYVS

YVEELADEVSGIFQNFANEIMNILF

>A0E3N3

MYLILILALFIEQIECLGIELIDKKPKCVYMNAKKDDYITIKYLVVDGVPKDVQIFFFDP

YNIQYDFESNEQQLKALYTGKYRICFKNNGSSKTLVEFDFDILGIDKNYASKNDMLQTKM

NLNQIDSDFRWIQMLQKESYHREQTINTNLITLYKQLTISNIIKVMLFIVIISFQLFLIN

QFVKSQDMQSVPV

>A0ECA5

MKKFLNYLKASNAEQQEPITQNQSDQEHFLVLVGDIIKYQNEPTNPTKIISVIRQIILYA

IKGDNELHKQLFEQFMEIDVLNRLYDILQRRLCHHSLIQEIFEQVSSLLINVKEITNKNY

ILSHSSISNMVLWKQSFEQYQPNIEIVETYISFLNLLAVKLDDITTMFFINLKYPQFPLL

WNAIRFYNHPELMVRNKSRSIVLHILNIKNQNIIAYTKSFPFLQYYFNLGNQVLLTIKQI

NVVVEKIKWRFEGQQQDQLAMLSEELKEQLQYIHDLFINNEYLEQLILDYAIQRPVIQLS

QVIKYGYQHFHIAVALQAFLLLLLYFRTNAKIIQYIYQVFLSQDKSKITINEEFWGYSNA

YDNDPDFMKKLCLQIYQTSKPIDLLDLKHENLEIKMSLQQLKQVDCINQQQRIESEIKIQ

ESQILNSVYQHSSNKEVLIYVLLFMKELGNQHNIQTYYQYILKLASEQDQQLVVVILSLD

IIYAFKQQFDSQNYIKQLYHQILTELILNIKTSQFFLDQLIDIFKKKPLSIKREFIIDQQ

INVHHEKYAILFDPYSSLAKIQKLEYCILLSQHQIFEDLPKINEQYPELTKLQVPIYQIG

DKPDCAKMACEYSETGHFHSTETYYILNVDNFLHVVQLDQQGVIVDVICSKLWQLLQVYG

IERVQVLQFTYFDFSKVLDSLQQQFYLRFQNFDDVKYIDSLSSQQQQQFGDWFKELLIQK

FEKLQQENHLIFK

>A0EE91

MQTKGEMDFDTFKFFGIFIIKNVIIMNFGIEYKQLILYVNYYSGHKLILITKMPRKTKAR

SRLDKYYNLAKDQGYRSRAAFKLFQLNRKYNFLNNARTVVDLCAAPGGWMQVCAQIMPTS

STIIGLDLVHIKPIPGCKAFTQDITTPQCVQLLKKEIPQKADVFLHDGAPNVGASWAKDA

YNQNDLVLSALRLASQFLKKGGVFVTKVFRSTDYNSLMWVFNKFFSKVEATKPLASRFVS

AEIFVVCLDYLAPEYIDEKLFDSKHVFKDTETDMLQQQIQKEIVSRTQRHRSGYADDVHQ

TVYQMINFEEFLHAENPYPIFIEYAGIKMTEEAKQKYLSLAKPPQDYEILMEDIKVLGKR

EIIQLLKWRSKIKHFISKQKKEQEQLEQQEQQESAEYEDLDEAEGEGEDDEVNEEEAVEQ

DDLDKLVDKQKEALEQQYIEEQKKELKEQRKQKQKQAQQEKKLAGRGIGFTPQEEDQELF

QFSKHKNILKLGYVDVEDKQEEKLKKEKLAFNNQKQLDKNLELIYENKKQKKENALERIR

NKKQALESDQEEVDADELACKPIKKVKKQEDHLKVSDLKSKFFDKEEFQKLKEQLKKQKD

DVFDNPLKNQNITQLEKKKKEKTDREQPADKFKNDSDSDEQEEKVMDPKQLKNLQKQIQE

DLNQLDDNKDVGLLKRVDKKEMEYQRKLNAKINQDPLPGEDEKVKQFELNLPISDMEKRR

RKLKKIAAREEKKAKQASEKDPNHKELEIVSEKKIEDYDIDQLATNLALAKKMMRKKTRE

QIIENSFGRDKWEDADLPQWFVDDEERHVFKMEPITKEEFQQEKQRLYEINSRVPKKIME

AKIRKWKKAQKKLKTAAKKAQTVFDTDGINEKTKMQQVRRIYNKEKANIQKEQERKVIVA

RKGQASGKLKSSRKVLTVDKRLKKDKRAMKAKARVGKGKKRRQVSRRKGKKQKS

>A0EEP7

MTEEQGKQRFAYTAPKQILEEAAQIGEDNQTRKQNLDKVEEKFKYKQKNRQLSPERKDVF

NDQGGENGRTYAEIMIQQDLENSRSEIENKIKKTETLNKEEKKLVKQQVKVEQNVAQQIK

QERSEWEQESKDVSKKPQKWETPSRDGPQSSARASRWDNTNKLQATPGRAGTVFGETPTP

GHMEIGDTPYKYGETPTPNHGEKEHLWLVNHGFGGGMTPHTPGTVMRTPMTPGQLGNMTP

DRVYQFRLEKEMEERNKYMTDEELTSILPGPKDGYEILRAPENYKPLRSSLKKLLNAKDS

IESPVQYQIPESIRIEVSATPSHPTIGQLPAIKPEEYNLFSALLQPINEDELTPEQAKER

KIMALLLKIKNGTPQMRKSALRQITQSAREFGPAPLFNQILPLLMSPTLEDQERHLLVKV

IDRVLFKLDDLVRPYVHKILVVIQPLLIHEDYYARVEAREIISNLAKAAGLATMITTMRP

DIDHNDDYVRNTTARAFAIVASALGIPALLPFLKAVCQSRKSWQARHTGIKIVQQISIFM

GCAILPHLKSLVEIIQHGLKDEQQKVKTITALALAALAEASFPYGIEAFDNVLIPLWEGI

KTHKGKGLAAFLKAIGFIIPLMDVEHATEYVKAIVPILKNQFEIQEEEMKKIVLMVIKQC

IQCAGIEAVYVRDQIMPEFFKYYWSKRTATDRRNYRQMVETTCEIAAKVGAAEILERIVG

DLKDENESFRKMVVETIEKFINQLGVSDIDSKLENRLMDGVLWAFNEQQSEDTQTMLSGF

GSIINAFGSRSKPYFSQLGGVLQWRLSNKSPRVRQQAADLIGKIAVCMKNCQEEARLGRL

GQLLFECLGEEYPEVLGSILGGLKAIVNVIGMNKMSPPIKDLLPRLTPILKNRHEKVQAE

LH

>A0EIC2

MEDNQAQIAFDYVAFIKNIPHNYPYECMGFILFLIYIFMYFTGNTTNKKLALKFASTTYD

FFKTNFTAVGITNKENGPLLQSISSNSFGFMAQGRNNLNCLAVTIELKKRQDLLSMLLFS

FIWPERDTITIDIPIAATPQPICFALVKKRDAKSYKEANIDLKYLCEKLSIDKIDDNLTL

VTLGEGKEPLTTIFDSKLCQALKKYDKYIQNIHFTDQKTLLANPYHLRATLINIESLEDY

TEFIQLILQIVDKIANFKLSQSARQQNEEERAKFEEIKFKDEKQKKIEEAQKKKTEKLQK

EKQKLLSLPREQQIKLQEKEKRDEIKKKYAKRTMYM

>A0EYR3

MDDMMNNNPAGRFINNNPTLFNTSTLMSILIALVIIILLVMLFQSSSNGNNSSGNNPENR

LAYTNPLNATMRANPFVNTAQRTML

>A0EYR4

MSVLAEKHYLDRRNFKYLFLASYFNLNEYDAIAAEAKPFINYYLKNNFNNLDESCVLKYL

DYLNGLNIKNIVMEKSINVLQYVKPQFKFYCNKNNLDILMFDDKVYLQPNTPIYATNLFV

SNPKNFRVILYAEFAKVFNERNFVNNAETYCLMNGNMGYVFEDAYVDWCGVRLCHMPKIT

NSLYPYRLYLIGEPMAKHFIENNIDLANKQDYIYKNFHKGLPLFKNNFRIINSKKFVTKK

PNKLFDEMRSELDTHSSYIKLIQRDYIYDADFSEELFELLNDYMSQTALFKFIVKFNDSD

KNVFNKNSVYNEIVVDRYAVNRYRKLNIKIEPNTTFPLVLKDQPSFIMMRDDMIQIKGTL

NAFYVPKVKLFAILSNNTLFGSTELLHFSPSLIQYSHNTPPKRLQNESYVIDKKQKLYLT

KFIFGDSVPAYLLIRGDYESSFKSLKDFNNTWVQNTLFKLLIMPDFVERSLELIDYNLRS

NF

>A0EYR7

MATLTTTDLTNASRYATHQHRLKFINRWRNKFPHILIDYEIRPATNDDFYVPPTLANRAI

AVKLTFSRRGCESMTCYPFNETAPIDYNTPFGYTQTSETSVAYAQPACYNLDRAAATREG

GENEIQAPELRYTSGNKCILVDTLSKMYLNSPYLRTDEHLIQGVDDVPGFNVKPTDSDSL

FPEMFSGEFNQAYCRRFGRTLNDGGCSMQWWESLIGFVLGDTIYITFKLLANNIFSELRN

FDYTRPSPELPVKPVVDAQQVLNDWRSVRDTRVDLQFENSFDTYETLNDLRINAFTKLVY

TADQGFATLPFAREQLRFRTHTLTDAAAAQNSFDVTEDDLEFIISQFLEDHALIFGIWVS

FGFDNVVDAMKFMLKKINTTLIPAMKQMLLTTSKRITVKMLGETYKAAIVHQFNRVAIKT

ISAVAKALTKITIKAASVVGILLIILTLTDLVLALWDPFGYGNMFPRQFPDDLSNSFLTA

FFESMGETRDMLEFIPEYFDELVETDDVEIFDSLLYILDYISELEINSNGQMLNFDQSAV

IDDFDEVTLVGSALSSSALYTKLDFMQYTQRFNATLYQNDDATIDTILAGLFSISAILIL

VNNSQDYRHLILFFIFVLLALYLLIRTSFRFYVNLRAFVSTARPKWYQNLYT

>A0EYT2

MSFARYYEIETQLLPQCCKYLNNKLMLYHLYLKGVNVVEPVNVVKTNVKVNYEGFIQMYN

KIVVFDFKYLHQTNPNEINDYIDVEKKHLSGHDANMLKLLAQDRWAKSDFVRLYKILTQS

NVKNLINFACNTLWERGYENHYTLGQQLSIRITTKLIQSGLDFKHQTGQEEKQTLSRGWH

NAPFEKFVNSITSISDVIKRHRYYKKYIVLELSPGQKCKQLIEYFKNHFTIIENRNAFNL

CAIEIEDDKNSLLYMKKFANLIDEKIVNVLFVTDVEYYMKTNNYMFYLYNSLKLYYYCLT

NKFVFERKDYEIIFLINIIVSLEWHNNGHLNSFTLEKSIIYNPLELSTRRLNSIKRAATQ

SRTLSNDSEIKMDFIKGKRMKTGTHYGHRMIGL

>A0EYV3

MYKLFGIFLLMCALHEIAVAVAPGKPLLDWADRNYALVKINSDATAYEKVVTVETQLQIP

VSWNVYSGNAGDVAYILFDDTQVWQGNAATKRATILIDKGGLYKMIVKLCNSDGCTSSDS

VDVKVADTDGAHLEPLPYEWQENNKHFDNGGGDNKVVAAYFVEWGVYGRSFPADRVPAPN

LSHLLYGFVPICGGDGINDSLKTITGSFEALQKSCAGRDDFKVSIHDPWAALQKPQKGVS

AWNEPYKGNFGQLMAIKKANPNLKVLPSIGGWTLSDPFYFMHDAHKRDVFVDSVREFLLT

WKFFDGVDIDWEFPGGKGANPLLGDSQHDGHTYVVLLKELREMLDDLERETGRNFELTSA

ISAGDDKIAVVKYNDAQNYLDKIFLMNYDFKGAWSNVDLGHQTALYAPKWNVNEHYTTDF

AVNALLDQRVRPNKIVLGVAMYGRGWTGVLDYSDDNPFSGVAVGPVTGTWEDGVVDYRHI

QSRINEYKYRYDDTAKAAYVTKADSGDLISYDSVQSVMDKGQYVLDNNLGGLFAWEIDAD

NGDLLNAMHIGLGNHRSHHTHFELYSHIKNINSRCILP

>A0EYW2

MDLFRNFIGNIIQSMPHVSKVAYVSSHLKKYLAEMEDNENEKFNKKFKTILQKFIDQEIT

LDQICTIIDAADGIQLNKSQLNYFVNQMYLDCYLIDILQRYIDYNQLNDENVHYVAEFLV

LEINKALING

>A0EYX7

MATVPISVDQIFVDIFADCQRPDDAVLKDFNLVDCLILKNDVLQKKQIVKNVANFQRLLQ

TMRNETTKCLKSYCHNVHDHVIAPHDWYVQGNCFIVMVRPFIENKYYETVKHNINFNQFL

QSNKQDYGNECVKANDYYYWPNITISYFGWRLYLQMKFNIDIGDYVPLLGNCAIGNVSLF

DFEPDYFVNIEMSLVHKEKKLFVNGRTEFTDKHDALFDVTMLDGSQSVCKINDKLVFSNK

NFFNYIRDDINLTKCQTVEKYKGLIRVDLQSLRSFKPATDEHHKKIVTKERLKVNNVITA

SSENDDIVTHVESCVKLLNEYMTKIMVQHEIANKNVLQHYLTASKYVNFDYMIILIWRLI

AKSNNFLFFETDIKLYLELLCEKLYLSGSVEFAEVQKRCEPYTKLTPKVFTRFCAHWTVF

SQDNSLESLACYYAIHLMIYNKLSQNNENNEKDCWEYNYENVIHSGASAEIMCKGFFKKI

QTANACLVFNGKHYVGVKKDDDLFKLTEKCSAVVMSSIKFNNWKYLYFTDEGVYNLFIND

YHDSCPFILGNTLLGALTLKSEKTYLPESVINFMLDTGKIERDIFRIYHVAKLCRDVKML

KSNIAIVLSFDNCKACNAYEQQKLNDCFREIWNFNQHELITLGLYLNENKMSNLVNNLKC

GECKLKKAPKKCTCYNEIQIELKTLKIILIIELLSNNIAILELAWSMLYSSVLYTKILMD

SIYVTKSTDINLRNIKSNAMYFNTNKTKIVNYLYNYINNIDHASQLIDYLSNFKNFLQNL

QNNVENNDDSAFDENESDTSCSSNQTQNENANASDDDDDDDIVDRKKNSVADTSVSKHKN

YNHCGKMQIIDNFYEHYTMNTTFLNRWNIWWDKLIVARKNDDLNTWLIRFYTRVILSKIN

LEEYNHFYINNIVTGYLYFRTFTNFNYINSLLVIHYCASLGIPSDYEKMCLYITGKPGSG

KSSNTEILEHIITVHKHNADSYTLSKKETDEMEADKMISQLYVINEMKECNDSFFKTSAD

STKSNSVCRKYQGSQKYEANYKLMIINNKPLYISNYDKGVRNRFAIVNMKHEFVENYKFT

GSVYSHIKQQKFPMERSYYETLYKPVRLFLSHILMYKRSKRDGYVSYKNIIKNDAIHNYN

LMCLDVNNNTVHALLYILNVRITNDCKLIDESKIEKMIEMAAPYVETMIHDSMKLKRNAN

NTARVPQLCVDFKKKFNEYYRENDRAYCGIDLAWKKNDFNTTPPLFKCGNE

>A0EYY5

MNVSATNLVKYSLQFRKNENDIKNVNFSVQLTKCEIDSLTFLFSKYYDQSKYVIVKGLTF

FNEFNKCVDAVKHNFDSKQDNNEIKQLFSMFLKHEFMGQIPNFKKIMQFLQKYLLTIDSP

SISDINSTCNVCPVNQLACLNCKILYLSASISMFDINTQNGWDIFLRPMFGLPLFIYILM

KTDYDNNGIFNSDDLMTNAFATFFYNLLSDKSVKYINVKTVQGLVDECRRVTASFDVQQL

EFLLCMLRNKNTCDTPLFLPFKNFIIQLACKTKIKQAKINKIASVVFTGFYLRIYIEAAT

PRLINNQNGNNSALRKQYPFGGPGKTLTPYEMELRNVCRFLLPTYTNEQFENFINKLYGI

KQDLSIDQYIVTEKLIRQLVSKHNLDEDFAVLLNHNV

>A0EYZ4

MLNRIGKANKQNKNVSLSRINSSMLAFDTSGTTSASGWIKTVSLLTNLTDNTSNKFDNRH

LFYDICYKLIVKALSTANVHTLFTFQNIFDTIIGLEQSVFSKSIILNYIVKFLIVYSDGN

DVKSAINLKLLNYFLTKYNVIL

>A0F081

MKDEADDLPEVLPSGPSAFIPTEEILEEGSTHEVMSDVLLSEGRADHINMVMGLHPTYLA

CFQRTQDALLQLDGPLPLSWRHYIVILAAARHQCSYLVQHHSSAFLLAGGDESWLRGVHC

APPKIQHLQTLNKLLAHRPWIITQEHIQELVCPGAEARWSLAELIQAVVLMTHAHSLASF

VWGCGIHPEPEQTEDQTLQPCSPTDSCCSVRGKARNQQEWSEAVNEVKLLMERMMMVQQQ

GEEFTQEEMVTRFERERTESLLEPADVQRSVLPDCVSRFVVDADFTYQDFSPRGAQAPPT

MRAQDYSWEDHGFSLMNRLYGEMAQLLDEKFQVACALTYHTMAMHSHVDTSTLRKAIWNY

IQCIYGIRYDDYDYGEVNQLLERSLKVYVKTVACHPEKTTARMYFSFWRQFRHSEKVHVN

LLLMEARMQAALLYALRAITRYMT

>A0FJB4

MPNNNGKQQKKKKGNGQPVNQLCQMLGKIIAQQNQSRGKGPGKKNRKKNPEKPHFPLATE

DDVRHHFTPSERQLCLSSIQTAFNQGAGTCALSDSGRISYTVEFSLPTQHTVRLIRATAS

PSA

>A0JM87

MLTSEKGVVEEWLSEFKTLPEASISSYASGLKNKISLVSALYEVIQKPQSELLEPVCHQL

FEFYRSKEKRLGTFTLQFLPELIWCYLSMSSSRELQSCGCIEALLLSIYNLEIMDEQGHS

KILSFTIPSLSKPSVYHEPSSIGSMALTEGALSQHGLTRVVYSGPHPQREMLTAQNRFEV

LSFLLLCYNSVLNDMPALSLLSLCQMCSRLCVCGYPRQNARKYQGIKNRIPISSEFMVQL

LTGIYYSFYNGEWDAARRALDDVLYRAQLELLPEPLLLANAIKASLPQGALKGNKEGSRC

IQVEITPTSSRISRNAVTSMSIRGHRWKRHEEPDTNNDSSDLANIVIPEISVTSVSGERA

GNGERGRALTENDVQHMQAVQEISAEPRAEGKAVTEIRRQKSVRKLLEDGVASPGRVQC

>A0JMQ4

MPPKKRSSGTPQKKELKGSLKSRSPDSGDNAVLSPERHKDKDPEFVFLSEELQSTNSICD

HAWRIWEREIRSMDKTNMPYSNRQQWGACLFIAGMELEGINLTFTQFLKAVGLSVKQFIS

LVRKMDVNVDTISPKVNSAVTRLENKYDVTLALYQRFVKTCEKIFAEPDNAKRKELWESS

WTMFLLAKGTFLQMEDDLVISFQLLLCVLEFFAKRLSPSLLQSPYNSVVSSSTLSPPTRT

SRRNQGKSKPRPAEMDMQLLETLCKEGDCSVDEVKNVYQSTFCAFLDSVGLLGLQGLPPM

EALSKQYEELYHKSKDFDARLFLSDDETLSPNKIEVSKVEVTPRKNLFAEDIAIPVPQTP

IRAAMTSIQQLRGDLTSGSDQPSSNLLVYYKNCTVDPSGEIKKRVEELGEVFIQRFAQAV

GQHCEGLGRKRFYLGAQLYYKVMESMLKSEEKRLSVQNFSKLLNNAAFHTSLLACALEVV

IATYVGSSLKNGGFGRSSGASDSVESDLCFPWILSVFQLPAFDFYKVIESFIKAEPTLKH

DMVKHLEQCEHVIMESLAWRTDSPLFDLLKQSREEGPGEQAEPPATLNQPLHHNHTAADL

YLSPVRPCRQPPVMEAEPPTPGTRAPRSNSLSLFYKKLYRMAYLRLKMLFSNLLTSHPEM

EPIIWTLLQHTLQNEYELMRDRHLDQLIMSAMYAICKVKNVDLRFKTIVTAYKELPNTNQ

ETFKRVLIREGQYDSIIVFYNLVFMQKLKTNILQYSSPRPPPLSPIPHIPCSPYKNSPLR

VPGSNNVYVSPLKSSRVSPLVMTPRSRILISIGESFGSADKFQKINQMLSSSDWSLKRSL

DGGSAPKPLKRLRFDMDGQDEADGSKSSGESALIQKLAEMSSTRSRMQEQKLKEESDKDH

PEP

>A0JNT1

MDLLSGTYIFAVLLACVVFQSGAQEKNYTVREEMPENVLIGDLLKDLNLSLIPDKSLTTP

MQFKLVYKTGDVPLIRIEEGTGEIFTTGARIDREKLCAGIVLDARCFYEVEVAVLPDEIF

RLVKIRFLIEDINDNAPLFPTTVINISIPENSAINSRYSLPAAIDPDIGINGVQNYQLIK

SQNIFGLDVIETPEGDKMPQLIVQKELDREEKDTYVMKVKVEDGGFPQRSSTAILQVSVA

DTNDNHPIFIEKEIEVSIPENAPIGSSVTQLHATDADIGENARIHFYFSNLVSNIAKRLF

HLNTTTGLITVKEPLDREESPSHKLLVLATDGGSTPARATVLVNVTDINDNVPSIDIRYI

VNPTNGTVLLSENAPLNTKIALITVMDKDSEHNGRVTCFTDHEVPFRLRPVFSNQFLLET

AAFLDFESTREYAIKLLAADAGKPPLNQSSMLLIKVKDENDNAPVFTQSFISLSVPENNS

PGAQLTKISATDADSGQNAEISYMLGFDAPPEFNLDQRTGILTAVKKLDREKQEKYYFTV

LAQDNGIPPLMSNATVFVTVLDQNDNSPIFTHNEYNFYVPESLPKHGTVGLITVTDPDYG

ENSAVTLSILDVNDQFTIDPQSGVIRPNISFDRERQESYTFYVKAEDGGRVSRSSTARVT

INVVDVNDNKPIFIDPPSNYSFEWVLPSTNPGTVVFKVVAIDDDIGMNAEVLYSIVGGNT

KGLFMIEQTSGNITLKEKCMVSDLGLHRVIVKANDLGQPDSLFNVVNVNFFINESVPNAT

LIYELVRRSIDAPANQNTETTSASSPTTDYVKIMVAIVAGTITVVLVIFITAVVRCRQPP

HLKASQKNKQNSEWVTPNPENRQMIMMKKKKKKKKHPPKNLLLNFVTIEEAKPDDGENE

RNSVTLDLPIELEEQTMGKYNWGTTPTTFKPDSPDLARHYKSASPQPAFQIQPETPLNSK

HHIIQELPLDNTFVGCDSISKCSSSSSDPYSVSECSYPVTTFKAPVSVHIRPTMKEVVRS

HTPMKEATTVEIWTHPHPQSQRRVTFHLPEGSQESISDGGLGDHDAGSLPSTSHALPLGY

PQEEYFDHAAPNNRTEGDGNSDPESIPLHPSKTTASRSFHIHHTLLSN

>A0JSB4

MDVDTLREICLSFPGAFEDFPFGPETSVFKVRAAVAGGSRPDAKMFAASAMDPADWSVSL

KCEPALAEQLRAIHPEITGAWHMNKTHWNGVRLDGSLPDDMIRDMVEDSYDLVVATMSRK

QREQLEWAGLARGEGA

>A0JXT4

MQDGTMSAEDLENYETDMELQLYREYRDVVGLFSYVVETERRFYLANHVDLQARSADGEV

YFDLTLQDAWVWDVYRSARFVKSVRVITFKDVNVEELPRNEELALPKDVDLGN

>A0KF76

MKRVFNLLAMGLILFFWFGVLVSLIAPLPGKLNGFLPVCGVIVALMHWVQASMVKAACKP

YFAVSRLEFTQVLVFGVFGMVEIRTRLQAIVDAKQRGTDNKAG

>A0KF96

MKKHHLCLISAAVLTAGCTSYRHPEPVQAKDALRHAMTEQNKGGALTSVPKSVQSELLQL

NRPPQAISMPEPRLRIAAHDVDAVEFFGSLFKGSRYSVAVHPGVAGQISVELKDVTLTEV

LAVVGDMYGFDVQRKGNVFHVYPAGLRTETIPVNYLMMSRRGLSRTSVSTGGVASNDNNS

SNNNNFDNANNSTNNSTSNRSSNGSSNSDSNGTRIETDTSSDYWTDLRDTLQTLIGSGDG

RAVITSPQAGLVTIRAYPKELKAVREFLNQSESHLKRQVVLEARILEVALNEGYEQGVDW

SGLSASWDGNKGITGGGSLTPSTIANTPNQIFSALGGGAGFKISDGNFNVAVNLLKTQGD

VNTLSSPRVTATNNQKAVIKVGTDEYFVTNASTTTTTSGTSAPIVTPNVELTPFFSGIAL

DVTPQIDEEGKVLLHIHPSVIDTEEQKKTIDVGTADPLILPLAKSSIRESDTVVQANNGD

IIVIGGLMKTDKQEIVSKVPLLGDIPWVGEAFTNRRESTKKVELVILLKPTVVEKDTWQN

ELQRSSELLDKWYPPKG

>A0KFX1

MNGLPIRAPALGWLLISLLAAPLAAAPLTLQGETQRYRIDPDTLAISVNQIPVSAGQPAR

QVDQLQQGEQQASWHWPAQDARVEVKLDGDDLLLTLVSDSPRQLAWFSLPKNQTHLSLAF

GEGSRFSVRDSDWLGYVGDELAELNTHYDLKLPLWSQDAGKQTYSWILQTPYDNRLRFSR

QGGRLQLSASHEFSSFNQQAPMVVRLSVGDDWLAGARRYRAWLQSEGAWQSLADKVARIP

AGRRLIGASHVYLWGDALLDRQDVTAWPALQAKLRQKPEWLQGFNSEEREALAANAPEPW

QQALIMAGLNRLIADQLPLPAQQGPQAQSKVLALRQQWVKSQFGEQLVAQERWGQGLSLP

VIEALQQAGLRKLWLGTPQWTAALQQGYALTAAQRQGYLVGSYDSYDTAIAPGTNDSWLT

AQLPPEIGKRCAIFEANGEPKPGFGGEGVYLNPGCTLPFAKSRMSELAKASGINSLFLDV

DGTAMASNDYNPAHQQGSQAMIADRNARMSWVGERLGLVLGSEDGNALTSKPLLFAHGIQ

SWGFGWTDAAMRKQAKSPYYLGKWWPDEAPQVFFQPARLKPRYLKTVFNPADRLPLYQAV

FHDSVLNSHHWLLDNLKFPGIKEERALLGLLYNTPPLVNLSRSTLASRLPELVRLDSQFG

PLHEALWDKALVGFRWRDGVGRVQETRFSDGSRLVANFGHSSIMIDGHKLPGRQLLVALK

DKPVSVLKIQG

>A0KFZ6

MDYEFRRDPFGGYRARFSMGHEAIGQWLIDEVGKDEEKLDELFAIIDQLSSRTRVEYRLL

GGDYSLLLTHEEAEVKANVLNIEQDEDLDDLAYYDDEQLAMCGLEDFAQVLGSWRAFIRN

EDMG

>A0KGN9

MSMNIFGTKLQQGRHYMTLWPRRPELNSMFPENRVIKATEFALKVIPALAVLSVMLQFQF

GELHYWPSVMTSVLFLLSLPLQGYYWLGQRADTRLPPSLASWYREINGKMNEQGGRRQLV

AQPRYEELADTLNAAFKQLDKSFLYE

>A0KHD3

MQIFLITFGVFLAVVVAMAIGYIFQKKTISGSCGGLGSIGIEKECDCPEPCDNRKKKMAK

EEARRKMLAENRII

>A0KHQ5

MAAFFHSRPRWTANAGGGQTGFIQTSTNCHIRVTWQRHSVPCPRNRCFVMKVFKNLSPRQ

IARYIKSFHHGSFMVEAHGRFEFSGGEINLESLTCRQTLSLARQINLEVRGLRHTTLLLG

>A0KHU7

MGRSREARMVCCKTHPFYQVVSMILQSMLLSLAMLGQGSYDINEQQINQYLQSQVQVNKQ

LELPGIIKAHVQLEQSDVQIGRQSPDTARVYGKGKLKIALPDQTEYDARLNMTYEARPRY

DKAQSALFLDNMKLIEYKLEPEAAQQKFGFMLGMLLQSMEKRLETQPVYRLNDKDPNQAW

LKENLLGLELSPGKIHLLTKPQ

>A0KIA9

MMRLTSVALLLFAGAAVAGKERNLDFYNIEEPQLIELFARQQGSPGQSIRDFSGLFLGNP

YVANRLVGSSDTQEQLVVDFGGLDCFTYLDYVESLRRATSVHHFIENLMHTRYVNGEVNY

LTRKHFFSDWVHGDNRVRDVTHLVGGAVVTTAKQLNQKADGGHYIPGLGVTARDITYIPS

ASIDENTLSLLEEGDYIGIYTDLAGLDVTHTGIFIRGENGPVLRNASSLSSNMQVVDSPF

LEYVQNKPGILVYRAEAERGSS

>A0KJH2

MTIHDFEHKLLGLIDGMVATATDDELFAGGYLRGHISLAVARAELEGKTLVRDVKSYVLR

SLNEAILQGELSEQDEKLVQAMWIRLQQQAEA

>A0KKJ9

MAVEIKYVVVRGGVEKMTFASKKEADAYDKLLDTADELMHLLAGAPVRLEPDQQESLAFY

LAEQRDLLQNVLRGAKGQGSGKSEQQEEQSESDKPKDKSLKRVA

>A0KL18

MIKILIVEDDPAIAEIHRRFVQRLAGFEVLGVALTLFDAREQIEILKPDLVLLDVWLPDG

EGFALLRELRQAGASLDVILLTAAREAGALQEAMRLGVVDFILKPVVFERLRDTLDKYCQ

NRAALAALADIDQQAVDALLGTPLQQVAAGGLPKGIDGLTLQRVLAALGSQGASAEEIGG

RVGVSRTTARRYLEFLVGQQLASPELEYGTVGRPERRYRQP

>A0KL50

MMHITLIYAGLLGLLFLLLSFWVVKRRAQFKVMIGEGEAPEMRAAIRAHGNFAEYVPLTL

LLMALCELAGVGAFWLHAGGLALLVGRILHAIGIQIPKAPNLPRLVGTLLFWLALGLFSV

LALAQGLAAG

>A0KLT9

MAKPGATGVTRIINATGYSMKGLKSAWINEAAFRQELMLILLLMPLAFWIGDTLEQILLL

VCISWLVVIVEVLNSAVEAVVDRIGSEHHELSGRAKDLGSAAVFIALALNALVWGALVGR

NLLGWW

>A0KNI6

MNRFALARREAALCLLLTLLYFLAWYGTAYFIPAEIECWDMPLWFLLSCILMPLLFIVLC

GLMVDRLFVDIPLDSPPLDAKEPSHES

>A0KNT7

MSEQPVSSQKNNPLHGLTLEAIVTALVAHYGWEELGQQINIRCFTDNPSIKSSLKFLRKT

PWAREKVESLYLYVQRKQAKSKE

>A0KQJ9

MASSLAAVEEPQMNWRIAQTDRDAEVVMLVNPALDWDSFPTLAEALLQEWELVALERECS

ADRHSWLLEFEGSRFRLEYEHYSGCWLAVEQPADAEVLLWLARQHQG

>A0KQN3

MRQDKIFQEQCVDEIRQMAESKSLCELTSRWMSLSCEKKYSYHFRWAGRPIIQYPQDIVA

MQEIIFDVKPDLIIETGIAHGGSIIFSAAMLAQLDLFEAIESGSTIDPRHSKRKVIGIDI

DIREHNRVQIESHPLSSRIQMLQGSSVDAEVIKKVYEIAAGYEKVLLCLDSNHTHDHVLA

ELEAYAGLVSVGSYCVVFDTIVEDLPESLSSDRPWGPGNNPKTAVHSFLAKDTRFVVDEF

IENKLMITVAPDGYLKRIK

>A0KTQ0

MSNTHAQGTVAQTIDQKLTESFSPTHLEVLNESHRHHVPPNSETHFKVVVVSDEFDGLRL

LARHRLVNNCLANELANGVHALSIHTFTQSEWAKDVEVPKTPNCRG

>A0KUS2

MKCAIMQPTFLPWSGYFNLMAEVDVFVFLDDAQFQKSSWHNRNRIPLNNMPNWLTVPVRH

QHLDQTLLATRLDNKTHWREKMTRMLEQTYAKHAFNSELYCFINLISNDNHTSLAELNIA

LIEMMAQKLSIQCKTIRSSELDIPGVRTRRLLSILHEIKATEYLSPQGAREYLTADDFTG

QTDIQLSFQEFLPQVYPQRKTNHFIEKLSMLDVVMNIGWSNSKAYITQNYSGN

>A0L365

MQINKSLLLGLCSVICSGMANAAVEGFSYTDTNATVKTTEGSLINPQTDAMVYLSGGLDN

KLKLSVINTSGVTVFNNTTDFIRVSDRLSFSGREFYGKSITIPKLMDGLYSLKIETINSS

GTTIDQYTKVVEVDTEGPIIGDWYGNGTSNYGAETGSNEIWWLNTQNTGEDSKIWITDVK

GAVSGSVIVQKLNSDSIVLTRPMVIDQAAFKAVINAQTDLSNILPSNNASDPYSMQAQLY

DAAGNITRSTVKTFYYDNYGGAPSSPFAVFDPDANLAESVSSSFVGFVAYTPGMTVKTNP

VRLLYRVPVDNVRDAGVKGGLALANSVGATNVIINGEYGYFDMTAPFGYNNANQIKWVDF

GRWMAGTIQYNLSLSETASKSPVLTSATFLLNDVRMSITTGIMHHNSILPATMTDFELRV

QPRDYVQRASFRGYSCDIPAGESICSLTGLTYEMTKGGYGYLHDAIGVVSVGLPMGTLYA

NYSYGESTYNDAQYPQMTSFKVDTTTNTLTAFVYLPGAGSWFERLVMKAARIKYGTKYLN

PISSIRNGVNWEYVFNLNSLPTGNHSLELEMVENHGPTTVSPLQTFKVENATPTISITNS

GSAVFNEIVGLDDLVVSIADESSYTIDSVIISGGSANDTVYLATVSKGNNVYSLQYPRLF

PSLTSGEIYTLTVKATDVFGNQGVSSKSFTYQPPNIVNVGSLTTLAVSRDLASSADKPVS

EVVINDLRTVESQLASGPQDALFTLRTDAEISVQVLGQVVKPGQTVTLNPVATSGVVKFS

VYPAASGQKGKAQFLFEILEVKSNI

>A0L4I4

MKSGSGTFKWMVISVAALFLLSGCAAPQNKAQGGAMVGALGGGLAGSLMGNSKHKERNAL

IGAALGGLLGYSIGNEMDKADLLKLNNAYESTPSYQTTDWVNPDSGRKYEVTPKPAARTA

GRVCRDAEIKVWIDGRPETAIQRACRNPDGTWQMI

>A0L5B3

MKIARMFKFESAHRLPYHDGKCHRLHGHSYRLEMIFSGTVRQPDEKDPQSGFVADFGIVN

DLVKEQLIDRYLDHRDLNASVPGIPYTSAEYLSAWIVGWCMRELEGHPQLGDLKIERVRV

WETINAWAEADRQDAEGMNFHV

>A0L5L4

MATPALHQRTILVTRPQPEADATALLILQAGGTPIITPMLNIAPAEDSQPLQQAIAAIHS

FDGVLITSANAARAYLDALAAQAQPLSGTPPCFAVGPKTAELLRAGGATVCQPSSRFDGE

ALAEAINQWQGKGRHFLFPRAAIGRETLIDGLENDDHRVTMVAAYRALPAQQLSAQAHRY

LREKRVDAALFFSSRTATTFFELLISQALEPAEMVLAALSPVTAERMASLGFAAHVIAPQ

ATAESLLQALADHWNRPTL

>A0L6T7

MSDNAQAFNGWMHGWSEMQKQTWDTWSKTAWDMLSKGGTPQQAANPMRFWQESMKPWMSM

MGGEPVQQMPWLVQALSGQSDAFMKFSEQFTQAFSNQAGATSGPTQGWAAQVEQAIANLK

KMFDPQAFLGDTPSAAAMWGMPMEGFQQFFSALNLNPAGGLGGFNAQAMGTPQQAMRSQI

EKFLSMPALGYHREAQEQQQQGVRYWMEYQQAFFDYLVLMSQVGSKSLDELRNLLLDEPE

KLNVSTLKALYDLWVKCAEEAYGEVTASEEHALIHAKLINAMMQLKQHQQQVSAGLYKSL

NLPDRQELERAHMGVQQLKRKSFELADQLADLQKENQRMQQQLSELSTLRDQMDAMALEL

AALKAASAGAVAATAAPGAKPKPAAVVKKATTKRSSSQQGES

>A0L7I7

MNLAVLARVPQLGRVKTRLAAELGQQAALEAYGQLLAHVAQQLRHWPGAVTLWSTPCLEH

PLFDQFFPSCGRRLQPQGDLGVRLAHVAAWGVQQGDGVLLLGADGASVTPELLHQAAQAL

AEVDVVMAPAEDGGYILLGMKQVHSILFQGIDWGSPRVAQQTRQAASEAGLTLWQSAVQW

DVDTREDWLRFLRMTSG

>A0L7M6

MKSIAQRIALSLDVRENQVVAAINLLDEGATVPFIARYRKEVTGGLSDIHLRDLLGLLTQ

LRELEARREVVLKSIQEQEKLTPQLERAIQEAESKTVLEDLYLPYRPKRRTKAQIAREAG

LAPLAETLFKQPEHHPARLAANYLNPEQGITDVEAALEGAQQILIEQFTEEASLVGQARE

SLWESGLLHAKVVKGKELEGEKFADYFEFEQPVNRLPSHRILALLRGKEAGILRLSLSHR

QDAQAQKGAMPFGALLICRHFKLENHNRPADLWRQSCGVEAWKKRLKPSLDSDLLKRLTE

LAQAEAVRVFAANLKDLLLSAPAGMVPVMGLDPGLRTGVKVAVVDATGQVQTTATIYPHQ

PRNDWEGSIKALSLLIKKYKVPLIAIGNGTASRETDRLVAELNRRHPDLKVTAVVVSEAG

ASVYSASAYASQELPEMDVSLRGAVSIARRLQDPLAELVKIDPKSIGVGQYQHDVDPKQL

DQALDGVVEDAVNSVGVDVNTASIPLLQRVSGLNSTLAQNIVAFRDKNGPFANRKGLNKV

PRFGAKTFELAAGFLRIQNGDTPLDGSAVHPEAYPLVERIVRQTRTTLPQLMGHPEVLKQ

LTPKDFVDTQFGEPTIRDILKELEKPGRDPRPSFKTATFQEGVEDIKDLQPGMVLEGVVS

NVTNFGAFVDIGVHQDGLVHISAMSNQFVKDPHQVTRAGAVVQVKVMEIDLNRRRIALSM

RLEDQPGAEASQATAQSATPHKGGAPTKRPTAPPQAPPDNAMAAAFAKLKQGG

>A0L7Z2

MDKDLSHIKLPISAAGHHVRHYQGIVPAVLLEGLQEAVETTQVQDAHCDLKAQIERGKLH

VTGTVTATVSMNCSRCLVDFERLLEGDVERWYATGVDPNNGSMGELAVTDETVYLEDDLF

TLAPLADEELLLHLPMVPLCGEGCKGICAQCGANLNEGPCGCDDGPQDSPFAALKLLKLK

>A0LDW7

MESAAAAFIGMGLAAAGMAGSGIGLGYLFGKTIESIARQPGAEAQMTKYMWIGAAFVEAV

ALYGLVIAFIIMSKG

>A0LFQ8

MSTVRNVLIGMLSDSLSRLLPEASEHLDPTCIELDIPKLPEHGDYATNVALALTRRLKRN

PREIAGKIVGGLNDPDNLLQKVEIAGPGFINFFFHPRAWHGILTDILADPRNYGRLDLGE

GHRVQVEFVSANPTGPLHIGHGRGAATGDALANILEACGYAVEREYYINDAGNQMDTLGR

SLYFRYQEALGEPVEFPDAHYRGEYMKDLARDFLRDNGDRYLRAPLDEVLPLFTRFAADR

ILEGIKDDLGEFGVSFDQWFSERSLHAEDAIRRTVEDLNRRGFIYESEGAVWFKSTAFGD

EKDRVVIRANGVSTYFAADLAYHRNKYDRGYDKVVDIWGADHHGYVERMLAGVEALGRNR

EDLRIVLVQLVNLLRAGKPVAMSTRAGEFVTLREVVDEVGKDAARFIFLTRRSDSPLDFD

LDVAKARTNDNPVFYVQYAHARLCSIFQVAAERGLVCDWANGKDIPDLGLLTLAQELQII

KFLGEYPAILANCSRSMEPHFIPYYLHELVSLFHSYYNQNRVIGDDPELTRARLFMAAAI

REVIRNALELLGVSAPEKM

>A0LH15

MRKATLALGVFVLSAFLLIGHSTLEAQNVLPDPGTLNQMENVAPGTTMFFNVTGKTTGGS

LWGTDYYTTDSSLAMAAVHSGAIRNGQTGVVKVIFYPGRAQYAGSTRYGVTSSSWGSYEL

SFAVEAAEGVKPVAGNAVMPNPGTLKNVPGAAPGQVMLFEVTGTGSGGSVWGSKMYTTDS

DLGMVAVHAGVLKAGETGVVKVTFRPGQTKYVGSTKNGVKSSNWGSYDLSFEIERVK

>A0LHK8

MRSIRVGLIGWGTVGCGVIQVLRENEADIRNRLGVPLELRRVADLDIDRPRPVSVPRDLL

TNRIDDILNDPDIDIVVELIGGLDAAREVIRRAIDSGKHVVTANKALLAHSGNELFRVAA

ERGRAIGFEASVAGGIPLIKALREGLAGNRIQTIFGILNGTANYILTRMAEGGLSFEDAL

GEAQQMGYAEANPALDVEGIDTAHKLAIASALSFGTPIRFDEVYTEGISHIDPDDIQFGE

EFGYSLKLLAIARHMDGRIETRVHPTLIPRHHVLASVKGAYNAVHVQGNAVGNIMLYGMG

AGMMPTGSAVVGDLIDLARDILGSTPGRVPALAFQPDRLAEIAIKPIADVSTCYYFRFSA

LDQPGVLSKVSGILGKFHISIAAVIQKGRRKASSVPIVMLTHEALESNVRAALAEIDQLD

IVTAPTQIIRIENQETNNFF

>A0LJ19

MKEKSLRRKDQIIPPKGKLGVLIPGVGGAVSTTFIAGVELVRRGLAEPIGSLTQLGTIRL

GKRFEHRAPKIKEFVPLTDPGQLVFGGWDLYEANGFEAAMYARVLSRDHLESIKDYLTGI

VPMKAVFDRRFVRNLDGTHIKKGNSLRKHVEALKEDIGQFKAANGLERCIMIWCGSTEIH

LKQEAIHQSIDALQRAIDANDSRIPPSMLYAVAALECGVPFINGAPNLTLDIPAMLELAE

QANVPIAGKDFKTGQTLMKTILAPGLKSRMLGLEGWYSTNILGNRDGLVLDDKDSFKTKE

ESKLSVLEYILQPHLYPTLYKNYYHKVTINYYPPRGDNKEGWDCIDIFGWLGYPMQIKVD

FLCRDSILAAPLILDLVLLTDLAQRVGMNGIQEWLSFYFKSPMHKESLYPEHDIFIQSMK

LKNTLRYLMGEEQITHFGLDYYLNGEER

>A0LJ38

MPGKVTGKRNWTYGSNSVIATLFFLGTLIFIVLIAEKHAWRLDLTEAGSYTLSEPTLNVL

KTVDQPVRIKGFFQTASPEEGKAQDLLDMYRFASKNITFEFIDPDRRPEVAKSYEVRTYG

TLVLEGYGRKQVAQNPDEEGITNALLKLSRNEQKKILFLIGHAEHPLASTDKDGFSSLKA

SLEKENYQAEELNLLQQAEVPKDAAVVIVAGPQKPLLPQEIDTLKQFVERGGRLMVLLDP

FFDGGLKDFLAGYGIKLGDDIVVDKLSRVFGGSYLMPVVVEYGQHRIAENFGAATFYPEA

RSVQPIKPAPQGIEVEILASTSPQAWAERNIQVVRQGQAAFDENEDAPGPIPLAVIAEID

VTAFKSGAQGEAPPKAPGKPAAKARDKGPEKVGYLLATGDSDFACNTYFGLSGNGDLFLN

MVNFMAQEENLITVKAREKGGQMMMLSQNQARGILLTVMVLVPLLVLLSGLAVYRVRRSQ

R

>A0LK78

MDVDDKAFEPKILGFLCNWCSYAGADLAGVSRLQYPANIRIIRVMCSGGISPHHLLHAFQ

KGADGVLIGGCHIGDCHYLKGNYMTMKRVKFLEGLLQFAGYDPRRLRLEWISAAEGLRFA

EVVREFTDQIRSLGPAPTFEAVPGMDAHPSEAFSGRGEFMA

>A0LKY6

MGSRNCCRMLFLTLFFAGIFLSGSVAMAIEEAMYKVLEKGKNFELRQYEPHVVAETIVEG

NFSEVGNEGFRRLFGYISGKNRSRRSISMTAPVSQEAESERIPMTAPVNQEVEGNKWRIT

FLMPSGYALETLPAPIDPRVSLREVPGRLMAAIKYSGTWSRERYEAKKALLEKAIRKRGL

KPVGEPIFARYNAPFTPWLLRRNEVVIPVDMPK

>A0LLN5

MKAGVGYSNGENSYSCGKAAAEGAVQTGGIRRPGLVFAFCHGNHDHRAFLGGLRSVVGDS

VPVVGGSSIGVITNNDLSYERFPTGVAVLEAGGINCRVGSAGDVNLDERAAGKKLAERFA

LIEKDRLFLLFYDSIKAPSDGRGPPLLNASTPLIEGVASGLDSRIPIIGAGLLGDYGFGP

TQQFCGSHVSSQSAVGVSLDVDFHVRIMHGCTPLDGVYRSITKMEGSTIYELDGRPIVEV

IDELYGNTEWRKSYPVALLTLGQNHGPRFGAYREDRYVNRLITGVLPDGRGVNIFEPDFE

CGSEIQFMLRDSGKMIKSARKNSSGLMRHITEEAGRKPLFGLYIDCAGRSGGCSNTASEE

AAEVQDVFNRYGVPLLGFYSGVEIAPLLRRSRGLDWTGVLLVLTGE

>A0LM55

MYLSLLSLDRLHRGTMRLLSDIYLLHKGIMSGFTRCGDGLRVLFRVEPENDDRIVRIMVQ

SDGSPSWELFTERHPCVIDMRTKVFSPALRAGHSYRFRLRANPAVKRNGKRYGLIRDETL

EEWLRRKEPALGLQFRSVLALDEGYVTGHKEGSGHPQRINIKTARFEGILTVSEPHLVQN

ALCCGIGPAKAFGCGLLSLARV

>A0LME6

MKPAELTAFATARVESRAHPTRGGSVPRQCVLDASGSGAWEKVGGISTIARMLYHLDKLG

IEEVVVLLSSEHGLKGLERWTGGLRLRQAGKDAHLSLAGNLLSMARLDRRFLYLDAAHLV

DPRLIKALAAASKTTLTFMDPRDARNGTVRAGLLHVDDLPAWAEEGDAALARRSKPLFPS

SIDPYSPEIRGPLTPYFLEVRSREEALEATRLLIRSQQKHVMDLPAEFIDPPFENALTFW

LCRTSISPDMVTLIGGAVAFFVAWLFWHGYFVAGALLTFVVEILDGVDGKLARTKLQFSR

FGRHEDVFDYLCENSWYVALGVGLSASTPGNWPFFAAALLIFSDTVDNVLYTLAGKWHGK

SIDLYRPFDAAFRRIAGRRNIYGFMFIVGFLAGFPLQTFILAAVWAAVTAAIHLVRLIQF

GRAVARGCG

>A0LSB1

MFESVLVANRGEIARRVIRTVQRMGLRAVAVYSEADAELPFVAEADEAVLLGPPPPAASY

LDVAKVLEAARQSGAEAIHPGYGFLSENAGFAQAVLDAGLVWIGPAPEAIAAMGDKVAAR

NRVAAAGVPVAPGTAEPLTDVEEAVRAAASIGYPLMVKAAGGGGGIGMKVAWDDAGLRSA

FATAQSAAQRFFASPAILLERYVPGARHVEVQILGLPDGRVVALGERDCSVQRRHQKVVE

ETPCPVLPEPVRRRMFMAAVRAGEAVDYRGAGTVEFLLDPASLRDDDPEAGVFYFLEMNT

RLQVEHPITEMVTGIDLVEQQILVAAGKPPSFNPDDIRRRGHAIEFRVYAEDPQRFFPNP

GTITAWAEPAGEGIRVDAGYRAGNTVTQYYDPLLAKLCVHGVDRAAALDRARRAVAAFRI

EGPKNNVPFFAELLENEEFCSGKYDTGLIERMRS

>A0LTL0

MPLSEHEQRVLEEIERALSADDPKFAATVRSIDPRIYRRRRYLRSLCAGVLGILLVPVGL

IIRQTVVIALGAVVAMAALVYAGAIWRRGRRRLGRSVPRSPKPRGRFMHRLEERWNRRRE

NPT

>A0LUN1

MNASVHIIEVTEFHLLLDGDSRVITARLHYDPADPYVITVHFDTGVTWVLGRDLLADGLH

MEIGDGDVHLRPEGDQLFLTLRSPSGEALLAASRSAIAGFMADTERVVPRGTEHLRVDLD

KELARLLP

>A0M722

MFQLGKTLVSEEIIKNDFLCNLSACKGACCIDGEAGAPVEPEERAIMEEIYPKVKPYLRK

EGIEAIEKQGVYITRENGEIETPLIDNADCAYVTFDEKGTALCGIEEAYNQGDISWKKPV

SCHLYPVRIQEYSSFSAVNYHRWEICDDACSLGAELQVPIYKFVKEALIRKYGEDWYKEL

EVVAKGITQK

>A0MBM6

SYQWQTATNPSVFAKLSDPPPQVSVPFMSPATAYQWFYDGYPTFGEHKQATNLQYGQCPN

NMMGHFAIRTVSESTTGKNVHVRVYMRIKHVRAWVPRPLRSQAYMVKNYPTYSQTITNTA

TDRASITTTDYEGGVPASPQRTSGKFGQQSGAVYVGNFRVVNRHLATHNDWANLVWESSS

RDLLVSS

>A0MD80

MAVNRDKVDLWKSDVARSVDFYNDWFMTFAPKAFRDTRVETTKQVEQALQWTENLTNIQP

ETLQNYPSVLPMLRMTTCPPIARDRLVGLAGISPNLVKSMEVDNRVPPKMKPRELHEHLE

KIGNIIEKMADPDIFVWKERGDKGTREEVHRASTIVADRLCGAVADPIIRNAQEQRQLAA

IKSWLEARGYRQLASGEAEDWLSMPKGTFSFRFNIPVTMDGGKQVNIPVDAVIMRKDAKD

GDFPALFEAKSAGDFTNTNKRRKEEAIKIQQLRKTYGHKITFDLFLCGYFDSGYLGYEAA

EGIDWVWEHRIDDLAQFGF

>A0MEW6

MTGNADSGKIATEKDPRKLVFTKLEVSPVFRESPVKELPLFPPISREHSEAKDKTDEEGI

TSRKHKGCRCKQSKCLKLYCDCFASGVVCTDCDCVDCHNNSEKCDAREAAMVNVLGRNPN

AFSEKALGSLTDNQCKAAPDTKPGLLSRGCKCKRTRCLKKYCECFQANLLCSDNCKCINC

KNVSEAFQPPAFSAHNSPQVYRRRRDRELTEWNSCPAPLFSIPDNSIQNALGSPMSCSPK

LPYRKKRSLMGYTSTLLPDLGDLCSLLVAASESATTTAEDQNRIFTKPDDKEAIELSSES

ESRNVEEEIQSRGRLIELIDVQYNGEEDSQCKTKTSVNETDIYMEQERAVLETFRDCLQK

FIKSRLESG

>A0MZ58

MAAVSVFPGVRLLSIGDANGEIQRHAEQRELRLEVRAGDIALFNAENVCVFKCTITRDTE

CSRVGKQSFIVTLGCNSVLVQFATPADFCSFYNIIKSCRCPDSEKSVFSERTEESSAVQY

FQFYGYLSQQQNMMQDYVRTGTYQRAILQNHTDFKDKVVLDVGCGSGILSFFAVQAGARK

VYAVEASSMAQHAELLVKSNNLTNRIVVIPGKVEEISLAEQVDMIISEPMGYMLFNERML

ESYLHAKKFLKPNGNMFPTIGDVHLAPFTDEQLYMEQFTKANFWYQPSFHGVDLSALRGA

AVDEYFKQPIVDTFDIRILMAKSVKYTVNFLDAKEADLHRIEIPFSFHMLHSGLVHGLAF

WFDVAFIGSIMTVWLSTAPTEPLTHWYQVRCLLQSPLFTKAGDTLTGTALLIANKRQSYD

ISIVAQVDQTGSKSSNLLDLKNPFFRYTGSTPSPPPGSHYSSPSENMWNTGGAYTMNTGM

GMGGMPTAYDLSSVIGGSSGISHSNLIPLANTGIVNHTHSRMGSIMSTGIVQGSAGGQAA

SNTSSHYPINNQFTMGGPAISMASPMSITTNTMHYGS

>A0NHX0

MVESINRKIDLVVQATSYMGLGEYGEIMIGDEGFEFFSDRDPRKFIQIPWKEVNLVVASL

IFKGKWIPRYAIQTKHNGMFKFSSKHPKEVLRMVRKYIDPDHIVRSLTFFQVMRRNFKWL

FGRIGKILKIKR

>A0NII2

MKQLITVKQNSTKELIIKKSRFIADIYPLKEEQEAKKIIENVRKKNPNANHVVFAYTVGL

NREIQRMSDNGEPVGTAGKPVLDAITKNNLINVLITVTRYFGGIKLGAGGLIRAYSQSAS

QTIENAQLATLVNYDRLQLIFDYSLIDKLKYFIEKQKAVVMETNYQAKVQAVILVETSDS

KNFQKRLIDLFSGRIIVKKINEELRPSIIENE

>A0NMJ6

MVYLTKRQVAGGSSGFGYPFGMWRLSKVRSLAGAAALLLASLGPALAQEQVDVALVLAVD

VSRSMSPEELQIQRRGYAAAIASPEVVRAIGYGAHGRIALMMFEWANESHAREIVGWSVI

ENAEDAEAFADKVLADNTYGQRRTSISGAIWHASALLADAPFKADRRVIDISGDGPNNQG

APVVQARDAAVEAGLVINGLPLMTTGGMGFQFNIPDLDVYYQRCVIGGPASFVIPVNDWE

QFPEAVRRKLILEIGGVKPPEPQVIPAQFTFEEPYDCLVGEKIWRRMREQYFWDQ

>A0NNH1

MVNRVSVYLQENGPERQPARPHSKSRPETQPAPFLAPQWRKLMSLFDEDLPNKSPPGTVA

VGEDLSRLSETELEERIEALRGEINRTQKELEQRSTIRDAANSIFQK

>A0NP11

MQETKSAPYGEKCISAVQETMSMSTFDKREQGFENKFVHDEELRFKATARRNKLLGLWAA

GLLGFEGEKADEYAKEVVRADFEEPGEEDVFRKLREDFDANNVEQSDHQIRRTMDELMHT

AIDQLQKEG

>A0NQ44

MKTDRDGGKTMRRRRVRARSIGFIGAAALAGILLASSAHAGLRVCNGSVDLVNVALGYET

DEGLRTEGWWTITANACSQVLQEPLKKARYYLHVADGFGESRLGGDITLCIREEKFVLYD

GDQCWQRGLIEADFFQVETEGKQDWTVLLSYD

>A0NQ51

MRFGRVFQEVSRMRALFLFLSIFAATLWISLSGSAGPAMAQEPQRNLPVEDLVIQSGDKE

HRFEAEVAATDKQRSMGLMFRKEMPEKRGMLFLFEGEGDRYFWMKNTPLPLDIIFIDARG

GIVSIADNTTPFSEDVIPSIGPAKFVFEINAGLAEKLGISAGDRVISPSMGLE

>A0NT16

MKTTYISTITLADASRRQIQNQASLVQRLSIEVSSGRKYDVGLDLGTKTGESVSLRSEFH

FLNGITDTNALTASRLDVSQAAMGDVLKDAQDFLSTLVALRENSGTADIVKADAVGNLEL

LTSRLNTQLNGNFVFAGINTDQEPFVDYSDPGAPNKLAADAAFLTEFGIAQTNPGVSGIT

PAAMSTYLNGAFDLMFQDPAWGTSWSSATDSVMVSRISASETVSSSVSANEQAFRDLAAA

YTMMSDLGNEDLDPETLKVVVNKAIEKLGTGIAEITSQMGQLGNVQEQVKLASERLKVQT

DILNRRINDLENVDPEETAVRLNTALTQLETTYAVTSRMQRLSILNYL

>A0NTV6

MRFMKSPCINICQIDAKSGLCTGCLRTLDEIASWAGYTDAKRSQILAELPNRTTQHPEAG

ERG

>A0NUM6

MVRLMDETGFSENGGGQTPGLMAGQGAAKPAADDGAYRVLARKYRPKTFEDLVGQEPMVQ

TLENAFETGRIAQAWMLTGVRGVGKTTTARILARGLNYEVPGVADRPTVKLTQEGTHCKA

IMEGRHVDVIEMDAASHTGINDIREIIDAARYRPATARYKVYIIDEVHMLSNAAFNGLLK

TLEEPPEHVKFIFATTEIRKVPITVLSRCQRFDLRRIDQSKLIGLLRRISDAEGIQISDE

ALMLIARAGEGSARDSLSLLDQAMAHGAGAIEAEDLRQMLGLADRARVIDLFGHIMAGRI

EDALGELQAQYEVGADPAIVLTDLADFTHLVTRMKVAPKSADEASVTEAERARGREFAEQ

ISVRLLSRAWQILLKGVQEVQAASKPLAAADMVLVRLAYAADLPDPGDLMAQIKNGQNPF

GGAGLSGGGAAPSGGGGGPSAMAVGMNYASGNSGSGPTMQASGRPQLSAIQGGRPAPQTA

PQAAPEPQAQPAYNLKNLYDCAALASEKGDIPFKVKIQRLMRLVKFEPGKIEIQPTDDAP

ADIAGEFGRKLTEWTGQRWFVVVSRNQGRPTIHEEQEANQQQLLSDAKSHPTVAALLAQF

PGARVVDVKVQVTEEDAAALADPMVSDPLLSEALGDDEDD

>A0NVS7

MKTMSKRLVAAALTGTLLLPTFASAEAGSRHGWRDQRGGHHQSYRHHNQNHKRNKNNDNL

GAAVAAGVIGLAAGAILLGATRQPSYAGPPPVNHYPPAPYPAPYPGQVHTAIGYQPWSPA

WYQYCSSKYRSFNPSTGTYTTYQGEQRFCQ

>A0NYI2

MLGKMFKSLFGSSGDGGKSASKATTVDYDGYLIVAEPQQANGQWQVCGRIEKQVGDTTKV

HTFIRADTLPDEEGAKNEMIRKAKMMIDQVGDSIFD

>A0P027

MRYALYFAADADDRLMQLGNTWLGRDPYTGAALPQPELSGMTPARFQELTTDPRRYGFHG

TLKAPFSLAVDQTEAGLLEACATFAAEIAPYEIDALGVNRLGRFLALTPEAEEPALRAFA

SLCVRRFEPFRAPLSDADLERRRKSGLSEIHDTYLVRWGYPYIFDEFRFHLTLSNKLEDD

TDMERLTNAARDHFSDVTGRKRLCRHVALYTEAERGAPFEVHTVFELTGNTAPADALSQS

GHLSQEETA

>A0P2I4

MPVLPSTVKTLTASAGRRLLCVATLLLAGAGLAPAALAGDTAELQLLGFSRHGDFFAFEQ

YGVQDGSGFPYSEIFVVDVIGDTWVPPSPFRLRKDVDTGLGEAADDALSETRAENRLAAQ

PLLQEKAIAGKGQTIGFNPRTELTSDPHKMLVAPRVTFLSGDDPIDLTLSEYPLPNAQCQ

SYGAETKGFRLTMIHNGVTRILNEDNSLPESRRCPLAYRIERIVTHFPDEAPPVFAILIQ

MDSLGFEGPDRRYLAITGRL

>A0P2X5

MVNLISVTIFLCTEERIGMKTSSDFEKRLLGYGLMTAEILYRLPDHPKLLQSFLWQTEDL

APKFPELTRFLVFWEREIEGRIHSVRVAHQTLISPVDFRYADGEIVVH

>A0P3R9

MQFHLNGFRTGDPVVQEPNPDAAFLADTDSLPEDVDVLIVGCGPAGLTLAAQLAAFPEIR

TRIVEQKAGPLQLGQADGVACRTMEMFEAFGFAERLEKEAYWVNETTFWKPDPASPDTIA

RSGRVQDVEDGLSEFPHVILNQARVHDFYLEIMRNSPNRLEPNYSRRLVNLEVDHEAGQE

RPVTVLLERTDPGHEGETETVRAKYVVGCDGARSVVRKALGRELKGDSANQAWGVMDVLA

VTDFPDIRFKALVQSAREGSILIIPREGGYLVRIYVELDKLRPDERVANRNITVEHLIAA

AGRILHPYSIDVKEVAWWSVYEIGQRLCDKFDDVPAEDASSRLPHVFIAGDACHTHSPKA

GQGMNVSMQDTFNLGWKLAHVLKGLSEPDLLASYSAERQAIAQNLIDFDREWAKLVSAPV

KSDSNPDGVDPAEVQKYFVQHGRYTAGTASRYLPSVLTGSGAHQHLAEGFQIGMRFHSAP

VVRLADAKPLQLGHVVKADGRWRIFAFADRSYPAVETSAMRRLCDFLEKDPSSPVLRHTA

EGEDIDAVIDLRAIFQQGHRDLALADMPSLLLPAKGRYGLADYEKMFCPDLKSGCDIFDL

RGIDRDKGCVVIVRPDQYVAEVLPLDAFEALSDYFNRILKRRH

>A0PB11

MALIFSVAGGAYAQESPLTEQDKALIEKGKQIAQKAQKMEMPSLLQNQHMDEAQAEAKAF

FKQLQTTNPTLKEMHRKQAEKGIYSDHRILVFASLSLGEQGLDDVLTAVSGQPDSVIVFR

GIPEGMNLGQGVKAIQALAAKKDPVPNIIINPTLFKTYNITAVPTIVMLEDEPLPGEQPN

VVAQVSGLSDPVWLAREVDNGEKGDLGVKGPVEKISEPDLIDVAKKRLANIDWEEKKKQA

IERFWTKQNFNELPRAPKSRTREIDPSVMITSDISTPDGTVFAHAGDVINPLCDPKEVCK

PGTRPFTQAVVVFDPLDKKQMELLAKKLPEIKQEPGVQRITYIATEFDKDKGWDSYKSVT

DNFDAPVYLLTPDLITRFELEHTPSVITARGKKFVVRELAEEGGE

>A0PC13

MAQPAKLQPIRTTAQSGAKQPQLKATKPSPEEGKKASPIVVQNEVCSSPIEDPSVVLSAA

KERPRNRAIPSESSSLLLEVRERVAAHRASKSRMKTVPLDRQDTAPVIHAERSIDGSSVI

YVSSSESSRASSPAPDITMKSQVKGKVANSVSGKKTPKVTTSKGKNKKDQPKMRPPEYAQ

YIQTEAAAGNLPKRPLTGFKFLDGYQIFYTGGDMRNASETTRARMELIVRYGGTLIPTYD

PNVVTHIVTDAPETTTVNALGLKRLRDIPDRIPTVKWSWVTWGIGKAASCTKDELYEKLR

DHLWQHAAFSDRLEAGMKATKSFIRAKSKGKGKEKARLVEEGSSPVSDSTQEPVAADEYE

SQVSANDVLSVAIPTEARPGGLPSPPASQQLGPAFSEASSSRAKLAHDPLAEFYDQAKAE

QMKEKQRVVSEAESDEEDVIPEIQPRKRGWTCDNPEAQITDCPNQDVIDKLTELMKLHEA

KPSEDDRWRVFAYSKAIRALRSYPRRIRSLEEASKIRGIGSKTAQKIVEILETGKLRRIE

YERTGDVVAAALFQGIYGVGRSTAYKWYNAGCRTLDDIRQGKGGVKLSAVQEIGLKYYDD

INSRMPREEAKAIYDIIKPIALSLDPKLFVEIMGSYRRGKATCGDIDILITRPTDDGKTH

AGVLGRLIQELHAVGVLTEDLALPEEIDDLEGVYRGLCRLPNVPNSKRRRIDFLTVPYHS

RGAALIYYTGDDIFNRAMRLKANVMGYSLNQRGLFGNVVRDPRDRRVKLNRGQIIASETE

EEIFRILGVPWQEPHERVRG

>A0PJ19

MDRLFREHQKFSKANTREETKEMRIYKDDSVMELAYSEINVGSTPTSPRMALSDYFSAVS

CTFDEEVRPPELQLFIYGDIHFHDRYEKDVELDLLVWQLLSPNQDSRALCVNILRMNATI

AMGDAFIRDGSYNFCQNCQETTSEQDLAALRFMSRLAKIIIKNSLTKDDVINAQRSLISY

YFGRVFKSVTLTWDSKCNLPSVHGYSTSETSLDHYIRMKIDLFKALSHNNLVYGGNYQLV

YQALFYYYVVTNGRYFSGFSSRREAIKSYTIPNDCPVVCNSIPRKPNLSLMFIRAILVIM

LIKDYSEIKETPIYQQQLELEDPARNACLVTDSGIRTELQNEPVTVPVTLPTLPTFSSTK

N

>A0PJS3

MESDILDILEQLGYDGPLAEEACLLAECGRGFSSSEYVNLLTWLTKQLTQFTETHTQDEI

ITADPLDVSRLLKDCCCPYEGLASRLANGDVKDTRDHLKIILFVSSELQSAQLLLSKTLR

DAEEREMRSCSPLQDLSVICHTLTLPDPAGRDPTDTFTDIQTQVNVLLEKLPETHIGAPA

LQRSISAEQWEELEKINSTLSAEYECRRRMLIKRLDVTVQSFSWSDRAKVKIDQMARAYQ

PKRHSLSVRSSVSLAHLLAARRDICNMVKTSSGSSRQNTSCAVNRILMGRVPDRGGRPSE

IQAPAPEMPTWRKRSDGGGGRGAGYRGGGGWQTGGGDGWRTGRWSRGGGRGGGHYYH

>A0PKE9

MNGGEPRSEQAGSALAAIRARQAELARQHDVLGEADRALVEALTRAHTVMRDSVRRLDAI

GAEIDGAVAGQDSLALDTPLGAREFQNFLLAKQREIATIVATAHELDRTKSAVLANLRAH

YGESVG

>A0PKF2

MTDANPAFDTVHPSGHILVRSCRGGYMHSVALSEGAMETDAAALAEGILLTADVSCLKAL

LEVREEIVAAGHTPSAEVPTNRDLDVAIERLLAHQLRPRPADRLEQPGFGGIGIGCDVGG

WFDGIGAKQFPRAA

>A0PKF4

MVSRPGGRAELSDKDLVESVLRELSEAADKWEALVAQAENVTYSVDLGCVHAVANSDGRL

LKLTLHPGVMTGYSHAELADRLNVALAALREEAEAENEARYGGPLQ

>A0PKY4

MTDERGEFVPWAGGLAVAAFVAAVVGVGDAVLSIGLIRVHPLLAVGLNIVAVGGLAPSLW

GWRNTPVLRWFVLGAGVGVAVAWLALIGLAGLGSR

>A0PNI0

MSFIETVSRIGMAIDGMGVAVIMLGAVVSSGLFVAHLPRRPDRAYREFRQNLGRSILIGL

GLLVAGDIINTILVTPNGESVAALAGVVAIRTFLSISLTVEMTGRWPWRQPTVSPETTAA

GGQLDAAADS

>A0PSE0

MSAGWIIPFIILGGALQTCGAAMNGQLYKHMINPWLASAISFALITIFFVGAFLIMPNPL

PTTKDIASMPWWAVVGGLVGAVQVYAGLTLVNKVGAGTFIGVTVTAALIMSLLIDHFGWL

RVDAHPLTLWRALGGAFLVCGVVLVAKF

>A0PXA9

MQITKDMTIGEIVRNFPSSIEILMSFGMGCVGCPSAQGESLEQAAMVHGMDIEKLLEALN

KAI

>A0PYS0

MINYVILGVSAILFILYILKIRKEQFTTKKMVTIAMFSAISFVLYLIEFIKFPQGGGITL

FSMLPTMLLSILYGNTLGLTSGILFGLLKLLNGATIVNPAQFLLDYIFATMALGLAGTFG

NDNKIKIGLGSLMAVVLSVFVNVLSGAVYFGQYAPKGMNVWLYSFMYNISYAGVEGALCI

IILLILPIKRLKINK

>A0PYT8

MGISKKFNKGLGVATTAMILMGGTSQLVHAEVKPSVVEKIQARKICKDIEQCIIDGTYNF

QSEKENLYFNAPNIVNNLKEAPKLEKVSLGTFYGEYKKYRIEIPSNVPKDYISSIKEIEV

NGVKYEVSKDGNVKEYNKYNIGMLGLDLSISAFNKEDNVITIKATGYKDNVLNVKKSSED

VDKEKDTKTSTKPKKEKVESESELKNKPSEKKEDKSSNKSTAEEKSGKDLNENKEDINKP

KVERVFAQKEGNKYSKYRVEFSNKTKWEVMSSYTKSITEVSVNDIKYNPSEDLLVNKNSR

YYFGLMGLDLSSNGFNKEQNVIKIKSNKYNTFIITVKKDGSILKINEEQYEKNKDSMDNK

KKKISVMKTALSDYGNDGKNKYQVILDCSYTEQNNYRQALKEVVVNGTRYKKALTVTENN

TFYASSIALDLNTASFNKDINEVVFKADGFEDITIFIRKDGSLATENSTEPLEDPDFNLK

HTNPTKEDKDKGNKASEKILLSENTKLEDGQYTIGFTAYKVDDPTDTSMLGGFFDSNVKV

EVKKGKIYTTWLNLLEADMLYDFRIENNGKYPKAKSTKYGEPDNYGKYNMQTFEIPMDNF

IKPHIGGVIVSAMGGQKSDIGNINKYTKVKLVFDKEIIKNWEGFKYEKENKKDKYQEQNK

IFSALSEAGLDFNKDGVIDSADLNRAQGELDLSCKGISNISWVKYLGGDVTKLFLNANGI

KEIPKDVFDRLANLETLDLSGNKLSTLPVGIFDKLTKLKSLSLSGNKLNNLNKDVFSKLV

NLEELALDRNQLTSIPNGIFDNLPKLKRISFSENKLDNIQDNLFNNNKELRVIDFSFNNI

KSIPTSIKNASNLSEIRAQHNRIEVLPKELGKLVNLKKLILSRNIINEIPLDIFKSLKKL

NVLEMNDNNISNIPDNIDKILPSLFKETYSAGIEVKYNELTKISDKLKELSKIGKFKYIP

QKSLTNLKLINDNGTLRWDHKMSSLDVLCWGETPHSFFDSIVPKTLEEYKQYLNGKSTID

VLNDRGWDWTIKVDIQKKNKKGEFETIHTITTEEEEDKFGSYKVGNIDNNDNYRIVKSLY

GSTNNEKSLIFKEIAYIGDSLNNAVQSNEKSKEQEKATTKSISVKVLKEKSDEPSMAGQY

VNKTVKYTEKDGKKYFTVTLNRIDWMKNVSIEVGGKEVAAEKNVNGNVGEYTFEVSNENE

EVTMKMNVVPMGNARVAFRLSKSNTSSKPQETSTEKPKEDNTKEKETNKDEGKVTSDNDN

KKNEKVKNIDFKILKEKTNEPSMAAQYVNKTVKYTEKDGKKYFTVTLNRMDWMKNVSIEV

GGKEVAAEKKLNGNVGEFTFEVSNENEEVTMRMNVVPMGNARVAFRLSKSDTSSKPQETS

SEKPKENTEDKEKQTENKNEVENKNEVVYEAGLELADKTNEQNEKMVKRYIDLKQKINVE

RKDKEIFMTLKLKAQQKDIKEIKVDGHVVKNFDVVGEEIGKVNRLAPLNNASNLKVEDNK

ESTTIRFKIPNEKSKVQITMHNEEENKDETFAIKLKGIKEVKNDNKKDDKDIKTDKENKK

DNDPINNKDSVEIKDKNMDNKKESSKEDKKEDKKQEKQEQQHKETNKEEALTSSNSLQKK

SPNTKERKSNKKATSIKEKKLPQTGMPLGSGLLATLGSAISGMGVVLMRKNKKKK

>A0PZH8

MKKGYVSLILHSHMPFVRHPEQEDSLEERWLFEAMSECYIPLINVYDNLIKDNIKFKITM

SITPPLMSMLEDEYLNERYLNYLEQSIDLSENEIIRTKDNEELNKLAYFYNDRFKKILDI

YNKYDRRLMNAFRKFDRLGVLEILTCSATHALLPLLTINPETIEAQIATAVQSYTECVGH

KPRGIWLPECAYTYDLDKILKKYGIKYFISESKAILNASPKPKYGTYTPISTHNGICVFG

RDMESSRQVWSSFMGYPGDFNYREFYRDIGYEAPMEYIAPYINRGGIRIDTGIKYYKITG

KTDNKQYYNREMAMKKVRDHAGHFADGRNAQLEYAKEHMDVSPLIVCPYDTELYGHWWFE

GPDFINEFIRMSAEDWTKYELTTPYHYIKENPIVQCSSPCPSTWGENSDYSVWLNPSNHW

IYRKLHRSEQAMIRLANTYKEPDEITKRALNQAARELMLAESSDWPFIIKNNTTVEYAVR

RVNSHISRFNKIYEDISRNSIDTKWLSNIESLDNIFPNIDYRIYSS

>A0Q035

MAEAEKKPSKFGSVFKVILVVFLAVILLGGGVFAGYMVASKTNPRGVVINTVQQETLNLK

TFGLDEFLLNLKSDDNSSRYLKTNISIGYADVKENKELEGELKDKKAIIRDTINSVLRSK

KKEDFATNEQVEKIKKEIKDKINPLLQNGQIYNVYFSEIIIQ

>A0Q0X0

MNNLQIFKNEQLIPLQENDNGEILVSGRELHEFLEVKTRYDIWFSRMKEYGFIENQDFIA

IVQKRTTAQGNETTYTDHAIKLDMAKELAMIQRNEKGKQARQYFIAVEKAWNSPEMIMKR

ALEIANRNVESLKLENAQKEKQLKEQKPKVLFADAVSTSHTSILVGELAKILKQNGVDMG

QNRLFKWLRDNGYLIKRKGTDYNMPTQYSMDLGLFEVKETSITHSDGHISISKTPKVTGK

GQMYFINKFIENEVLQEVACSR

>A0Q8J0

MNLFFNQNLAIGYKSQAQIIRVLSESWVASEIFCPNCGSIISSYKNNNPVGDFYCLSCAE

DYELKSKKDSMGTKIVDGAYQTMIEKIQSSTNPNFFFLNYDAKSLEVINFAVIPKHFFVP

EIVERRKPLSQNARRVGWIGCNILLDSIPESGKIFYVKNKQLQDGKEILQSWKKTLFLRN

SSKPELRGWIVDIMKCIERIGKNQFTLQEVYSFEQELKLKYQGNSYIKDKIRQQLQLLRD

RGYIEFVSRGVYRLRR

>A0QEK3

MAIRRAVLLIADIGGYTHYMQWNRTHLAHAQLTVAGLLESVINAGKGLKLAKLEGDAAFF

WAPDSSAKVVVCERLSRMRTSFLQRRERMKNDIACQCASCAQLEQLSLKFVVHVGEVADQ

RVKRHVELAGFDVILVHRMLKNLVPVAEYVLMTDPVVDCLDEPMRALCMPLVHVFEGIGE

TPTYYIDLAEVRVPVTEPQRSLLRRLGATMRFEFNSLPFTLGMKEPAEGFRNLGRGGEEI

PA

>A0QGF5

MRSIIGREDIQDDYDVLRAVVARIQRHSYDALTNPERIRLLEVLEYQTRRLQVPGHQLIN

QIEQDATPAEIGGKLAHVLADRLLITRAEAARRIDDARDLGVRRTLTGEPLPPRYAATAA

AQRDGRIGASHVAVIRRFFDQLPCWVDAPTRDAAEADLARWAGEHRPESLRKVADRIACY

FNPDGVFHDEDRLRHRGLTLGAQQPDGMSQLRGWLTPEARATVEAVLAKLAAPGMCNPDD

DAPVVDGPPPEAASQRDSRSAAQRNHDGLNAALRALLAGGKLGQHNGLPASIIVTTTLRD

LESAAGKGLTAGGTLLPMSEVIRLARHAHHYLAVFDNGKPLALYHTKRLASPGQRIVLYA

KDRGCSHPGCDVSGYYCEVHHVTGYAKCGRTDIDQLTFACGGHHPLAEQGWITRKNGRGE

TEWIPPPHLDHGRPRTNAFHHPEKLVADSDNDTGDDTSDAPDDPQPPIP

>A0QGN0

MATTSRISRRGNDSAATALAEPDQPDAHRALAEDVRWFAGSPLAAAFGRLALDQVAHREI

AAAVDRSGRFADNFTDRGIRSAAFTALAAFGDSSDVEASRADLKRLHRDVRGTGKGAFSD

TRYSALDPELWTWVAVSGLNLLYQAYLRVCGRRLSTDEKEVVYQTLRRELQFLELPSKQG

KLPATLDEMLDYYDTVAAKHLADNEFLQFASRSFVAPPVPGLLPRQLRPVLRLVWPVLTS

LAARPVVVCSAAVAHPTMRRLLGVRWGAREQAEFAVYVAALQLGWRWLPRRLTLEPLAYN

RYQYERLRDRYRSVLLDSFAAPGRG

>A0QJY2

MVLPHGRKGRKERQHMATDHTSDAPDPKQRDLESARFRRDTGYLTTQQGVRVDHTDDALT

VGERGPTLLEDFHAREKITHFDHERIPERVVHARGAGAYGYFEPYDDRLAQYTAAKFLTS

PGTRTPVFVRFSTVAGSRGSADTVRDVRGFATKFYTEQGNYDLVGNNFPVFFIQDGIKFP

DFVHAVKPEPHNEIPQAQSAHDTLWDFVSLQPETLHAIMWLMSDRALPRSYRMMQGFGVH

TFRLVNARGEGTFVKFHWKPRLGVHSLIWDECQKIAGKDPDYNRRDLWEAIESGQYPEWE

LGVQLVAEDDEFSFDFDLLDATKIIPEEQVPVLPVGKMVLNRNPDNFFAETEQVAFHTAN

VVPGIDFTNDPLLQFRNFSYLDTQLIRLGGPNFAQLPVNRPVAQVRTNQHDGYGQHAIPQ

GRSSYFKNSIGGGCPALADEDVFRHYTQRVDGQTMRKRAEAFQNHYGQARMFFKSMSPVE

AEHIVAAFAFELGKVEMPEIRSAVVAQLARVDDQLAAQVAAKLGLPEPPEEQVDESAPVS

PALSQVTDGGDTIASRRIAVLAADGVDVVGTQRFTELMEQRGAVVEVLAPVAGGTLAGGS

GGELRVDRSFTTMASVLYDAVVVACGPRSVSTLSDDGYAVHFVTEAYKHLKPIGAYGAGV

DLLRKAGIGNRLAEDTDVLNDQAVVTTKAAADELPERFAEEFAAALAQHRCWQRRTDAVP

A

>A0QNW0

MARPEPFVHITPDEQLDDLRRRLRATRWNDAPEDAVWSIGADSGYLRELVDYWVDEFDWR

QRELELNALPRFRASLDGLGIHFVHARAVEGSPAPVPLILTHGWPDSFWRYAKVLALLTD

PASHGGDPADAFDVVVPDLPGFGYSDRPRIPALNAAEVAALWSRLMTALGYPRYGAVGGD

IGSSVSRFLALDFPEQVVAVHRMDAGLPAGTAELGDLSEDERRWIKEATRWVGAEGAYAA

MHRTKPQTAAVGLTDSPAGLAAWIVEKMRAWSDCGGDVESVFSKDDLLTNVTVYWMTATI

SSSMRMYRANAAIPVEQYARRVEVPTGYSLFRGDIVRPPHAWLHRTSNAVYITEPPRGGH

FAPYEQPELYAEELRNFFRPYRNHT

>A0QPT4

MTSHWIRETIAQHKTGDPVGVYSVCSAHPTVVTAAVMQAAADNSFVLVEATSNQVDQFGG

YTGMRPADFRDLVHDIADEQGFDRDRVVLGGDHLGPNRWQDQPAAVAMTNADALIAAYVE

AGYRKIHLDCSMRCADDPEVLSDEVVADRSARLLRVAEQTANRLGIEPPVYVIGTEVPVP

GGAHETLTRLTPTPAEHARRTIEAHQAAFAAAGFEYVWPRIAALVVQPGVEFDHVNVIDY

ERTATAQLRRVLDTEDHMVFEAHSTDYQKPDQLRELVEDHWAILKVGPGLTFAMREALFA

LSHIEAELVDPASRANLIDVVERRMLAEPRYWHSYYEGDPVTQRTARRYSYSDRLRYYWA

DAEVDAARRTLLANLDRTGIPAPLISQFLPTQYERFRAGELDPDPRSLVIDRIRDALRPY

AAACRTTDHHATALTTGVAR

>A0QRN0

MSGITQNVREATMAIRDFRRACVPALVGGVALTTALGVGAVATAPAPAHVASDVGLKALI

THGSATNWNADGIDKFYGLDWNEKYGPTVVSQFSLFPYNVNPDNLKAALRNNVGDPEPDL

VLTSGRGSGAATVTMLLLLNSGRPEDRELVLNTRWILDNSVNRPNGGYGSRYIPFSILGV

YPWPTPTDEGVDIVDVGYEYAWNSSAPAYVTNVVALLNSIIAYAYRYKSQAFTGLPVDPA

LGIDPVTGEPKLEKGYHYIVEVDGTVTKEPLKSLGNTNTAYVTYRADGLPMLQPLRDFGG

QFGNAVADLLQPALKVIVDASYPGGDPVANPDRYITASLFTPPDVAIKAIQTLPSAIHQG

VEDFKKDLGLTKTKAPAADERQSEPKPVEEQSAPQRDDDTTEAPSAAVAVSSKLATESAA

TSVKSVADSGDVKDVKDVKAVEDTKKATDKRVRPRPLRKLVDRVTKALTPKATRPAKTSK

PSTGTESGGASTGQSDSGDAKSGDSES

>A0R489

MEKKSGRIEMDPEDVGRDLGRLVLTIVELLRQLMERQALRRVDVGDLSDDTVERLGVGLM

RLEEAMEELREYFGLRPEDLNIDLGPLGPLLPREHG

>A0R7R5

MKRKTVAAGFALCAAVAGVGVGVIRAENKPDPNVARAIEELKKQLPNISAPQISESEIPG

LFKLVAGQQIMYWSPGGYMIVGEMYDVKKGKNITAEQRQEIMKEYETVLSKKVQKMPLDN

AVKIGNGKNVVIEFTDPDCPYCKKMGKFLDEQKNITRYVFLFPLKMHPNAHAKSAYVLSQ

TDKQEALKRVFSGEFDKKPVPEAIASAKDQVNKNIKLGEELGISGTPTVFVNGSLVRGVD

FKRLKMLLESGQS

>A0R7Y2

MMQNLQRKVPSNVLSFVSSHIKGSEEVMERYKAVCSIIKSDEVAVRLLEGLIDAATRYFG

KVVEMENRLQTARFRLESEELKALTEDLDRSRRFAHDAMISDLHIFNRYLVKEYGEDLSE

AGFQGGIFPNPDAIRDRIAIADWAGELLSGIYAARKK

>A0RHF6

MRCLHMSVLDNFDQWKSFLGERLEQAQGKGLDGGAVSDMAFRVGDYLANEVEARNDQEKL

LAELWKVADEQEQHTIANLMVKFVQHK

>A0RR72

MNNERLDNISNSLGISKRKRTLFELEQISDNEMKLIIKNGKLNLSVPWFGMSGNTPCTLV

PAGLFEAIINTLKNAQKENFELKLEKSIWQHIPVDFGDVWSVAIDEIKKSKFKKEPNLDR

VVKKIKKEHPNLFVDMQSLIQSKEN

>A0RRK0

MQRNIYIAYALWFFLGGFGAHRIYCGKFLSGLLQLLLFWIGSLTAIFLVGYIFLAIWGIW

WLADLFFTSNWVEKLNSVNCIEKSISDSHKLKNVEKLYELYKNGAMSYDEYLRRKDEILG

>A0RTK1

MRHRMDPPTHRIIVDERERRSGIPDLLKSVDINVEVKTLPIGDYIVAHETVVERKSLPDL

ISSVFDGRLYDQCDRLRENFEHPIILMEGNVDEIDDIIENPLTFYGAVSRIALDFKIPII

PTPSAAHTAKLLVSMCLKKDRAAGPFLKKIKKSNDVQKQQLSSLSSLPGVGEKLAGRMLE

KFGTPLRTFNASSAELSKVAGLGPSRAKKIRKMLDTKSSLHKKGNQSTLQE

>A0RV62

MGKKELRDRDVWQKKSGLMGKKAEDEFEAVFLAEFTGTEYVIRKEPKELKDIYSKNPKHG

VSIDYAITNKKTKKTLYVEIKSQEGYVPGETLPKDGRGNAHERSCKFFTPGLLKVMCKLS

NLPVGTLPFWIVYQGRITRDAKRTREITYWFDEYGAHFFLWKSSSSTPLINHFKKNLRHI

LD

>A0RYA2

MAFRAGLALKDMEFVQFHPTGILPSGILITEGARGEGGYLLNNKGERFMKKYAPGKMELA

PRDIVSRSIMTEINEGRGFKHETGVDCMKLDLRHIGDEKIKEKLGGIREISIKFSGADPS

QEVLDIRPVCHYMMGGIHSDIDGRTELQGVWTAGEAACNSTHGSNRLGANSTSECIVWGK

ITGELAAEYITGGVPVPQFPYHMVAAEEKRIYDGIFRGNGNVNPYEIRQELTEVMNEKAH

VYRDGAGLADGLRRIRQLRDSAWRHTDDRAKEYNTNYINVMEVDSMFRVAEVVLVGAINR

RESRGSHARTDYPKRDDVNFLHHTLAYHDPREPLMKTHPVTITRYKPVERKY

>A0S5D7

MEVLVSFFVVVYLQVVTSAPKSLHEMKVECPHTVGLGQGYVTGQVELSYVPLTDVPNMLI

ESSCNFDLHVAAGAVQKFTEVSWAKKSSTTATTNAGESTFEATSKEVSIKGTCLITADIF

DTVYRSRKTVLCYDLSCNQTHCQPTLHLIAPIFTCMSIRTCMVSILSSRVQVTYEKTYCV

TGQLVEGVCFIPMNGVGYTQPIHAYDIVTLPVTCFLVPKKAEQLKIVTELEKLAGKTGCA

ENSFQGLYVCFIGKHSEPIHVPISSDLRSSTIMSRMAMHPRGEDHDTDKTGQGVLRIAGP

VKAKVPHTESSDTMSGIGFSGVPMYSSLSTLVKDSDPKYIFSPGVIPDSNHSSCEKKTLP

ITWTGFLPVAGKSEKITGCNVFCTLAGPGAMCEAYSENGIFNISSPTCLVNKVQKFKGSE

QRVSFVCQRIDNDVVVYCNGQKKVILTKTLVIGQCIYTFTSLFSLLPGVAHSLAVELCVP

GLHGWATIALLITFCFGWLIIPAVTLLILKTLKVVTYSCSHYSTESKFKVLLEKVKVEYQ

RTMGSMVCDICHHDCETAKELETHKKSCPEGQCPYCMTLTEATQSALQAHYTICKLTDRF

QDALKKSLKRPEVKRGCYRTLGVFRYKSRCYVGLVWGLLLTVELVIWAASAETPVMEPGW

TDTAHGVGMVPMKTDLELDFSLPSSSSYSYRRVLINPANQEEKIPFHFQLEKQVIHAEIQ

VLGHWMDATFNIKTAFHCYGECKKYAYPWQTAKCFFEKDYQYENNWGCNPGDCPGVGTGC

TACGIYLDKLKPVGRAFKIVSLRYTRKVCVQLGVEQTCKHIDINDCLVTPSVKVCMIGTI

SKLQPGDTLLFLGPLEQGGIIVKQWCTTTCTFGDPGDIMSTNKGVSCPEHSGSFRKICHF

ATTPVCEYQGNTVSGYKRLMATKDSFQSFNVSEVHVTSSQLEWIDPDSAIKDHLNVVVNR

DLSFQDLSDNPCKVDMHTQAVEGAWGSGVGFTLTCSVGLTECPSFMTSLKACDSAMCYGA

TVVNLVRGSNTVKIVGKGGHSGSLFKCCHNKDCSDIGLAASAPHLERVTGFNQIDSDKVY

DDGAPPCTITCWFTKSGEWLLGILKGNWVVVAVLVVILMLSIFLFSFFCPVRNRKKQV

>A0S601

MATSMITSPLLAPTRAKGMPSLSRRGSSFAIVCGGGKKIKTDKPYGIGGGMTVDVDANGR

KGKGKGVYQFVDKYGANVDGYSPIYSPEEWSPSGDVYVGGTTGLLIWAITLAGLLGGGAL

LVYNTSALAG

>A0XZS5

MEKQEPTFNSFKAFYPYYLKEHRNVTCRRLHFIGSLLVLMVIITALLSQKYALLWLLPVI

GYGFAWVGHFFFEKNRPATFKHPFYSLWGDWVMFKDILTGKIKF

>A0Y197

MLLLSIAVSALAVGLISLTLIIAFKNKHTSDVADVHQKLKECAEQVHILRSEAAELRTGL

LSIGKRVLEVEQQNQDLLQQQSAQKYDDPDAKIYSRAVKMVELGADLDEVIRECELPRAE

AELLFSLHKKKGA

>A0Y3Q0

MSDSPDVQKRSTNSNLLLAIITLIVVACVVAFVLLKPSDEPVARIEATEPVKERPIMELK

PLNEPDIIEPEEPTTVEQPAELEVPAQRIVEPEPEVATLPALNDSDTMVVAKIDEYLSDS

VMSLLVTDDVIRRGVVFIDNLAQGKVAKKHNPVIKPQESFSVTEGDILTIDPNSYERYTP

YVKIFTSMSAAQVVRMFEEYQPLINDAYTEIGYSDDEFKQTLNDAIDLLLDTPEPEGALP

LLRDSVTYQYAFSEWEQLPAAQKQLLRMGPDNMKKVKAALRNIKAQLESK

>A0Y3T6

MNDTHQTFQAGRRINTQADEQIEATLAPAKIVAQGQSEYIEEPNDELLEDEIDLEPVYKK

SKWQTLKGVFAISFLVLVLLEFAYSLVFTFQQSVILGGVYLTAVVSGVLLIGRMLWREYR

MLRSLKRNQLHRVKADRLLNSEQVGGALPWLEKLNKHQQLDNFDTFKNQVATHHSDKEIM

TLYANSLLITQDTEAKKLINRFATESALLVALSPLALVDMMAVLWRGTKLIEQIGKIYGI

GFGYASRIKLYRMLIKQVMFVGSAELVSDLAATALSAELLGKLSGRAAQGVSAGIFTARI

GYKAMELSRPLPRLEHKRSLLKETVQSIAGKIIKRGKTEPTK

>A0Y7L2

MKHLDRRDIVKGLLITMAAGLSSSCATISGAPARVVVIGGGFAGMSCARTLKKLSPSLAV

TLVEPKQHYIACPFSNLVIGNGRPMAAQTFSYDRASADGVNHIMQAATAIDADGHQVTLA

DGRRIDYDRLVLAPGISLNFDVLEGYDQAAAELMPHAWQAGGQTTLLARQLAAMQNGGTV

AISVPENPYRCPPGPYERASLIAHYLKTHKPNSKLLVLDSKERFSKQALFMAAWKSEFGD

IIEWQGLNDGAKVISVDPAAMTLHTEFDRIKVDVANIIPPQRAGKIAQLSGVANATGWCP

INAATFESTLYADIHVIGDAAIANAMPKSAFAANTQAKLCALQIARLLNGKKPIESKLIN

TCYSLVTPDYGISVAGVYQPDKERWLAIKGAGGTSPLLASRKTRRIEAQYARQWYASITA

EVFG

>A0Y7S1

MRLIQCEYLRLEEETGVIRAGSKEIERQLQQLPAEQAIVERANRLLSITEKNSLQAPSRH

YRQLSIALLGFFGILGGLATVEVFSGVEQASANFFWILLALLGVNAISMFIWLLLIGWPN

SADQNDSWLMSIQAWLSRKSSQDTSALRAWQQAISTTGGQQWHLSVISHGLWLAFLSGTL

VMAWLLLSMRQFDFVWETTILSGDAFVSISQWLATLPSLLGIQLPTASHVMVSRVGESPQ

DPDELRILWSNLLLSSLAIYGLLPRAVALLFAIFARRHHWNNVLPDVSTPYYYQLKMRLS

PDTVAEGIVDDDAETCHQTHNFSASNTATIAKLQQLDDVCLAGLEYDGEWPPALSAIGLD

DLAPIMFSVNAIDRSSREQLLSEAGNNSIRNIVLIARFEAAPDRGIIRLIRQLNSKPATL

WLLLIQHRSQQTSQQWHDWLSAATDAQLPQQQVGRLNIGIGNDGA

>A0Y8N8

MEGPSRPKLGISSCLLGNPVRFDGGHKRSDYVTAKLANYFDFSSFCPEQAIGLPTPRQPI

RLVAENSDAEVRAVHVRDYSVEHTKDLHQYAATVIKKMAGFSGYIVKKDSPSCGMERVKV

YEKDNAPAERRGVGIFTAKLMLDLPGFPIEEEGRLNDPRLRENFITRVFTMFRWQELLRR

GLTKKSLVEFHTRHKFLLLAHHEITYRKLGRLLADLGSVSVDVIATSYIKLLMTGLCHLA

NPRKHANVLMHIMGFLKDQMSADEKVELLGLIDDHRTGLVPVIVPLTLLNHFLRRYPQNY

IVKQYYLEPHPRELMLRNVI

>A0Y9W1

MLDNCTIEERWNAVDKLVERWLQERQLVIVQFCALSGVHELNQDDDPSSMRLQNFCQLLV

DYMSAGHFEVYYEVIREAEAFQDGSVNLAKGLMPEITLTTKISMDFNDLYTSGKGDLGHL

SSSLSKLGENLAARFELEDQLINTMHESHREQVA

>A0YAI8

MKKSMFTLIISITMAFPVAAETLTVVIKNVESANGYIMLVILASEQEFDGDIEAIASMKQ

RSLAGDNTFTVGNLPEGDYGFRIMHDKNSNGELDSNFVGMPTEPWGFSNNATGTMGPPGW

SDVKFSLSGKATQIITLN

>A0YAX2

MDDLVELFNRLFKSTENTLLVQGATEPVYIPASGACVHHQVVFSYDYFSSALHELAHWCL

AGKERRLVLDYGYWYAPDGRDQLQQNEFEKVEIKPQALEWIFSKCCGKTFRVSLDNLQGL

ETEGQAFKKAVYQQVLRYCDAGIPARAKILCEQLRGFYQTPDCLCASNFSIGELD

>A0YCS0

MFVKIKEFFSVQLSIEENSDPQQQLQLAAAALLIELSRADYRRDREEQIAIEAALKKSFS

VTDEQLTALIELAEEENQQATSLYEFTKLVKDNYSAEQRFELVKMLWEVAIADGEISKYE

DHLIRKIADLIYLPHSDFIRAKLAVIKA

>A0YDD8

MKIQKYRVKIKQVGSTWTSEIWRKVSKNEAAVSKGQAGFASEQEAQSWGETELKAFLSHL

GDRNQRRAEKRS

>A0YFN6

MANIQITHDYTMPADELKAKIDELAAEMAGRYQLQCTWSADNCMKFQRSGASGEIRINDG

HLALSLKLGMMLGAFKSTIEKDMKKFLIDTIR

>A0YFU6

MNLAEVEHELLERAIQAFHTETGLLLDIIQEQVVFDRYQVDAIIKIPKYGIELGVEVKRW

AQQANLGALADQIKRMPIEGILVADYINPNMARRLKTMDVQFIDAAGNAYLNQPPVYVHV

TGNKKPDTKAAFKEGVNRAFDATGLKVVYGFLCNPELVDATYREIAERTGVALGTIGRVL

NGLTEAGFIVDRGKGKGRRLVKKRRLLDRWVEAYPEKLKPKQRVGEFTADDPYWWEEVHA

EKYGAYWGGEIAAAKYTNYLKPETVTLYLPEYTGKKLLARARLRKLREGEAGGQGTVRIY

RPFWPVEDEEQHYNNNMAEVPGVVNPILAYADLIATGDSRNLETARMIYEQYIAKHIGED

>A0YG29

MQDELMQQGVELMLYGMGTVFTFLALLIVATTCMSIVVRRFVKPEPLPAKLVHRQPVDEN

DEQLVAIIGAAIHKYRSRNK

>A0YGS1

MATPVVLSNDDIANRLGVNSQWELKDNKLYRRLMFEDFIHAFGFMAQIAMVAEKINHHPE

WANVYRTVDIYLTTHDAGGVSEKDFELLAKIEQLIGQP

>A0YGX4

MIIATAGHVDHGKTSLIKQLTGVDTDRLEEEKRRGLSINLGYAFKKLDDGQVIGFIDVPG

HTRFINSMIAGVGGIDMAMLVVAADDGVMPQTTEHLDVLRLLGQQQFVVVITKIDRVDIA

RVKEVTAQIKRLIGEDVTCFEINNINGEGIAELQRYLDSDARNREAANDCGHFRMSIDRV

FSIKGSGLVVTGTALAGKVEEGDTLLLQPHQTHVRVRGIHAQDKKVTAGYAGQRCAINIS

GDIDRENVARGNALVGESLSTLSRNCDVRFTLLASVNFPLKHLCPVKIHLGAGRFTGKIF

FLEQKRKLMPGESILAQILFDDPVSICHGDRFFLRDDSESISLGGGIVLDPLAPQAKKST

AERLSTLNILQSSDPKAVLNALVIEQHQIVDFEAFKRSWNIRDQEQDQFIGEGTFVVAGE

MLSKAHWSDLTANVLSLVTKQHHEQPSSDGVSTLTLKAGLKDSVFTAVLSELVHAHNVVI

KNGLVSLKQHTSRLSPEQQALWTKIEQVYQESDTHLPVVADLLKATNIQKKPLLSFLQSR

VNDGQLLRVNENRFARPQELYQLSNKAIELGADGRAFDARQYRDAIGMGRNLAIELLEYF

DEIRFTERSGNERHIINASTPEKLFKL

>A0YHZ3

MNQPLSRPKIILDTDPGGDDAFAFFWLISLVKKGLAELLAVTSVEGNVNAKLTFTNACKL

LQLNNFSDIEVGRGVIKTQKEIDDAAHIHGNDGLGNLAQTLPSPQQSYENARYSDDILIE

KLTAFPGEITLIALAPLTNLAAAETKSPGILKQAKEIIIMGGAFNVAGNVTPEAEFNIAY

SPEAAEIVFNNSNHLVILPLDVTRSLIFRPEMVQKISEVNPESDLTQFIVALAKAMTQTS

LSHRETQGIEGFLVHDAVTLAYLFYPETLQFRRARVEVEVVGKLTPGKTLFDRRHHAKIA

ANAWVAQQVNSADLFAAFVEDLKSLVKSDP

>A0YIF2

MKLFYYKRRDGSSNFGDQLNHWLWPQLLPGFFDEDETTTFIGIGTLLNQFLSQRVPQAKQ

LIIFSTGVGYEQPLKTLDKNWKLYCVRGKLSAQKLGLSPDLAITDGAILTRRLYQPSPQK

NYRFTYIPHIHHATFAGQIWQRICEQIGFRYLDPRSSVDEVLSTISQTEVLLAEAMHGAI

IADTFRVPWIPIITSPRILPFKWQDWCSSIDVQYYPYFLSPLIKSYPKYARGIRSGFNAF

THWKNSFLQFPQISQFWQEQEEVYGMQLLKIAQTGRQQLSSDSQLERLTVQLEEKLDRLK

TDWYSKS

>A0YIL0

MLPHGANLSQKDNHKTKYKDDISKQYLSEIRIKYQQWHEQNTSLKGPLIQKNNNDQQILE

YRVNYLNDYKDFIDQQKYAEKFDSRSNLHSTVLEEFMYYLFKDLVAEFSEYALIGKSHTF

KDIFFLPSSYRNMLRSPHALIERKDHDFAIGVQVDAILQCRGQTEREHDSWDIPAVAVEC

KTYLDKTMLQDASTAALQLKNKNPNALYIVVAEWLKLTNAVNLRKYQIDQVYVLRKQKNT

DREFRYLETYVKNPIYSDVVIHLFETVREFLSYPSWEGGVNYGLQRGYLI

>A0YJ99

MTTLLVPANLQAAGRLGLDAFDGTKVELRPDWTESDLQAIFRATYRQVLGNEYVMKSERL

TSAESLLRQGNLTVRDFVRAVALSELYKKKFFFPNNNQRFVELNFKHLLGRPPHDEQELA

FHTRLVEDKGYDAEINYYFESEEYENKFGDNIVPYYVGFQVLTGSRTVGFSRMFQLYRGY

ANNDRGQVGSKNGHVFKEVARNQASTIPQTAGISSQSTKMDTPQKSFGGLGNRQQRVYRV

EVTNRIGSSLKDTNVRRSNKAYLVPYEELSSRIQQILRSGAKIISVRET

>A0YQ31

MLTSPYRGYQEHEWSAITRQLIEEFPLSSEVIISTVEAAWEDLYSSSFGDFRLQIGRDIF

LPAQAIGVILERLIAVRLAHQNSGWRGSQTKPEKDIVCTFNERYSFEIKTSSSKNSFYGN

RSTGYRSDNRLKSRTGYYLIINYKLPKEDDLERKIWKIRFGWIDDEDWIGQNKPTGQQAS

IGTKKAGLKLVTLKSSQ

>A0YQI8

MNALSTYDLIKEPLLDLLRDIKTGKIQLVDFQRSWCWTEERIKEAIASVSLGFPIGAVML

LQRGNPDIKFKYRPIEGVASDNLIEPKGLILDGQQRTTTLWMSLLSNQPVWIDRGKRYKP

DQRWYYLDIEKALDYPHTDRVDAIIGLKANKKLQQLAEPTIDCSTTEKEFELGLFPVSEV

FNFMQWRSGYWKYWQGNSQKIELIERFEREVIKQFEHYQMGLFVLRPSLPKEAVCYIFEK

HNQKQQELTQFNLLTSSFAAEDFNLRFDWQNREKRFAAYRVLRLLKPSDFLQSIALIDSY

TQRMEALEKGCSVDKLPSVSMSRNRILNLSQDQYQKWSEPISVGLEKAARFLYEQAIFDA

DDLPYPMQLVVMAPLFVILGEGVKVDAIRRLLLQWFYCGAASGTYSRGREGKAAKDLIEV

PQWLQDGNIPATVLEAFLTEERLQNLTNSQGSTYRAISALLRREGALDFFSGESINSVQY

FDEKIENHHIFPQQWCKSQGIPRTRYNSIVNKTPLKLKTNKLLGGKAPSEYLAKLRKQGM

SKKRIDQILFSHLIEPYTLWTDDFDAFFEYRTAALLGLMTEAMGKESNSQSISVKPLKIK

LLSIR

>A0YTL9

MAQILDPLPSNVSSQILCCYVNATSQIQIARISNIANWYFERVVFPGQRLLFEALSEALL

EIHTGSMASAILSDRIPCARLQIDQNAPSVPTEDAHSSQAAQAYHQRVMSMSQKPRQPVP

ESLAKPALTAVD

>A0YTW8

MVKIVGIGGSLRWESYSQQALKIAAQRVEVLGAEVEILDLRQMRLPFCDGGKEYPDYPDV

EKLRETVKQADGLILATPEYHGSVSGVIKNALDLMSFEQLDGKVAGLISVLGGQVNSNAL

NDLRLIMRWVHAWVIPEQIAIGQAWGAFNEEGKLLDEKLSQRFDGFAQSLVDNTTKLRGN

>A0YU33

MKRKTRTSPARSTFNYTALAVIGGVLILGIGIGIAISSTTTFSPENVASSQFIDRSAPST

ETCIKFGASAMVTDMRVFVTLNPFNVYISQPRMQPGCVLRTSNWTILKQNNLISSEQERD

CKQRMNTFGYTGELESSPEISCIYQNNSAENLFLSQPGGGGMTPARPRAESDRF

>A0YVL5

MKLRSFLTILAVSSVLILLLSIAIFYKLLGQSPLVAVTGGVTTTPTAAIFVPKTVPVMVS

LLVNPDRIEAFEQVLTRPQDRPRSHAEFNRIKKTLLANTHLNYNRDIKPWLGNETTVALM

TTDIDRNLKNGEQPGYLFALSATDVKLATTTLAQFWQQQQSGGVDVVSEPYKGVKLTYSQ

PDQNGSRPLSTAIFNRFVLVANSPKILREAINNVQAASLSLSQSLAYQQALEQLPESRVG

LVFLNLGRWGLELENPIVNLPSQPNLTLSLGLSPQGLLAHTALVKGEDKDGKSVEPALSE

PIQAWQYIIGKSGFAIAGTDLNHLWEQLSVTLAGNSGLENLVNQPIRVIKDIWRLDLPQD

IFNWVTGEYALAMIPPTPEKQKQKPDWIFVAERFSPQAIDAIKHLDELAVEEGYSLGSFE

LGERTLSAWTTLNPVPLPTTPNVNPQVLQAKPKGVHATVGEYEIFTTSVEAMDQALSLVS

QKTLLNDPEFKIAFQQIPTASDGYFFLDWEASVGFLNQQIPLLRLLELSAKPFFDHLRSL

TISSAGAVEGVAKATVFIRLK

>A0YY36

MMLEPGTLWKRTQEQTQFALQTGALQSIPTEYQFIEQDGILFFVRIVSNLARKEKAKQQQ

EQKTETSGKPFNPFLPYEEDLFVANLSPTHLCLLNKYNVVDDHLLIVTREFEEQENLLTL

QDFEALWMGLAEIDGFVFYNAGADAGASQRHKHLQLIALPLVPNGVKVPIEPAINSAQFL

GAIGNCPQLPFIHGLIRLDPLLVDSPEEAAATTLEFYYNLLSKVGLHGEDGMKGKQSGAY

NLMMTREWMLLIPRVQEGFDGILVNSLGCCGAMLVKTEEQLQHLKEITPMTVLRNVCIRK

>A0YY62

MNITKLFSSGVFGIVSIFLLKSIPVSAQPIQSVSSTYNSTSDIQIHFQPPHSTSDVADNG

KPKTEGTGTRSGNKCLATKIPLTVLTGQSSSLSLTASSHPTFFVYVPYTSTEVSYGVFSL

YDEQTKQEVWNVVFQLSKQPGIISIPSPKDQKPLELEKNYRWFFELHCAKNDAINQEPMM

VRGKVKRVSSEGFETELSAATPLEKVAIYAKQGLWYDAIGQLATLQQSDPHNSQLNTLWV

ELLKASNHQQLKPIYQESLVGDIILNLSSHIEENRSN

>A0YY82

MKVRLMWYLGWVLMGSALLFPASQRLEVQAQFIDNPKNSPNNITQTLTKDELYFGLSKQQ

GQVSEAEWKVFLEEVITPRFQAGLTVIDANGQYLNAAGILTREQTKLVILIHSDTPQEKQ

FIQEIITQYKQRFNQESVLRVTSSVAVSF

>A0YYA2

MSSTPTPSSTPTQTPDAVETPKKTEPQPSYVKLAMRNMVRKGGKSLFHFSLTAITLLGFL

VGVSYITR

>A0YZ83

MVSRFTHNLVTFVDITHTGCTGTEIMTTTPSKTTVPSKVELTPSFIIPAVLLLTGIPLIL

VQKVLGFVIALLGLFLLIQTATIRIQFTETALDVSRSGKLLRHFPYADWINWEIFWPGVP

ILFYFKEVNSIHFLPIIFDPKTLKACLEANCGNLKTRER

>A0YZ87

MTKKLTEYNFTFIDLFAGIGGIRLPFEELGGKCVFSSEWDEAAQETYEANFGERPLGDIT

TIDPGSIPHHDILLAGFPCQAFSIIGKMEGFNEARGTLFYNIEMILKAKQPQAFLLENVK

QLKTHDKGKTFKVITEHLQNLGYHLYTEVLNALDFGLPQKRERTYIVGFLENIHFSFPKP

IQKRVSLSEILEPDEQVDKSLFASEFIQKKRIQRLKVKPFYPSIWHENKSGNISVLPYSC

ALRSGASYSYLLVNGVRRLSSRELLRLQGFPEQFKIVVSHAEIRKQTGNSVPVPVVRAIA

KQMLNALEDKKPALEKPEQLSINFASFNKGVIIEMSVSLNEAKDALDNIINKARVHFYKP

IQVAEILYHHRVFDDLTLSNLNTYRKSSKKWRDIICKRFLGRITTSSSRYQDNLFEQNAT

PPEVLMVLGEENKSKLGIVEAYIYRKFIERYSQMTSGLAYCMKSDIENFKLTEFIGQFQN

NPGLKRSIDKIYEIVVYALFKVLIEELNVTVKVQLDFNKLDLLKEFEDFTQKVLSINSQN

PSFTASAKFYRVGVTNAADRGLDMWANYGSAVQIKYLSLNEGLAEGIVSNITADRIVIVC

RDSEERTIVSLLNQIGWKSRIQSIITQSHLEAWYEKALRGSFRNILGARLIEILIEEIKY

EFPSSESNDLVSFMEEREYTLLSDPFWSV

>A0Z0Z4

MKLLSIAMKVLGFICVCAVIWFAGPLFAFAGFKPLGAPATRLTLIALIIFAWFGKKLYRR

IQTAIANKQLTKEIVAKPLEETSAADADIATLSHNLSEALTTLKDKAGAGLYELPWYIII

GAPGTGKTTALANSGLRFPMAEHTDSASVQGVGGTRNCDWWFAEEAIFLDTAGRYTTQDS

DAEADNTAWLSFLKMLRKGRSRRPINGVVLTVSTSSLLESSPGARDRLLHALRQRLQELQ

GHLGVNPPVYLLVTKTDLLQGFQEFFEDLGQRERQQVLGFTFDLSQNESLAAIPEKFRAL

LSSINKRVLWRMRSERDRRHNNSIFAFPQQLATLSEPLTEFCQSLFSASRYEATPLLRGI

YFTSGTQEGTPIDRLVGSYARSLSVQESVGHGQARSFFVHDVVRSVMLSERELVGSNKKL

EQRNRWIQRGVIAGSAASLVLGLILWGTSYARNNSLVAQYEQKIADFQTLQAAKPGPDGL

DGLLERLDAAASPLRFVQEKNSDTPLTLRFGLYQGHVLAEEAETLYQAELEHFLLPTMTY

LIGQNLSRPSTDPQLIYEALKVYLMLSQPKRREPQLIRTWMGHEWEQLLPGKPRMRGRLQ

EHVNAITALDFKTATADTTLVQTARTTLSQSSMSALLYARIKQDYEFKGQPQVMLLDAAG

PYGKEVFSVAAESPDPSVSQLFTREGYKDSFQPQLQALGQLAADENWVMENAATKLTDEE

IKELQEEIQALYFTEYIQTWDSVLGGIRISPFKDLSTATRRLKFLSGESSPFVVLAKRVF

EETSLATGAKILDTGVASGSVIAGAADRLGKMLGAQQQDKLVDQIMGPERTVDKHYEGLH

QFFRANDTSSTPAKDTQTLINDVYKEFDLLSSGLLNDADTRLSGLFDSDQILQLQLQARD

TAPPLNRWLTEIANNSRGAVFGELRSGINKAWASDVAGFCNQAASGRYPFSTQSSREITL

TDFAKLFGPNGLIASFQKDHLDSFIASKGRRWSWQKFPGIDATLSTAALTQLRRADRIRR

AFFASGTPDPSISFGLSPVYLDANVRRVSIEVDQEAFQYRHGPARVFPIKWPAQTGVESA

VRFEFKDDSAEAIAQTIRGQWSLFRFLDQTQRTSVSSENINFTLEYKGRKTIWQIKSQGI

ENPFAKDVFANFSCIKSF

>A0Z1A8

MMILLTIFMMGLGSYAMRAVFILLLSNRSFPPSALKALEYVGPAVMAALVMTMLLSPEGG

SAPRVNEWAGLVTAAVVALVTRNHIYVLTLAMAVYWGVGYLV

>A0Z3N7

MNSQAPVAARKVLGEDSPTIKTRRSVAGNDVAVLSAIYEEDVNIANWHRELPEPLRCSVE

HLLDLAPAFGMSLMTTPAKAYEGVSEALPKNYEGTLAEDIAEIVDMFCFLFDLKRAGLRL

TALNEAMCPRFHVDKVPCRLVTTYSGRATEWLSQEGVDRTKLGMGYLGLPDSDLGLYKSA

DDIRTMRSGDIALLKGEAWIGNENGGLVHRSPNLTGGARRLLLTLDFGG

>A0Z438

MTEDKEYSRMRWASRRGLLELDLLLEPFMEACFRALEPQLRDDYQQLMGHEDQDILNWIM

GREALADETLTAITEQIRVHNRSKLR

>A0Z5A7

MSLDTWLSEWGVTLGVTALMALMVFIVWDLARRNNAGRYGTFILFIALAMGLLGFAIKGV

IQFLMEGTGV

>A0Z5B9

MTTQPVYKVIFQNGGQVFEVFARQIFQSDMWGFIEIEELVFGERSAVLVDPSEEKLKNEF

AGVSAVISLCTRLFALMKLKKRAPFACPRPPPAAPKSHIYRLTAFPPFPPGAMTDLSKGL

ELRLNDHHILVNEGM

>A0Z5U9

MPATLTLTAVEHLPRVTAGDDLVALIVSATARQALSPEPGDILVIAQKIVSKAEDRLVAL

ASVSPGVAATDLAAETGKDARLVELILSESRTIVRSRPGLIIAEHCCGHVLANAGIDASN

IAQDDEERVLLWPQDPDASASGLNQRFSQSYGFEVPVIINDSMGRAWRLGTTGHAIGVAG

LDPLWNQVGERDLYGNELRVTEPATADGLAAAAALVQGEAAEGNPVVWIRGCPRSSGEPR

SSRALLRPHSADMFR

>A0ZB92

MVQRGSKVRILRPESYWFQEVGTVATIDQSGIKYAAIVRFDKVNYSGINTNNFAIDELVE

VEPPKAKAKK

>A0ZDJ4

MTKPHSTVIPSCTWSRPIGLGWDKPYTVRYASNIDDGPWHGMPLGGFGAGCIGRSSRGDF

NLWHIDGGEHTFQNIPACQFSVFESDGTSAQAYALATEAPENGSLSAWKWYPASTATQST

GTYHALYPRSWFVYENVLQAQLTCEQFSPIWAENYQESSYPVAVFNWKAHNPTNAPITLS

IMLTWQNMVGWFTNALKSPEVRIRDDGSPVYEYQPRLGDSQDNYNCITENTENFSCFCSR

VVDDDSVAEGDGSWCIATIKHPQFKVFYNTCWNPAGTGADVWETFAADGSLTNYQDATPA

LENTQIGVAIALRFTLQPGETLAIPFVLSWDFPVTEFAAGINYYRRYTDFFGRGGDHAWA

IASTALTQYQTWYKNIENWQQPIIDREDLPDWFKMALFNELYDLTSGGTLWSAASELDPI

GQFAVLECLDYRWYESLDVRLYGSFGLLHLFPELEKSVIRAFARAIPHSDNHQRVIGYYY

TIGADTTTAVRKVAGATPHDLGAPNEHVWEKTNYTCYQDCNLWKDLGSDFVLQVYRDFLL

TGADDVQFLADCWAGIVQTLDYLKSFDLDGDGIPENSGAPDQTFDDWRLQGVSAYCGGLW

LAALEAAIAISDILLTNHRGAEDTEELGQRYRVWLAQSRTIYQEKLWNGQYFRLDSESGS

DVVMADQLCGQFYARLLGLPDIVPSDRALSALQTVYHACFVKFCNGEFGAANGVRPDGSA

ENPNATHPLEVWTGINFGLAAFLVQMGMQDEALKLTGAVVQQIYHNGLQFRTPEAITASG

TFRASTYLRAMAIWAIYLVIDAKKHILHSDTNTV

>A0ZE39

MDVSNPNLLLKRVVNVKVIVTPLWKKEVQEQLQAQINQSDQQLQQLDVEGQRAVAAIQKQ

SLQPPGPQTLQQIDSIQGQVNQKKTELLEQKNQLLQNLQQVQFLELDQEVNQFQMEGFFR

AEKGDNLISKMQVEILLRDGVIEDIRGDI

>A0ZIV1

MTVGLWGVESAQALLRQHHDSPGVLRYHSQVSIKDEKGYAWQVLLFKQNYNSPVKDLRLR

VVGFPGIAEIAHPQPLEIEAASGKLLSASDVYALAAPAPNVGEYNLTNVLPQLSSTDALK

LYVPTKSVKPLVLKIPNNVVTEWQWLVTEID

>A0ZJ60

MTGQELRKLLLDKWGYSYDVQFRRTKGKIFLQVMWKYLEQASFPLTEAEYQEHLDSIANY

LQALGGVVQVQTFIAQTRDRPRLGKAVSIPLDLGERASEWIV

>A0ZJG5

MQWQPIENLPSNWENLASAELPPLVTVWNEQAERLRSSGEFQTFMDRLRREIAIETGIIE

RLYTLDRGITRLLIEQGINEALIPHGATDRPIKQVISLIQDQEAAIEGLFDFVGGQRTLS

TFYIKQLHQLLTQNQDSTEAKTPTGQIIRVDLIRGDWKQQPNNPERPDGLIHEYCPPEQV

ASEMDNLIQWHHQHRIEKIPPEVEAAWLHHRFTQIHPFQDGNGRVARCIASLVFMQAGWF

PLALTRDDRAAYIAASEQADQGNLSNLINLFSKSQKQAFIRSLGLSEQILSETRRAQAVI

ASIADKIKQNQSATIQDRCQKVEGFATNLFEIASTRLQDIANEIKLSVQNLLSDAQIFTR

AAPAGDPKSYYHRFQVVETAKQLGYFANLRPYHSWIQLVIDVESTTTILLSFHVIGHEYR

GLLACSACAYHRDNTEEGERNISDIQSLTDSLFQFSYADEESNLTERFKQWLEEVIVTGL

EYWNESI

>A0ZLK6

MQIQFREINPFDVWIWLKFTTIPSGREKQYIEEVFNSWFYLGKLGAFNAENLQVQETGLE

ISYMNYDPEGYDKSLLALMHNMGEFEYEGQWARCWFDMGTADAIALDILINALKQLNQEY

VAIEELYIGGENEDWPVEDSENPNHSMYDN

>A0ZMM9

MEYEKFVDAAGKLPFIPDRETADAAVKAVLGILASKLEDEQAHQLSDSLPEPLNYERLHS

HQIRKLPISADEYIAVICEQFKLDDIQASELIQETLRVTKEAVKDKINEFTSRLPADWSA

LIEAA

>A0ZS32

MNNREQIEQSVISASAYNGNDTEGLLKEIEDVYKKAQAFDEILEGLPNAMQDALKEDIGL

DEAVGIMTGQVVYKYEEEQEND

>A0ZT57

MRFSGSPATPWPSSDPLLTRWNSSLTGAPSSPGLASSSLPPTSPSTNSSDLLDRNEALAR

VEVAVLALIFVAAVVGNSCVLLALQRSRRKSSRMHLFILHLSLADLVVALFQVLPQLCWE

VTYRFRGTDALCRVVKHLQVFGMFASAYMLVAMTADRYIAVCHPLKTLQQPGRRSHAMIG

SAWALSLLLSTPQYAIFSMCEVRSGSQVYDCWAHFIEPWGARAYVTWIAVSIFVVPVLIL

ATCYGFICYIIWTNIRGKTRPSNGGAQLLSTSAVSSVKTISRAKIRTVKMTFVIVSAYVI

CWAPFFTVQTWSVWDKNFMWIDSENTAVTVTSLLASLNSCCNPWIYMFFSGHLFQDFIHS

FICWRKMQHNTQKEDSDSSCRRQTSFTRINNRSPTNSMEAWKESPKSIRSTRFLPIQT

>A0ZVQ8

NNRPMVYQWRSSEFDPSSILRPFRSTGTQIYLEIDNRRCSRLNDGECFVSAKKAAQFLAA

AHSRHALSTAFPISQIQESSDDRDGPVFEDYSKLTYFWIGGGVVLFTILVLGVLVGSNRK

RARGITWFPEGFLRNNSGQRRRSHRRGPDGQEMRNLHKQCSINHLEIMDNGLGPPESQWS

DDDMDHPPMKRMRSDRSPNPSYGDTHTVMTTDDYDDNDPRPWTQQHLDAADIRNPDILAL

TPPQGDNDLDPNCVDVDVRGPGGLTPLMLASFRSGVNGFADMKDEFDEDNGSGNILTDLI

MQGADINISTERTGETPLHLAARYARADAAKKLLDAGADANAQDNTGRTPLHAAVAADAQ

GVFQILLRNRATNLNAKMHDGTTPLILAARLAIEGMVEILINAEADINAADDLGKTALHW

AAAVNNIEAVQVLLSHGANRDAQDAKEETPLFLAAREGSYQAVKILLEHFANRDITDHMD

RLPRDVAVERRHKDIVRLLDDYMPSSPPINIPPNGPMGSPGLMGPNNMLGSTKSVKPKKR

PKLNTIAGLPIKEQEMLCAEASVRRKPSMKKRKEPPPMLIKNSDGSITLSPVNSLESPSA

VSYMEGTPSPRDSIFNGHMGMSHPNLLNVDGNCTMSNKQPPSYEDCISAATHIYDMMGMD

ALTMGLGNNFGNNGLLSNQQMQQKNLKPNHPRQNSMPSSMSQNLTVPTSLAYTMSNTMNK

QRPSLPTSPTHMAAMRAAHHQKTVQMQSHPNVGNTYDYPP

>A0ZXG5

MANKISTVRTYPLNGSVNFTITFEYLARKFVLVTLIGKDRKELVLNQDYRFTTKTQITTG

RTWTAADGYEMIEIRRFTSATDRLVDFADGSILRAYDLNISQIQTLHVAEEARDLIADTI

GVNNDGNLDARGRKIVNLAFATSDYDAVPLKQITDRESSVWNAVTKASEHADRSNKEANR

SRDEADRAKREADRSTQQAGVSATQAVEAKKQADRSNSEANRSKGYADSMTASVEAAKGH

AESASKEANRSRAEANRAADEVTKAAAEVSKAAAHVASAKNQADRSSTEANRAKSEADRA

KTEADKLGNINEFAGTLEKVEGVTPTFKSGIKLRSGDFYAESGSFTKKTQGGDWSQWTGA

LTPDVQHDIEGQGNIAYLLERATTLSKEPLYAKCLYNNGVGENFFIRQYLRDAHFDLRAD

GALISSSGWSIPADGNLYIKKYGSNLDTWVNKRLSAHAYSKGEVNKMVDGLLTTEQGDAR

YARKSSGWTEVWQGSAGGGVSVSLSQDVRWRTIWILVNNGMCSVQIGADATYFVVVMGGW

LKFTISNNGRTFRNDQDRNTVPEQILVRN

>A0ZZH6

MKNKVQLITYADRLGDGTIKSMTDILRTRFDGVYDGVHILPFFTPFDGADAGFDPIDHTK

VDERLGSWDDVAELSKTHNIMVDAIVNHMSWESKQFQDVLAKGEESEYYPMFLTMSSVFP

NGATEEDLAGIYRPRPGLPFTHYKFAGKTRLVWVSFTPQQVDIDTDSDKGWEYLMSIFDQ

MAASHVSYIRLDAVGYGAKEAGTSCFMTPKTFKLISRLREEGVKRGLEILIEVHSYYKKQ

VEIASKVDRVYDFALPPLLLHALSTGHVEPVAHWTDIRPNNAVTVLDTHDGIGVIDIGSD

QLDRSLKGLVPDEDVDNLVNTIHANTHGESQAATGAAASNLDLYQVNSTYYSALGCNDQH

YIAARAVQFFLPGVPQVYYVGALAGKNDMELLRKTNNGRDINRHYYSTAEIDENLKRPVV

KALNALAKFRNELDAFDGTFSYTTDDDTSISFTWRGETSQATLTFEPKRGLGVDNTTPVA

MLEWEDSAGDHRSDDLIANPPVVA

>A1A142

MAAEPTSPAQPPQLPTAGSLAGILDVLETDDDFRALIAGEIEEPESDIDPSITVGVPDGL

RPALAAGAAGKQPVVLVVASSREAEETVESIRSWYDGDPNDVAQLEAWETLPHERLSPRA

DTVASRMAVFRRLKHPEEGSTLFGPIRILVMPVRSLIQPVVAGLGDVEPLVFSQGEELLL

DEASRKLVENAYTRVDLVMDRGEFAVRGGIIDVFPPTLPHPVRIEFFGDEIDTIREFHAS

DQRTYGKDISTVWATPCRELQLTDQIRQRAKSLIGSIPNAEDMLGSIANAIPVEGMESLL

PALVDDMEPVQGMLPKHALVMLSDPEKLRRAADDLAKTANEFLAASWHVAASGHGAGAPI

TFDQANFYDFEETISALVFSKHDVWKLTSFGVDATREGHIQLDATKPGEYRGDENKAASG

IEGLLDAGYAVTITAAAQGTLTRLKRAINETGIANFDCVRSHAIDGFVDDAAKIALLTER

DLTGRTSAAGQAKTPKRRRKAIDLMELKTGDYVVHEQHGIGRFVEMRQRTIGKGENQTTR

EYLVIEYAPSKRGAPADKLFIPTDQLDQVSKYIGAETPKLNKLGGSDWAATKAKARKHVH

EIAEDLVKLYSARQRTKGYAFSKDTPWQKELEDAFPYQETADQLTTIDEVKSDMEKPIPM

DRLICGDVGFGKTEIAVRAAFKAVQDSKQVAVLVPTTLLVQQHFETFTERFEGFPVNVAA

MSRFQTAKEINETIKGLEDGSVDVVIGTHKLLNPKIKFKDLGLVIIDEEQRFGVEHKETL

KALRTNVDVLSLSATPIPRTLEMAVTGIREMSTLATPPEDRLPVLTYVGAYEDAQVTAAI

RRELLRGGQVFYVHNRVQDISSVAAKIHELVPESHVGIAHGKMGEKQLDGVIRDFWHRDI

DVLVCTTIIETGLDISNANTLIVDHADRFGLSQLHQLRGRVGRGRERAYAYFLYDPTKPM

TQQSHDRLATIAQNTALGSGFDVAMKDLELRGTGNLLGDEQSGHIEGVGFDLYVRMVSEA

VEQYKEPERKESVAVTIDLPIEASIPVDYIDSDKLRLEAYQKLASARTEDDLDELRDELT

DRYGKPPVDFEALFDVARLRFKARKLGISEIIAQGRNVRVSKFEPRESVQMRMARIYKGI

QYRPLTKTYLVPAPFAGSLGSKPMSSDEVVGWTSQLLDDLDWKPTPRQ

>A1A2C4

MVLSTRLEKSRKRDIPTWKPFRASGSGRCLPPVVSVRYVSRSYGFTARCDAFWHALNNRD

FGKLPIKGCSVAWNMRIGVIISDEPRINDEIRVSQVRLIGPNGEQVGVIATSVALNLAKE

ANLDLVEVAPNAKPPVAKLIDYGKYKYNEKIKAREARRNQSTAEIKEIRFRLKIDDHDFE

VKKGHVVRFLNGGDKVKVTIMLRGREQSRPIGGVELLQRLASEVEEYGTVEFAPKQEGRN

IIMTLAPKGKKVHTQSEQRRRGDQSRAERQARQAARLAAKQEAQAAAAAAAKAAVASDKP

KTSETK

>A1A7T1

MNKRIECVMLKMSLYVIILLFSLQFSAAITGKESEVVSPLLMDVNPSLTMENISELSTSS

EPSQQGVFPVICTRLHPGSVMKRQLLTGWGPVFIIGDDPFSLRWMSEHLEILKSLNALGL

VVNVESVERMEVLQQRADGLLLLPVICDNFVQALQLNAYPVLITEMEISQ

>A1AAK4

MEGTTMAQIAIFKQIFDKVRNNLNYHWFYSELKRHNVSHYIYYLATENIHLVLENDNTVL

IKGQGKVVNVRFSKNKCLIEATLKGFKSGELSFYEYRKNLATAGVFRWITNIHENKRYYY

TFDNSLLFTENIQNTTQIFPH

>A1ADL5

MAEIIPMTEEQKFQLEIYKLVMNQNAAAEEAFQFIGTDELKLELFKIHFQSGGANSDITT

RTIEAVRKSKEALDLFTTGA

>A1AE27

MSKIDYQKLREIAEKTKIAGEAPVMPFDQRINALNDFMKHFSPDIALALLDERERNQQYI

KRRDQENEDIALTVGKLRVELEAAEKRIAELEAEPVSQTYKLNELSGNYPVTPDGWISCS

ERMPAQDDWILIYSKHGEYMAGQVQGEYVELSDGTLSWLGNALYWMPLPEPPQEVK

>A1AEL5

MYLRPDEVARVLEKVGFTVDVVTQKAYGYRRGENYVYVNREARMGRTALVIHPTLKERSS

TLAEPASDIKTCDHYQQFPLYLAGERHEHYGIPHGFSSRVALERYLNGLFGEAS

>A1AF66

MNIFTEAAKLEEQNCPFAMAQIVDSRGSTPRHSAQMLVRADGSIVGTIGGGMVERKVIEE

SLQALQERKPRLFHGRMARNGADAVGSDCGGAMSVFISVHGMRPRLVLIGAGHVNRAIAQ

SAALLGFDIAVADIYRESLNPELFPPSTTLLHAESFGAAVEALDIRPDNFVLIATNNQDR

EALDKLIEQPIAWLGLLASRRKVQLFLRQLREKGVAEEHIARLHAPVGYNIGAETPQEIA

ISVLAEILQVKNNAPGGLMMKPSHPSGHQLVVIRGAGDIASGVALRLYHAGFKVIMLEVE

KPTVIRCTVAFAQAVFDGEMTVEGVTARLATSSAEAMKLTERGFIPVMVDPTCSLLDELK

PLCVVDAILAKQNLGTRADMAPVTIALGPGFTAGKDCHAVIETNRGHWLGQVIYSGCAQE

NTGVPGNIMGHTTRRVIRAPAAGIMRSNVKLGDLVKEGDVIAWIGEHEIKAPLTGMVRGL

LNDGLAVVGGFKIGDIDPRGETADFTSVSDKARAIGGGVLEALMMLMHQGVKATKEVLEV

A

>A1AM41

MSEQSLLWVAFGLLMTIMLAIDLGLNRRAHKVSFRQALSWSIIWVSLALAFNAGIYTVLG

KTKALEFFAGYIIEKSLSVDNLFVFIMIFGYFNIRGEHQARILKWGIIGALVLRGLFIFT

GIELLTNFHWLFYLFGALLLFTAWKMAFGGNDEVDPENNLLVKLARRLFPFTKRVRGDWF

FTRRQGMLVASPLFLSLVMVESSDVVFAIDSIPAIFAVTLDPFIVFSSNIFAIMGLRALY

FLLAGMIGMFAYLKLAISFILAFVGGKMIATASGFHIPIQVSLTIIFTTLALAVLASLLA

DARNNRQIAHLENK

>A1AND6

MNLNIILWIGGMLFSLGIFALKVGLGLGYGRVGRRGIALTLGGYMGLFMLIAVAAERLMR

ILQPILAKGPWLHTLLATGMIAWGMVVIFGKRHEHQAADCCERPPRARLLMLVPCPVCLT

AMTFSIWAALNAIKLPPLLTGLCLGSAFALMALLVALIARPRSAGASETSLGMAMIVVGL

YFIASLFLPAKIEAARGMYSSFISENRIAQSTDATGVMIFILLLALAGYLAGRGGKGERG

>A1ANF2

MKLIRLIPLFVFCMTSVAQGHMLWLAPAEHSLKSGGTTTIDIGWGHSYPGGEKLKEENIE

GVFAVDSSGRRTQLERIAPARYRFAPRSKGNHTIVVTQRPGFMSTTPDGRRMGSRREHGD

AVSCMHFAMSGKTVISVGSKDRKRSGRPILPFEIIPLKGTDKARVGDEVSLMLYFNGKPL

PNVKLKAVDADSARRKEGSWAQEAVSDSKGVARIRLGAKGQWLITAHYEKPYQDTAVCDK

DMYLTTLTLTIK

>A1AUM4

MKSFIAACCMLLLLTTAAHAVRIKDLASFEGVRENQLVGYGLVVGLNGTGDSDQARLQLQ

SVATMLERMGSTISASQIKLKNVAAVMVTATLPPFAKQGNQMDVTVSSLGDSKSIAGGTL

IMTPLKGADNQVYAVAQGSVLTNSFAFGGQAATAQKNHPTAGRIPSGALVERELPNTLAG

KSQLRLNLAQADFTTASRVASAVNGKFGAGVATTGDPGAVIIQIPGNYTSRVIDFVAAVE

TLDVLPDTVAKVVLNERTGTIVMGERVRISTVAVSHGNLSLVIKETLQVSQPAPLSSRGE

TQVVPRTQVKVEEEARRLMLLQQGATIGDVVRALNTLGVTPRDLIGILQAIKAAGALQAE

LVII

>A1AWQ7

MFWQEDTKKEHFTLPETIQDAVFNIFAKILPINHSFLLAQTLLKHLPWLDEVDAGIFNIS

VADGNGWTQNHKDGFYYPSKRSKLTIRVPKDKLYETQQLLGKTLDLGKYQIKIIKSLKPK

LLSDMPVLFSKNIACNEAMSEDDFLQVTFEQLKILGISVKKMMVGLEKNIKTDTHIIHTR

SLMIADLKKDESVLLQEKGLGDYRLLGCGLFIPHKDITSI

>A1B020

MRVQMLGLCRFSYLGGRGFQVMHETLAERRAFLYDPERLARRWFWFENVTLPGLLAQTDP

DFTLVLMTGPDLPEPWLSRLRELAGIMPQARLELVPPMEKHLDACMAAIAPHIEPDADVV

GHFRQDDDDAVAIDYIRDARTDFAAMRPLWERRRRLSCDYSRGLVLKVTRDGVSVEQRVI
HNASAGLTVYLPPDAQRSAVHFQHWKIGLSMPGVTLAGKAMFVRSLNHDNDSGAIGAGYP
CPDDPAGRGRILVERFRIDLDTLDRAARDFGLPGADRPR

>A1B306

MKILFVHQNFPAQFLHLAPALAARGHHVMSLTDEKNPRPSPVRVVRYKTPPELNLSPMLG
RTYSEMAERGWLAARGCRALRDRHDYTPDLIFGHSGWGETLFLREIWPDAKLLVYAELMY
RTRGHDVGFDPEISPDSDEARVNTVARSAHLIQGLVQADAGLAPTRYQADSFPPELRRKL
TVIHDGIDTVKVCPNPLAEFPLPDGRKLRAGDEVLTYVSRSLEPYRGFHRFMRALPEVLR
ARPNAQVVLVGGEGVSYGGLPKDAAGWKEKLLAELDGQLDLSRVHFMGRVPYPQYLALLQ
VSRVHCYLTYPFVLSWSLTEAMAAGCYVVGSDTEPVRELVRDGKNGRLVPFFDQSALEVA
LIRGLAGDPDAARLQAAARETILQGYDLHRHSLPQLVDWVESFAS

>A1B4D4

MSDLLVIAFDDEATGFELRTELVKMQKEYLIELEDAVVVTRPSADDIQLHQAVNLTAAGA
LGGGFWGTLVGLIFLNPLIGAAVGAGAGAIAGKLSDIGINDDFMRELGQSIPPGGSAVFI
LVRKMTADKVLARLESFHLRGRVMQTSLPEAEEERLREAFAGGKLGSALGMPQVDRPAPG
AAPAAGSEPSPSA

>A1B586

MTRGGRIKDRDTPERRCIVTGEVQPKAGLIRFVADPDGEVVPDLAEKLPGRGFWVVADRQ
ALDKAAAKGLFSRGAKARVTAPPELLAIIETGLARRVTDTLSLARKAGLAVAGFEKVKDW
LAAGKAKVLLQASDGSERGKGKLWTPPGGRWFGCMTASELGLSFGRDHVIHSALAPGGLT
EKLIRDASRLTGLREHDGGNAAAGKE

>A1B8Y6

MSDFAVIWDWLAFAIRWTHVVTAICWIGSSFYFVALDLGLRKVPGLPPGAHGEEWQVHGG
GFYHIQKYLVAPERMPEHLIWFKWESYMTWISGAALLMVTYWVGSELFLIDPAKMDLAPW
QAILISAGSLSIGWLIYTNLCKSKLGETPTLLMLILFGLLMVMGWAYNQVFTGRAVMLHL

GAFTATIMTANVFFVIMPNQRIVVADLKAGRPADPKYGKIAKLRSTHNNYLTLPVVFLML

SNHYPLAFATEYNWVIAGLIFLMGVTIRHFFNTMHAGGGMRRWTWAVTTILFVIVMWLST

APMFRQTLEEAEARVLTPTQQAMVDVAGWDDAYNAVMGRCSMCHAREPSYEGIHTAPKGV

LLETPDDITRAAREIYIQAGVTDAMPPANVSFMEPQERQAIIDWYRSLPRTFALN

>A1BET7

MQPQLSRPFTKDNQVRTSKSGPWSGNAAHKAEKYFITAAKRGKNDNLQLEISPASGRRKL

SPTTEMINKIISGEIELFVLTTQPDIAINLAQKVLDNENRYVIDFDKRGVKWTMRDIPVF

YDSLHRELCVEIDRRTYTLNEFFK

>A1BH82

MSRFFERYEAEPSRFFDEVISHEGRPRAHYNKLLNRFSQFSSDDIKARRQILNIFFRNQG

ITFTVYGLEEGIERIFPFDMVPRVLPAHEWKIIEKGLEQRITALNKFLRDIYHHQKILKD

KIIPAELVLGSQHFRREFIGVNPPLGIYIHVAGSDIIRDGEGNYLVLEDNLRTPSGVSYM

LQNRQAMKRAFPVLFEKYQVRPIENYPQELLRTLQEISPVTRREPNVVLLTPGIYNSAYF

EHSFLARQMGIELTEGRDLVVNNNKVYTRTSRGLERVDVIYRRVDDAFLDPLVFRPDSKL

GVAGLINAYRKGNVALANAIGTGVADDKVIYSFVPKMIKYYLGEDPILQNVPTWLASNPS

DLKYILANLGSLVVKAANESGGYGMLIGPESTAEQQEKFAELIVSNPRNYIAQPTISLSR

HPSFYNDTDLCGCHIDLRPYVLSGKTTTIVPGGLTRVALKRGSLVVNSSQGGGSKDTWVV

DE

>A1BHM7

MALFGTKDTTTAHSDYEIILEGGSSSWGKVKGRAKVNVPPASPLLPADCNVKINVKPLDP

AKGFVRFSAVIESIVDSTKNKLVVEADIANETKERRICVGEGSVTVGDFSHSFSFEGSVV

NLFYYRSDAVRRNVPNPIYMQGRQFHDIIMKVPLDNNDVIDTWEGTVKALQSTGSFNDWI

REFWFIGPAFTALNEGGQRISRIEVNSIGTQSGEKGPVGVSRWRFSHGGSGIVDSISRWA

ELFPSDKLNKPASVEAGFRSDSQGIEVKVDGEFPGVSVDAGGGLRRILNHPLIPLVHHGM

VGKFNDFTVDTQLKIVLPKGYKVRYAAPQFRSQNLEEYRWSGGAYARWVEHVCKGGTGQF

EVLYAQ

>A1BY90

MESVFDLNKLVDSEMAEKAKTGVPKAADGTALPDIDAELQKRLDDLRAFLRKTQSASEIT

NPGFELGRPALKESTFNSDKHTHIYGKWSIDQLSRIVPKKISNNMATAEEMAKVQITLEG

LGVPTEQGAEVLLQVAIYCKDVSSSSFMDSSGTFDWKGGSILSDSVIAALRKDDNTLRRV

CRLYAPITWNFMLTHKAPPSDWAAMGFKYDDRFAAFDCFEYVENPAAIQPAEGLIRKPTP

SEKIAHNTYKRLALDRSNRNELYSNLNTEVTGGTLGPEISRNFNHAKK

>A1C4N7

MGKRKKSSRQPQQPKKKEPLPTTFACLFCNHENSVIVKLDKKLGLGNLTCKVCGQRFQTG

INYLSAAVDVYSDWVDACDAVAKDTANRYVDGHDNHNGEDNGIRGIHSHEHSLPSSKWGD

GPGDGEEIASYLDDE

>A1C4U3

MSAPSMAPTPAPASLAPALAAKPAVSPSPGPGTPGSITTKEWVIPPRPKPGRKPATDTPP

TKRKAQNRAAQRAFRERRAARVNELEDQIKKIEDEHDIHIAAFKEQIGNLSCEVEQCRSE

MTWWRDRCHTLEKEVSVERSAKEALVKEFRLSLSDKNAASSDAVPFPRATSSRPENDGVP

ANNASHDDGHHSDNREEVPLGCNDCSTSHCQCIEDAFAMPGVLAQEESRRPGSTKPGRLE

PEIKPDPEEMEIDFTARFATMKPQDHSPTSVSSPAVDPCGFCQDGTPCICAEMAAQQEEN

ERRNTFENNRLAPIQNMSQFTPPPSDGDVRSEMTLPSLSQATNPCANGPGTCAQCLADPR

STLFCKTLAASRSASGTPSGCCGGKGAGGGCCQSRSSNPSRSGSTGNANSIPGPSTTPSL

TLSCADAFTTLSRHPSFTRATDDISNWLPKLHTLPNPRDIPLSDRGTPRAAMEVEAASVM

GVLRYFDRRFADK

>A1C8Y8

MATDQKAMLVPPKRANTDYPLIDSDPHLRRVFGYARSSDYAIAGGMAAASPLAFWAMERV

SPSHVGRGGFAPVMRLATAIGLIGGLHVLYQRSCNRFYGFTENSREVEMDMKEMVDKVKK

GEPLYGTSQVSSYLQGVAARNSRYSQLFIHVLPWFNLVNHSQHGIDTAKYYQQAEQELET

ERLANAGSA

>A1CA14

MEPPETPAPVFAIRAFKSALFGTPGAEDEEQSDREAKTDSKQENRSRSKSEAPKFLAPDR

ATNVIATGKVDLDTGMNAMASPTKSILVTPGTASNRRKTVSFGDGVVDNERKRDDIFNNK

SLRTPMNLSGNLSSQWISGSSDGKDKPRSKLTQALLDSRDKSPNETTDRKIDDDPFATNE

TKMVPKSTKAEENDDTINLEDPRSQSGKYWKAEFDNYRAKTTNEIRKLIQYRSAAKKFAL

MKETEASRLAKKLKEEESKVAEMERHVTQLASTMVTENAKADREQLVQDLTKQTALAMQY

KHRVNMLRKLLERHGVVDGDLDDSVDKSDAEGSPEKVSQELRKTQQALNQANAKIDEMKR

QQSEVDKLKELVQNSEQKASELARENLTLKQSLARVKQEMSKYEGRRKAKEAKLKQREIK

LEGRIQEYRQRLKTASQEHREMEEDLRGSFDEERRRMQDQIDLLKLKVAALEKVPDDRVY

SRHSYSPRKEFTGVQVYDFGRISPRKADIDETQDIDEPPSPSPRAKDRRMRSTQNPTVEL

DIVKAAQALGLEGVHSHQFLTGESPYKTAASLQRATAQETDLIPPSSPPEQLFDDSTGAL

AMKPMYSQRDYRAHQPTHTTIDSLARHLASLDSQQPRTQRSKSRRAPTKYSLDTLSGLPD

SRAAERPGKRHSIGALQRESIPPERMLAAQARLKRKDQTRKSREVLREHAKTYNT

>A1CB33

MSTGNPAADDSHAGDSHSGADSRRPKKLIFAPGDIAATPDLKVTATETKEPASPAPDAPQ

TLAERLRSEVSTPDSTMESQIVSHPARAHQFVSNPPLTISQMHPTNPLHQFNTWFRDRRL

APSSAPETCTLATASLPSGRVSARVVYLKELDERGWVVYSNWGSREGKGGQVFGAEAETG

DFPAAIPRPEEAESLLEELPGGNRWAALTFSWSGLERQVRIEGMLEPLSRQESEMYWETR

ERGSQIGAWASWQSKVLWSAEPETMVDRRRKSVAAAAAAQGQVAATSAAACAEIPADIDE

TDIDDGRALLEQRVQQMEKRFAGMEKIPLPPFWGGVRLVPESVEFWQGRRSRLHDRFRYV

RVKEAGDESSFKWRIERLSP

>A1CCN4

MKNFAPSLALSLLLPTVQAQQTMWGQCGGAGWSGATDCVAGGVCSTQNAYYAQCLPGATT

ATTLSTTSKGTTTTTTSSTTSTGGGSSSTTTKTSTSAGPTVTGSPSGNPFSGYQQYANPY

YSSEVHTLAIPSMTGALAVKASAVADVPSFVWLDVAAKVPTMGTYLENIRAKNKAGANPP

VAGIFVVYDLPDRDCAALASNGEYAIADGGIAKYKAYIDAIRAQLLKYPDVHTILVIEPD

SLANLITNINVAKCSGAKDAYLECINYALKQLNLPNVAMYIDAGHGGWLGWDANIGPAAE

MYAKVYKDADAPAALRGLAVNVANYNAWTIDTCPSYTQGNKNCDEKRYIHALYPLLKAAG

WDARFIMDTGRNGVQPTKQQAQGDWCNVIGTGFGIRPSSETGDDLLDAFVWVKPGAESDG

TSDTTAARYDAHCGYTDALKPAPEAGQWFQAYFEQLLTNANPAF

>A1CD79

MASPLIRLTPHPFSSLPAHPSLDSSSSSSPSRPSIQEFLRTILSEAQTLLADTIPETFEV

DRKLRPSPPATANVQLWRRTLRPAPGAASSRKDEFWACRKSIHEDAVVEGSASFDEFRAG

LRDNHAENERAYTPSVAALNRLLDWPIEPEIDGWERVVMQVNLITHKFHPSVLISPRTFI

SLLVSADLPDPVAKGFVTVQIPLTADPVETIPRALREKIAASAPRNAIFASYASVEQVLS

LPFAVRSNSDARSSRKYVAQVEWTMATTSDAGGAIPRWVQQSWTLGGVPRAITADVGLFI

KWTGHRRITSHPATSTTPTTVPAARTKPAEPTEEQDT

>A1CIB7

MAEPSAATEKAPNASTKPTPKPVNPAFRMLGMPNLRFKLPSRNWMIFFTITGSLSAAIIY

DRREKRRVQQKWSDLVAHISKEILPIDETRRKLTVYLAAPPGDGLRVARDHFKEYVKPIL

VAAALDYVVIEGRREGDVRAMLAERIRKHRRKAGEPSSVVEEIGNEDIIADARQRIGVTE

EPGPKGDLVIGRHTWREYIRGLHEGWLGPLDPPLPPVTPTEEPSPSAEVPAASEPADGAP

VAEFVEETTEKKEEPENKDEKPAKPTGPTPAYIFPAEYSSHSLPATLPQSFESSVPIQFP

HLLGFLNTPIRLYRYLNRRHLADDIGREVAGIVLASSSRPYQDNSFSSDSELSGASVEAG

ASPVSTSDDLTASSSYEQQTVLEHEESEWHKSVHKKDETNPDKEREWLNEIVLDPRIASR

MQRSLLSPEDEARSQRLAESTEYILGEERPIPVPFWKRMWVKYGYGEDEEALKMKPIIGN

LDGEDGL

>A1CNU9

MAHGPFERKPLGLDLPHAPTPDSPPGIAALFVIRFDIKAGYVISWKRAIPGVEIEGVVEY

RSLPSGLHNVSEDLIYFVYEQYAGISAFVNLPADEAERNANMFAVGVLVPLGSGRLGKSW

RHAPKLKELAHVYAKDMSNTQILSDYWETYEMRTNGASAIPPDSPLESPLSLRFRERPDS

MHRNRTFSDALVLEASRPALTPFHPASSLPEFLDCFGPLIFPLYRAALLRKRILLMAEAP

VHIPCNFDAVYDLSLLASLPYSLLPLLPPAGSPPPRPRPLFNVGIHDIPYLSSFVGVSPS

SEPDAAWIACSTDSVLSMKSELFDVLVTLPPVHSKNAAEKVFPKISVLPDRTAHHNSSQA

IYLKATQRDARRYSILRKGLRHLSHSQDTQSGSDEDSDAASTYSSSPVVEPLSWTRLAYT

SFIWWASAGEKRDGLSEEEEEEHQIEQDTRLLASVESMPTPSSGSVGRQSMQAEGAQQPP

EIAMVAYFRRLTTQIFVTLSDVIARQDSRDGEDDEDVDAPYEDGPADDDNDDTEPSVHIG

RQATLEDDSRAPLLESGAKRSDGDDEPVRITSEDMTEMGLDVWSASDRIFVEELVQSWWG

RKAYIDSARIKCCGISIV

>A1CNV4

MVKRKLGALEKVEADLPNLQHKVRRDPKSYIEDFRAQHYQYESHREIFMAAPTAATDTGI

ISLRELIDFLAHVADCYPTITKDFPQQLIDILTQHHLILEPELREKIVGSLVLLRKKELL

DSATLLQTLFPILISTPSKTLRALIFQKILMDLRTSNSKTINHKLNRTMQTVLFNLVTSD

RTSSKGLWAIKITRELWKRQIWTDAKAVEVMKEASLSENEKVIVGGVRFFLGGDKEREEM

EDESSDEEAIDLGRVKHQVGINKKTRKKSRAVEKAKATVKKKERKKNQPHPLNFSALHLL

HDPQGFAENLFSKHLQNSKSKLNLDQKLQVLQLVSRLVGLHRLHIMHLYSYFQKYLTPRQ

PSVTSFLASLAQASHDLVPPDVLEPLVLKIANEFVSEAAASEVATAGLNAIREICVRQPL

AMNETLLQDLVMYRKSKDKGVVMAARGLLSLYRDVAADMLKRRDRGKEASMGLRSGEKKE

RRFGEQETGEIEGLELLEKWKEEERRKRRLEKGLPSDAEDDEDDEAEDEAAWAKWNVEDD

EDSDGSGGWIDVQSDAEIELSDSDDDEERPSKKAKSEGEEDNAQATGEQTEANKLKLATT

RILTPADLAKLAELRNEAAVNALLPKKHRSGQNNGPSRHADDPLTAAEIEGLAALSAGKS

TREERIAHAKEGKQDRSEYKSVTARRKERKEEQGKSSTNKEKERRKNIFMTLGKAKGKGK

RSLVETRNVLRAHQERRKRGGRRGNNG

>A1CQK2

MDAYPEDYVVHNLPFLLLSGLEADTQDDNESSASDYPLLQEKGPTIYSDFPPLSGPTADE

LRSNLLEEDVSRVPWDAGQPTGARSSGIGYRIKSVGRSYRLPPRKADPPPLSPPTSPTGG

QQNDAAPAPSFVLHSPISPLTPSSPTFPDGLLTPLWVSKHQNLVPAAVINFFPFCLDSNM

SSLRDNQLKIEINGLKQEWTASGYNTRFIVVLLPEEGAGDYIEEMEERVASIRRATNLDQ

KSIILLPPDANAAELKELTRGLLSLIQPLVTEYYRDLSKHARRKRNRSTIPPPTAPPTSG

TSQTLSLQGWNVRYEFKLGIFAEFRQEIDAALRNYESAYETLFGQDVIENIAGWSPRFND

ARLLSDALAIRIIRCLLWTGQTSAAVRSWIDHRTRSQDILNRRGKGTRNYGWEAWEARWS

MVMAQLIRQAGIPGISDDRSDKDGAAEQSIIFVHRRKSASANEVTYPWEQLHHEGYWLYR

SAKHAMARRTLAEQIPDEDRISPGQSPASQIANKSYMYDTYLAPGPHIEASGAGSTGFNH

SQQILNALKGALEQFAKRKQTRKIESLSLEIAEEYMRVNSWAEAYEMLRPLWPSLTWRHS

GWWLLMAKFGWALRECALRMQDSEMALRVDWELMHRVFQPRPGWHYSIHRGLAEFPDTQP

KASVVLKAEDVISCVTATFVFEKSEGNVGEPLKGQLILTSCAQRSSDPIRLSEVKIVFEG

CLRPLKIQADQDVNADAKTPCIISSLTLREPTSADSTSLNSPTIGLTALVGLGDLTLGPS

QTKVFNVTSIPREAGESRVASITLLIDEEKFDLACAITELARQEAYWWQETTHGAVRRRV

GKGRDTSRCKIMPKPPKIRIKTSGIKSTYYTNERVILQMDVHNEEDEAADVTAEIRLFGQ

SKLGGQILWLEDEDETESHRSRDSSPIEGAHHFLRRSIGIMERSSRRDLALVLTDTLEPS

TYELEINCVYNLVSDIQTPIIATHRVTVSVVRPFEANYEFLPRLHPEPWPDFFTMDEDSL

EDDAVAKPRGLHQRWCLNSKVVSFALEPLIIEKISVVLLNLGSGAICNIGPEMVVSADEP

EIRPEELRESNFTLDLQKLVLGDRRPTALNLALEIQWRRSMEDTASPADHTSATTTTTLP

IPRFIVPTGEPRILASAVASQTLNGVIHLDYTLENPSTHFLTFNLAMEASENFAFSGPKT

MVVQLVPLSRHTVRYNLLASKRGLWIQPQLIVVDTYFNKTLRVQPTEEMRSDKKGILVWV

DADD

>A1CRH4

MLSSFHRRCIGVAYKRSNSDFRGQNIKGDADWRRTNWGNSASPRAASPLTSGAESGPDTK

SAGAGAGASTSSSINRPSSPTPPGGPRAAMRRRAAADHKESLRNARPSSTRAAGAGGSSG

TMLKLYTDESPGLRVDPVVVLVLSLGFIFSVVGLHVIAKITRKFSS

>A1CRI7

MHVEGMPTRGPGRPIGSSRQSTPAKSGRPPGRPPGRPPGRPPGRPPGRPLGRPGRPPGRP

PLNPIVDREGTPDVFKMAIMKLRNVGALDEEIKEPSSMDWQAERPGLEEHLQNITMQPSY

VPRAGEVVLWTPNFDGDLSWNPEHACVEIFDPNTNRWLGVPEWRAGIVGQPPEEEIVLRD

LVETASKGWGVNYSGFRVETFPDPHSADKSYSLQYKYVHLKCIKPFNAFELFLQGIPRET

LHPSIEYAMTIMSSFSLLEKFHFRGRWPNASIYCRGIFIGAELLILGDAIRLKPTGYTPE

SAHKPVVTDVMVIDEIRLDLIQCTDDIKSDQLAEKYHVRIGGKVYTNNRQRASTETDPPK

PPPRPLSPEKVLSTFQYIGMGGYGEWYSLYSGNMVDISQAMVIGRCYEPDAMRLLYGSLS

LGRDLRGVLSGRKYSRRTDERIPEGQDWLWGDFRTQTLAIESLNGEDVGHYSEVRDVKMW

RANLKVLAGKATAADFRDAKIPGELGRPSHKSRSTFDQVRKTSKMVSVGLGAAITDVSNN

VSSADESNVQLQLGTDDEADEESEEEEDFTLRVEDLRGGTEESEGGDYAPGNHEHTAKRP

KHE

>A1CU98

MSKVVRSVKNVTKGYSSVQVKVRNATSNDPWGPTGTEMAEIAGMTFSSPTDFYEIMDMLD

KRLNDKGKNWRHVLKSLKVLDYCLHEGSELVVTWARKNVYIIKTLREFQYVDEDGRDVGQ

NVRVAAKELTTLILDEDRLRSERSDRKLWKSRVNGLDEYQSHGAEPSRRSDRRERRRPAG

DDEDVEYRLAIEASKAEAEEERKRRERQTMMAEDDEDLAKAIKLSKEEEELRKRELEESN

AQSLFDDTPVPTAQAQPTGYNQGYQQQGAVDWFGNPINPQQPLSTGYLNNQYAQATGFQN

QPTGMPNGYANGFQSQPNAFDHNPYGQLQNNFLQPQATLQPQQTAFSPNNPYGTDVFSQQ

QQQQQGQENFSPSGSNNPWAHNGAPQANTLQPMPTGSNNPFAARTQFQARPHTSTGPPSL

NTLAEERATNQFTSSNPITNFQAPAFTAAPAPPKSTPPQMNNPHHARLNALLSTGEGQDT

FGNVGDLRIPAQHTAPGTFVNSAGQGLDRLRATHTGNNPFYNQQQFVPQSTGFAQSTNNP

WGGQQSYQQQHQAGGSLIDL

>A1CUM3

MVKDKSTVINEFTDLVNMTPNELRDWLKEEQSQSAGWKRDSDETVGHESGRRIVEILEQN

PSKDPGGYSDDDIAHMRKVVSYCKRHLAREGHVKRDTGSKSYKSLKNWGHDPLKEEEEEE

E

>A1CYI5

MSLRGFHTTCDTIVLKDKHILSSRCKRPDGTYSFSEIDLNDCLGNSNGKFVWGGESFADS

ASQVHLALEGGEGRPMLHAQLNDRHGSVQSASVNLGECIQNEAGGLEYMHC

>A1D1H0

MPGLLRKLIIIAAVDGLILQPHGNGGRNSNNYEPQSVRIDYKTNKISSLSAPASDPAVRK

DAGLEAYGLVGLLSVASYSFLISITQRQQVAQIQGKPIYSITNVAIIPLSSQADASRAIS

QAKETLLQGEAGVGETASEGSISDNETDGGEPDINSEPSSPTQDDPSPRDRKGSIAEDVI

GKRVRFGRFAASWLSRKTLGLPGLSNVGQDTTERLLGNEKELGTELQPPIAKLEKSPAAS

DAPGAGADSPLESGKEPSDPTVELLPKLLRYTRLIYSSQNFFFAYDYDLTRFFGAQEARN

DHIPMHKVVDELYFWNKNLMNPFIKADAHSFILPLVQGFVGQREFTVSAKPEQPDSNVAE

EHTEGRMLGEKDEAQSIKVDAEKRDFLLTLISRRSVKRPGLRYLRRGVDDEGNTANTVET

EQILSVPGWDPGHNVYSYLQVRGSIPLYFSQSPYAFRPVPVLHHSTETNQLAFDRHFRNL

ARKYGKLQAVSLIDKQAGELKLGNEYEKYARVLNESGGIDGVPLGMEWFDFHNECRGMKF

ENVSRLVKRLESVLNEYGDTIVRNGTVTQSQTGIVRTNCMDCLDRTGVAQCAFGQWALER

ELKLEGIDIDLSGDSSTQWFNTLWADNGDAISKQYSSTAALKGDYTRTRKRDYRGALNDL

GLTLSRYYNNIVNDYFSQACIDYLLGNVSTRIFAEFAMEMRTADPGISVQKLRRSAIDTS

CKIVISDQSEEFLGGWTMLTPRQPNTLRTLPFEEAVLLLTDAAIYSCRFDWNTDKVTSFE

RIDLRSISRLNYGTYITSILTESQANEQGNVGLVIEYREGGENAWRVNTRSLKSDVDTKA

QNSNAQASGERGLYSWFRGGTQSTTRFIAFKALPQSNSVAKNNTAGSGTVSETDWVRSIC

EEIERAMMAGEGPRPSEEGKPPSVIENSEIISLEDAKKRTGILEHLVYDIKKLVWA

>A1D4L6

MEYTTRGVCGQDGCRETRYYLDNGLWFCRRGHQQEEDPDDFGTQGKTSRVKKAIYQLILW

KQCHALVQGRGFPAELEVVVRDLWALRLESLSERLNDPAEDDREPELFSSQPATTLDESE

GAFKLSGRAAQWPRLIDTIGLCYLGCLLMRLPVTIGEFHRMLMREDVPFIRVLRLIPREM

RDKLPQEYIALLETTRLIKAEHLHKATLELSLLYHNKFGVQFPSLNTPATLYRYIKRLAL

PVDIYPAVKRLQSLVSFTFEFPTTVPGRSRPLHLPEVQLITLIVISTKLFFPFDDIKRYP

VSTREPSAQVLDWKLWEQVQRHFDRRETAGGRIGKGNEILVTEKDVFNMTPDQLDEYMDW

YETSWLDHSRVSNPLAEFFPIGPTGSEGQTVSDSTEGDDEAMHAMLETVTSQLKPRKVSD

PKADVARPGTSYPRYRTESELPETARSFYEIAAKVAGVSLSTLVRAVSQAETKISRWLDD

KRRIEHHGDPAEMDVTGEAGTEDLEAMEDEDMSGLSDES

>A1D707

MATQATQTAPVIPPRPSRSPQSNLAPKSISDMPKIPPRPNRRVERSVSPLRGSYAPSPLN

EPPNSAGLTRTVSNDLPQRPPSVTIPSLGEEGIEYQNLDIGNLSDSHHQIGTPAETRNVG

SDLKIHAPRPSLPSSSAKAKVQAVTRTDSRQAAAAGLGVSASPVPDDHHERSSRSLHSRA

SGSRADSSTASSDRRRSLQLPADEHGIPEIGQRVPMYPNAGDVQAPSPSPYQLEHGGQRQ

GRHHHRTRSGREASLPPGSYGLHGHGVPATDKFEKAWYEKHPDEYVKVEHGQYGPGVGTP

RPDWALSSDDLNKIVRSSAVTGSGLGTSPNVAGTPDEEVGYIATDEYTHRMATPAPESRR

SSRLIGESPLRNTDVPASEVGVEREGRKSEDAGVIHVDDPYHPLHHPDGFAPTPAPEEQS

HEMGVGIEDEDEPILAADEVRPESAYQHPAVSPTFDRRPSEYDGRSRAPSVNHSRSNSRS

ATHQGGMPALVRYNSRDEREETHTPLDDVEEYEPLFPEDDRDKKSVSAADRFKQRPEMLK

HRFPSEDIWEDSPNSLQLHATVSTPDIPKHEAFETPEQESFRRSHAPHLDPRQVAEHILE

SEESKEKTQPRPDICKQRFPSRDIWEDAPESQRLVTTIEPSEESEVKSPDVPSKPAIPSR

PQKLSEQTPAVDASSKPATSPTEKRQPPSIPGRPKPQIPARPAKPITRGAGEEVPAKEKP

AVPVRPNGSKIAALKAGFLSDLNSRLQLGPQAPKPQEKKEEEAPAEKAPLSDARKGRARG

PARRKPASENVSTRLPTIPEIKITETWNVWQVDEDGNLTVETGVKAKQPEPAAPAVSTSK

DSMAPALSKNIVGESTDPSPVTATKESAPETAETIPSTDGDAAAVSATEPTKPDESEVQQ

KSADAQSGITPSTSPPVDDAIEAMAATADGKRASEGSVLPEEQ

>A1D8H8

MQITKISLFLFVGIGVVASPIHAESDGLNARAVNAADLEYKGECFTKDNTCKYKIDGKTY

LAKCPSAANTKCEKDGNKCTYDSYNRKVKCDFRH

>A1D9R8

MPRARSRSRSREPSEGRDPENVMRGYKATLRNPNVSQEAKQHAQQELDRYESGDATSKSE

EDRHASNVKRGLKAATHNPNVTDMGKKQARDKLQAMGEQPEEPGD

>A1DA36

MKLLAALASALAWGVSLAEAKAVFAHYMVGNTKSLGLIDWRHEMQAAQAAGIDAFVLNMA

SKDPTNNIALPMAFTAADDMGFQLLFSFDYAGNGPWDKSIVIDMIKEYGAKDTYFKTAGK

PFVSTFEGPNNADDWKDIKKETNCFFMPDWSSVGAQPAVHLGDGIADGLFSWDAWPKGPA

NMTTYPDASYYDFLGSKPYMMPISPWFYTNLPGYGKNWLWRGDDMWFQRWQQAISLDRQP

DFIEIISWNDYGESHYIGPLDDRQYEAFDIGRAPFNYVKDMPHDGWRETLPYYISMYKSG

TATVTEERLVAWYRVNKNGACSDGGTTGNTANQLQFEYSPNVMMEDRVFYDVLLTSNAQV

QVSIGGVVQAGGWDQEPYGGVGVYHGSVPIGMASGQVVVTVKRGGTTIATITGASITSSC

NGGLNNYNPWVGSARGAPIIPVTTTGDLSKLDCVKGFGVFDFIGVCDFACANGYCPSAAC

TCLKKGVANAPNETGLAGYPLPGKSGSFAGLCSFDCNHGYCPDSVCGQTPDDGVVLGYSP

FLPPACTGGTGAGAFQGLCDFGCHLGFCPIHACTCTSTGILVQTPPKTNVTGYYLDSSTD

DYGLCKFACEHGYCPDVCGSRPIGDDGNQGKYPTITLDPAVWTAPTAQCAPPCVLVLPPS

SLASPTTISFDPWQTSLEFGWMTTDTVDGTVTTHYTAVTVSTEISIPPVITDLISFSEVI

LTTTVDGGVPSIIIPTASVSPPPFVITPTPVADITAAPVARTIRPPPWPWSGASAMPDPS

GTSTTTPTSGPVVVVPIVTGPFPTTVFPTETASWVRDWLPEPTATQVDDGDPVPVVPCWA

WFIWSCPPNVGGIVLPGFKNPGIYPEGGPPPVGPNPPPGLTLKIPWPQITIGPDRKPTYP

DKPDPEEVSCETATASVCTTTLSYGIVAKRAAEGAAPTRAPRIPFEEYRKLTKRAVTTTT

STISFCTQVTGCGATDITTTTAIATTATPIPRVVIPHDPWSVDGIRTALQQQLGGSALDL

FESRTDQLGTMFFFVPAFTNDQTDAIKGHAQVADAYIPQGQLISYLGMARDPTSGGGQPA

SDDDWFMDTLNSTESELQERSILAKRSEIVQSNLPDVMVSLSWPYGIGPVPDQGDYRFDS

SAGEGTYVYHVDYGAQPSHPEFSGVSFLHPLLPGPYPVSGWMENDRKRHGTKCLSKAVGK

TVGIARKATVVATVWDFTKMIFEHYLDGLAKVHADISTGARGAKSVVNFSISFPQGLVSD

AFVDKLALLIREIIKLGAVFVTGSGNEAGSPNGYPALFGDPDNRNYIPELIVVGSVTGWG

FLGGHADALWVTCYAPGFFLRLATSGPTDSRDPGYQSTLAGTSYASATVAGLAAYFRGLD

DTLTTAAMVKERIVRLAYRRQPTLAYPDEDYPDNVVWNGQKWGRSIVRDCSGGSKAKRQS

NGGSCPVAFPPQPSPLTFRTGPPQPTCAGAGCGSSCAGFFCPGTPLKQNPDFLDPRNPDS

VQNPDSPYYEDWDGTITRTTTPTKTIPTTTPTPPKISSVPIGGPCRLTDECEDNCPKPGA

VQCQSGACTCWPPPPKTTPPHAAMCYDVQQCLDVYDCGSGAFMVCEPTDYSNGNGLCQCI

KGNSS

>A1DG04

MSRSATPALPLHNAQASRPSSTAPVTGVRSPSTSTSTYSLLGPEETAERLQTSLLHGLTP

AEAEIRLLRDGPNELPHEEPEPLWLRFFKQFKETLILLLLASAAISFFMGNYDDAVSITL

AVTIVVTVGFVQEYRSEKSLEALNRLVPHHAHLIRDVPPSSAPLMNNSTTATLGPDIELE

DLASKSPSSASAAIKASSTVLASELVAGDLVLFTTGDRIPADIRITAATDLTIDESNLTG

ENEPVAKYPEALRSTKAAVSHSPKIVSPPRSPFYDAPASGAVGADIRLNEQHNIAFMGTL

VRSGYGQGIVIGTGAKTEFGSISVSLQEIESPRTPLQLSMDRLGQELSYISFGVIALIVV

IGLVQGRKLLEMFTIGVSLAVAAIPEGLPIIVTVTLALGVLRMAKRGAIMRRLPSVETLG

SVNVVCSDKTGTLTLNHMAVTKMWHFDCPEPFEVHNDISSLTPGPTARTVLRIGNIANNA

RLWRVSANSPASASSAAVLSSTVDRASGAVKSRWVGQPTDVAILDLLDTFGEDDLRDRIS

RRVAETPFSSERKWMGVIIGSAQNDAPSFTGANNVAYVKGALEQVLTRCDTYLTKDGREV

ILDEPRRHTVRQAAEHMASEGLRVLAFASGAVRDTPRGGRVFGSRSGTPLSSTSQGDEDD

RYTGLVFAGLVGMNDPPRKDVHKSVRRLMAGGVRVIMITGDAETTAVAIAKKLGMPVRDS

PGSRPVLSGQDLDRMSTADLAQAISTTSIFARTSPEHKMKIVRALQSRGDVVAMTGDGVN

DAPALKKADIGIAMGKLGTDVAKEAADMILTDDDFSTILRAIEQGKGIFYNIQNFITFQL

STSVAALSLVLLSTLLGFKNPLNAMQILWINILMDGPPAQSLGVEPVDPSIMNRPPRSRT

ARVLTRPLIQRVLTSAFMIMLGTLAIYVYEMGDADDVSNPGKRSRVVTAHDTTMTFTCFV

LFDMFNALTCRSESKSVLRGELSLFGNKMFNYAVLGSLFGQACVIYVPFFQRIFQTEPLN

AAHLFRLVCISSTVFWVDEGRKYLNAVKRRRAVGVGYSVNV

>A1DJA8

MTIKNLIPIALLALVHFPPMTMAQGSACAPGGTFDLSKWKLQLPTGKAGSPDEVSASELK

GCNGYKSEYFFTSPDDGSLVMTVPGSTSTTDCVTTPNSKHCRTELRENSPSKWSPSASKN

RLFGDLLVKQITDDRVVVGQIHIDGSISTKPVCELYYSSKGDLTMGVNRCRTCGQDTFTV

GHVPVGQRFTYEIRYEKGILSVSLNGQAFQTLDTFDLDNPDSYFKAGNYNQGNGPTEVHF

FAIRVSH

>A1DNR2

MTSQTKNTAPIEHSPEKEDPAEASSWTATAGSFGRWVLTPMGFAITVYGLNVIAWGGMLF

LLLCNAAPAMCHPSCNDLYSSRRIWIEINSQILNALFCVTGFGLAPWRIRDLYLWCRWRL

GRGAGTRRKWFDRLAEIHANWFYQRLEPNGSSLLTTTLDSPSEGRGATPTPRYKLHVVIW

GNVLNTVFQICLAACMWSMNRFNRPSWTTGLFVGLACVAAGVPGILMWLEKKRIKKTNEH

PGALLLIPRSTLLGVKSHTAGSGNDAVEAA

>A1DP12

MTTDSDKAIQPATREAILLWKKLSMYRPRPAVGTEGGNTPLSKPLQRSVSSNSIDRLSKP

FKCPGSATPTRTSDKPARKRRKVNYAGAEGEVEDNSVKPWTNEERLALATRDANKFPVFK

VKDKETTFKQRFRIPLINKSSDEYNPSRPAPTLGMRQGATFVVKPLHDPSGEFAIVLYDP

TVDDIDETPESMPEDKGTEETKAKLDEPLMHKSLADILGLKKKVESRPKVPVVIDPRLAK

VLRPHQVEGVKFLYRCTTGMIDKNANGCIMADGMGLGKTLQCISLMWTLLKQSPEAGKTT

IQKCIIACPSSLVGNWANELVKWLGKDAITPFAIDGKASKAELTSQIKQWAIASGRAVVR

PVLIVSYETLRMYVDALKDSPIGLLLCDEGHRLKNKDSLTWTALNSLNVTRRVILSGTPI

QNDLSEYFALLHFANPNLLGSQNEFRKRFEIPILKGRDAAGTEEDRKKGDERLAELSGIV

NKFIIRRTNDILSKYLPIKYEHVVFCNLSRFQLDLYNHFIQSPEIKSLLRGKGSQPLKAI

GILKKLCNHPDLLDLTRDLPGCEHTFPEDYVPPEARGRDRDIKSWYSGKMMVLDRMLARI

RQDTNDKIVLISNYTQTLDLFEKLCRSRGYGSLRLDGTMNINKRQKLVDKFNNPDGEEFV

FLLSSKAGGCGLNLIGANRLVLFDPDWNPAADQQALARVWRDGQKKDCFVYRFIATGSIE

EKIFQRQSHKQSLSSCVVDSAEDVERHFSLESLRELFQFKPETRSDTHDTFKCKRCRPDG

TQYIKAPAMLYGDTSSWNHFVNDGENGQLSKIQDLLIRQETGEKDVSAVFQYISH

>A1DRN0

MYDYLIVGAGLFGSVFACEMNKRGYAVKVIEKRNHTGGNIYCEKIEGINVHKYGAHIFHT

NDKSIWDYVNKFAEFNRFTNSPIALSKGKVYNLPFNMNTFNSMWGVINPAEAKKIIEEQK

KELGNKEPSNLEEQAISLVGRDIFETLIKEYTEKQWGRPCKDLPAFIIKRLPVRFTFDNN

YFNDKYQGIPIGGYNQLIEKMLEGVDVQLNVDFFDNKDFWLSSAKKVVYTGPIDLFFDYE

FGRLEYRTLSFEHEVLNLDNYQGNAVINYIDSCHKFTRIIEHKHFEFGTQPRTVITKEFP

KEYAEGDEPYYPVNNSDNNRKYSKYKTKTEDFKNVIFGGRLAEYKYYDMHQVVASALLAV

NNEIS

>A1EGS1

EVIMTVLHAGGKFDNSLYKVSGGLHGVGVSVVNALSRRLELGIYQEGRLHRQVYHQGVPD

APLSVVGTTALRGTSIRFWPDLSIMETDTFLFDTLAHRFRELAFLNPILTIVLREEESLR

EETFHFEGGIKSYNEFLNENKKTIHEVLFFRRELPSGAQFEVAFQYQETTDNETILGFAN

NIFTKEGGTHIKGFRTALTRVINRFIKDKQLNKGEELRGEDIREGLTAVVSVRIPDPQFE

GQTKAKLGSSWVSGAMETFLAEEMQERFEEFPQIAKKIADKAIQTAMAREAARKAKELAK

RKNVLEGSNLPGKLADCQESDPAKCELYIVEGDSAGGSAKQGRDRKFQAILPLKGKILNV

EKAGGAERFVTHDEVRALITAVGCGLGNEEYSQKNLRYHKIIIITDADVDGSHIRTLL

>A1ET09

MMKLWVINMKSRFVVFGASHSEGVSSKTGAPYLIPVLFVGKPIRQWKNDKGQCLTFGLQH

QEVKFVSSDAMTRKLEQTAFPVLVTFDNEPDPEDPSRNLVIDYQVVCSLFDNVPGGKPLD

KPQPIKS

>A1HLW6

MAEMEEVREALALLLENYWIVREEAPEQYNLIRRHEAHLRRYFLEKCGWRLMATPQFYKL

EKIPAQPASWMGIQEFSHPRDYALLCCVLAFLEEKSVNEQFLLSDLCESLLALYPHEADE

RLNWESYEHRRSLVRALKFAVERGLVRLVDGDSEQFALRAESEALYEVTVLARYFLRSYP

KDLHQYKSLAELQSAELFDDEATTGRGRRARVYRQLLLTAAYNAADARPEDFLYLRNMHR

RLRDELESYTGLQFELYKDCVLLTSPERTNWCKQIFPFYQSGLHTVILHLASFCRDRWPA

GEGWQQSFSPVAFERLVGECRECYGAGWTKEYRTATLSRLAAEVLAELEGWNMAAYDPET

GFITLRPALARLAGRYPDGWPSRSEPAD

>A1HMT7

MEPVAIKRKNNLMLFLLIIFLFTAISFGATNAAFMVTQNIYHGVTVEGVPVGGLTIQEAE

RRLRTHFGERLKHPLIELVYENNKWEITASQIDFSIDTASLARKAYQVGRRGGILQRLRE

RYLAIYHSYTLPLEISYNHEKLQAVVTKIAKEVNRDAQNASLAQHKGGVTITPEIIGLKV

NVAQTVADIVSKMNRSLPVRSELAVEKVVPPILAQDLVAIDGIIAAYTTQFDPRDENRTQ

NIQLAAKSINGVLVRPGQVFSFNAHVGPRLAENGYREAPVFVDGRLSTDWGGGVCQVSST

LYNAVLLADLGIEERTAHFRPPGYVPLGQDATVADNQLDFKFRNTTANNIYLTTEVTGNQ

LTVYIFGKRMPNSPEIRIITADKQILEPKTVIKQDPTLEMGKEVIESEGQKGYQVTTYRI

KLRNGQEIAREFLASDEYNPVDRLIRVGTKTSPKEPSK

>A1HMU9

MITTIRVLDETTANKIAAGEVVERPASVVKELVENSIDAQSRSIEVEIVDGGINYIRVSD

DGIGMSAADARLAILRHATSKIRTADDLYNINSLGFRGEALPSIAAVSRFTLTTRLHAEP

IGTYIEIQGGLVTDIREAGGSVGTTVTVSDLFFNTPARRKFLKTPATESAYIHDILGKIA

LARPDVAIKLINNKRQVLATTGTGRLFDAAASLYGYKAATELLPLDYTDGDIHITGYVGK

PHLLKSSRQWQTWTVNGRTISNRMLSKALDNAYHSLLPKNGFPLAIIQIVIPSDKVDVNV

HPQKNEVKFSDEQAVFRAVYKAVLSALETAKGPQQIAATVSLPASSPGKIAEEFKRTPEN

NVVPAALFMLKESNAPVWREETLPVTTVRETMQSATELPAIRYGLHQDTHDRSSLVLRVL

GQINDCFIVAAGSDGLYIIDQHAAHERILYEHLAQSAGRVPAQQLLVPRVVDFDERDIDL

ILQNEPLFYELGFRLEQIGPRSMRLLEVPSDIPGTEIEGLLREILSALHNMQTPKAHEIR

HIFLQTAACRSAVKAGENLNMRQMQALIDELCSTNRPYTCPHGRPAIVRFTSAELAKMFK

RQ

>A1HMZ7

MRRKKTCPIRIGNVIVGGNAPISVQSMTNTKTENVEATVAQIRRLAEAGCDIVRVAVPNM

AAAEAIESIKEQVSLPVVADIHFDYRLALAAIERGADALRLNPGNIREPEHVMAVVKQAK

KRRIPIRIGVNAGSLDPALLDKHGGHPTPEAMVESALQHVTILENLDFYDIKISLKANDV

PMTIEAYRLMSDTVDYPLHLGITEAGTVRSGIIKSAVGIGALLAEGIGDTIRVSLTGDPV

EEVRVGNEILKSLGLRSYGPTLVSCPTCGRCDIDLEKLAIQVEQRLSGIRKPIKVAVMGC

VVNGPGEAREADIGIAGGKGQGLVFRKGEIVKKVDEDELIPALFAELDRLIKEAE

>A1HNA4

MKIYTKTGDQGNTSLYTGERVAKDSLRVEAYGSIDETDAALGLARALCLKQEVKQAIYDM

QRMLWQLMADVASLGEKGSRITAEHVRELEQMIDRFDVMLPPLTKFVIPGDTPGSAALHV

ARTVARRAERQMWRLAREESVNEHVLVALNRLSDLCFVLSRVESEEAKEA

>A1HPN9

MATVFSELEQLLVYRKLLADVVIKKLMAFAKDSSWLQGELASDLITKAEELGLSGNLINS

YLIHLISFDNNVFSRTAEKTGGKVGASLLAAAAHDIAIFRQVFAASQQCGFDGIILNYTP

TYKRQDNHTAAVQEWLLAGADQYTAEQVAALLSGHYARYGYGEMASCKAFRWDKQKGLVG

VKHIDPIQLEDIVGYERQKKTLLRNTEAFLAGKPAHNVLLVGARGTGKSSSVKALANRYY

DAGLRLIEVTKNDLTDLPAIMNAARQWGKKFIVFIDDLSFEETEAGYKQLKSVMDGGVET

KPDNVLIYATSNRRRLIRETWQDRAGDEVHHQDTIHEKISLADRFGITLTYPSPNQDEYL

RIVEDIARKNNVDLPTVELRAQALRWEMAHSGRSGRIARQFVDDLISKISCG

>A1HSX0

MWPSMCRVIKTREFLPPASNISSNGVNLAVAHLFGAAAHFIGIYFMCCSPCHWCGASVTI

VRRNLHNHGGFLMKFELVLELMGDMALLAMAANFIGRNRYIASCAERPTTPRSWLTLTVI

FSVLSILGTYTGVPVEGALANTRLVGTLMGGIMGGPWVGLGIGFISGLHRYLIGGFTAEI

CGAATLLGGLMAGMARQKYGLHGINWKKAALLALAAEGLQKGMVLLLAKPFEAAWALEKA

IAVPTTVVTMLGTVVFMLILKDIKTEQELHGAKAAQLSLEIASRTLPFLRHGLTMESAQK

TAEIIFAFTGMDAVSISNREQVLAFVGKGADHHKPGEPIMTQSTKQTLISGMLHIVHTAQ

EQGCPVAGCPLQSSVVAPLVVNGAVIGTVKLNRTVPNGITEVDIRMAEGIAHLLSVQIEL

AEIDCQRKMREKAELKALQAQINPHFLFNTINIIMSFCRTNPDTARSLLGHLATMLRHSF

ADRQDFVTLKEEMEGIAAYLEIVKARFGSRLTVKTEIDPILLEAPIPLLTLQPLVENAVQ

HGLFPKLSHCVLTIAAGLRDGAMVIEVRDNGVGIPVEKLRTILAGQGQGIGIRNVYKRLT

GIYGKEYGLAIESTLGQGTTVRIVIPYEGRDLAHAG

>A1HTV6

MASKGIIARVLPNSIASELELVPGDRLLAVNGKPVQDIIDLSFALAEETVELLVEKANGE

QELIEIEKDYNEDLGIEFESAVFDQVRRCANRCIFCFVDQMPPGMRGSLYVKDDDYRLSF

LYGNFITLTNLGPRDLERIRRFHLSPLYVSVHATDGDVRQKMLGNKRAGQIMQQLRDLID

AGVELHTQVVLCPNINDGLILEKTIADLAELYPHVLSLAIVPVGLTRFREGCYPLKSFTA

DQAAAIINTVHHWQRRFQQQWGTAFVYLADEFYLAAGHPIPEYDLYGDFPQLENGVGIVR

AFLAEWEKQTTDGAAYAEKHYIDVVCGVSAAKIIEPLLADLKIPNLYTRVVAVENIFFGP

SVTVTGLLTGQDILTALRRLPGPRTGVIIPGVALRKGEAVFLDDLTPDKLAQELGTAVRT

AYFAKDLHHLLTAWR

>A1HTY7

MSFSAEVKNELARVAGDNQCCHLAELAALMRMGGAVSIGGNKNLGINFTTENAAVARKVL

ALIKRGFSLKTEVVVTRGRRLKKNNAYHIKVLPSPVVAELLAALGIMKGDSINVGRDSGM

LRKACCRRAYLRGAFLGGGSVNRPEGDYHLELVTGNLDFAKTLVRLLKSFGLPGRLTDRK

GDYIVYLKDGEAITSFLRIIGAHSALMEFENVRVVKDMRNKVNRLVNCETANLQKTVNAA

LRQVENIEFIARTIGLDKLPPSLKEAAEARLAYREATLQELVDALDGRVSKSGMNHRLRK

LEQIARELRGEAP

>A1HU25

MTSRRTLVLLVFLLIAAFSAGCFRSAPDTNAPPKPQVGILDVQKAVKAHPKYAELQRRQQ

ELNTLLAQVEAEKQQTAQPSARPTLPDGAAGLDEALAREFDARVAAKQAEIKARLDAKTA

EVREELHRQLAAYTQEVDKEYYPQIFSLQLKLKTVQLTKDEMEALQKQHDNLQSERAAKI

AAKERQLAAELEKRMAPEQNAAEQELAAYAEQLNAAYAQQAAAKKLEMAQRLPGGQTSAP

AAGGPRTSAEQQAALKRTEIKVLQDFIHKDIADKAAKAAGERGLTAVLTTYKVNISAVDI

TDAVIAEFKK

>A1HU36

MKTIGLLAGVGRLPVEFARAARGMGFTVIAVAVVGGVDDELAAAADKLYTIGIGEVGKII

NTLKAEGVKEVTMLGKVTKELMFSGAVRLDERAQRLLAGLKDNSDDTIMLAFVRELAAEG

IGVLDQTAFIRSLMPAPGTLTKREPTPAERADMEFGYAMARQIGGLDIGQTVVVKNKAVM

AVEAIEGTDACIRRGGALGRGGVTVAKVAKPNQDMRFDVPAVGVGTLEAMIEAGATALVI

EAGKTLVVDRERVVALADQHNITIVAM

>A1HU98

MTNLSEQTMMFRVDNEENNAAAFIINTVYQALKEKGYNPINQMVGYLLSGDPTYITSHNN

ARGLIRKLERDELLEELVRAYLKDK

>A1IHL6

MMTNVQRMIQMNKKSLHFLLLKRKVLGIAFNLPEGEEFTFHDLNSRANVTCSKEVQQNVG

RWFAYFVKHAPRVPFIIIGKDTHGHLVYLKTGPNPHHNSNPSKGGVR

>A1INQ6

MNLDLTAQKVRLSWKDILWGYGNKYLGWADVAAYARKMTLSDHDERVFKLSLINKSNILE

LKPVLEDLASEMRDYSPKNWLYVLLSDVFHRKEEFEDPLGEVEKIYADFDYPEEIESFVR

YMPPKDGYIPSAHTYEENIARLYSHWEHYLNNGGGQG

>A1INV8

MRYTIKTFQTLADETFPSKNFKILSFNGVARPITIVCPTHGVQTVSAGQAFIRSKHGCPA

CGLVLSTSVLASRGRSVSILDTATGETLSFPSVQAAAKALNTSYGSIRTKLDGRSSPDNL

VCNRYKVMP

>A1IQ50

MNKTLKRRVFRHTALYAAILMFSHTGGGGGAMAQTRQYAIIMNERNQPEVQWNGSYSIKD

KDRKREYTHHNHQQGGSSVSFNNSDELVSRQSGTAVFGTATYLPPYGKVSGFDAAALKER

NNAVDWIHTTHPGLIGYSYDGVVCRSATDCPKLVYKTRFSFDNPDLAKTGGGLDKHTEPS

RDNSPIYKLKDHPWLGVSFNLGAEGIAKNGKTINKLVSSFNEKNSNNNLVYTTEGRDISL

GNWQRETTAMAYYLNAKLHLLDKKQIQNITDKTVQLGVLKPSIDVRTRNTGTAGILSYWA

KWDIKDTGQIPVKLSLTQVKAGRCVNKDNPNKNTKTSSPALTAPALWFGAGQDGKAEMYS

ASVSTYPDSSSSRIFLQNLKRKTDTSRPGRYSLATLNKSDIESREPSFTSRQTVIRLDGG

VQQIKLDRNNTEVTGFNGNDGKNDTFGIVSEGSFMPDASEWKKVLLPWTVRAFNYDGRFN

TVNKEENNGKPKYSQKYRSRNNGKHERNLGDIVNSPIVAVGEYLATSANDGMVHIFKQSG

GDKRSYNLKLSYIPGTMPRKDIESKDSTLAKELRAFAEKGYVGDRYGVDGGFVLRRITDD

QDKQKHFFMFGAMGLGGRGAYALDLTKADDNDPTKASLFDVKDNGNNGNNGNNRVELGYT

VGTPQIGKTHNGKYAAFLASGYATKQIDSGENKTALYVYDLESNNGTLIRKIEVTDGKGG

LSSPTLVDKDLDGTVDIAYAGDRGGKMYRFDLSGNNPNSWTVRTIFQGTKPITSAPAISQ

LKDKRVVIFGTGSDLSEDDVLSTDEQHIYGIFDNDTNTGTAQEGLGKGLLEQKLSEENKT

LFLTDYKRSDGSGDKGWVVKLKDGQRVTVKPTVVLRTAFVTIHKYTGNDKCGAETAILGI

NTADGGKLTKKSARPIVPAANSKVAQYSGDKKTSSGKSIPIGCMEKDGGTVCPNGYVYDK

PVNVRYLDEKKTDGFSTTADGDAGGSGTFKEGKKPARNNRCFSGKGVRTLLMNDLDSLDI

TGPMCGMKRISWREVFY

>A1IQL3

MPAETTVSGAHPAAKLPIYILPCFLWIGIVPFTFALRLQPSPDFYHDAAAAAGLIVLLFL

TAGKKLFDVKIPPISFLLFAMAAFWYLQARLMNLIYPGMNDIVSWIFILLAVSAWACRSL

VAHYGQERIVTLFAWSLLIGSLLQSCIVVIQFAGWEDTPLFQNIIVYSGQGVIGHIGQRN

NLGHYLMWGILAAAYLNGQRKIPPALGAICLIMQTAVLGLVNSRTILTYIAAIALILPFW

YFRSDKSNRRTILGIAAAVFLTALFQFSMNTILETFTGIRYETAVERVANGGFTDLPRQI

EWRKALAAFQSAPIFGHGWNSFAQQTFLINAEQHNIHDNLLSNLFTHSHNIVLQLLAEMG

ISGTLLVAATLLTGIAGLLKRPLTPASLFLICTLAVSMCHSMLEYPLWYVYFLIPFGLML

FLSPAEASDGIAFKKAANLGILTASAAIFAGLLHLDWTYTRMVNAFSPATDDSAKTLNRK

INELRYISANSPMLSFYADFSLVNFALPEYPETQTWAEEATLKSLKYRPHSATYRIALYL

MRQGKVAEAKQWMRATQSYYPYLMPRYADEIRKLPVWAPLLPELLKDCKAFAAAPGHPEA

KPCK
